# FoodAllergenSensitization in a Five-Year Allergy Clinic Cohort and Food Allergy Knowledge, Attitudes and Behaviours in Romanian Adults: Two Observational Studies

**DOI:** 10.3390/nu18203324

**Published:** 2026-10-10

**Authors:** Valentin-Cristian Iovin, Diana Lighezan, Andreea-Adriana Neamțu, Corina Porr, Anca-Maria Căpraru, Cristian Sarău, Iulius Jugănaru, Adriana Muntean, Meda-Ada Bugi, Carmen Panaitescu

**Affiliations:** 1 Doctoral School Department, “Victor Babeș” University of Medicine and Pharmacy, 300041 Timișoara, Romania; valentin.iovin@umft.ro; 2 Department of Hematology, “Victor Babeș” University of Medicine and Pharmacy, 300041 Timișoara, Romania; lighezan.diana@umft.ro; 3 Department of Toxicology, “Victor Babeș” University of Medicine and Pharmacy, 300041 Timișoara, Romania; 4 Research Centre for Pharmaco-Toxicological Evaluation, “Victor Babeș” University of Medicine and Pharmacy, 300041 Timișoara, Romania; 5 Department of Pathology, Clinical County Emergency Hospital of Arad, 310037 Arad, Romania; 6 Department of Pathology, “Pius Brînzeu” Clinical County Emergency Hospital, 300723 Timișoara, Romania; 7 Allergology Department, Faculty of Medicine, “Lucian Blaga” University, 550169 Sibiu, Romania; 8 Doctoral School, University of Medicine and Pharmacy of Craiova, 200349 Craiova, Romania; anca.mitran@umfcv.ro; 9 Department of Pharmacy, Poiana Mare Psychiatry Hospital, Gării Str. No. 40, 207470 Poiana Mare, Romania; 10 Medical Semiology I, Department V Internal Medicine I, “Victor Babeș” University of Medicine and Pharmacy, 300041 Timișoara, Romania; csarau@umft.ro; 11 Department XI Pediatrics, Discipline I Pediatrics, “Victor Babeș” University of Medicine and Pharmacy, 300041 Timișoara, Romania; juganaru.iulius@umft.ro; 12 Department of Pediatrics I, “Louis Țurcanu” Children’s Emergency Hospital, 300011 Timișoara, Romania; bugi.ada@umft.ro; 13 Department of Allergology and Clinical Immunology, “Iuliu Hațieganu” University of Medicine and Pharmacy, 400012 Cluj-Napoca, Romania; adriana.muntean@umfcluj.ro; 14 Department of Pharmacy, University of Medicine and Pharmacy “Vasile Goldiș”, 310025 Arad, Romania; 15 Department III Functional Sciences, Physiology Discipline, “Victor Babeș” University of Medicine and Pharmacy, 300041 Timișoara, Romania; cbunu@umft.ro; 16 Centre of Immuno-Physiology and Biotechnologies (CIFBIOTEH), “Victor Babeș” University of Medicine and Pharmacy, 300041 Timișoara, Romania; 17 OncoGen Centre, “Pius Brînzeu” Clinical County Emergency Hospital, 300723 Timișoara, Romania

**Keywords:** food allergy, food sensitization, anaphylaxis, adrenaline auto-injector, allergen labelling, health literacy, elimination diet, skin prick test, cross-sectional survey, Romania

## Abstract

**Background/Objectives:** Food allergy is reported far more often than it is confirmed by testing. Both ends of that gap matter nutritionally: dietary restriction adopted without confirmation, and unpreparedness for severe reactions. We characterized each end in two cohorts from one source population. **Methods:** Study 1 retrospectively analyzed 4404 patients assessed in a single allergy service over 2020–2024, with food sensitization being a positive skin prick test and/or specific IgE to at least one food allergen. Study 2 surveyed 500 adults by convenience sampling, using a 27-item questionnaire with a 10-point knowledge score. In Study 1, testing was indication-driven, and the food-tested subset could not be identified, so all Study 1 figures are proportions among patients assessed, not prevalence estimates. Analyses reported effect sizes with confidence intervals throughout, applied Holm–Bonferroni adjustment to the confirmatory comparisons, and used multivariable regression. **Results:** Food sensitization was documented in 185 of 4404 patients (4.2%; 95% CI 3.6–4.8) and concentrated in early childhood (13.05% at 0–5 years), age being the only independent demographic predictor (adjusted OR for 0–5 vs. 18–30 years: 4.61; 95% CI 2.87–7.41)—an association confounded with the indication to test. Of food-sensitized patients with a recorded severity score, one third (34.1%) had no active symptoms at assessment. In the community cohort, 17.6% (95% CI 14.5–21.2) self-reported a food allergy, but only 6.6% (95% CI 4.7–9.1) reported one as test-confirmed—a gap of 11.0 percentage points (95% CI 8.5–14.0). The mean knowledge score was 5.08/10: 70.2% knew anaphylaxis can be fatal, yet only 47.8% named adrenaline as first-line treatment, 33.4% had never heard of an auto-injector, and 15.4% always read allergen labels. Educational level and health-sector training were the strongest predictors of knowledge. **Conclusions:** Food allergy is labelled more readily than it is verified, while preparedness for severe reactions is thin. Better access to diagnostic confirmation and public information stratified by educational attainment are needed. The two cohorts share a source population but are differently selected and measure different constructs, so their proportions are complementary rather than interchangeable.

## 1. Introduction

Adverse reactions to food form a heterogeneous group in which the decisive distinction is between immunologically and non-immunologically mediated events. Under the harmonized nomenclature of the World Allergy Organization, subsequently updated by the European Academy of Allergy and Clinical Immunology (EAACI), food hypersensitivity denotes reproducible reactions triggered by a food, food allergy being the subgroup in which an immunological mechanism is involved, whereas food intolerances—lactose intolerance being the paradigmatic example—rest on non-immunological, predominantly enzymatic, pharmacological or toxic mechanisms [1,2]. The distinction is not merely academic. Confusion between allergy and intolerance shapes what people eat, how often they consult a physician, and how prevalence data should be read [3,4].

Mechanistically, food hypersensitivity reactions are classified according to the involvement of immunoglobulin E (IgE) as IgE-mediated, non-IgE-mediated (predominantly cell-mediated), or mixed [3,5]. IgE-mediated reactions begin rapidly, usually within minutes to two hours of exposure, and range from urticaria and angioedema to acute gastrointestinal, respiratory and cardiovascular involvement, culminating in anaphylaxis. Non-IgE-mediated reactions are delayed and predominantly gastrointestinal, and include food protein-induced enterocolitis syndrome, food protein-induced allergic proctocolitis and food protein-induced enteropathy [5,6]. Eosinophilic oesophagitis is a chronic, food-antigen-driven type 2 inflammatory disease in which the mechanism is regarded as predominantly non-IgE-mediated [5,7].

Prevalence estimates vary considerably with the method of assessment, which is itself a major public health problem. Meta-analyses commissioned by the EAACI document a systematic discrepancy between self-reported prevalence, which is considerably higher, and prevalence confirmed by oral food challenge, the reference standard, which lies at approximately 1% [8]. For the eight major allergens, lifetime self-reported prevalences span a wide range and are generally higher in children than in adults [9,10]. The EuroPrevall study demonstrated marked geographical differences in sensitization and food allergy across European centres using standardized methodology [11], and large population studies outside Europe confirm both a substantial gap between self-reported and probable IgE-mediated allergy and a considerable adult burden [12]. Data for Romania are limited; a recent cross-sectional study of schoolchildren in Cluj-Napoca reported a self-reported food allergy prevalence of approximately 9%, with milk, egg, strawberries and tree nuts most frequently implicated, and identified gaps in school staff awareness of pupils’ medical histories [13].

This heterogeneity reflects a well-defined methodological hierarchy: prevalence declines progressively from self-reported allergy, to sensitization demonstrated by specific IgE or skin prick testing, to symptoms plus sensitization, and finally to challenge-confirmed allergy [8,11]. Sensitization is considerably more frequent than clinically expressed disease, so a positive test in the absence of clinical reactivity justifies neither a diagnosis of allergy nor an elimination diet [3]. Both under-diagnosis and over-diagnosis are documented, and both are harmful: the first exposes patients to severe reactions, the second imposes dietary restriction, nutritional risk and anxiety without benefit [3,12].

The nutritional dimension of this problem is frequently underestimated. Elimination diets, particularly those targeting staple foods such as milk or wheat, can compromise the intake of essential nutrients and impair growth in children, which is why guidelines recommend dietetic involvement and nutritional monitoring [14]. At the population level, the ability to make safe food choices depends on allergen labelling, harmonized in the European Union by Regulation (EU) No 1169/2011, which requires the declaration of fourteen allergen categories [15]. A labelling framework, however, protects only those consumers who can read and interpret it, which makes food and health literacy an integral part of allergen risk management [16]. In parallel, hospital admissions for food-induced anaphylaxis have risen in several European countries over recent decades without a parallel rise in mortality [17,18], and access to adrenaline auto-injectors remains unequal across countries [19], so that community preparedness is as much a determinant of outcome as clinical care.

Against this background, two questions can be asked of one and the same population, through two different cohorts drawn from it. How frequently, and in whom, is food sensitization identified when it is objectified by testing in patients who actually reach out to an allergy service? And how does the general population perceive, understand, and act on food allergy? The present work addresses both, in two cohorts drawn from a single source population: a five-year retrospective clinical cohort and a cross-sectional community survey. That shared origin is what makes the pairing informative rather than merely adjacent: what the clinic records and what the community believes describe one population rather than two unrelated settings, even though the two cohorts view it through different windows—the clinical one dominated by young children, the survey confined to adults. Local data on either question are scarce in Romania. The specific objectives were (i) to estimate the proportion of patients assessed in an allergy service who are sensitized to food allergens, and to describe their demographic, temporal and clinical profile; (ii) to characterize co-sensitization patterns and the associated clinical phenotypes; (iii) to estimate the proportion of adults who self-report a food allergy and to compare it with the proportion reporting a test-confirmed allergy; (iv) to measure knowledge of food allergy, including recognition of anaphylaxis and of its first-line treatment; (v) to identify the socio-demographic determinants of that knowledge; (vi) to describe declared attitudes and behaviours—label reading, emergency response and healthcare-seeking—and the information sources the population relies on; and (vii) to compare declared confidence in recognizing a severe reaction with measured knowledge.

## 2. Materials and Methods

### 2.1. Study Design

The work comprises two complementary observational studies conducted in Romania. Study 1 is a retrospective, descriptive and analytical study of patients assessed in a hospital allergy service, in which the exposure of interest—food allergen sensitization—was objectified by allergy testing. Study 2 is a cross-sectional, questionnaire-based study of adults, in which food allergy was self-reported and knowledge was measured by an objective score. The two studies draw two different cohorts from the same source population: a clinical cohort of people who presented for allergological assessment, and a community cohort of adults sampled from the general population. Because both draw on one source population, their findings bear on one another, although the clinical signal is concentrated in an age stratum—young children—that the adult survey does not sample. Because the cohorts are selected differently, one by the act of seeking specialist assessment and the other by convenience sampling, and because they measure different constructs, sensitization documented by testing on one side and self-reported allergy on the other, their proportions are not interchangeable and are never substituted for one another in what follows. Both are reported with reference to the STROBE recommendations for observational studies [20].

### 2.2. Study 1: Retrospective Allergy Clinic Cohort

#### 2.2.1. Setting and Participants

The study was based on the electronic database of an allergy service and included all patients assessed between 1 January 2020 and 31 December 2024. Inclusion criteria were presentation for allergological assessment during the study period and the availability of testing allowing classification of sensitization status. Incomplete records were excluded. For patients with multiple presentations, a single record per patient was retained so that the unit of analysis was the patient rather than the visit. After de-duplication, the analyzed cohort comprised 4404 unique patients, of whom 2234 had at least one documented sensitization (to aeroallergens, food allergens, drugs or insect venom) and 2170 were non-sensitized.

#### 2.2.2. Definitions and Variables

Sensitization status was established using the tests routinely performed in the service—skin prick tests with commercial extracts and/or determination of allergen-specific serum IgE—according to the department’s standard protocols. Food sensitization was defined as a positive test result to at least one food allergen; the present analysis did not distinguish the individual food involved. Testing in routine practice is indication-driven rather than systematic, so the proportions reported here are proportions positive among patients assessed in the service and not population prevalences; the implications of this are set out in the limitations. In particular, the records did not allow identification of the subset of patients who actually underwent food-specific testing, so the number tested for food allergens is unknown and the reported proportions could not be re-expressed against that more informative denominator. It must also be emphasized that sensitization documented by testing is not equivalent to food allergy confirmed by oral food challenge, and that a diagnostic strategy based on skin prick tests and specific IgE detects only IgE-mediated sensitization. For these reasons, Study 1 outcomes are described throughout as sensitization rather than food allergy, and no clinical diagnosis is implied by them.

The primary variable of interest was food allergen sensitization, coded as a binary variable. Independent variables comprised sex, age in completed years, year of presentation, sensitization to the main aeroallergen classes (tree pollen, grass pollen, ragweed pollen, mugwort pollen, house dust mites, animal dander and moulds), sensitization to drugs and to insect venom, and the associated clinical manifestations (rhinitis, conjunctivitis, bronchial asthma, asthma exacerbation and urticaria/dermatitis). A global symptom severity score, assigned in routine practice on a 0–3 scale, was also recorded; it was missing for a proportion of patients and is reported with its missingness. For analysis, age was grouped into seven categories (0–5, 6–12, 13–17, 18–30, 31–45, 46–60 and 61 years or older), and patients were classified as pediatric (under 18 years) or adult. Patients were considered food-only sensitized when no other sensitization was documented, and co-sensitized when one or more additional sensitizations accompanied the food sensitization.

### 2.3. Study 2: Cross-Sectional Population Survey

#### 2.3.1. Participants and Sampling

The target population comprised adults (aged 18 years or over) drawn from the same source population as the clinical cohort of Study 1. Sampling was by convenience, using mixed distribution: an electronic form circulated through online platforms and social networks, and printed questionnaires distributed in public spaces and in the waiting rooms of medical practices, in order to include population segments with limited digital access. Individuals aged at least 18 years who gave informed consent were included; questionnaires incomplete in essential sections were excluded. The final analyzed sample comprised 500 respondents.

#### 2.3.2. Questionnaire

The instrument was a structured, self-administered questionnaire of 27 items, comprising closed questions (dichotomous or multiple choice) and a small number of free-text fields, organized in six sections: (A) socio-demographic data; (B) self-reported food allergy; (C) general knowledge of food allergy; (D) recognition of manifestations and of anaphylaxis; (E) attitudes and behaviours in the face of a reaction; and (F) information sources and healthcare-seeking. Mean completion time was approximately 7–10 min.

Items were developed from the core concepts described in the introduction and from comparable instruments described in the literature [21,22], and covered the allergy–intolerance distinction, the allergens subject to mandatory labelling, the reactivity threshold, the thermal stability of allergens, the recognition of anaphylaxis and the role of adrenaline. Wording deliberately avoided technical terms so as not to confer an artificial advantage on respondents with medical training. Knowledge items had a single correct answer or, for multiple-choice items, a predefined set of correct options, allowing objective scoring. Distractor items were included, such as “headache without other symptoms” among the manifestations of an allergic reaction. For the item on allergens subject to mandatory declaration, a correct answer required selection of the exact complete set of regulated allergens without inclusion of the distractors (sugar, water), which explains the very low proportion of correct answers to this item and reflects the genuine difficulty the public has in discriminating regulated allergens.

#### 2.3.3. Knowledge Score

The principal dependent variable was the level of knowledge, operationalized as a score obtained by summing correct answers to nine objectively scored questions from sections C and D and to the item on management of a severe reaction, giving one point per correct answer and a maximum of 10 points. The item assessing declared familiarity with the auto-injector was not scored, as it measures self-reported familiarity rather than knowledge. Multiple-response items (Q12 and Q16) were scored all-or-nothing: a point required selecting the exact predefined correct set with no distractor (for Q12, the six allergens subject to mandatory declaration offered among the options; for Q16, the five true manifestations offered, with the isolated-headache distractor excluded). This deliberately conservative criterion was chosen because partial recall of the regulated allergen list, or of the manifestation spectrum, offers incomplete protection in practice; its consequences are quantified in the Results. The score was reported as a raw value and as a percentage of the maximum, and was categorized into three levels: low (score below 5), intermediate (5–7) and high (8 or above). Because all-or-nothing scoring makes the two multiple-response items much harder than the rest, a sensitivity analysis repeated the main knowledge analyses on an eight-item score omitting them. For the analysis of information sources, respondents were grouped by age into 18–24, 25–34, 35–44, 45–59 and 60 years or over. Calibration was assessed by the Spearman rank correlation between declared confidence and the knowledge score, by comparing mean scores across the four confidence levels, and by reporting the proportion of the most confident respondents who nonetheless scored below half marks. Independent variables considered were age, sex, educational level, area of residence, training in a medical or health-related field, and a family history of food allergy; these six were the predictors entered in the multivariable model. Internal consistency was assessed with Cronbach’s alpha.

### 2.4. Statistical Analysis

All statistics reported in this article were computed directly from the individual-level datasets provided as Supplementary Materials, in Pythonversion 3.13.16 (Python Software Foundation, Wilmington, DE, USA) using the open-source libraries pandas (version 3.0.5), NumPy (version 2.5.3), SciPy (version 1.18.1) and statsmodels (version 0.15.0); figures were produced with Matplotlib (version 3.11.2)*.* Continuous variables are described by mean and standard deviation (SD) and by median and range; categorical variables by absolute and relative frequencies. For all key proportions, 95% confidence intervals (CI) were computed by the Wilson score method, which is preferred to the normal approximation at the small subgroup sizes encountered here. In both studies, proportions were compared between groups by the chi-squared test with Yates’ continuity correction for 2 × 2 tables, and means by Student’s *t* test for independent samples. Each confirmatory comparison of proportions is reported with an effect size—the odds ratio (OR) with a 95% CI obtained by the Woolf method on the log scale—and the principal mean difference with its 95% CI and Cohen’s d; for tables larger than 2 × 2, Cramér’s V accompanies the chi-squared statistic. The comparisons of clinical manifestations across the sensitization groups of Study 1 were treated as its confirmatory analyses and their *p* values adjusted for multiplicity by the Holm–Bonferroni procedure, the family being the set of outcomes compared within each of the three groupings; adjustment was applied within and not across those families, since panels A and B contrast overlapping subsets of the same 185 food-sensitized patients rather than independent samples. All remaining analyses in both studies are exploratory, and their *p* values are reported as nominal and unadjusted.

In Study 1, the Cochran–Armitage test for trend was applied both to the annual proportion sensitized across the five years and to the gradient across ordered age groups, using age-group midpoints as scores (2.5, 9, 15, 24, 38, 53 and 70 years, the open-ended top group being scored at 70); heterogeneity between age groups was tested separately by a 7 × 2 chi-squared test. Seasonality was tested on the proportion of assessed patients who were food-sensitized in each season (a 4 × 2 chi-squared test), which uses each season’s own attendance as its denominator, in addition to a goodness-of-fit test on the raw presentation counts; a sex-by-year interaction was examined in an exploratory logistic model. Multivariable logistic regression identified the factors independently associated with food sensitization; because the age–sensitization relation was expected to be non-linear, age was modelled categorically (the seven age groups defined above, with 18–30 years as reference), and this specification was tested against a model with age entered as a continuous term scaled per 10 years by a likelihood-ratio test. Model discrimination was quantified by the area under the receiver operating characteristic curve (AUC), with a 95% CI from 2000 bootstrap resamples, and calibration by the Hosmer–Lemeshow test over deciles of predicted risk. In Study 2, means were additionally compared across more than two subgroups by one-way analysis of variance (ANOVA), and multiple linear regression identified independent predictors of the knowledge score, whose coefficients are reported both in score points and standardized in units of the score’s standard deviation. Self-reported and self-reported-as-test-confirmed allergy are nested categories reported by the same individuals, so the difference between them is quantified descriptively, as an absolute gap with a 95% CI for paired proportions computed by Tango’s score method, rather than by a significance test, whose result would be forced by the nesting. For the pair of objectively scored knowledge items whose within-person discordance structure could not be recovered, the McNemar statistic is reported as the conservative lower bound implied by the observed marginal difference, which holds for every admissible discordance structure. Internal consistency is reported as Cronbach’s alpha with a 95% CI computed by the Feldt method. The threshold for statistical significance was set at *p* < 0.05, and all tests were two-sided.

### 2.5. Ethical Considerations

Both studies were approved by the Ethics Committees of the “Pius Brînzeu” County Emergency Clinical Hospital and of the “Victor Babeș” University of Medicine and Pharmacy, Timișoara (approvals no. 316 of 3 August 2022 and no. 70 of 28 May 2025, respectively). Study 1 used retrospective, anonymized data collected in the course of routine clinical activity, with no intervention on patients and no possibility of patient identification; patients had given informed consent upon admission, covering the use of their anonymized clinical data. In Study 2, participation was voluntary and anonymous and was preceded by informed consent given through explicit acceptance of participation; no identifying data were collected. Both studies were conducted in accordance with the Declaration of Helsinki and with data protection legislation.

## 3. Results

### 3.1. Study 1: Food Allergen Sensitization in the Allergy Clinic Cohort

#### 3.1.1. Cohort Characteristics and Overall Proportion Sensitized

The cohort comprised 4404 unique patients, 2428 female (55.1%) and 1976 male (44.9%), with a mean age of 29.1 years (SD 19), reflecting a predominantly adult population with a substantial pediatric component. At least one sensitization was documented in 2234 patients (50.7%). The general characteristics of the cohort are summarized in Table 1. Male patients were younger on average than female patients (25.5 versus 32.0 years; *p* < 0.001) and more often pediatric (41.9% versus 26.7% aged under 18 years; *p* < 0.001), a compositional difference relevant to the sex contrasts reported below; sex-stratified characteristics are shown in Table 1.

Sensitization to at least one food allergen was identified in 185 patients, 4.2% (95% CI 3.6–4.8) of all patients assessed. Referred to the sensitized subpopulation (*n* = 2234), food sensitization accounted for 8.3% (95% CI 7.2–9.5), confirming that food allergens are a minority but non-negligible cause of sensitization among patients presenting to an allergy service. Throughout this section, such figures are proportions positive among patients assessed in the service, not population prevalences. The number of patients who underwent food-specific testing could not be reconstructed from the records, so the 4.2% carries no meaning as a prevalence estimate and most plausibly understates the proportion positive among patients actually tested for foods.

#### 3.1.2. Distribution by Sex

Of the 185 food-sensitized patients, 102 were male and 83 female. Related to the size of each sex stratum, 5.16% of men (95% CI 4.3–6.2) and 3.42% of women (95% CI 2.8–4.2) were sensitized to food. The difference was statistically significant (χ^2^ = 7.80; *p* = 0.0052), male sex being associated with approximately 1.5-fold higher odds of documented food sensitization (OR 1.54; 95% CI 1.14–2.07). The effect size was nonetheless small (φ = 0.04), so sex accounts for only a marginal share of the variation in sensitization status.

#### 3.1.3. Distribution by Age

**Figure 1:**
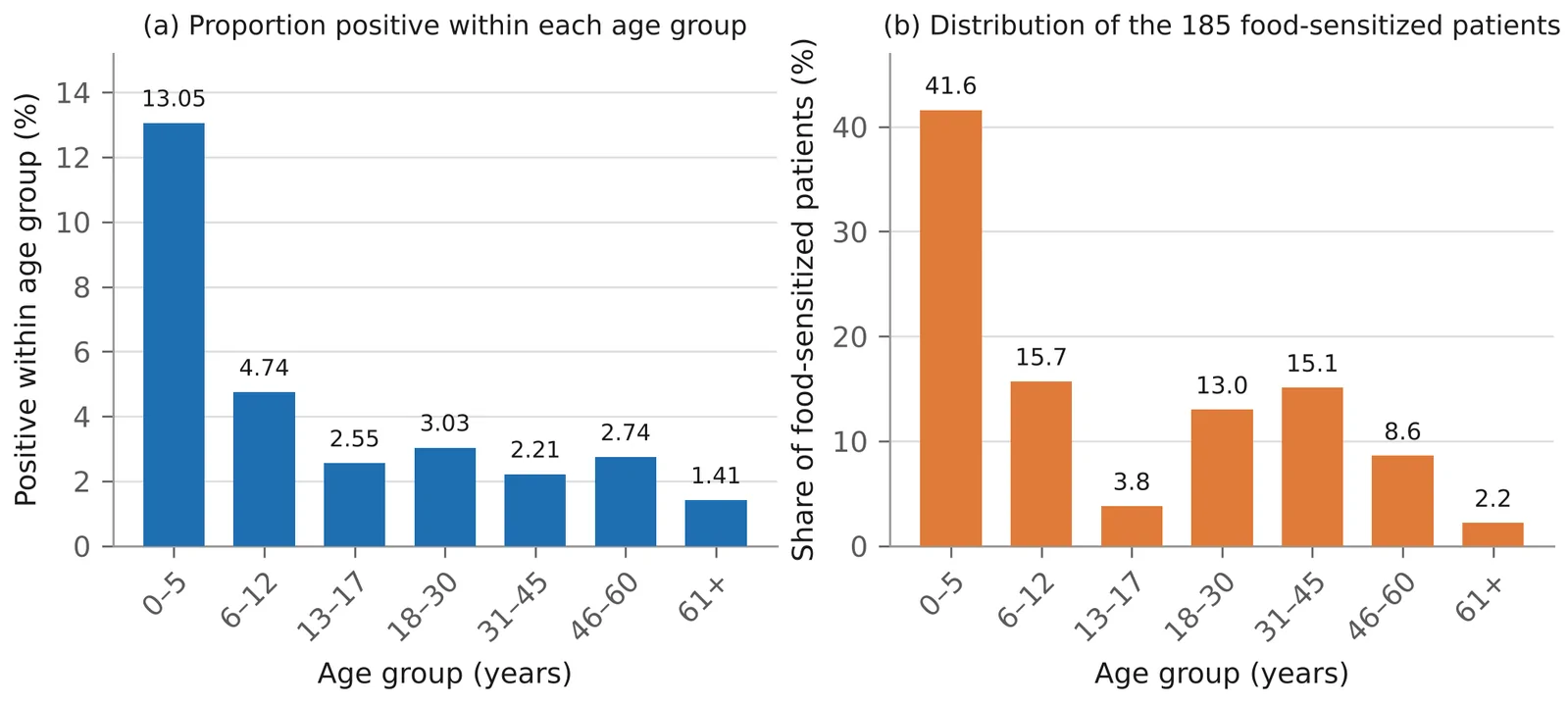
Food allergen sensitization by age group in the allergy clinic cohort: (**a**) proportion positive within each age group; (**b**) distribution of the 185 food-sensitized patients across age groups.

The mean age of food-sensitized patients was 17.8 years (SD 18.5), with a median of 9 years and a range of 0–76 years, significantly lower than that of the rest of the cohort (29.6 years, SD 18.9; mean difference 11.8 years; 95% CI 9.0–14.5; t = 8.28; *p* < 0.001; Cohen’s d = 0.62, a moderate effect). Food sensitization was markedly concentrated in the pediatric categories: 113 patients (61.1%) were under 18 years of age. The highest within-group proportion was recorded in children aged 0–5 years, where 13.05% (95% CI 10.6–16.0) of those assessed were food-sensitized, the proportion declining broadly with age, to 1.41% in patients over 60 years (Table 2, Figure 1a,b). The proportion sensitized differed markedly across the seven age groups (χ^2^ = 140.9; *df* = 6; *p* < 0.001; Cramér’s V = 0.18). Partitioning that heterogeneity shows that the age effect is not a simple linear decline: the Cochran–Armitage linear component was highly significant (z = −8.14; trend χ^2^ = 66.2 on 1 *df*; *p* < 0.001) but accounted for less than half of the total heterogeneity, the departure from linearity being larger still (χ^2^ = 74.7 on 5 *df*; *p* < 0.001). The pattern is therefore better described as a sharp excess in the youngest children than as a gradient: 13.05% at 0–5 years against 2.83% in all older patients combined (OR 5.15; 95% CI 3.79–7.00; *p* < 0.001), while the decline across the remaining groups was shallow and not strictly stepwise, the 18–30 and 46–60 groups sitting marginally above their immediate juniors. Dichotomized at 18 years, the proportion positive was 7.65% (113/1477; 95% CI 6.4–9.1) among pediatric and 2.46% (72/2927; 95% CI 2.0–3.1) among adult attenders, more than threefold higher odds in children (OR 3.29; 95% CI 2.43–4.45; *p* < 0.001) (Figure 1a,b). The 0–5 year group also accounted for the largest share of all food-sensitized patients (41.6%).

#### 3.1.4. Annual Evolution, 2020–2024

**Figure 2:**
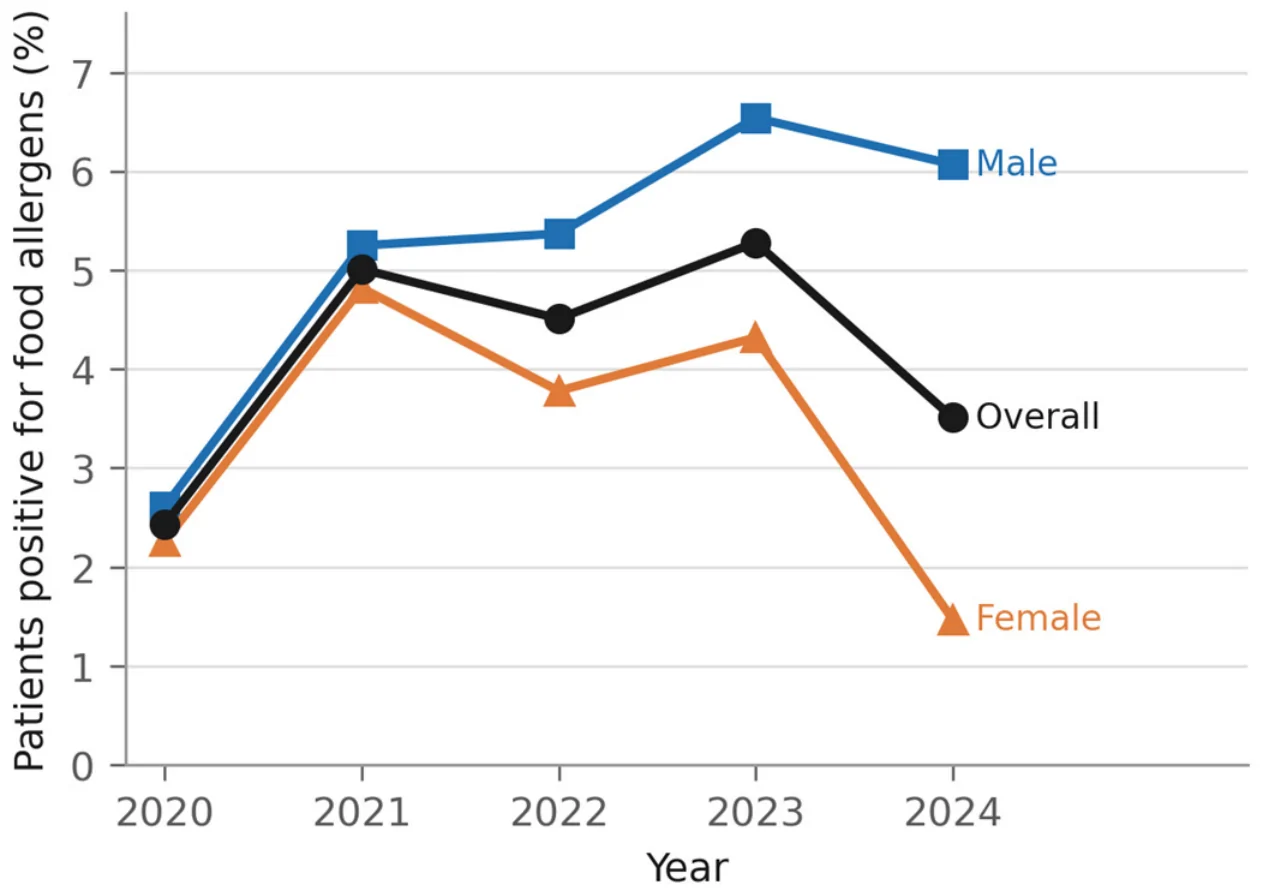
Annual proportion of assessed patients with food allergen sensitization, overall and by sex, 2020–2024. An exploratory sex-by-year interaction was nominally significant (*p* = 0.023, unadjusted).

The annual number of food-sensitized patients ranged from 19 (2020) to 47 (2021). Expressed as a proportion of patients assessed that year, food sensitization was 2.43% in 2020, 5.01% in 2021, 4.51% in 2022, 5.28% in 2023 and 3.51% in 2024 (Table 3, Figure 2). The low value for 2020 coincided with the lowest annual attendance of the five years (782 patients, against 853–976 in each subsequent year), consistent with reduced use of medical services during the pandemic. Although the proportion was higher over 2021–2023, the trend test did not demonstrate a statistically significant temporal trend across the period as a whole (z = 0.96; *p* = 0.338). The sex-specific series diverged in the final years—in men the proportion rose from 2.61% in 2020 to 6.07% in 2024, while in women it fell from 4.32% in 2023 to 1.47% in 2024. An exploratory logistic model found a nominally significant sex-by-year interaction (β = 0.25 per year; *p* = 0.023, unadjusted), consistent with the diverging series; the annual strata carry fewer than 30 events each, however, so this should be read as hypothesis-generating. The overall male excess is addressed by the multivariable model in Section 3.1.10.

#### 3.1.5. Co-Sensitization Profile

**Figure 3:**
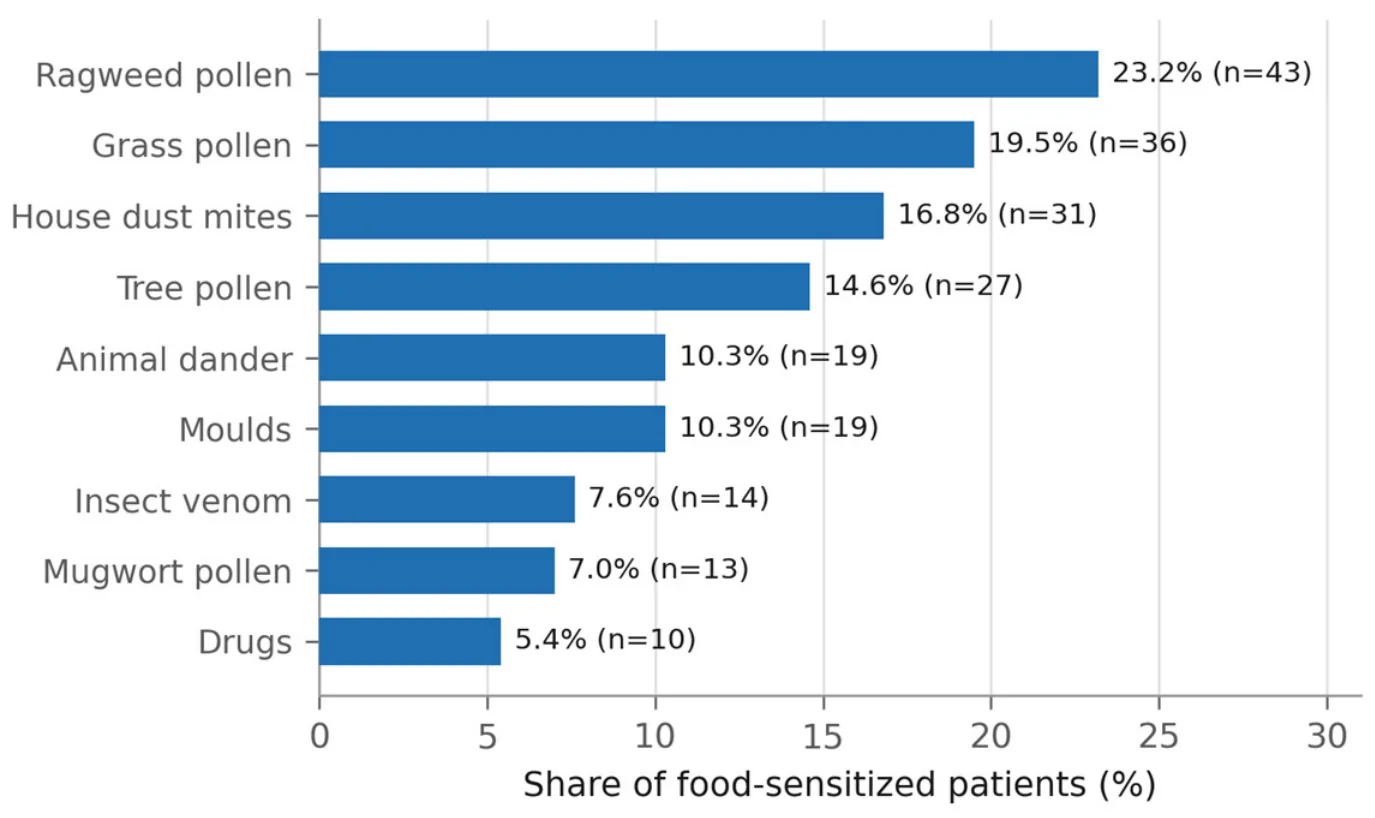
Co-sensitization profile of patients with food allergen sensitization (*n* = 185). Patients could have more than one co-sensitization.

Almost half of the food-sensitized patients—91 of 185 (49.2%)—had food-only sensitization, with no other documented sensitization. The remaining 94 patients had at least one additional sensitization, most frequently to aeroallergens. The most frequent co-sensitizations were to ragweed pollen (*n* = 43), grass pollen (*n* = 36) and house dust mites (*n* = 31), followed by tree pollen (*n* = 27); associations with insect venom (*n* = 14) and drug sensitization (*n* = 10) were less common (Table 4, Figure 3). Patients co-sensitized to any pollen (*n* = 63) were on average older than those without pollen co-sensitization (21.3 versus 16.0 years), a difference that did not reach significance (t = 1.88; *p* = 0.062).

#### 3.1.6. Associated Clinical Manifestations

Among food-sensitized patients, the most frequent associated clinical manifestation was rhinitis (51.9%; *n* = 96), followed by urticaria/dermatitis (37.8%; *n* = 70) and conjunctivitis (24.9%; *n* = 46). Bronchial asthma was present in 16.8% (*n* = 31) and asthma exacerbations in 5.9% (*n* = 11). The contrast that follows is partly definitional, because the comparison group is composed predominantly of aeroallergen-sensitized patients, in whom respiratory and ocular disease is expected; it is reported descriptively and interpreted with that caveat in Section 4.3.

**Figure 4:**
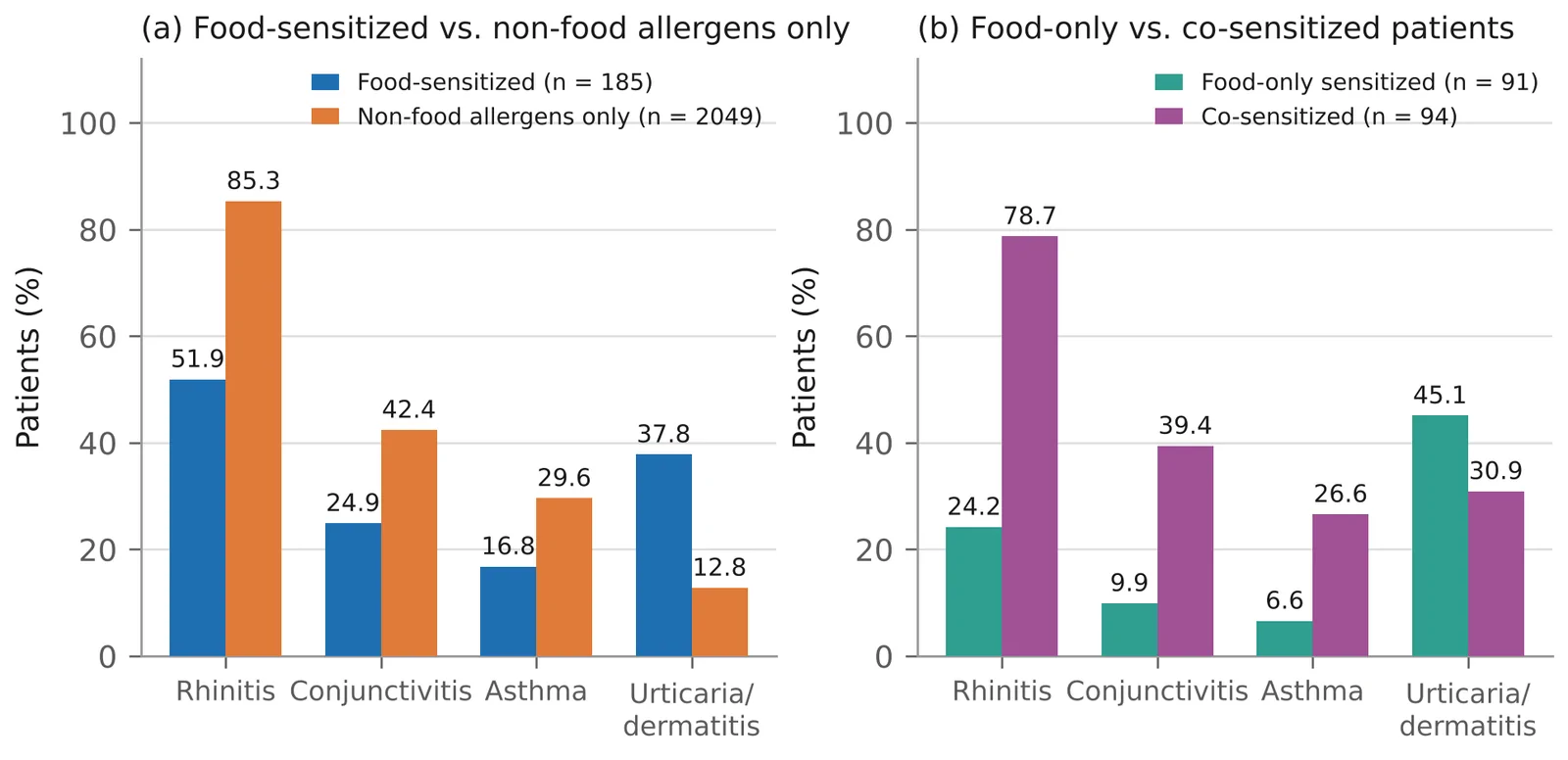
Associated clinical manifestations: (**a**) food-sensitized patients versus patients sensitized to non-food allergens only; (**b**) food-only sensitized versus co-sensitized patients. Odds ratios with 95% confidence intervals and Holm-adjusted *p* values for these comparisons are given in Table 5.

Compared with patients sensitized to non-food allergens only (*n* = 2049), the clinical profile of food-sensitized patients was displaced towards cutaneous manifestations. Urticaria/dermatitis was almost three times more frequent (37.8% versus 12.8%; OR 4.15; 95% CI 3.00–5.74), while every respiratory and ocular manifestation was substantially less frequent: rhinitis (51.9% versus 85.3%; OR 0.19; 95% CI 0.14–0.25), conjunctivitis (24.9% versus 42.4%; OR 0.45; 95% CI 0.32–0.63) and asthma (16.8% versus 29.6%; OR 0.48; 95% CI 0.32–0.71). All four differences remained significant after Holm–Bonferroni adjustment (adjusted *p* < 0.001 throughout) (Table 5, panel A; Figure 4a). This contrast supports the clinical observation that food sensitization is expressed predominantly through the skin. The respiratory contrast should, however, be read with the comparator group in mind: patients sensitized exclusively to non-food allergens are aeroallergen-sensitized by definition, so part of their excess of rhinitis and asthma reflects the way the group was constituted. The excess of urticaria/dermatitis among food-sensitized patients carries no such circularity.

The distribution of food-sensitized patients by global severity score, computed from the individual records, was as follows: 56 patients (30.3%; 95% CI 24.1–37.3) had no significant symptoms recorded at the index assessment (score 0), 33 (17.8%) scored 1, 74 (40.0%) scored 2, one (0.5%) scored 3, and for 21 patients (11.4%) no severity score was recorded. Among the 164 patients with a recorded score, 34.1% (95% CI 27.3–41.7) were symptom-free. That roughly one third of patients with documented food sensitization carried no active symptoms at assessment is consistent with clinically silent sensitization, although absence of symptoms at a single assessment does not establish tolerance—patients may be avoiding the trigger or between episodes—and the 21 unscored patients add further uncertainty in the direction of either group. The severity score refers to the index assessment, whereas the manifestation variables may reflect clinical history, so the two sets of figures should not be read as describing the same time frame. Among the components of rhinitis symptomatology, the most frequently present manifestation was nasal obstruction (38.9% of patients), followed by rhinorrhoea (31.4%), sneezing (30.3%) and itching (27.0%), values consistent with the degree of aeroallergen co-sensitization in this group.

#### 3.1.7. Food-Only Versus Co-Sensitized Patients

Comparison of the two subgroups revealed marked clinical differences (Table 5, panel B; Figure 4b). Food-only sensitized patients were on average younger (15.2 versus 20.4 years), a difference at the margin of statistical significance (*p* = 0.053), and had a profile in which cutaneous manifestations predominated. Respiratory and ocular manifestations were distinctly rarer in this group: rhinitis occurred in 24.2% versus 78.7% of co-sensitized patients (OR 0.09; 95% CI 0.04–0.17), conjunctivitis in 9.9% versus 39.4% (OR 0.17; 95% CI 0.08–0.38) and asthma in 6.6% versus 26.6% (OR 0.19; 95% CI 0.08–0.50), all three remaining significant after adjustment (adjusted *p* ≤ 0.001). Urticaria/dermatitis was more frequent among food-only sensitized patients (45.1% versus 30.9%; OR 1.84; 95% CI 1.01–3.35), but this difference did not reach significance once the continuity correction and the multiplicity adjustment were applied (adjusted *p* = 0.066).

#### 3.1.8. Pediatric Versus Adult Patients

Comparison of pediatric (under 18 years, *n* = 113) with adult patients (*n* = 72) revealed important differences in profile. In children, male predominance was pronounced (67.3% boys), whereas in adults the ratio was reversed (36.1% men); this was the largest of the differences between the two groups (OR 3.63; 95% CI 1.95–6.76; adjusted *p* < 0.001). Pediatric patients less often had any additional sensitization (44.2% versus 61.1%; OR 0.51; 95% CI 0.28–0.92), a less prominent respiratory profile (rhinitis 46.9% versus 59.7%, OR 0.60, 95% CI 0.33–1.08; asthma 12.4% versus 23.6%, OR 0.46, 95% CI 0.21–1.00) and a lower frequency of conjunctivitis (17.7% versus 36.1%; OR 0.38; 95% CI 0.19–0.75). After Holm–Bonferroni adjustment across the six comparisons, only male sex and conjunctivitis remained significant (adjusted *p* < 0.001 and *p* = 0.040); the other differences were consistent in direction but underpowered at these subgroup sizes and should be read as hypothesis-generating. Cutaneous manifestations were essentially identical in the two groups (37.2% versus 38.9%; OR 0.93; 95% CI 0.51–1.71) (Table 5, panel C). The respiratory and co-sensitization gradient is consistent with the natural history of the atopic march, in which the respiratory component and aeroallergen sensitization accumulate with age; the cutaneous component, by contrast, did not differ between the age groups.

#### 3.1.9. Seasonal Distribution of Presentations

The distribution of the 185 food-sensitized patients’ presentations by calendar month showed seasonal variation, with a maximum in the autumn months (September–November), when 62 presentations were recorded (33.5%; 95% CI 27.1–40.6), and a minimum in winter (36 presentations, 19.5%; 95% CI 14.4–25.8); spring accounted for 47 presentations (25.4%) and summer for 40 (21.6%). The raw presentation counts departed from an even seasonal distribution (goodness-of-fit χ^2^ = 8.49; *df* = 3; *p* = 0.037), but this test ignores attendance. When each season’s own attendance is used as the denominator, the proportion of assessed patients who were food-sensitized was essentially constant across the year—3.75% in winter (36/959), 4.29% in spring (47/1096), 4.13% in summer (40/969) and 4.49% in autumn (62/1380); χ^2^ = 0.80; *df* = 3; *p* = 0.85. The apparent autumn excess therefore reflects seasonal variation in overall clinic attendance, not in food sensitization, and no seasonal signal remains once the denominator is accounted for.

#### 3.1.10. Factors Independently Associated with Food Sensitization

To establish which demographic factors were independently associated with food sensitization, a multivariable logistic regression model was constructed with food sensitization as the dependent variable and age group, sex and year of presentation as predictors (Table 6). Categorical age fitted the data decisively better than a continuous linear term (likelihood-ratio χ^2^ = 32.1; *df* = 5; *p* < 0.001), confirming that the age association is not a uniform gradient but a threshold effect located in early childhood. Age was the only significant independent predictor: relative to adults aged 18–30, children aged 0–5 had 4.61-fold adjusted odds of food sensitization (95% CI 2.87–7.41; *p* < 0.001), whereas no other age group differed significantly from the reference—the odds in the 6–12-year group were elevated but compatible with chance (adjusted OR 1.52; 95% CI 0.87–2.65; *p* = 0.14), and estimates from adolescence onwards lay at or below unity. For comparability with a conventional continuous specification, a linear-age model gave an adjusted OR of 0.69 per additional 10 years of age (95% CI 0.63–0.76; *p* < 0.001), but the likelihood-ratio test shows this summary to be an oversimplification of the true age pattern. The male predominance observed in the univariate analysis (crude OR 1.54; *p* = 0.005) attenuated after adjustment for age and no longer reached significance (adjusted OR 1.24; 95% CI 0.92–1.69; *p* = 0.16), suggesting that the male excess is largely explained by the younger age structure of male patients. Year of presentation was not significantly associated with food sensitization (adjusted OR 1.05; 95% CI 0.94–1.17; *p* = 0.40), consistent with the absence of a temporal trend. The model was well calibrated (Hosmer–Lemeshow χ^2^ = 6.01; *df* = 8; *p* = 0.65) with 23 events per estimated parameter, and its discrimination (AUC 0.694; bootstrap 95% CI 0.652–0.735) is that expected of a model built from demographic variables alone: age and sex locate the risk, but they do not by themselves identify the sensitized patient.

### 3.2. Study 2: Knowledge, Attitudes and Behaviours in the General Population

#### 3.2.1. Sample Characteristics

The sample comprised 500 respondents, 297 women (59.4%), 195 men (39.0%) and 8 (1.6%) who preferred not to state their sex, with a mean age of 46.7 years. Most respondents lived in urban areas (62.2%). Educational level was distributed across all categories, with upper secondary education the most frequent (36.0%); 8.0% reported training in a medical or health-related field (Table 7).

#### 3.2.2. Self-Reported Versus Test-Confirmed Food Allergy

**Figure 5:**
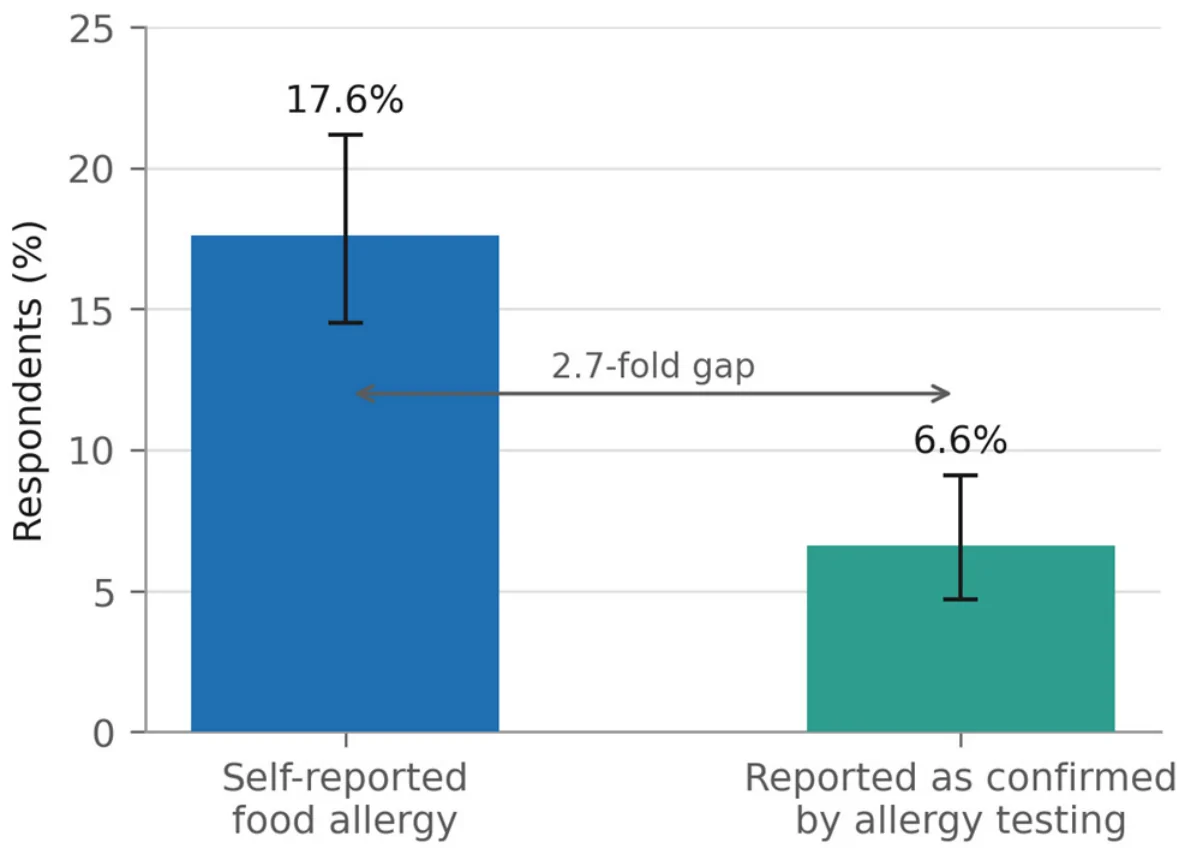
Self-reported food allergy versus food allergy reported as confirmed by allergy testing in the general population sample (*n* = 500). Error bars are 95% Wilson confidence intervals. Test confirmation is a nested subcategory of self-report, so the two bars are not independent.

A total of 88 respondents (17.6%; 95% CI 14.5–21.2) declared that they had a food allergy, and a further 40 (8.0%) were unsure. Of the 88 who self-reported an allergy, only 33 (37.5%) stated that it had been confirmed by allergy testing; 37 (42.0%) reported a physician diagnosis made on the basis of symptoms alone, without testing; and 18 (20.5%) relied on their own assessment (Table 8). Referred to the whole sample, food allergy reported as confirmed by allergy testing was present in only 6.6% (95% CI 4.7–9.1), against a self-reported prevalence of 17.6%—a ratio of 2.7. Because test confirmation is a nested subcategory of self-report, the 55 respondents who differed between the two categories necessarily all fell on the self-report side; a significance test would therefore be uninformative, and the quantity of interest is the size of the gap itself: 11.0 percentage points (95% CI 8.5–14.0, by Tango’s score method for paired proportions) (Figure 5).

A family history of diagnosed food allergy was reported by 116 respondents (23.2%), while 17.0% did not know. The item on incriminated foods was answered by the whole sample, including the 40 respondents unsure whether they had an allergy, and 372 (74.4%) named no food. Among those named, the most frequent were fruit and vegetables (44 respondents, 8.8% of the sample), milk (40, 8.0%) and tree nuts (33, 6.6%), followed by egg (22, 4.4%), fish (15, 3.0%), wheat/gluten (14, 2.8%), soy (13, 2.6%), shellfish (12, 2.4%) and peanut (11, 2.2%). The leading categories are separated by only a few respondents and their confidence intervals overlap almost entirely, so the ranking should not be over-read; fruit and vegetables is moreover a composite of many foods competing against single-food categories.

#### 3.2.3. Level of Knowledge

The mean knowledge score was 5.08 points out of 10 (SD 2.06; median 5; range 0–10), equivalent to 50.8% of the maximum. Only 68 respondents (13.6%; 95% CI 10.9–16.9) reached a high level of knowledge, while 205 (41.0%; 95% CI 36.8–45.4) were at a low level and the largest group, 227 (45.4%; 95% CI 41.1–49.8), at an intermediate level. Internal consistency, assessed by Cronbach’s alpha on the item-level data, was modest (α = 0.58; 95% CI 0.52–0.63), a value to be expected for a questionnaire spanning heterogeneous knowledge domains that had not been formally validated beforehand. An eight-item sensitivity score omitting the two all-or-nothing multiple-response items behaved almost identically (mean 61.1% of its maximum; α = 0.58; Spearman correlation with the full score 0.98), so the findings below are not artefacts of the strict scoring of those two items.

**Figure 6:**
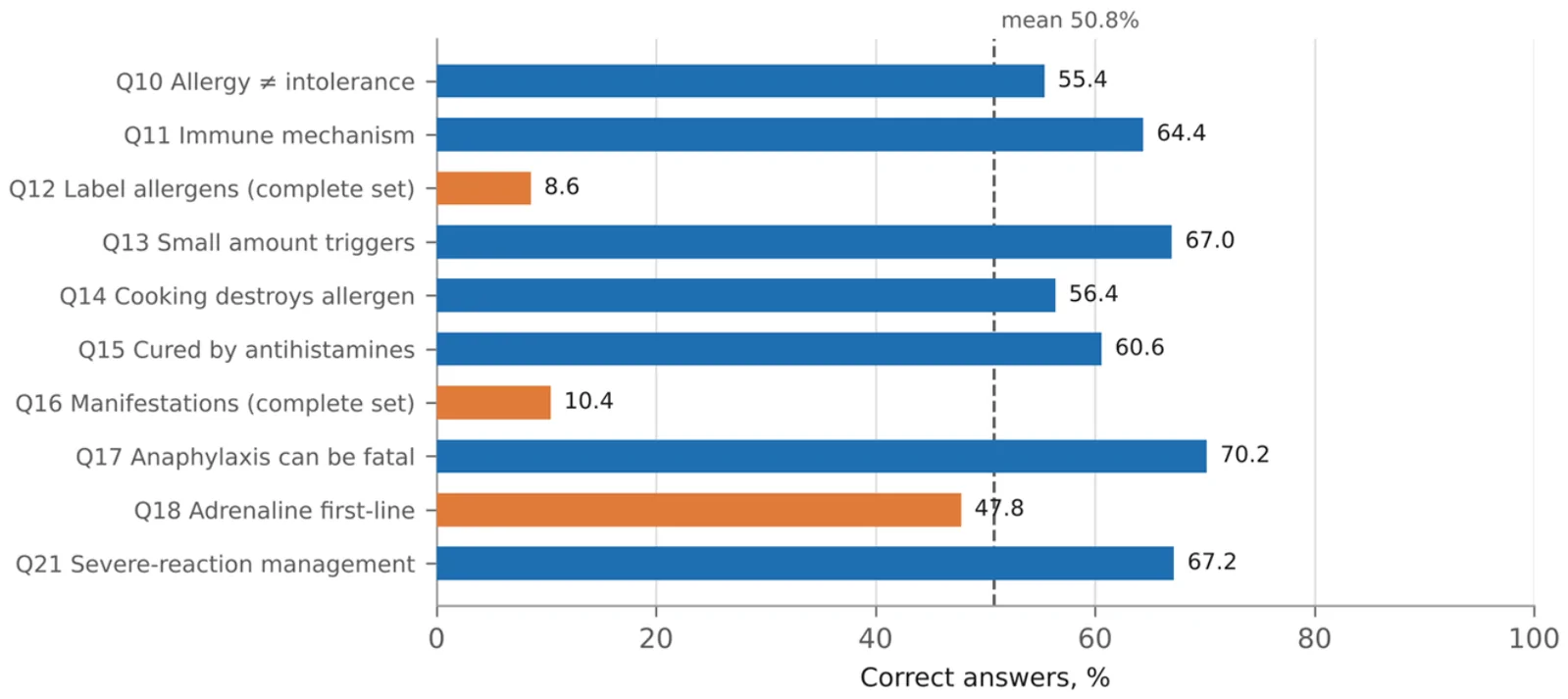
Proportion of correct answers to each knowledge item (*n* = 500). Items answered correctly by fewer than half of respondents are shown in orange; the dashed line marks the mean score of 50.8%. The two lowest items were scored all-or-nothing and therefore measure exhaustive recall rather than working familiarity.

Item-level analysis revealed important gaps (Table 9, Figure 6). That anaphylaxis can be life-threatening was recognized by 70.2% of respondents (95% CI 66.0–74.0), but only 47.8% (95% CI 43.5–52.2) correctly identified adrenaline as the first-line treatment of an anaphylactic reaction. As both items were answered by the same respondents, this 22.4-percentage-point drop is a within-person discrepancy, and it is highly significant under every discordance structure the observed marginals permit (McNemar χ^2^ ≥ 30.6; *p* < 0.001). The distinction between food allergy and food intolerance was correctly made by only 55.4% (95% CI 51.0–59.7), and complete identification of the regulated allergens offered by the questionnaire by only 8.6% (95% CI 6.4–11.4). This item and the item on the manifestations of an allergic reaction (10.4%; 95% CI 8.0–13.4) were the weakest results, and both were scored all-or-nothing—a point required the exact correct set (for labelling, the six regulated allergens listed among the options: milk, egg, peanut, gluten-containing cereals, shellfish and soy; for the manifestations item, the five true manifestations offered, with the isolated-headache distractor excluded) with no distractor selected. They therefore measure exhaustive recall rather than working familiarity and substantially understate practical knowledge: for example, 90.4% of respondents avoided the manifestation item’s distractor, 75.2% selected at least three, and 44.2% at least four, of its five correct manifestations. Correct management of a severe reaction (calling the emergency number or using adrenaline) was indicated by 67.2% (95% CI 63.0–71.2).

#### 3.2.4. Recognition of Anaphylaxis and Emergency Behaviour

**Figure 7:**
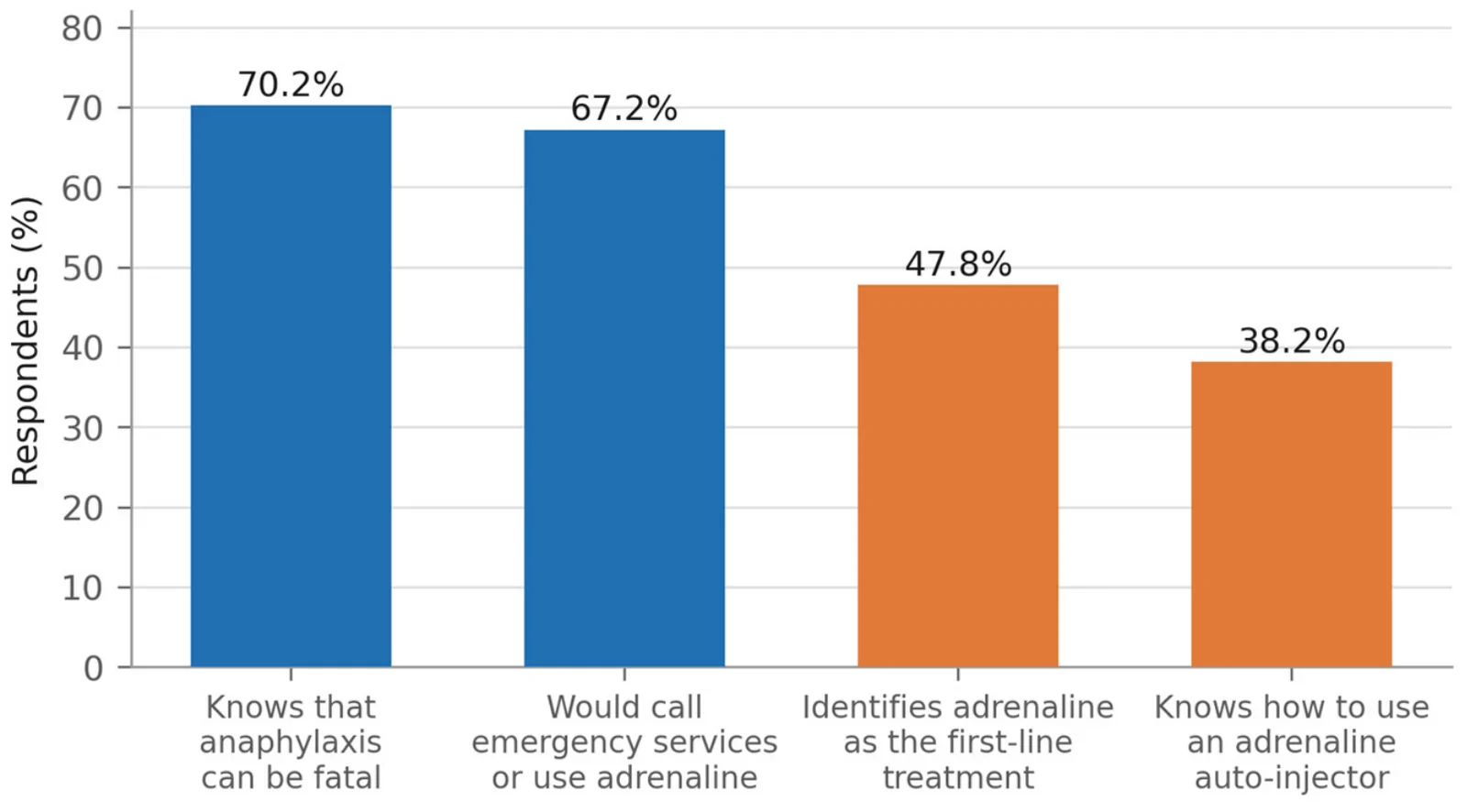
The anaphylaxis knowledge funnel: from recognition of the danger to knowledge of the concrete response (*n* = 500).

Familiarity with the adrenaline auto-injector was limited: 38.2% of respondents stated that they knew what it is and how to use it, 28.4% had heard of it without knowing how to use it, and 33.4% (95% CI 29.4–37.6) had never heard of the device. Set against the 70.2% who recognized that anaphylaxis can be fatal, this leaves a 32.0-percentage-point gap between knowing that the condition is dangerous and reporting the ability to use the device that treats it. The two items are not directly comparable as tests of knowledge—the first is objectively scored, the second is self-rated familiarity—so the difference is reported descriptively, but its direction and magnitude are consistent with the item-level gap described above. Faced with a severe reaction (difficulty breathing or facial swelling), 67.2% would have acted correctly, but a concerning proportion would have adopted inadequate management: 16.0% would have taken an antihistamine only and stayed at home, and 12.4% would have waited to see whether the reaction worsened. For a mild reaction, the predominant response was to take an antihistamine (59.0%) (Table 10). Taken in sequence, these figures describe a knowledge funnel that narrows from recognition of danger to knowledge of the concrete solution (Figure 7).

#### 3.2.5. Attitudes and Behaviours

Reading food labels to identify allergens was an inconsistent behaviour: only 15.4% stated that they always check labels, 38.0% do so sometimes, 26.6% only when they or someone close to them has an allergy, and 20.0% never. Regarding confidence in their own ability to recognize a severe allergic reaction, only 17.4% declared themselves very confident, while 42.4% considered themselves slightly or not at all confident. Only 29.4% felt they had sufficient knowledge to manage a food allergy, and interest in educational programs was high (71.4% answering yes). Label-reading was distinctly more prudent among respondents with a self-reported allergy: 30.7% of them (27/88) always read labels, against 12.1% (45/372) of those who reported no allergy (OR 3.22; 95% CI 1.86–5.57; χ^2^ = 17.2; *p* < 0.001; the 40 respondents unsure of their status are excluded from this comparison).

#### 3.2.6. Healthcare-Seeking and Information Sources

For a suspected food allergy, respondents would turn first to the family physician (48.0%) and only then to an allergist (33.4%); a minority would go to an emergency physician (6.6%) or a gastroenterologist (6.2%), and 5.8% would not consult a physician at all. As the principal information source, the internet and social networks exceeded the physician (32.8% versus 29.4%), followed by press and television (11.0%), pharmacy staff (9.6%) and family or friends (8.8%); 8.4% do not seek information on the subject (Table 10). A clear generational gradient was apparent: the use of the internet and social networks as the principal source declined with age, peaking at 46.9% in the 25–34 year group and falling to 24.6% among those aged 60 or over, while reliance on the physician rose across the same range, from 26.4% in the youngest respondents to 35.2% in the oldest.

#### 3.2.7. Determinants of the Knowledge Score

Educational level was strongly associated with the knowledge score (ANOVA: F = 67.17; *p* < 0.001), the mean score rising monotonically across all five levels: 2.88 (SD 1.54) with lower secondary, 4.30 (1.79) with upper secondary, 5.57 (1.85) with post-secondary, 6.11 (1.60) with university and 7.15 (1.13) with postgraduate education. Medical training was likewise an important factor: respondents with such training scored 7.60 (SD 1.10) on average versus 4.86 (1.98) for the remainder (difference 2.74; 95% CI 2.12–3.36; t = 8.62; *p* < 0.001; Cohen’s d = 1.42). Urban residence was associated with a higher score than rural residence (5.41 versus 4.53; difference 0.88; 95% CI 0.51–1.24; d = 0.43; *p* < 0.001), and a family history of allergy with a higher score (5.46 versus 4.97; difference 0.49; 95% CI 0.06–0.92; d = 0.24; *p* = 0.025). No difference was found between the sexes (5.00 versus 5.13; *p* = 0.50).

**Figure 8:**
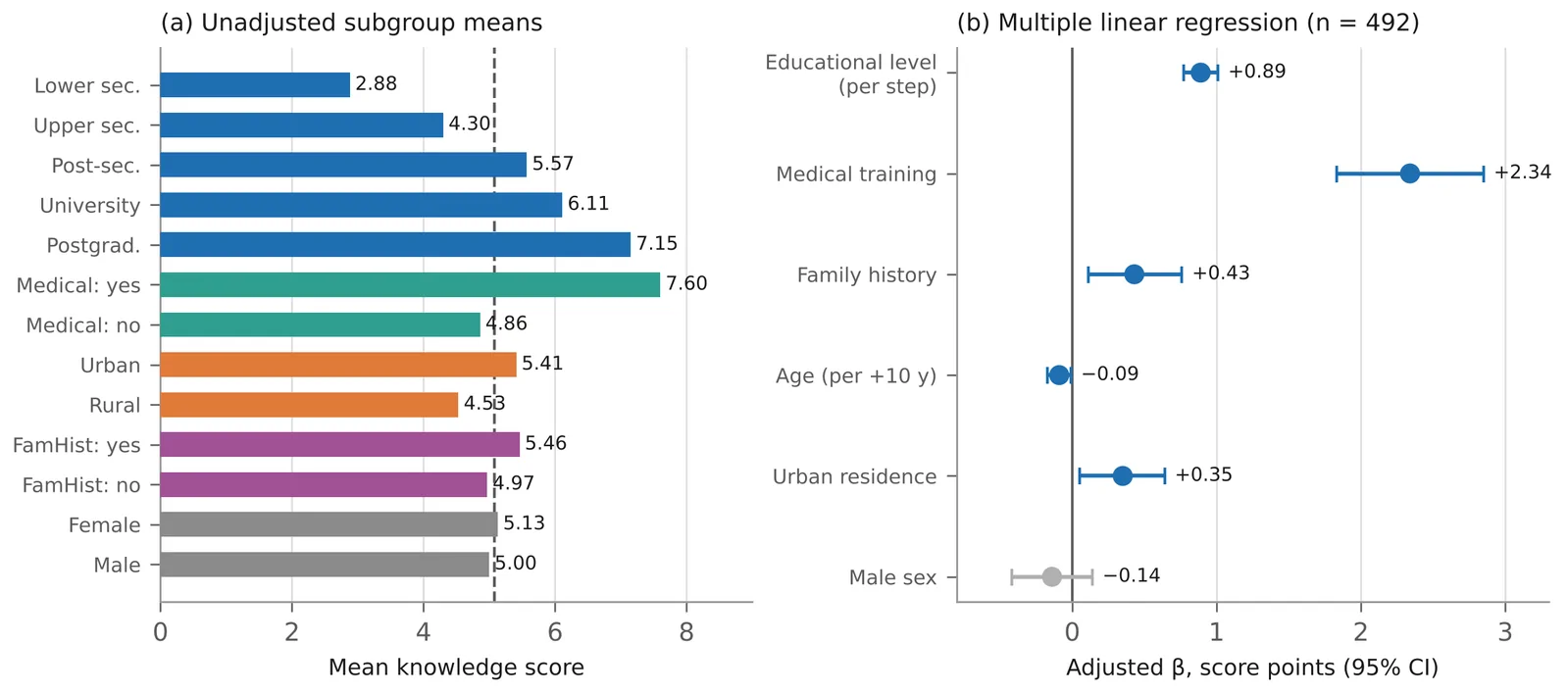
Determinants of the knowledge score: (**a**) unadjusted subgroup means, with the dashed line marking the overall mean of 5.08; (**b**) adjusted coefficients from the multiple linear regression model, with 95% confidence intervals (significant predictors in blue).

To separate the independent effects of these partly inter-correlated factors, a multiple linear regression model was constructed with the knowledge score as the dependent variable (Table 11, Figure 8a,b). The model explained 44% of the variance in the score (R^2^ = 0.44). Educational level (+0.89 points per educational step, equivalent to +0.43 SD; *p* < 0.001) and medical training (+2.34 points, or +1.13 SD; *p* < 0.001) remained by far the strongest independent predictors, the coefficient for medical training corresponding to more than a full standard deviation of the score. A family history of allergy (+0.43 points; *p* = 0.009) and younger age (−0.09 points per additional 10 years; *p* = 0.025) had smaller but significant effects. The urban–rural difference attenuated substantially after adjustment and retained only marginal significance (+0.35 points; *p* = 0.023), which did not survive in the eight-item sensitivity model (*p* = 0.086); educational attainment therefore accounts for much, though possibly not all, of the apparent urban advantage. Sex was not associated with the score (*p* = 0.32). Eight respondents who did not state their sex were excluded from the model, giving *n* = 492.

The linear age term should not be read as a simple gradient: descriptively, mean scores were highest at 25–44 years (5.43), lower at 45 years and over (4.93) and lowest among the youngest respondents (18–24 years: 4.60), so a single linear coefficient describes only part of the pattern.

#### 3.2.8. Knowledge in Relation to Allergy Status and Confidence Calibration

Respondents who self-reported a food allergy obtained a higher mean knowledge score than the remainder (5.98 versus 4.89 points; difference 1.09; 95% CI 0.62–1.55; t = 4.58; *p* < 0.001; d = 0.54); the direction of this association cannot be established from cross-sectional data, since better-informed respondents are also more likely to recognize and report an allergy. Self-reporting an allergy was also significantly more frequent among people with a family history of allergy (29.3% versus 14.1% of respondents without a known family history; OR 2.53; 95% CI 1.55–4.15; χ^2^ = 13.25; *p* < 0.001), a pattern compatible with both the hereditary component of the disease and heightened awareness in affected families.

Declared confidence in recognizing a severe reaction was only weakly related to measured knowledge (Spearman ρ = 0.14; *p* = 0.001). The mean score rose across the four confidence levels from 4.63 (SD 1.84) among respondents who were not at all confident to 4.87 (2.20), 5.11 (2.04) and 5.68 (1.88) among the very confident—a spread of less than one point on a ten-point scale—yet about one third (30/87; 34%) of those who declared themselves “very confident” scored below 5 out of 10. A similar picture emerged from the comparison of self-assessed knowledge with the actual score: respondents who felt “sufficiently” informed scored only marginally higher than those who acknowledged insufficient knowledge (5.24 versus 4.74 points), indicating that subjective perception of competence is only weakly correlated with the actual level of knowledge.

## 4. Discussion

### 4.1. Principal Findings

Three observations dominate these results. First, food allergen sensitization documented by testing was found in 4.2% of patients assessed in an allergy service and was overwhelmingly a condition of early childhood, age being the only independent demographic predictor. Second, of the sensitized patients whose severity was recorded, one third had no active symptoms at assessment—a pattern consistent with clinically silent sensitization, and a reminder, within the clinic itself, that a positive test is not by itself a diagnosis, although symptom-free status at one visit does not establish tolerance and a further 11% had no severity recorded at all. Third, in the adult population, self-reported food allergy exceeded self-reported test-confirmed allergy almost threefold, while knowledge of how to recognize and treat a severe reaction was deficient in a way that is directly relevant to survival. These are two cohorts from one population, differently selected and measuring different things, so their proportions cannot stand in for one another; read side by side, however, they converge on a single pattern: the label of food allergy is applied more readily than it is verified, and preparedness for the minority of genuinely dangerous reactions is correspondingly thin.

### 4.2. Sensitization Epidemiology in Context

Clinic-based proportions such as the 4.2% reported here depend heavily on referral pattern and on which patients are selected for food testing, and are therefore not readily compared across services; what can be said is that the figure is far below the rates of self-reported food allergy in European population studies, which frequently exceed 5–10% [8,9,10]. The age distribution—a marked concentration in the pediatric categories, with a maximum of 13.05% in children aged 0–5 years—is consistent with the known natural history of food allergy, which typically begins in the first years of life and tends to attenuate with age for certain foods [4]. The male predominance observed in the crude analysis reproduces a well-documented pattern at pediatric ages [23], but its effect size was small (φ = 0.04) and it disappeared after adjustment for age; taken with the fact that it was pronounced in children (67.3%) and reversed in adults (36.1%), this suggests that the male excess in this cohort is largely a consequence of the younger age structure of male patients rather than an independent effect of sex. Age, by contrast, was a strong signal however it was analyzed, though these are three views of one association in one dataset rather than three independent confirmations. Its shape matters more than its size: the effect is concentrated in the first five years of life rather than distributed as a smooth decline across adulthood—a conclusion the model comparison makes formal, since categorical age fitted the data decisively better than a linear term. That shape is compatible with the early-childhood onset described for the major food allergens [4], but it is not evidence for it—the present analysis could not identify the food involved, and a cross-sectional clinic series cannot distinguish resolution with age from cohort effects, from changing referral practice, or from the fact that infants presenting with suspected food reactions are far more likely to be tested for foods than adults referred for rhinitis. This last possibility is the most serious and is taken up in the limitations. The absence of a significant temporal trend across the five years, despite higher values in 2021–2023, should likewise be interpreted cautiously, given the reduced attendance in 2020 and the retrospective nature of the data.

### 4.3. Two Clinical Phenotypes

The clinical study distinguishes a food-only sensitized subgroup from a co-sensitized one, but the contrast must be read carefully. Its respiratory half is close to definitional: patients are placed in the co-sensitized group precisely because they carry additional sensitizations, overwhelmingly to aeroallergens, and aeroallergen sensitization is directly associated with rhinitis and asthma. Its cutaneous half—urticaria/dermatitis in 45.1% of food-only against 30.9% of co-sensitized patients—did not survive adjustment for multiple testing (adjusted *p* = 0.066), and the age difference between the groups was borderline (*p* = 0.053). What can be said is that food-only sensitized patients had markedly less respiratory and ocular disease; the suggestion that they represent a distinct classical childhood phenotype, and that co-sensitized patients represent pollen–food cross-reactivity, remains a hypothesis. Two observations might be read as supporting it, and the data bear neither out. The apparent autumn excess of presentations disappears entirely once each season’s attendance is used as the denominator: the proportion of assessed patients who were food-sensitized was flat across the year (3.8–4.5%; *p* = 0.85), so there is no seasonal signal to explain. Its peak months, October and November, in any case fall after the mid-August to late-September ragweed maximum recorded for this region [24]. The greater mean age of pollen-co-sensitized patients (21.3 versus 16.0 years; *p* = 0.062) did not reach significance and is in any case consistent with the accumulation of aeroallergen sensitization with age quite generally, not specifically with food cross-reactivity. There are also immunological reasons for caution. Pollen–food allergy syndrome (PFAS) is driven chiefly by birch-family PR-10 homologues and secondarily by mugwort [25,26]. Among the 94 co-sensitized patients in this cohort, 27 (28.7%) carried tree pollen sensitization and 13 (13.8%) mugwort, against 43 (45.7%) ragweed and 36 (38.3%) grass; the categories overlap, since a patient may carry several. The profile is therefore ragweed- and grass-dominant rather than birch-dominant, which is not the pattern a PR-10-driven PFAS population would show, although ragweed-associated food reactivity is described and cannot be excluded. Moreover, PR-10 and profilin allergens are heat- and protease-labile, so commercial extract-based skin prick tests and standard specific IgE panels have poor sensitivity for the plant foods involved; if PFAS were common here, extract-based testing would under-detect it by design. Confirming the mechanism would require oropharyngeal symptom data and molecular component testing, neither of which was available [27,28].

The one comparison in this group that is neither definitional nor borderline is the excess of urticaria and dermatitis among food-sensitized patients relative to those sensitized to non-food allergens only (37.8% versus 12.8%; OR 4.15). That contrast is robust and has practical value in orienting diagnostic hypotheses.

### 4.4. The Perception Gap and Its Nutritional Consequences

Within the survey, 17.6% of respondents self-reported a food allergy but only 6.6% reported one confirmed by allergy testing, a gap of 11.0 percentage points (95% CI 8.5–14.0). This is a within-cohort contrast and is the legitimate one to draw. The clinic figure of 4.2% is not set against it here or anywhere in this paper: although the two cohorts come from the same source population, the clinical one is filtered by the act of seeking specialist assessment and reports sensitization documented by testing, while the community one reports allergy as the respondent perceives it, so the two proportions answer different questions. The gap is consistent with observations in the European literature [8,9] and indicates the scale of unverified diagnosis—pure self-assessment in one fifth of self-reporters and an untested clinical diagnosis in a further two fifths—and of the frequent confusion between allergy and intolerance—a confusion also evidenced by the fact that fewer than two thirds of respondents correctly distinguished the two concepts. Two qualifications bound the interpretation. The self-reported figure of 17.6% sits above the 5–10% range usually reported in European population surveys and above the roughly 9% found in Romanian schoolchildren [13], which is what convenience sampling in medical waiting rooms would be expected to produce. And the 6.6% is itself a self-report of having been tested, not a verified diagnosis; since even a genuine positive test demonstrates sensitization rather than clinical allergy, and since respondents may have been given unvalidated intolerance panels, the 6.6% likely overestimates verified allergy among those who report it, and it is not itself a prevalence of confirmed allergy.

Why does the label so consistently outrun its verification? The survey itself points to several convergent mechanisms. The most direct is diagnostic conflation: fewer than two thirds of respondents distinguished allergy from intolerance, so any adverse food experience—enzymatic intolerance, functional gastrointestinal symptoms, simple aversion—can plausibly be filed by the respondent under “allergy”. Second, part of the gap is generated within the healthcare system itself: two fifths of self-reporters described a physician diagnosis made on symptoms alone, without testing, so unverified labels are not only self-assigned but also medically conferred, and such labels are rarely revisited where access to allergology services and to oral food challenge is limited—delays and constraints in access to specialist care pathways have been documented in Western Romania for other conditions as well [29]. Third, the information environment reinforces the label: the internet and social networks were the principal information source in this sample, and the same channels carry direct-to-consumer “food sensitivity” testing of no diagnostic validity, whose positive results can anchor a lasting conviction. Fourth, labels acquired in childhood may simply persist: a substantial share of early-life IgE-mediated allergy resolves with age [4], but the label seldom expires with it unless re-testing occurs. Finally, expectation consolidates belief—once a food is suspected, symptoms on exposure are more readily noticed and attributed to it, and the apparent confirmation further discourages formal testing. These mechanisms are not mutually exclusive, and a cross-sectional design cannot apportion their relative weights; what the data do show is that the unverified fraction is large, and that its strongest correlates—education and health-sector training—are precisely the levers most accessible to public-health intervention.

The nutritional stakes of that overestimation are what make it more than a bookkeeping problem, and they must be stated for what they are: an inference from the literature rather than a finding of this work. Neither study collected dietary data—no avoidance items, no intakes, no anthropometry—so we can say nothing directly about what our respondents eat. What is established elsewhere is that an unverified label of food allergy translates into avoidance behaviour, that avoidance which is not clinically warranted narrows the diet without conferring protection, and that when the avoided foods are staples such as milk or wheat the nutritional cost can be appreciable in children, which is why current management guidelines couple allergen avoidance with dietetic counselling and nutritional monitoring [14]. Set against those findings, the size of the unverified fraction in our sample—three respondents in five who claim a food allergy have no test to support it—identifies a population in which unnecessary restriction is plausible and unmeasured. The practical corollary is that access to accurate diagnosis is itself a nutritional intervention, since it is what allows unwarranted restriction to be lifted while genuine risk is respected; quantifying the restriction actually undertaken is the obvious next study.

### 4.5. The Anaphylaxis Knowledge Gap and Allergen Label Literacy

The mean level of knowledge (50.8% of the maximum) conceals gaps of vital relevance. The most concerning is the discrepancy between recognition of anaphylaxis as a life-threatening condition (70.2%) and knowledge of its first-line treatment: only 47.8% identified adrenaline, and one third of respondents had never heard of the adrenaline auto-injector. A population that knows the danger but not the remedy is at real risk of mismanaging allergic emergencies outside clinical settings, and the deficit compounds the documented inequality in the availability of auto-injectors between European countries [19,30].

In parallel, only 15.4% of respondents always read food labels for allergens. The corresponding knowledge item is weaker evidence than it appears—its all-or-nothing scoring demands exhaustive recall of the full regulated set offered—so the 8.6% should not be read as the population’s label literacy. The behavioural finding stands on its own, however, and suggests that the European consumer protection instrument, the labelling regulation [15], is underused in practice [16,31]. A labelling rule confers protection only to the extent that consumers can locate, read and act on the information it mandates; measured that way, our data suggest that a substantial part of that protection is not currently realized in this population.

Declared confidence tracked measured knowledge only weakly (Spearman ρ = 0.14): mean scores rose by less than one point across the full range of confidence, and one third of the most confident respondents scored below half marks. Confidence is therefore a poor proxy for competence in this domain, which matters because people who believe themselves prepared are less likely to seek information or help. Respondents with a self-reported allergy or a family history were better informed and read labels more systematically; whether experience produces the knowledge or knowledge produces the report cannot be determined from these data.

### 4.6. Implications for Practice and Policy

Three implications follow. First, better diagnostic differentiation between allergy and intolerance is needed, through facilitated access to allergy testing and to oral food challenge, in order to reduce both over-diagnosis and under-diagnosis, and with it both unnecessary dietary restriction and unrecognized risk. Second, the robust cutaneous excess among food-sensitized patients relative to those sensitized to non-food allergens only (OR 4.15)—the one clinical contrast in this series that is neither definitional nor borderline—can orient diagnostic hypotheses in day-to-day assessment. Third, educational interventions should be prioritized by educational level and by absence of health-sector training—the two strongest predictors our regression identifies—and only secondarily by area of residence, whose apparent advantage was largely—though in the adjusted model not entirely—accounted for by educational attainment, and should be adapted in format by age group: digital channels for younger people, the physician as intermediary for older people. Their content should centre on the recognition of anaphylaxis, the use of the adrenaline auto-injector and the practical reading of allergen labels. The preference of respondents for the internet and social networks over the physician as principal information source carries a double significance: a risk of exposure to unverified information—of which dietary attribution is a staple example: common skin conditions such as acne are widely blamed on particular foods in lay sources, although their determinants are predominantly hormonal and immune, including described associations with autoimmune thyroiditis, rather than dietary [32]—but equally an opportunity to use those same channels for scientifically validated campaigns. The high level of interest in educational programs (71.4%) confirms that the population is receptive to such initiatives.

One dietary intervention in this field does have first-line evidence behind it. The LEAP trial showed that early oral introduction and regular consumption of peanut in infants at high risk substantially reduces the risk of peanut allergy [33], and the EAACI prevention guideline accordingly recommends introducing well-cooked egg and peanut in the first year of life as part of complementary feeding, rather than delaying them [34]. That recommendation speaks directly to the population in which our clinical study found sensitization concentrated: more than two in five food-sensitized patients were under six years of age. It also sets a boundary on the message this paper carries. The case for avoiding unverified restriction in older children and adults is a case about diagnosis; the case for timely introduction in infancy is a case about prevention, and the two should not be conflated when communicating with parents.

## 5. Strengths and Limitations

The strengths of this work lie in the size of the clinical series (4404 unique patients over five consecutive years), the size of the population sample (*n* = 500), the fact that every reported statistic was computed from the individual-level data published alongside the article and is therefore independently reproducible, and the multidimensional approach of the survey, which goes beyond measuring knowledge to include attitudes, behaviours, the relation of confidence to competence, and the healthcare-seeking pathway, allowing concrete target groups for intervention to be identified.

Several limitations must be acknowledged, and the first is the most consequential. In routine allergy practice, testing is indication-driven: a young child presenting with a suspected food reaction is far more likely to be tested against a food panel than an adult referred for seasonal rhinitis. Because the records available did not allow the food-tested subset to be identified, the denominator of every proportion reported for Study 1 is all patients assessed rather than all patients tested for foods. The figures are therefore proportions positive within a service population, not prevalences, and the age association—although large and consistent across analyses—is confounded with the indication to test and should be regarded as hypothesis-generating rather than established. A sensitivity analysis restricted to patients with a documented food panel would settle this and is the first thing we would recommend to anyone replicating the work. Second, the series is single-centre, which restricts generalizability beyond the service studied. Third, sensitization was documented by skin prick tests and specific IgE rather than by oral food challenge, the diagnostic gold standard [3]; some sensitized patients are clinically tolerant, a possibility consistent with—though not established by—the third of scored patients with no active symptoms at assessment; the severity score was moreover unrecorded for 11% of food-sensitized patients. Fourth, an IgE-based definition detects no non-IgE-mediated food allergy at all—not food protein-induced enterocolitis syndrome, allergic proctocolitis or enteropathy, nor non-IgE cow milk protein allergy, which is the commonest driver of elimination diets in infancy and therefore of exactly the nutritional concerns this paper raises. Fifth, commercial extracts perform poorly for the heat-labile plant food proteins involved in pollen–food cross-reactivity, so that mechanism would be under-detected by the methods used. Sixth, the individual food involved could not be identified; the data derive from routine clinical collection with the recording inconsistencies that entails, some subgroups are small, and the population is a selected one. Study 2 has its own constraints. Convenience sampling, part of it in medical waiting rooms, enriches the sample for people with health problems, health-service contact and plausibly greater health-consciousness and engagement with health information and will therefore have inflated both the 17.6% self-report and the 6.6% test-confirmation figure; the sample is also better educated than the Romanian adult population, which will have raised the mean knowledge score, so the knowledge deficit reported here is more likely understated than overstated. Responses were self-reported and susceptible to social desirability bias, and the reported allergies could not be medically verified. The knowledge score is an ad hoc instrument with modest internal consistency (α = 0.58); measurement error of that magnitude attenuates the associations estimated from it, and two all-or-nothing items depress both the mean and the reliability—though a sensitivity analysis without them left every conclusion unchanged—so the score is best read as an ordinal indicator rather than a calibrated measure. No dietary variables were collected in either study, so the nutritional consequences discussed above are inferences from the literature and not observations of this population. Finally, although both cohorts derive from the same source population, they are differently selected—one clinical, one community—and measure different constructs, so their proportions must not be read as a direct comparison; the community cohort is also not a probability sample of that population, so neither cohort supports a population prevalence estimate. In addition, the questionnaire recorded residence only as urban or rural, so the overlap of the online arm of the survey with the clinic’s catchment could not be formally verified.

## 6. Conclusions

Among 4404 patients assessed in an allergy service over five consecutive years, 4.2% had documented food allergen sensitization, concentrated in early childhood, with age the only independent predictor—an association that the indication-driven nature of routine testing leaves confounded. A third of those with a recorded severity score had no active symptoms at assessment, a pattern consistent with clinically silent sensitization, though not proof of tolerance. Patients sensitized to food alone had substantially less respiratory and ocular disease than co-sensitized patients, though that contrast follows in large part from how the groups are defined; the differences in age and in cutaneous manifestations did not reach significance.

In a parallel survey of 500 adults, self-reported food allergy (17.6%) exceeded allergy reported as test-confirmed (6.6%) almost threefold, a gap of 11.0 percentage points, and knowledge was predominantly low to intermediate. Recognition that anaphylaxis can be fatal was not matched by knowledge of its treatment: 70.2% knew the condition can kill, 47.8% named adrenaline, and a third had never heard of an auto-injector. Only 15.4% always read allergen labels. Educational level and health-sector training were the strongest independent determinants of knowledge, while declared confidence tracked it only weakly.

The two cohorts are drawn from the same population but selected in different ways and measuring different constructs, so their proportions are not interchangeable; read together, they point in the same direction: the label of food allergy is applied more readily than it is verified, while preparedness for the minority of genuinely dangerous reactions is thin. Both ends of that problem are addressed by the same measures—better access to diagnostic confirmation, so that avoidance is applied where it is needed and lifted where it is not, and educational interventions stratified primarily by educational attainment, focused on anaphylaxis recognition, adrenaline use and practical label reading, delivered through the channels each age group actually uses. Three things would most advance this work: a sensitivity analysis restricted to patients actually tested for foods, a population sample recruited without reliance on medical settings, and, above all, direct measurement of the dietary restriction that unverified food allergy labels produce—the quantity this paper argues matters most and is the one it could not measure.

## Figures and Tables

**Table 1:** General characteristics of the allergy clinic cohort (*n* = 4404).

Characteristic	All Patients	Male	Female
Total patients, *n* (%)	4404 (100)	1976 (44.9)	2428 (55.1)
Age, mean ± SD, years	29.1 ± 19.0	25.5 ± 18.4	32.0 ± 19.1
Pediatric patients (<18 years), *n* (%)	1477 (33.5)	828 (41.9)	649 (26.7)
Sensitized patients, *n* (%)	2234 (50.7)	1156 (58.5)	1078 (44.4)
Non-sensitized patients, *n* (%)	2170 (49.3)	820 (41.5)	1350 (55.6)
Sensitized to food allergens, *n* (%)	185 (4.2)	102 (5.2)	83 (3.4)
Food-sensitized among sensitized patients, %	8.3	8.8	7.7

SD, standard deviation. Male and female percentages are computed within each sex stratum (first row: percentage of the whole cohort). Male patients were younger (*p* < 0.001) and more often pediatric (*p* < 0.001) than female patients, and more often carried any sensitization (58.5% versus 44.4%; χ^2^ = 86.1; *p* < 0.001); the sex contrast in food sensitization is reported in Section 3.1.2.

**Table 2:** Food allergen sensitization by age group, as a proportion of patients assessed.

Age Group (Years)	Assessed, *n*	Food-Sensitized, *n*	Positive in Group (%)	95% CI (%)	Share of Food-Sensitized (%)
0–5	590	77	13.05	10.6–16.0	41.6
6–12	612	29	4.74	3.3–6.7	15.7
13–17	275	7	2.55	1.2–5.2	3.8
18–30	793	24	3.03	2.0–4.5	13.0
31–45	1266	28	2.21	1.5–3.2	15.1
46–60	585	16	2.74	1.7–4.4	8.6
61+	283	4	1.41	0.6–3.6	2.2
Total	4404	185	4.20	3.6–4.8	100.0

95% CI computed by the Wilson score method. Heterogeneity across age groups: χ^2^ = 140.9; *df* = 6; *p* < 0.001; Cramér’s V = 0.18. Cochran–Armitage linear component on age-group midpoints (2.5, 9, 15, 24, 38, 53, 70 years): z = −8.14; trend χ^2^ = 66.2 on 1 *df*; departure from linearity χ^2^ = 74.7 on 5 *df*; both *p* < 0.001.

**Table 3:** Annual proportion of assessed patients with food allergen sensitization, overall and by sex, 2020–2024.

Year	Patients, *n*	Food-Sensitized, *n*	Positive (%)	95% CI (%)	Male/Female (%)
2020	782	19	2.43	1.6–3.8	2.61/2.26
2021	939	47	5.01	3.8–6.6	5.25/4.82
2022	976	44	4.51	3.4–6.0	5.37/3.78
2023	853	45	5.28	4.0–7.0	6.54/4.32
2024	854	30	3.51	2.5–5.0	6.07/1.47

Cochran–Armitage trend test across the five years: z = 0.96; *p* = 0.338. An exploratory logistic model found a nominal sex-by-year interaction (β = 0.25 per year; *p* = 0.023, unadjusted), consistent with the diverging sex-specific series; given the small annual event counts this is hypothesis-generating only.

**Table 4:** Co-sensitizations associated with food sensitization, by allergen class (*n* = 185).

Associated Allergen Class	*n*	% of Food-Sensitized Patients
Ragweed pollen	43	23.2
Grass pollen	36	19.5
House dust mites	31	16.8
Tree pollen	27	14.6
Animal dander	19	10.3
Moulds	19	10.3
Insect venom	14	7.6
Mugwort pollen	13	7.0
Drugs	10	5.4

Patients could have more than one co-sensitization; 91 patients (49.2%) had food-only sensitization.

**Table 5:** Clinical manifestations across sensitization groups, with odds ratios, test statistics, and unadjusted and Holm-adjusted *p* values. Panel C additionally includes sex and co-sensitization status.

Comparison and Manifestation	Group A, %	Group B, %	OR (95% CI)	χ^2^	*p*	Holm *p*
A. Food-sensitized (*n* = 185) vs. sensitized to non-food allergens only (*n* = 2049)						
Rhinitis	51.9	85.3	0.19 (0.14–0.25)	129.2	<0.001	<0.001
Conjunctivitis	24.9	42.4	0.45 (0.32–0.63)	20.9	<0.001	<0.001
Bronchial asthma	16.8	29.6	0.48 (0.32–0.71)	13.1	<0.001	<0.001
Urticaria/dermatitis	37.8	12.8	4.15 (3.00–5.74)	82.2	<0.001	<0.001
B. Food-only (*n* = 91) vs. co-sensitized (*n* = 94)						
Rhinitis	24.2	78.7	0.09 (0.04–0.17)	52.9	<0.001	<0.001
Conjunctivitis	9.9	39.4	0.17 (0.08–0.38)	19.9	<0.001	<0.001
Bronchial asthma	6.6	26.6	0.19 (0.08–0.50)	11.9	<0.001	0.001
Urticaria/dermatitis	45.1	30.9	1.84 (1.01–3.35)	3.4	0.066	0.066
Mean age, years	15.2	20.4	—	—	0.053	—
C. Pediatric < 18 y (*n* = 113) vs. adult ≥ 18 y (*n* = 72), among food-sensitized						
Male sex	67.3	36.1	3.63 (1.95–6.76)	16.0	<0.001	<0.001
Any additional sensitization	44.2	61.1	0.51 (0.28–0.92)	4.4	0.037	0.148
Rhinitis	46.9	59.7	0.60 (0.33–1.08)	2.4	0.121	0.242
Conjunctivitis	17.7	36.1	0.38 (0.19–0.75)	7.0	0.008	0.040
Bronchial asthma	12.4	23.6	0.46 (0.21–1.00)	3.2	0.073	0.220
Urticaria/dermatitis	37.2	38.9	0.93 (0.51–1.71)	0.01	0.936	0.936

Group A is the first-named group in each comparison, Group B the second. OR, odds ratio; CI, confidence interval; χ^2^, chi-squared statistic with Yates’ continuity correction (*df* = 1 throughout). Odds ratios are for Group A relative to Group B and, for outcomes as frequent as these, are further from unity than the corresponding risk ratios. Holm *p*, *p* value adjusted for multiplicity within each panel by the Holm–Bonferroni procedure; the mean-age row is an unadjusted Student’s *t* test. Panels A and B contrast overlapping subsets of the same 185 food-sensitized patients, and in panel A the comparator group is aeroallergen-sensitized by definition. Food-only: food sensitization with no other documented sensitization; co-sensitized: food sensitization plus at least one further sensitization.

**Table 6:** Factors independently associated with food sensitization (multivariable logistic regression with age modelled categorically, *n* = 4404).

Variable	Adjusted OR (95% CI)	*p* Value
Age Group		
0–5 years	4.61 (2.87–7.41)	<0.001
6–12 years	1.52 (0.87–2.65)	0.14
13–17 years	0.82 (0.35–1.93)	0.65
18–30 years	1.00 (reference)	—
31–45 years	0.72 (0.41–1.25)	0.24
46–60 years	0.90 (0.48–1.72)	0.76
≥61 years	0.47 (0.16–1.37)	0.17
Male sex (vs. female)	1.24 (0.92–1.69)	0.16
Year of presentation (per year)	1.05 (0.94–1.17)	0.40

OR, odds ratio; CI, confidence interval; AUC, area under the receiver operating characteristic curve. Categorical age fitted the data better than a continuous linear term (likelihood-ratio χ^2^ = 32.1; *df* = 5; *p* < 0.001); for comparability, the linear-age specification gave an adjusted OR of 0.69 per +10 years (95% CI 0.63–0.76). Crude OR for male sex: 1.54 (*p* = 0.005). Model calibration: Hosmer–Lemeshow χ^2^ = 6.01, *df* = 8, *p* = 0.65; discrimination: AUC 0.694 (bootstrap 95% CI 0.652–0.735); 185 events, 23 events per estimated parameter.

**Table 7:** Characteristics of the survey sample (*n* = 500).

Characteristic	Category	*n*	%
Sex	Female	297	59.4
	Male	195	39.0
	Prefer not to say	8	1.6
Residence	Urban	311	62.2
	Rural	189	37.8
Educational level	Lower secondary	60	12.0
	Upper secondary	180	36.0
	Post-secondary	70	14.0
	University	149	29.8
	Postgraduate	41	8.2
Medical training	Yes	40	8.0
	No	460	92.0

Mean age 46.7 years.

**Table 8:** Basis of the reported diagnosis among respondents self-reporting a food allergy (*n* = 88).

Basis of the Reported Allergy	*n*	% of Self-Reporters
Confirmed by allergy testing	33	37.5
Physician diagnosis on symptoms alone, without testing	37	42.0
Own assessment, unconfirmed	18	20.5

Self-reported food allergy: 88/500 (17.6%; 95% CI 14.5–21.2). Reported as test-confirmed, referred to the whole sample: 33/500 (6.6%; 95% CI 4.7–9.1); gap 11.0 percentage points (95% CI 8.5–14.0, Tango score method).

**Table 9:** Proportion of correct answers to the knowledge items (*n* = 500).

Item	Correct Answer	*n*	Correct (%)	95% CI (%)
Q10. Allergy is the same as intolerance	False	277	55.4	51.0–59.7
Q11. Allergy involves the immune system	True	322	64.4	60.1–68.5
Q12. Allergens subject to mandatory labelling	Complete set	43	8.6	6.4–11.4
Q13. A small amount can trigger a reaction	True	335	67.0	62.8–71.0
Q14. Cooking always destroys the allergen	False	282	56.4	52.0–60.7
Q15. Permanently cured by antihistamines	False	303	60.6	56.3–64.8
Q16. Manifestations of an allergic reaction	Complete set	52	10.4	8.0–13.4
Q17. Anaphylaxis can be fatal	True	351	70.2	66.0–74.0
Q18. First-line drug in anaphylaxis	Adrenaline	239	47.8	43.5–52.2
Q21. Management of a severe reaction	Emergency call/adrenaline	336	67.2	63.0–71.2

95% CI computed by the Wilson score method. Mean knowledge score 5.08/10 (SD 2.06; median 5; range 0–10); Cronbach’s α = 0.58 (95% CI 0.52–0.63, Feldt method). Knowledge level: low (score < 5) 41.0% (95% CI 36.8–45.4), intermediate (5–7) 45.4% (41.1–49.8), high (≥8) 13.6% (10.9–16.9). Q12 and Q16 were scored all-or-nothing (the exact correct set, no distractor) and measure exhaustive recall rather than working familiarity; an eight-item sensitivity score omitting them is reported in the text.

**Table 10:** Declared behaviour, attitudes, healthcare-seeking and information sources (*n* = 500).

Item	Response Option	*n*	%
Mild reaction (Q20)	Antihistamine	295	59.0
	Emergency department	87	17.4
	Wait for it to pass	42	8.4
	Call emergency services	36	7.2
	Do not know	40	8.0
Severe reaction (Q21)	Emergency call/adrenaline (correct)	336	67.2
	Antihistamine only, stay at home	80	16.0
	Wait to see if it worsens	62	12.4
	Do not know	22	4.4
Auto-injector (Q19)	Knows how to use it	191	38.2
	Has heard of it, cannot use it	142	28.4
	Has never heard of it	167	33.4
Label reading (Q22)	Always	77	15.4
	Sometimes	190	38.0
	Only if an allergy is present	133	26.6
	Never	100	20.0
Confidence in recognition (Q23)	Very confident	87	17.4
	Somewhat confident	201	40.2
	Slightly confident	158	31.6
	Not at all confident	54	10.8
Sufficient knowledge (Q26)	Yes	147	29.4
	Partly	238	47.6
	No	115	23.0
Interest in education (Q27)	Yes	357	71.4
	No/do not know	143	28.6
Physician consulted first (Q24)	Family physician	240	48.0
	Allergist	167	33.4
	Emergency physician	33	6.6
	Gastroenterologist	31	6.2
	Would not consult a physician	29	5.8
Information source (Q25)	Internet/social networks	164	32.8
	Physician	147	29.4
	Press/television	55	11.0
	Pharmacy staff	48	9.6
	Family/friends	44	8.8
	Does not seek information	42	8.4

**Table 11:** Independent predictors of the knowledge score, computed from the individual-level data (multiple linear regression, *n* = 492; R^2^ = 0.44).

Variable	β, Points (95% CI)	β in SD Units	*p* Value
Educational level (per step)	+0.89 (0.77 to 1.01)	+0.43	<0.001
Medical training (yes vs. no)	+2.34 (1.83 to 2.85)	+1.13	<0.001
Family history of allergy (yes vs. no)	+0.43 (0.11 to 0.76)	+0.21	0.009
Age (per +10 years)	−0.09 (−0.17 to −0.01)	−0.04	0.025
Urban residence (vs. rural)	+0.35 (0.05 to 0.64)	+0.17	0.023
Male sex (vs. female)	−0.14 (−0.42 to 0.14)	−0.07	0.32

β, regression coefficient in knowledge score points; the third column expresses the same coefficient in units of the score’s standard deviation (SD = 2.06)—the outcome is standardized, the predictors are not; CI, confidence interval. Eight respondents who did not state their sex were excluded, giving *n* = 492. R^2^ = 0.44. In an eight-item sensitivity score omitting the two all-or-nothing multiple-response items, educational level, medical training, family history and age remained significant with materially unchanged coefficients, while the urban–rural coefficient was not significant (*p* = 0.086) (R^2^ = 0.43).

## Data Availability

The original contributions presented in this study are included in the article/Supplementary Materials. Further inquiries can be directed to the corresponding authors.

## Supplementary Materials

The following supporting information can be downloaded at: https://www.mdpi.com/article/10.3390/nu18203324/s1, File S1: the full 27-item questionnaire used in Study 2, with its scoring key; Table S1: de-identified individual-level data for the allergy clinic cohort, 2020–2024 (*n* = 4404), with codebook; Table S2: de-identified individual-level responses of the population survey (*n* = 500), with codebook.
