# Supplementary material for: FoodAllergenSensitization in a Five-Year Allergy Clinic Cohort and Food Allergy Knowledge, Attitudes and Behaviours in Romanian Adults: Two Observational Studies
Source: Nutrients. 2026 Oct 10;18(20):3324. doi: 10.3390/nu18203324 (PMC13708197; doi:10.3390/nu18203324)
Supplement: Supplementary file 1 [file nutrients-18-03324-s001.zip › nutrients-4576026-supplementary.pdf]

File S1. The full 27-item questionnaire used in Study 2, with its scoring key

Romanian original with English translation

Supplementary material to: “Food Allergen Sensitization in a Five-Year Allergy Clinic Cohort and Food Allergy Knowledge, Attitudes and Behaviours in Romanian Adults: Two Observational Studies”.

Respondents completed the questionnaire in Romanian; the English translation is provided for the reader. The knowledge score (0–10) sums one point for each correct answer to the nine objectively scored items of sections C and D marked “Scored” below and to item 21; item 19 is not scored. Multiple-response items (12 and 16) are scored all-or-nothing against the predefined correct set. Item-level coded responses for all 500 participants are published as Table S2.

Section A — Demographic characteristics (items 1–5)

1. Vârsta (ani împliniți)

Age (completed years)

- (răspuns numeric) — *(numeric answer)*

2. Sexul

Sex

- masculin — *male*
- feminin — *female*
- prefer să nu răspund — *prefer not to say*

3. Mediul de proveniență

Area of residence

- urban — *urban*
- rural — *rural*

4. Ultimul nivel de educație absolvit

Highest completed educational level

- gimnazial — *lower secondary*
- liceal — *upper secondary*
- postliceal — *post-secondary*
- universitar — *university*
- postuniversitar — *postgraduate*

5. Aveți pregătire în domeniul medical/sanitar?

Do you have training in a medical or health-related field?

- da — *yes*
- nu — *no*

Section B — Allergy history and self-report (items 6–9)

6. Considerați că aveți o alergie alimentară?

Do you consider that you have a food allergy?

- da — *yes*
- nu — *no*
- nu sunt sigur(ă) — *not sure*

7. Alergia dumneavoastră a fost confirmată de un medic?

Has your allergy been confirmed by a physician?

- da, prin testare alergologică — *yes, by allergy testing*
- da, doar pe baza relatării simptomelor — *yes, but only on the basis of reported symptoms, without testing*
- nu, este o autoevaluare proprie — *no, it is my own self-assessment*
- nu este cazul — *not applicable*

8. La ce alimente reacționați? (răspuns multiplu)
Which foods do you react to? (multiple answers)

- lapte — *milk*
- ou — *egg*
- arahide — *peanuts*
- alune/nuci — *tree nuts*
- grâu/gluten — *wheat/gluten*
- pește — *fish*
- fructe de mare — *shellfish/seafood*
- soia — *soy*
- fructe/legume — *fruits/vegetables*
- altele — *other (free text)*
- niciunul — *none*

9. Există alergii alimentare diagnosticate în familia dumneavoastră?
Are there diagnosed food allergies in your family?

- da — *yes*
- nu — *no*
- nu știu — *I don't know*

Section C — Knowledge (items 10–18)

10. Alergia alimentară și intoleranța alimentară sunt același lucru.
Food allergy and food intolerance are the same thing.

- adevărat — *true*
- fals — *false*
- nu știu — *I don't know*

Scoring: Scored. Correct: FALSE (1 point).

11. Alergia alimentară presupune o reacție a sistemului imunitar.
Food allergy involves a reaction of the immune system.

- adevărat — *true*
- fals — *false*
- nu știu — *I don't know*

Scoring: Scored. Correct: TRUE (1 point).

12. Care dintre următorii sunt alergeni cu declarare obligatorie pe etichetă? (răspuns multiplu)
Which of the following are allergens subject to mandatory declaration on food labels? (multiple answers)

- lapte — *milk*
- ou — *egg*
- arahide — *peanuts*
- gluten/cereale — *gluten/cereals*
- fructe de mare — *shellfish/seafood*
- soia — *soy*
- zahăr — *sugar — distractor*
- apă — *water — distractor*

Scoring: Scored all-or-nothing. Correct: exactly the six allergens (milk, egg, peanuts, gluten/cereals, shellfish/seafood, soy) with neither distractor (1 point).

13. O cantitate mică de aliment poate declanșa reacția alergică.
A small quantity of the food can trigger the allergic reaction.

- adevărat — *true*
- fals — *false*
- nu știu — *I don't know*

Scoring: Scored. Correct: TRUE (1 point).

14. Gătirea distruge întotdeauna alergenul.
Cooking always destroys the allergen.

- adevărat — *true*
- fals — *false*
- nu știu — *I don't know*

Scoring: Scored. Correct: FALSE (1 point).

15. Alergia alimentară poate fi vindecată definitiv cu antihistaminice.
Food allergy can be permanently cured with antihistamines.

- adevărat — *true*
- fals — *false*
- nu știu — *I don't know*

Scoring: Scored. Correct: FALSE (1 point).

16. Care pot fi manifestări ale unei reacții alergice alimentare? (răspuns multiplu)
Which of the following can be manifestations of a food-allergic reaction? (multiple answers)

• urticarie/mâncărimi ale pielii	*hives/itching of the skin*
• umflarea buzelor sau a feței	*swelling of the lips or face*
• dificultate în respirație	*difficulty breathing*
• scăderea tensiunii arteriale/leșin	*drop in blood pressure/fainting*
• vărsături/diaree	*vomiting/diarrhoea*
• durere de cap fără alte simptome	*headache without any other symptom — distractor*

Scoring: Scored all-or-nothing per the instrument's key: exactly the five true manifestations (hives/itching of the skin, swelling of the lips or face, difficulty breathing, drop in blood pressure/fainting, vomiting/diarrhoea), with the headache distractor not selected (1 point).

17. Anafilaxia poate pune viața în pericol.
Anaphylaxis can be life-threatening.

• adevărat	*true*
• fals	*false*
• nu știu	*I don't know*

Scoring: Scored. Correct: TRUE (1 point).

18. Care este medicamentul de primă intenție în anafilaxie?
What is the first-line medication in anaphylaxis?

• adrenalina (epinefrina)	*adrenaline (epinephrine)*
• un antihistaminic oral	*an oral antihistamine*
• un corticosteroid în comprimate	*a corticosteroid in tablet form*
• nu știu	*I don't know*

Scoring: Scored. Correct: adrenaline (epinephrine) (1 point).

Section D — Behaviours and confidence (items 19–23)

19. Cunoașteți autoinjectorul de adrenalină?
Are you familiar with the adrenaline auto-injector?

• da, știu ce este și cum se folosește	*yes, I know what it is and how it is used*
• am auzit, dar nu știu cum se folosește	*I have heard of it, but do not know how it is used*
• nu am auzit de el	*I have never heard of it*

Scoring: Not scored (measures declared familiarity, not knowledge).

20. Cum ați proceda la o reacție ușoară (urticarie)?
What would you do in a mild reaction (hives)?

• aș lua/aș administra un antihistaminic	*I would take/administer an antihistamine*
• aș merge la camera de gardă	*I would go to the emergency room*
• aș suna la 112	*I would call 112*
• aș aștepta să treacă de la sine	*I would wait for it to pass on its own*
• nu știu	*I don't know*

21. Cum ați proceda la o reacție severă (dificultate în respirație sau umflarea feței)?
What would you do in a severe reaction (difficulty breathing or facial swelling)?

• aș suna imediat la 112 / aș folosi autoinjectorul de adrenalină dacă este disponibil	*I would immediately call 112 / use the adrenaline auto-injector if available*
• aș lua doar un antihistaminic și aș rămâne acasă	*I would only take an antihistamine and stay at home*
• aș aștepta să văd dacă se agravează	*I would wait to see whether it worsens*
• nu știu	*I don't know*

Scoring: Scored. Correct: call 112 immediately / use the auto-injector (1 point). This is the tenth scored item.

22. Citiți etichetele alimentelor pentru alergeni?
Do you read food labels for allergens?

• întotdeauna	*always*
• uneori	*sometimes*
• doar dacă am/are cineva o alergie	*only if I or someone close has an allergy*
• niciodată	*never*

23. Cât de încrezător(oare) sunteți că ați recunoaște o reacție alergică severă?
How confident are you that you would recognize a severe allergic reaction?

• foarte încrezător(oare)	*very confident*
• oarecum încrezător(oare)	*somewhat confident*
• puțin încrezător(oare)	*slightly confident*
• deloc încrezător(oare)	*not at all confident*

Section E — Healthcare pathway and attitudes (items 24–27)

24. La ce medic v-ați adresa pentru o suspiciune de alergie alimentară?
Which physician would you consult for a suspected food allergy?

- medic de familie — *family physician*
- medic alergolog — *allergist*
- medic gastroenterolog — *gastroenterologist*
- medic de urgență — *emergency physician*
- nu m-aș adresa unui medic — *I would not consult a physician*

25. Care este sursa dumneavoastră principală de informare despre alergiile alimentare?
What is your principal source of information on food allergies?

- medicul — *the physician*
- personalul farmaceutic — *pharmacy staff*
- internetul / rețelele sociale — *the internet / social networks*
- presa / televiziunea — *the press / television*
- familia / prietenii — *family / friends*
- nu mă informez pe acest subiect — *I do not seek information on this subject*

26. Considerați că aveți cunoștințe suficiente pentru a gestiona o alergie alimentară?
Do you consider that you have sufficient knowledge to manage a food allergy?

- da — *yes*
- parțial — *partially*
- nu — *no*

27. Ați fi interesat(ă) de programe de educație despre alergiile alimentare?
Would you be interested in educational programmes on food allergies?

- da — *yes*
- nu — *no*
- nu știu — *I don't know*

Table S1. De-identified individual-level data for the allergy clinic cohort, 2020–2024 (n = 4404), with codebook

De-identified individual-level records of all 4404 unique patients assessed in the allergy service between 1 January 2020 and 31 December 2024 (Study 1).
One row per patient. See the codebook on the next page.
These data underlie every Study 1 statistic reported in the article.

Codebook

```
ID   sequential record number (order of presentation; not a hospital identifier)
Yr   year of presentation (2020-2024)
Mo   calendar month of presentation (1-12)
Sex  M = male, F = female
Age  age in completed years (values above 90 top-coded to 90)
Tre/Grs/Rag/Mug  sensitization to tree / grass / ragweed / mugwort pollen (1 = positive, 0 = negative)
HDM/Dan/Mld      sensitization to house dust mites / animal dander / moulds
Food             sensitization to at least one food allergen
Drg/Ven          sensitization to drugs / insect venom
Rhi/Cnj/Ast/AsE/Urt  rhinitis / conjunctivitis / asthma / asthma exacerbation / urticaria-dermatitis (1 = present)
Sev  global symptom severity score at the index assessment (0-3; NA = not recorded)
Prt/Obs/Rho/Snz  rhinitis components present: pruritus / nasal obstruction / rhinorrhoea / sneezing
```

Sensitization was established by skin prick testing and/or allergen-specific serum IgE in routine practice. Records are fully de-identified: names, exact dates and any direct identifiers were removed before export; one row = one unique patient.

ID	Yr	Mo	Sex	Age	Tre	Grs	Rag	Mug	HDM	Dan	Mld	Food	Drg	Ven	Rhi	Cnj	Ast	AsE	Urt	Sev	Prt	Obs	Rho	Snz
1	2020	1	M	0	0	0	0	0	0	0	0	0	0	0	0	0	0	0	0	0	0	0	0	0
2	2020	1	M	0	0	0	0	0	0	0	0	0	0	0	0	0	0	0	0	2	1	1	1	1
3	2020	2	F	0	0	0	0	0	0	0	0	0	0	0	0	0	0	0	1	2	1	1	1	1
4	2020	3	M	0	0	0	0	0	0	0	0	0	0	0	0	0	0	0	1	0	0	0	0	0
5	2020	3	M	0	0	0	0	0	0	0	0	0	0	0	0	0	0	0	1	1	0	0	0	0
6	2020	7	M	0	0	0	0	0	0	0	0	0	0	0	1	0	0	0	0	2	1	1	1	1
7	2020	9	M	0	0	0	0	0	0	0	0	0	0	0	0	0	0	0	0	1	1	0	0	0
8	2020	10	M	0	0	0	0	0	0	0	0	0	0	0	0	0	0	0	0	0	0	0	0	0
9	2020	9	M	0	0	0	0	0	0	0	0	0	0	0	0	0	0	0	0	0	0	0	0	0
10	2020	10	M	0	0	0	0	0	0	0	0	0	0	0	0	0	0	0	1	2	0	1	0	0
11	2021	3	M	0	0	0	0	0	0	0	0	0	0	0	1	0	0	0	0	0	0	0	0	0
12	2021	4	M	0	0	0	0	0	0	0	0	0	0	0	0	0	0	1	0	0	0	0	0	0
13	2021	3	F	0	0	0	0	0	0	0	0	0	0	0	0	0	1	0	1	2	0	0	0	0
14	2022	5	F	0	0	0	0	0	0	0	0	0	0	0	0	0	0	0	0	1	0	1	0	0
15	2022	8	F	0	0	0	0	0	0	0	0	0	0	0	0	0	0	0	0	0	0	0	0	0
16	2022	9	F	0	0	0	0	0	0	0	0	0	0	0	0	0	0	0	1	0	0	0	0	0
17	2022	10	M	0	0	0	0	0	0	0	0	0	0	0	0	0	0	0	0	0	0	0	0	0
18	2022	12	M	0	0	0	0	0	0	0	0	0	0	0	0	0	0	0	0	2	0	1	1	1
19	2023	2	F	0	0	0	0	0	0	0	0	0	0	0	0	0	0	0	1	1	0	0	1	0
20	2023	2	F	0	0	0	0	0	0	0	0	0	0	0	0	0	0	0	1	0	0	0	0	0
21	2023	2	M	0	0	0	0	0	0	0	0	0	0	0	0	0	0	0	1	0	0	0	0	0
22	2023	2	M	0	0	0	0	0	0	0	0	0	0	0	1	0	0	0	0	0	0	0	0	0
23	2023	3	M	0	0	0	0	0	0	0	0	0	0	0	0	0	0	0	1	0	0	0	0	0
24	2023	3	F	0	0	0	0	0	0	0	0	0	0	0	0	0	0	0	1	0	0	0	0	0
25	2023	4	F	0	0	0	0	0	0	0	0	0	0	0	0	0	0	0	1	0	0	0	0	0
26	2023	5	M	0	0	0	0	0	0	0	0	0	0	0	1	0	0	0	0	2	1	1	1	1
27	2023	5	M	0	0	0	0	0	0	0	0	0	0	0	0	0	0	0	1	2	0	0	0	0
28	2023	6	M	0	0	0	0	0	0	0	0	0	0	0	0	0	0	0	1	0	0	0	0	0
29	2023	7	M	0	0	0	0	0	0	0	0	0	0	0	1	0	0	0	0	0	0	0	0	0
30	2023	7	F	0	0	0	0	0	0	0	0	0	0	0	0	0	0	0	1	0	0	0	0	0
31	2023	7	M	0	0	0	0	0	0	0	0	0	0	0	0	0	0	0	1	0	0	0	0	0
32	2023	8	F	0	0	0	0	0	0	0	0	0	0	0	0	0	0	0	1	2	0	1	1	0
33	2024	2	M	0	0	0	0	0	0	0	0	0	0	0	0	0	0	0	1	2	0	1	1	1
34	2024	3	F	0	0	0	0	0	0	0	0	0	0	0	0	0	0	0	1	0	0	0	0	0
35	2024	4	F	0	0	0	0	0	0	0	0	0	0	0	0	0	0	0	0	2	0	1	0	1
36	2024	4	M	0	0	0	0	0	0	0	0	0	0	0	0	0	0	0	1	2	0	1	1	0
37	2024	6	M	0	0	0	0	0	0	0	0	0	0	0	0	0	0	0	1	2	1	0	1	1
38	2024	6	F	0	0	0	0	0	0	0	0	0	0	0	0	0	0	0	1	2	0	1	1	1
39	2024	7	F	0	0	0	0	0	0	0	0	0	0	0	0	0	0	0	1	0	0	0	0	0
40	2024	7	F	0	0	0	0	0	0	0	0	0	0	0	0	0	0	0	1	0	0	0	1	1
41	2024	7	M	0	0	0	0	0	0	0	0	0	0	0	0	0	0	0	1	2	0	1	0	0
42	2024	7	F	0	0	0	0	0	0	0	0	0	0	0	0	0	0	0	1	2	0	0	0	0
43	2023	8	M	0	0	0	0	0	0	0	0	0	0	0	0	0	0	0	1	0	0	0	0	0
44	2024	10	M	0	0	0	0	0	0	0	0	0	0	0	1	0	0	0	0	0	0	0	0	0
45	2024	10	M	0	0	0	0	0	0	0	0	0	0	0	0	0	0	0	1	0	0	0	0	0
46	2024	11	M	0	0	0	0	0	0	0	0	0	0	0	0	0	0	0	1	0	0	0	0	0
47	2024	11	M	0	0	0	0	0	0	0	0	0	0	0	0	0	0	0	0	0	0	0	0	0
48	2020	8	M	0	0	0	0	0	0	0	0	1	0	0	0	0	0	0	0	0	0	0	0	0
49	2020	10	F	0	0	0	0	0	0	0	0	1	0	0	0	0	0	0	0	1	0	0	0	0
50	2021	11	F	0	0	0	0	0	0	0	0	0	0	1	0	0	0	0	0	1	1	1	1	1
51	2021	1	F	0	0	0	0	0	0	0	0	1	0	0	0	0	0	0	0	2	0	1	1	1
52	2023	6	M	0	0	0	0	0	0	0	0	1	0	0	0	0	0	0	1	0	1	0	0	0
53	2024	1	M	0	0	0	0	0	0	0	0	1	0	0	0	0	0	0	1	2	0	0	1	0
54	2024	4	M	0	0	0	0	0	0	0	0	1	0	0	0	0	0	0	1	0	0	0	0	0
55	2020	2	M	1	0	0	0	0	0	0	0	0	0	0	1	0	0	0	1	2	0	1	0	0
56	2020	2	M	1	0	0	0	0	0	0	0	0	0	0	0	0	0	0	0	2	1	1	1	1
57	2020	2	M	1	0	0	0	0	0	0	0	0	0	0	0	0	0	0	0	2	0	1	0	0
58	2020	5	M	1	0	0	0	0	0	0	0	0	0	0	0	0	0	0	0	1	0	1	0	0
59	2020	7	M	1	0	0	0	0	0	0	0	0	0	0	0	0	0	0	0	1	0	0	0	0
60	2020	8	M	1	0	0	0	0	0	0	0	0	0	0	0	0	0	0	0	0	0	0	0	0
61	2020	10	M	1	0	0	0	0	0	0	0	0	0	0	0	0	0	0	0	1	1	0	0	0
62	2020	11	M	1	0	0	0	0	0	0	0	0	0	0	0	0	0	0	0	2	0	0	1	1
63	2020	10	F	1	0	0	0	0	0	0	0	0	0	0	0	0	0	0	0	0	0	0	0	0

ID	Yr	Mo	Sex	Age	Tre	Grs	Rag	Mug	HDM	Dan	Mld	Food	Drg	Ven	Rhi	Cnj	Ast	AsE	Urt	Sev	Prt	Obs	Rho	Snz
64	2020	12	F	1	0	0	0	0	0	0	0	0	0	0	0	0	0	0	0	2	1	1	0	1
65	2021	4	M	1	0	0	0	0	0	0	0	0	0	0	0	0	0	0	1	1	0	0	0	0
66	2021	5	M	1	0	0	0	0	0	0	0	0	0	0	1	0	0	0	0	0	0	0	0	0
67	2021	6	M	1	0	0	0	0	0	0	0	0	0	0	0	0	0	0	1	0	1	1	0	0
68	2021	9	F	1	0	0	0	0	0	0	0	0	0	0	0	0	0	0	0	2	0	0	1	1
69	2021	10	F	1	0	0	0	0	0	0	0	0	0	0	0	0	0	0	1	1	1	1	0	0
70	2021	12	M	1	0	0	0	0	0	0	0	0	0	0	0	0	0	1	0	2	0	0	0	0
71	2021	2	F	1	0	0	0	0	0	0	0	0	0	0	0	0	1	0	1	0	0	0	0	0
72	2021	10	M	1	0	0	0	0	0	0	0	0	0	0	0	0	0	1	0	1	0	1	0	0
73	2021	11	M	1	0	0	0	0	0	0	0	0	0	0	0	0	0	0	0	2	0	0	0	0
74	2021	12	M	1	0	0	0	0	0	0	0	0	0	0	0	0	0	1	0	0	0	0	0	0
75	2021	2	M	1	0	0	0	0	0	0	0	0	0	0	1	0	1	0	1	2	0	0	1	1
76	2021	5	M	1	0	0	0	0	0	0	0	0	0	0	0	0	0	1	0	0	0	0	0	0
77	2022	1	M	1	0	0	0	0	0	0	0	0	0	0	1	0	0	0	1	2	1	1	1	1
78	2022	1	F	1	0	0	0	0	0	0	0	0	0	0	0	0	0	0	0	1	1	0	1	1
79	2022	2	M	1	0	0	0	0	0	0	0	0	0	0	0	0	0	0	1	0	0	0	0	0
80	2022	4	M	1	0	0	0	0	0	0	0	0	0	0	0	0	0	0	0	1	1	0	0	1
81	2022	5	M	1	0	0	0	0	0	0	0	0	0	0	0	0	0	0	0	2	1	1	1	1
82	2022	5	F	1	0	0	0	0	0	0	0	0	0	0	1	0	0	0	0	2	0	1	0	0
83	2022	5	M	1	0	0	0	0	0	0	0	0	0	0	0	0	0	0	0	1	1	0	0	0
84	2022	5	F	1	0	0	0	0	0	0	0	0	0	0	0	0	0	0	1	1	1	1	1	0
85	2022	6	F	1	0	0	0	0	0	0	0	0	0	0	0	0	0	0	0	2	0	1	0	0
86	2022	6	F	1	0	0	0	0	0	0	0	0	0	0	0	0	0	0	0	2	0	0	0	0
87	2022	8	M	1	0	0	0	0	0	0	0	0	0	0	0	0	0	0	1	0	0	0	0	0
88	2022	9	M	1	0	0	0	0	0	0	0	0	0	0	0	0	0	0	0	0	0	0	0	0
89	2022	9	F	1	0	0	0	0	0	0	0	0	0	0	1	0	0	0	0	2	1	0	0	0
90	2022	9	F	1	0	0	0	0	0	0	0	0	0	0	0	0	0	0	0	2	0	1	1	1
91	2022	10	F	1	0	0	0	0	0	0	0	0	0	0	1	0	0	0	0	0	0	0	0	0
92	2022	11	F	1	0	0	0	0	0	0	0	0	0	0	0	0	0	0	0	2	0	1	1	1
93	2022	11	F	1	0	0	0	0	0	0	0	0	0	0	0	0	0	0	0	1	0	1	0	0
94	2022	11	M	1	0	0	0	0	0	0	0	0	0	0	0	0	0	0	1	1	0	1	1	1
95	2022	12	M	1	0	0	0	0	0	0	0	0	0	0	0	0	0	0	1	2	0	1	1	0
96	2022	12	M	1	0	0	0	0	0	0	0	0	0	0	0	0	0	0	1	0	1	1	1	1
97	2022	12	M	1	0	0	0	0	0	0	0	0	0	0	0	0	0	0	0	2	0	1	1	1
98	2022	12	M	1	0	0	0	0	0	0	0	0	0	0	0	0	0	0	0	1	0	1	1	0
99	2023	2	M	1	0	0	0	0	0	0	0	0	0	0	0	0	0	0	1	0	0	0	0	0
100	2023	3	M	1	0	0	0	0	0	0	0	0	0	0	1	0	0	0	1	1	0	0	1	0
101	2023	4	F	1	0	0	0	0	0	0	0	0	0	0	0	0	0	0	1	1	1	1	1	1
102	2023	5	F	1	0	0	0	0	0	0	0	0	0	0	1	0	0	0	0	1	1	1	0	1
103	2023	5	F	1	0	0	0	0	0	0	0	0	0	0	0	0	0	0	1	2	1	1	1	1
104	2023	5	F	1	0	0	0	0	0	0	0	0	0	0	1	0	0	0	0	0	0	0	0	0
105	2023	5	F	1	0	0	0	0	0	0	0	0	0	0	0	0	0	0	1	1	1	0	1	1
106	2023	6	M	1	0	0	0	0	0	0	0	0	0	0	1	0	0	0	0	0	0	0	0	0
107	2023	8	M	1	0	0	0	0	0	0	0	0	0	0	1	0	0	0	0	1	0	0	0	0
108	2023	8	M	1	0	0	0	0	0	0	0	0	0	0	0	0	0	0	1	1	1	1	1	1
109	2023	11	M	1	0	0	0	0	0	0	0	0	0	0	1	0	0	0	0	0	0	0	0	0
110	2023	12	M	1	0	0	0	0	0	0	0	0	0	0	1	0	0	0	1	0	0	0	0	0
111	2024	1	M	1	0	0	0	0	0	0	0	0	0	0	0	0	0	1	0	0	0	0	1	1
112	2024	2	F	1	0	0	0	0	0	0	0	0	0	0	0	0	0	0	0	0	1	0	1	1
113	2024	2	F	1	0	0	0	0	0	0	0	0	0	0	0	0	0	0	1	1	0	1	0	0
114	2024	3	M	1	0	0	0	0	0	0	0	0	0	0	0	0	0	0	0	0	0	0	0	0
115	2024	5	M	1	0	0	0	0	0	0	0	0	0	0	0	0	0	0	0	1	1	1	1	1
116	2024	5	M	1	0	0	0	0	0	0	0	0	0	0	0	0	0	0	1	2	0	0	0	0
117	2024	5	M	1	0	0	0	0	0	0	0	0	0	0	0	0	0	0	1	1	0	0	0	0
118	2024	8	M	1	0	0	0	0	0	0	0	0	0	0	0	0	0	0	1	0	0	0	0	0
119	2024	9	M	1	0	0	0	0	0	0	0	0	0	0	0	0	0	0	0	0	0	0	0	0
120	2024	12	M	1	0	0	0	0	0	0	0	0	0	0	0	0	0	0	1	1	1	0	0	0
121	2024	12	F	1	0	0	0	0	0	0	0	0	0	0	0	0	0	0	0	1	1	0	0	0
122	2020	10	F	1	0	0	0	0	0	0	0	1	0	0	0	0	0	0	0	0	0	0	0	0
123	2021	9	F	1	0	0	0	0	0	0	0	1	0	0	0	0	0	1	0	0	0	0	0	0
124	2021	9	M	1	0	0	0	0	0	0	0	1	0	0	0	0	0	0	1	0	0	0	0	0
125	2021	10	F	1	0	0	0	0	0	0	0	1	0	0	0	0	0	1	0	0	0	0	0	0
126	2021	5	M	1	0	0	0	0	0	0	0	0	0	1	0	0	0	1	0	0	0	0	0	0

ID	Yr	Mo	Sex	Age	Tre	Grs	Rag	Mug	HDM	Dan	Mld	Food	Drg	Ven	Rhi	Cnj	Ast	AsE	Urt	Sev	Prt	Obs	Rho	Snz
127	2021	5	F	1	0	0	0	0	0	0	0	1	0	0	0	0	0	1	0	0	0	0	0	0
128	2021	12	F	1	0	0	0	0	0	0	0	1	0	0	0	0	0	1	0	1	1	0	0	0
129	2021	12	M	1	0	0	0	0	0	0	0	0	0	1	0	0	0	0	0	1	1	0	1	1
130	2021	8	F	1	0	0	0	0	0	0	0	0	0	1	0	0	0	0	0	0	0	0	0	0
131	2022	2	M	1	0	0	0	0	0	0	0	1	0	0	1	0	0	0	1	0	0	0	0	0
132	2022	11	F	1	0	0	0	0	0	0	0	1	0	0	0	0	0	0	0	0	0	0	0	0
133	2022	11	M	1	0	0	0	0	0	0	0	1	0	0	0	0	0	0	0	2	0	0	0	0
134	2023	1	M	1	0	0	0	0	0	0	0	1	0	0	0	0	0	0	1	2	0	1	0	1
135	2023	2	M	1	0	0	0	0	0	0	0	1	0	0	0	0	0	0	1	2	0	1	0	1
136	2023	3	F	1	0	0	0	0	0	0	0	1	0	0	0	0	0	0	1	2	1	1	0	0
137	2023	4	M	1	0	0	0	0	0	0	0	1	0	0	0	0	0	0	0	1	0	1	0	0
138	2023	4	M	1	0	0	0	0	0	0	0	1	0	0	1	0	0	0	1	0	0	0	0	0
139	2023	10	F	1	0	0	0	0	0	0	0	1	0	0	0	0	0	0	1	0	0	0	0	0
140	2023	11	F	1	0	0	0	0	0	0	0	1	0	0	1	0	0	0	1	2	1	0	1	1
141	2024	2	M	1	0	0	0	0	0	0	0	1	0	0	0	0	0	0	0	2	1	0	0	1
142	2024	2	M	1	0	0	0	0	0	0	0	0	1	0	0	0	0	0	0	0	0	0	0	0
143	2024	11	M	1	0	0	0	0	0	0	0	1	0	0	0	0	0	0	0	2	0	1	1	0
144	2020	10	M	1	0	0	0	0	1	0	0	0	0	0	1	0	0	0	0	0	0	0	0	0
145	2023	9	M	1	0	0	1	0	0	0	0	0	0	0	1	0	0	0	0	0	0	0	0	0
146	2023	4	M	1	0	1	0	0	0	0	0	0	0	0	1	0	0	0	0	1	0	1	0	0
147	2023	6	M	1	0	0	0	0	0	0	1	1	0	0	0	0	0	0	1	0	0	0	0	0
148	2020	10	F	1	0	1	1	0	0	0	0	0	0	0	1	0	0	0	0	0	0	0	0	0
149	2021	1	M	1	0	0	0	0	1	1	0	1	0	0	0	0	0	0	1	0	0	0	0	0
150	2020	1	F	2	0	0	0	0	0	0	0	0	0	0	0	0	0	0	0	2	0	1	0	0
151	2020	1	M	2	0	0	0	0	0	0	0	0	0	0	0	0	0	0	1	1	0	1	0	0
152	2020	1	F	2	0	0	0	0	0	0	0	0	0	0	0	0	0	0	0	1	0	1	0	1
153	2020	1	M	2	0	0	0	0	0	0	0	0	0	0	1	0	0	0	0	0	0	0	0	0
154	2020	8	M	2	0	0	0	0	0	0	0	0	0	0	0	0	0	0	0	0	0	0	0	0
155	2020	8	M	2	0	0	0	0	0	0	0	0	0	0	1	1	0	0	0	2	1	1	1	1
156	2020	9	F	2	0	0	0	0	0	0	0	0	0	0	0	0	0	0	1	2	0	1	1	1
157	2020	9	M	2	0	0	0	0	0	0	0	0	0	0	1	0	0	0	0	2	0	1	0	0
158	2020	9	F	2	0	0	0	0	0	0	0	0	0	0	0	0	0	0	0	2	1	1	1	1
159	2021	6	F	2	0	0	0	0	0	0	0	0	0	0	1	0	0	0	0	1	0	1	1	1
160	2021	7	F	2	0	0	0	0	0	0	0	0	0	0	0	0	0	0	0	0	0	0	0	0
161	2021	9	F	2	0	0	0	0	0	0	0	0	0	0	1	1	0	0	0	2	0	1	1	1
162	2021	9	M	2	0	0	0	0	0	0	0	0	0	0	0	0	0	1	0	0	0	0	0	0
163	2021	9	M	2	0	0	0	0	0	0	0	0	0	0	0	0	0	0	1	1	1	0	1	1
164	2021	10	M	2	0	0	0	0	0	0	0	0	0	0	0	0	0	0	1	2	1	1	1	1
165	2021	12	F	2	0	0	0	0	0	0	0	0	0	0	0	0	0	0	1	0	0	0	0	0
166	2021	11	F	2	0	0	0	0	0	0	0	0	0	0	0	0	0	0	1	0	0	0	0	0
167	2021	12	M	2	0	0	0	0	0	0	0	0	0	0	0	0	0	0	1	1	1	1	0	1
168	2021	6	F	2	0	0	0	0	0	0	0	0	0	0	0	0	0	1	0	1	0	0	0	0
169	2021	2	F	2	0	0	0	0	0	0	0	0	0	0	0	0	0	0	0	0	0	0	0	0
170	2021	1	M	2	0	0	0	0	0	0	0	0	0	0	0	0	0	0	1	1	1	1	0	0
171	2021	12	F	2	0	0	0	0	0	0	0	0	0	0	0	0	0	1	0	2	0	1	0	0
172	2020	12	M	2	0	0	0	0	0	0	0	0	0	0	1	0	0	0	1	2	0	0	1	1
173	2021	9	F	2	0	0	0	0	0	0	0	0	0	0	0	0	0	0	0	2	1	1	1	1
174	2022	1	M	2	0	0	0	0	0	0	0	0	0	0	0	0	0	0	0	2	1	1	1	1
175	2022	1	F	2	0	0	0	0	0	0	0	0	0	0	0	0	0	0	1	2	1	1	1	1
176	2022	2	M	2	0	0	0	0	0	0	0	0	0	0	1	0	0	0	0	2	0	0	0	0
177	2022	3	F	2	0	0	0	0	0	0	0	0	0	0	0	0	0	0	0	1	0	1	0	0
178	2022	3	F	2	0	0	0	0	0	0	0	0	0	0	1	0	0	0	0	0	0	0	0	0
179	2022	3	M	2	0	0	0	0	0	0	0	0	0	0	1	0	0	0	0	2	1	1	0	1
180	2022	4	F	2	0	0	0	0	0	0	0	0	0	0	0	0	0	0	0	2	1	0	1	1
181	2022	4	M	2	0	0	0	0	0	0	0	0	0	0	1	0	0	0	0	1	1	0	0	0
182	2022	8	M	2	0	0	0	0	0	0	0	0	0	0	0	0	0	0	0	0	0	0	0	0
183	2022	9	M	2	0	0	0	0	0	0	0	0	0	0	1	0	0	0	1	2	0	1	1	1
184	2022	10	M	2	0	0	0	0	0	0	0	0	0	0	1	0	0	0	0	1	0	1	0	0
185	2022	10	M	2	0	0	0	0	0	0	0	0	0	0	1	0	0	0	0	1	1	0	1	1
186	2022	11	F	2	0	0	0	0	0	0	0	0	0	0	0	0	0	0	0	2	1	1	1	1
187	2022	11	F	2	0	0	0	0	0	0	0	0	0	0	0	0	0	0	0	2	0	1	1	0
188	2022	12	F	2	0	0	0	0	0	0	0	0	0	0	0	0	0	0	0	2	1	1	1	1
189	2022	12	M	2	0	0	0	0	0	0	0	0	0	0	0	0	0	0	1	1	1	0	1	1

ID	Yr	Mo	Sex	Age	Tre	Grs	Rag	Mug	HDM	Dan	Mld	Food	Drg	Ven	Rhi	Cnj	Ast	AsE	Urt	Sev	Prt	Obs	Rho	Snz
190	2023	1	F	2	0	0	0	0	0	0	0	0	0	0	0	0	0	0	0	2	1	0	1	1
191	2023	1	F	2	0	0	0	0	0	0	0	0	0	0	0	0	0	0	1	2	0	1	1	1
192	2023	2	F	2	0	0	0	0	0	0	0	0	0	0	0	0	0	0	1	2	1	0	1	1
193	2023	2	M	2	0	0	0	0	0	0	0	0	0	0	1	0	0	0	0	0	0	0	0	0
194	2023	3	M	2	0	0	0	0	0	0	0	0	0	0	0	0	0	0	0	2	0	1	0	1
195	2023	4	M	2	0	0	0	0	0	0	0	0	0	0	1	0	0	0	0	2	1	1	0	0
196	2023	4	M	2	0	0	0	0	0	0	0	0	0	0	1	1	0	0	0	2	0	1	1	0
197	2023	4	F	2	0	0	0	0	0	0	0	0	0	0	1	1	0	0	0	2	1	1	1	1
198	2023	4	M	2	0	0	0	0	0	0	0	0	0	0	1	0	0	0	0	2	0	0	0	0
199	2023	4	F	2	0	0	0	0	0	0	0	0	0	0	0	0	0	0	0	0	0	0	0	0
200	2023	5	M	2	0	0	0	0	0	0	0	0	0	0	1	0	0	0	0	0	0	0	0	0
201	2023	5	M	2	0	0	0	0	0	0	0	0	0	0	1	0	0	0	1	2	0	0	0	0
202	2023	6	M	2	0	0	0	0	0	0	0	0	0	0	0	0	0	0	0	1	0	0	0	0
203	2023	9	M	2	0	0	0	0	0	0	0	0	0	0	1	0	0	0	0	1	0	0	0	0
204	2023	11	F	2	0	0	0	0	0	0	0	0	0	0	0	0	0	0	1	2	1	1	0	1
205	2024	1	F	2	0	0	0	0	0	0	0	0	0	0	0	0	0	0	1	1	0	1	1	0
206	2024	1	F	2	0	0	0	0	0	0	0	0	0	0	0	0	0	0	1	2	0	1	0	0
207	2024	2	M	2	0	0	0	0	0	0	0	0	0	0	0	0	0	0	1	0	0	0	0	0
208	2024	3	F	2	0	0	0	0	0	0	0	0	0	0	0	0	0	0	1	1	1	1	0	1
209	2024	3	F	2	0	0	0	0	0	0	0	0	0	0	0	0	0	0	1	2	0	0	0	0
210	2024	3	M	2	0	0	0	0	0	0	0	0	0	0	0	0	0	0	0	0	0	0	0	0
211	2024	4	M	2	0	0	0	0	0	0	0	0	0	0	0	1	0	0	0	1	0	1	0	0
212	2024	4	M	2	0	0	0	0	0	0	0	0	0	0	0	0	0	0	0	2	0	0	1	0
213	2024	4	M	2	0	0	0	0	0	0	0	0	0	0	0	0	0	0	1	1	1	1	1	1
214	2024	5	M	2	0	0	0	0	0	0	0	0	0	0	1	0	0	0	1	2	1	1	1	1
215	2024	6	M	2	0	0	0	0	0	0	0	0	0	0	0	0	0	0	0	0	0	0	0	0
216	2024	6	M	2	0	0	0	0	0	0	0	0	0	0	0	0	0	0	0	0	0	0	0	0
217	2024	7	M	2	0	0	0	0	0	0	0	0	0	0	0	0	0	0	0	2	1	1	1	1
218	2024	8	F	2	0	0	0	0	0	0	0	0	0	0	1	1	0	0	0	1	0	1	0	1
219	2024	9	F	2	0	0	0	0	0	0	0	0	0	0	0	0	0	0	0	2	1	1	1	1
220	2024	10	M	2	0	0	0	0	0	0	0	0	0	0	0	0	0	0	1	1	0	0	0	0
221	2024	10	M	2	0	0	0	0	0	0	0	0	0	0	0	0	0	0	1	0	0	0	0	0
222	2021	4	M	2	0	0	0	0	0	0	0	1	0	0	1	0	0	0	0	0	0	0	0	0
223	2021	9	F	2	0	0	0	0	0	0	0	0	0	1	0	0	0	0	0	2	0	0	0	1
224	2021	11	F	2	0	0	0	0	0	0	0	1	0	0	0	0	0	0	0	0	0	0	0	0
225	2021	3	F	2	0	0	0	0	0	0	0	1	0	0	0	0	1	0	1	1	0	0	1	1
226	2021	10	M	2	0	0	0	0	0	0	0	0	0	1	0	0	0	0	0	2	0	1	1	1
227	2021	8	F	2	0	0	0	0	0	0	0	1	0	0	0	0	0	1	1	2	0	1	1	1
228	2023	3	M	2	0	0	0	0	0	0	0	1	0	0	0	0	0	0	1	0	0	0	0	0
229	2023	10	F	2	0	0	0	0	0	0	0	1	0	0	0	0	0	0	0	2	1	1	1	1
230	2024	5	M	2	0	0	0	0	0	0	0	1	0	0	1	0	0	0	0	0	0	0	0	0
231	2024	7	M	2	0	0	0	0	0	0	0	1	0	0	0	0	0	0	1	2	0	1	0	1
232	2021	12	F	2	0	0	0	0	0	0	0	1	0	1	0	0	0	1	0	0	0	0	0	0
233	2020	1	M	2	0	0	0	0	1	0	0	0	0	0	1	0	0	0	1	1	0	1	1	0
234	2021	9	F	2	0	0	0	0	1	0	0	0	0	0	0	0	0	0	1	2	1	1	1	1
235	2020	10	F	2	0	0	0	0	1	0	0	1	0	0	1	0	1	0	1	0	0	0	0	0
236	2021	9	M	2	0	0	0	0	1	0	0	0	0	1	0	0	0	0	0	2	0	0	0	0
237	2021	11	F	2	0	0	1	0	0	0	0	0	0	1	0	0	0	0	0	2	0	0	0	0
238	2023	8	M	2	0	1	0	0	0	0	0	1	0	0	1	0	0	0	0	1	1	0	0	0
239	2024	9	M	2	0	0	0	0	1	0	0	1	0	0	1	0	0	0	0	0	0	0	0	0
240	2024	10	M	2	0	0	0	0	1	0	0	1	0	0	0	0	0	0	1	1	1	0	0	1
241	2020	9	M	2	0	0	1	0	0	1	0	0	0	0	1	1	0	0	0	1	0	1	0	0
242	2022	11	M	2	0	1	1	0	0	0	0	0	0	0	1	0	0	0	0	2	1	0	0	0
243	2021	10	M	2	1	1	1	0	0	0	0	0	0	1	0	0	0	0	0	1	0	1	0	0
244	2023	11	M	2	0	1	0	0	1	1	0	1	0	0	1	0	0	0	0	0	0	0	0	0
245	2021	3	F	2	0	0	0	0	0	0	0	0	0	0	0	0	0	0	0	2	0	1	0	0
246	2020	1	M	3	0	0	0	0	0	0	0	0	0	0	0	0	0	0	0	1	0	0	0	0
247	2020	3	M	3	0	0	0	0	0	0	0	0	0	0	0	0	0	0	0	2	0	1	1	1
248	2020	3	F	3	0	0	0	0	0	0	0	0	0	0	0	0	0	0	0	1	0	0	0	0
249	2020	7	M	3	0	0	0	0	0	0	0	0	0	0	0	0	0	0	0	2	1	1	1	1
250	2020	7	M	3	0	0	0	0	0	0	0	0	0	0	0	0	0	0	0	2	0	0	1	1
251	2020	9	M	3	0	0	0	0	0	0	0	0	0	0	1	1	0	0	0	0	0	0	0	0
252	2020	9	F	3	0	0	0	0	0	0	0	0	0	0	0	0	0	0	0	2	0	1	1	1

ID	Yr	Mo	Sex	Age	Tre	Grs	Rag	Mug	HDM	Dan	Mld	Food	Drg	Ven	Rhi	Cnj	Ast	AsE	Urt	Sev	Prt	Obs	Rho	Snz
253	2020	11	M	3	0	0	0	0	0	0	0	0	0	0	0	0	0	0	1	0	0	0	0	0
254	2020	11	M	3	0	0	0	0	0	0	0	0	0	0	0	0	0	0	0	2	1	1	1	1
255	2021	4	M	3	0	0	0	0	0	0	0	0	0	0	1	0	0	0	0	2	1	1	1	1
256	2021	5	M	3	0	0	0	0	0	0	0	0	0	0	1	0	0	0	1	1	1	1	0	0
257	2021	7	M	3	0	0	0	0	0	0	0	0	0	0	1	0	0	0	0	2	1	1	1	0
258	2021	8	M	3	0	0	0	0	0	0	0	0	0	0	0	0	0	0	0	0	0	0	0	0
259	2021	9	M	3	0	0	0	0	0	0	0	0	0	0	0	0	0	0	1	1	1	1	0	0
260	2021	9	F	3	0	0	0	0	0	0	0	0	0	0	0	0	0	0	0	2	0	1	1	1
261	2021	12	F	3	0	0	0	0	0	0	0	0	0	0	1	0	0	0	1	0	0	0	0	0
262	2021	12	M	3	0	0	0	0	0	0	0	0	0	0	0	0	0	0	1	0	0	0	0	0
263	2021	6	F	3	0	0	0	0	0	0	0	0	0	0	0	0	0	1	0	1	0	0	0	0
264	2021	5	F	3	0	0	0	0	0	0	0	0	0	0	0	0	0	1	0	0	0	0	0	0
265	2022	1	M	3	0	0	0	0	0	0	0	0	0	0	0	0	0	0	0	1	1	1	1	1
266	2022	1	F	3	0	0	0	0	0	0	0	0	0	0	1	0	0	0	0	0	0	0	0	0
267	2022	1	F	3	0	0	0	0	0	0	0	0	0	0	1	0	0	0	0	0	0	0	0	0
268	2022	2	F	3	0	0	0	0	0	0	0	0	0	0	0	0	0	0	0	0	0	0	0	0
269	2022	2	F	3	0	0	0	0	0	0	0	0	0	0	1	0	0	0	0	2	1	1	1	1
270	2022	2	F	3	0	0	0	0	0	0	0	0	0	0	0	0	0	0	0	1	0	0	0	1
271	2022	2	M	3	0	0	0	0	0	0	0	0	0	0	1	0	0	0	0	2	0	1	1	0
272	2022	2	F	3	0	0	0	0	0	0	0	0	0	0	0	0	0	0	0	1	0	0	0	0
273	2022	3	F	3	0	0	0	0	0	0	0	0	0	0	0	0	0	0	1	2	0	1	1	0
274	2022	3	F	3	0	0	0	0	0	0	0	0	0	0	0	0	0	0	1	2	0	1	0	0
275	2022	4	F	3	0	0	0	0	0	0	0	0	0	0	1	0	0	0	0	2	1	1	1	1
276	2022	4	M	3	0	0	0	0	0	0	0	0	0	0	1	0	0	0	0	0	0	0	0	0
277	2022	4	F	3	0	0	0	0	0	0	0	0	0	0	0	0	0	0	1	0	0	0	0	0
278	2022	4	F	3	0	0	0	0	0	0	0	0	0	0	1	0	0	0	0	2	1	1	1	1
279	2022	5	F	3	0	0	0	0	0	0	0	0	0	0	0	0	0	0	0	0	0	0	0	0
280	2022	5	M	3	0	0	0	0	0	0	0	0	0	0	0	0	0	0	0	1	0	0	0	0
281	2022	6	F	3	0	0	0	0	0	0	0	0	0	0	1	1	0	0	0	2	1	1	1	1
282	2022	6	M	3	0	0	0	0	0	0	0	0	0	0	1	0	0	0	0	2	1	1	1	1
283	2022	7	F	3	0	0	0	0	0	0	0	0	0	0	0	0	0	0	0	1	0	0	0	0
284	2022	7	F	3	0	0	0	0	0	0	0	0	0	0	0	0	0	0	0	2	0	0	0	0
285	2022	9	M	3	0	0	0	0	0	0	0	0	0	0	0	0	0	0	1	0	0	0	0	0
286	2022	9	F	3	0	0	0	0	0	0	0	0	0	0	1	0	0	0	1	1	0	0	0	0
287	2022	10	F	3	0	0	0	0	0	0	0	0	0	0	1	0	1	0	0	2	1	1	1	1
288	2022	10	M	3	0	0	0	0	0	0	0	0	0	0	1	1	0	0	0	0	0	0	0	0
289	2022	11	M	3	0	0	0	0	0	0	0	0	0	0	0	0	0	0	0	2	1	0	1	1
290	2022	11	M	3	0	0	0	0	0	0	0	0	0	0	0	0	0	0	0	2	1	0	0	0
291	2022	11	F	3	0	0	0	0	0	0	0	0	0	0	0	0	0	0	0	1	0	0	1	1
292	2022	12	M	3	0	0	0	0	0	0	0	0	0	0	1	0	0	0	0	2	0	1	1	1
293	2023	3	M	3	0	0	0	0	0	0	0	0	0	0	1	0	0	0	1	2	1	1	0	1
294	2023	5	F	3	0	0	0	0	0	0	0	0	0	0	0	0	0	0	1	2	1	0	0	0
295	2023	5	M	3	0	0	0	0	0	0	0	0	0	0	0	0	0	0	0	0	0	0	0	0
296	2023	5	F	3	0	0	0	0	0	0	0	0	0	0	0	0	0	0	0	2	0	1	0	0
297	2023	5	F	3	0	0	0	0	0	0	0	0	0	0	1	0	0	0	0	0	0	0	0	0
298	2023	5	M	3	0	0	0	0	0	0	0	0	0	0	0	0	0	0	1	2	0	1	0	0
299	2023	6	F	3	0	0	0	0	0	0	0	0	0	0	0	0	0	0	0	0	0	0	0	0
300	2023	7	M	3	0	0	0	0	0	0	0	0	0	0	1	0	0	0	0	0	0	0	0	0
301	2024	3	F	3	0	0	0	0	0	0	0	0	0	0	0	0	0	0	0	0	0	0	0	0
302	2024	3	M	3	0	0	0	0	0	0	0	0	0	0	0	0	0	0	0	1	0	0	0	0
303	2024	4	F	3	0	0	0	0	0	0	0	0	0	0	1	0	0	0	0	0	0	0	0	0
304	2024	8	F	3	0	0	0	0	0	0	0	0	0	0	0	0	0	0	0	0	0	0	0	0
305	2024	9	M	3	0	0	0	0	0	0	0	0	0	0	1	1	0	0	0	2	0	1	1	1
306	2024	10	F	3	0	0	0	0	0	0	0	0	0	0	0	0	0	0	1	2	1	1	1	1
307	2024	12	F	3	0	0	0	0	0	0	0	0	0	0	0	0	0	0	0	1	0	1	0	0
308	2021	4	M	3	0	0	0	0	0	0	0	1	0	0	0	0	0	0	1	0	0	0	0	0
309	2021	9	M	3	0	0	0	0	0	0	0	1	0	0	1	1	0	0	0	0	0	0	0	0
310	2021	9	M	3	0	0	0	0	0	0	0	1	0	0	1	0	0	0	0	2	1	1	1	1
311	2021	9	F	3	0	0	0	0	0	0	0	1	0	0	0	0	0	0	1	1	0	1	0	0
312	2021	3	M	3	0	0	0	0	0	0	0	1	0	0	1	0	0	0	0	2	1	0	0	0
313	2021	8	M	3	0	0	0	0	0	0	0	0	0	1	1	0	0	0	0	0	0	0	0	0
314	2022	9	M	3	0	0	0	0	0	0	0	1	0	0	0	0	1	0	0	2	1	1	0	1
315	2022	11	M	3	0	0	0	0	0	0	0	1	0	0	0	0	0	0	1	2	1	1	1	1

ID	Yr	Mo	Sex	Age	Tre	Grs	Rag	Mug	HDM	Dan	Mld	Food	Drg	Ven	Rhi	Cnj	Ast	AsE	Urt	Sev	Prt	Obs	Rho	Snz
316	2023	4	M	3	0	0	0	0	0	0	0	1	0	0	0	0	0	0	1	2	0	1	0	0
317	2024	9	M	3	0	0	0	0	0	0	0	0	1	0	0	0	0	0	0	1	0	0	0	0
318	2021	8	M	3	0	1	0	0	0	0	0	0	0	0	1	0	0	0	1	2	0	0	1	0
319	2022	6	M	3	0	0	0	0	1	0	0	0	0	0	1	1	0	0	0	1	0	1	0	0
320	2022	7	M	3	0	0	0	0	0	0	1	0	0	0	1	0	0	0	0	2	0	1	1	0
321	2022	9	M	3	0	0	0	0	1	0	0	0	0	0	1	0	0	0	0	1	0	1	1	0
322	2022	12	M	3	0	0	0	0	1	0	0	0	0	0	1	0	1	0	0	2	1	1	1	1
323	2023	3	F	3	0	0	0	0	1	0	0	0	0	0	1	0	0	0	0	0	0	1	1	1
324	2021	9	F	3	0	0	0	0	1	0	0	0	0	1	1	0	0	0	0	0	0	0	0	0
325	2023	5	M	3	0	0	0	0	0	0	1	1	0	0	1	0	0	0	0	0	0	0	0	0
326	2020	11	M	3	1	0	1	0	0	0	0	0	0	0	0	0	0	0	0	1	0	0	1	1
327	2022	5	M	3	0	1	0	0	0	0	1	0	0	0	1	0	0	0	0	0	0	0	0	0
328	2022	12	M	3	0	1	0	0	0	0	1	0	0	0	1	0	0	0	0	0	0	0	0	0
329	2023	8	F	3	1	0	0	1	0	0	0	0	0	0	1	0	0	0	0	1	1	1	1	1
330	2023	11	F	3	0	0	0	0	1	1	0	0	0	0	1	0	1	0	0	0	0	0	0	0
331	2021	8	F	3	1	1	0	0	0	0	0	1	0	0	1	1	0	0	0	2	0	1	1	0
332	2022	4	M	3	0	1	1	0	0	0	0	1	0	1	1	1	0	0	0	2	1	0	1	1
333	2023	8	F	3	0	0	1	1	1	0	0	0	0	0	1	1	0	0	0	1	0	1	0	0
334	2023	10	M	3	1	1	1	0	0	1	1	1	0	0	1	0	0	0	1	0	0	0	0	0
335	2020	1	M	4	0	0	0	0	0	0	0	0	0	0	0	0	0	0	1	0	0	0	0	0
336	2020	2	M	4	0	0	0	0	0	0	0	0	0	0	0	0	0	0	1	2	1	1	1	1
337	2020	2	F	4	0	0	0	0	0	0	0	0	0	0	0	0	0	0	1	1	1	0	0	0
338	2020	2	M	4	0	0	0	0	0	0	0	0	0	0	1	1	0	0	0	1	1	1	1	1
339	2020	9	F	4	0	0	0	0	0	0	0	0	0	0	1	0	1	0	0	0	0	0	0	0
340	2020	9	M	4	0	0	0	0	0	0	0	0	0	0	1	1	1	0	0	0	0	0	0	0
341	2021	1	M	4	0	0	0	0	0	0	0	0	0	0	0	0	0	0	1	2	1	1	1	1
342	2021	5	M	4	0	0	0	0	0	0	0	0	0	0	0	0	0	0	1	0	0	0	0	0
343	2021	6	M	4	0	0	0	0	0	0	0	0	0	0	1	0	0	0	0	0	0	0	0	0
344	2021	8	F	4	0	0	0	0	0	0	0	0	0	0	0	0	0	0	1	2	0	1	1	0
345	2021	8	F	4	0	0	0	0	0	0	0	0	0	0	0	0	0	0	0	0	0	0	0	0
346	2021	9	M	4	0	0	0	0	0	0	0	0	0	0	0	0	0	0	0	0	0	0	0	0
347	2021	9	M	4	0	0	0	0	0	0	0	0	0	0	1	0	0	0	0	0	0	0	0	0
348	2021	10	M	4	0	0	0	0	0	0	0	0	0	0	1	0	0	0	0	1	0	1	0	0
349	2021	10	F	4	0	0	0	0	0	0	0	0	0	0	0	0	0	0	0	0	0	0	0	0
350	2021	8	F	4	0	0	0	0	0	0	0	0	0	0	0	0	0	1	0	1	0	0	0	1
351	2021	10	F	4	0	0	0	0	0	0	0	0	0	0	0	0	0	1	0	2	1	0	0	0
352	2022	1	M	4	0	0	0	0	0	0	0	0	0	0	1	0	0	0	0	1	0	0	0	0
353	2022	1	F	4	0	0	0	0	0	0	0	0	0	0	0	0	0	0	0	2	1	1	1	1
354	2022	1	M	4	0	0	0	0	0	0	0	0	0	0	0	0	0	0	0	0	0	0	0	0
355	2022	2	M	4	0	0	0	0	0	0	0	0	0	0	0	0	0	0	0	0	1	0	0	0
356	2022	2	F	4	0	0	0	0	0	0	0	0	0	0	0	0	0	0	0	1	0	1	0	0
357	2022	2	M	4	0	0	0	0	0	0	0	0	0	0	0	0	0	0	0	0	0	0	0	0
358	2022	2	F	4	0	0	0	0	0	0	0	0	0	0	0	0	0	0	1	1	0	1	1	1
359	2022	3	F	4	0	0	0	0	0	0	0	0	0	0	1	0	0	0	0	0	0	0	0	0
360	2022	3	M	4	0	0	0	0	0	0	0	0	0	0	1	0	0	0	0	0	0	0	0	0
361	2022	3	M	4	0	0	0	0	0	0	0	0	0	0	0	0	0	0	0	0	1	0	1	1
362	2022	4	F	4	0	0	0	0	0	0	0	0	0	0	0	0	0	0	0	0	0	0	0	0
363	2022	5	M	4	0	0	0	0	0	0	0	0	0	0	0	0	0	0	0	1	0	1	0	0
364	2022	5	F	4	0	0	0	0	0	0	0	0	0	0	1	0	0	0	0	2	1	1	1	1
365	2022	5	M	4	0	0	0	0	0	0	0	0	0	0	0	0	0	0	1	0	0	0	0	0
366	2022	6	M	4	0	0	0	0	0	0	0	0	0	0	0	0	0	0	1	2	1	1	1	1
367	2022	6	M	4	0	0	0	0	0	0	0	0	0	0	0	0	1	0	0	0	0	0	0	0
368	2022	7	M	4	0	0	0	0	0	0	0	0	0	0	0	0	0	0	1	0	0	0	0	0
369	2022	9	M	4	0	0	0	0	0	0	0	0	0	0	0	0	0	0	0	2	1	1	1	1
370	2022	9	M	4	0	0	0	0	0	0	0	0	0	0	0	0	0	0	0	1	1	1	1	1
371	2022	10	F	4	0	0	0	0	0	0	0	0	0	0	1	0	0	0	0	0	0	0	0	0
372	2022	11	M	4	0	0	0	0	0	0	0	0	0	0	1	0	0	0	0	0	0	0	0	0
373	2022	12	F	4	0	0	0	0	0	0	0	0	0	0	0	0	0	0	1	2	0	1	1	1
374	2023	1	F	4	0	0	0	0	0	0	0	0	0	0	1	0	0	0	1	2	0	0	0	0
375	2023	1	M	4	0	0	0	0	0	0	0	0	0	0	1	0	0	0	0	2	1	1	1	0
376	2023	2	F	4	0	0	0	0	0	0	0	0	0	0	1	0	0	0	0	1	0	0	0	0
377	2023	3	F	4	0	0	0	0	0	0	0	0	0	0	0	0	0	0	0	2	1	1	0	1
378	2023	4	M	4	0	0	0	0	0	0	0	0	0	0	0	0	1	0	0	0	0	0	0	0

ID	Yr	Mo	Sex	Age	Tre	Grs	Rag	Mug	HDM	Dan	Mld	Food	Drg	Ven	Rhi	Cnj	Ast	AsE	Urt	Sev	Prt	Obs	Rho	Snz
379	2023	5	F	4	0	0	0	0	0	0	0	0	0	0	1	0	0	0	0	2	1	1	1	1
380	2023	5	M	4	0	0	0	0	0	0	0	0	0	0	0	0	0	0	0	2	1	1	1	0
381	2023	5	M	4	0	0	0	0	0	0	0	0	0	0	0	0	0	0	0	1	0	0	0	0
382	2023	5	M	4	0	0	0	0	0	0	0	0	0	0	0	0	0	0	1	1	0	0	0	0
383	2023	6	F	4	0	0	0	0	0	0	0	0	0	0	0	0	0	0	0	0	0	0	0	0
384	2023	7	M	4	0	0	0	0	0	0	0	0	0	0	1	0	0	0	0	2	0	1	1	0
385	2023	10	F	4	0	0	0	0	0	0	0	0	0	0	0	0	0	0	1	1	0	1	1	0
386	2023	11	F	4	0	0	0	0	0	0	0	0	0	0	0	0	0	0	1	2	0	1	1	1
387	2023	11	M	4	0	0	0	0	0	0	0	0	0	0	0	0	0	0	0	1	0	1	0	0
388	2023	11	F	4	0	0	0	0	0	0	0	0	0	0	0	0	1	0	0	0	0	0	0	0
389	2024	1	M	4	0	0	0	0	0	0	0	0	0	0	0	0	0	0	1	1	1	1	1	0
390	2024	1	F	4	0	0	0	0	0	0	0	0	0	0	0	0	0	0	1	1	1	1	1	1
391	2024	2	F	4	0	0	0	0	0	0	0	0	0	0	0	0	0	0	1	1	0	1	0	0
392	2024	2	F	4	0	0	0	0	0	0	0	0	0	0	0	0	0	0	1	2	0	0	0	0
393	2024	2	F	4	0	0	0	0	0	0	0	0	0	0	0	0	0	0	1	2	1	1	1	1
394	2024	3	F	4	0	0	0	0	0	0	0	0	0	0	0	0	0	0	0	1	1	1	1	0
395	2024	4	F	4	0	0	0	0	0	0	0	0	0	0	0	0	0	0	0	1	0	1	0	0
396	2024	4	F	4	0	0	0	0	0	0	0	0	0	0	0	0	0	0	0	2	1	1	0	0
397	2024	5	F	4	0	0	0	0	0	0	0	0	0	0	1	0	0	0	0	1	0	0	0	0
398	2024	7	M	4	0	0	0	0	0	0	0	0	0	0	0	0	1	0	0	1	0	1	0	0
399	2024	8	M	4	0	0	0	0	0	0	0	0	0	0	1	0	0	0	0	2	0	0	0	0
400	2024	10	F	4	0	0	0	0	0	0	0	0	0	0	0	0	0	0	0	0	0	0	0	0
401	2024	10	F	4	0	0	0	0	0	0	0	0	0	0	1	0	1	0	0	1	0	0	0	0
402	2020	1	M	4	0	0	0	0	0	0	0	0	1	0	0	0	0	0	0	1	0	0	0	0
403	2021	9	M	4	0	0	0	0	0	0	0	0	0	1	1	0	0	0	0	1	0	1	0	0
404	2021	12	F	4	0	0	0	0	0	0	0	1	0	0	0	0	0	0	0	2	1	1	1	0
405	2022	6	M	4	0	0	0	0	0	0	0	1	0	0	0	0	0	0	1	0	0	0	0	0
406	2023	5	M	4	0	0	0	0	0	0	0	1	0	0	0	0	0	0	0	1	0	1	0	0
407	2023	7	F	4	0	0	0	0	0	0	0	0	0	1	0	0	0	0	1	0	0	0	0	0
408	2023	11	F	4	0	0	0	0	0	0	0	0	1	0	0	0	0	0	0	0	0	0	0	0
409	2024	5	M	4	0	0	0	0	0	0	0	0	0	1	0	0	0	0	0	1	1	1	1	1
410	2024	9	M	4	0	0	0	0	0	0	0	1	0	0	0	0	0	0	0	2	1	1	1	1
411	2021	12	M	4	0	0	0	0	0	0	0	1	0	1	0	0	0	0	0	2	0	0	0	0
412	2020	1	F	4	0	0	0	0	1	0	0	0	0	0	1	0	0	0	0	1	0	1	0	0
413	2020	12	F	4	0	0	0	0	1	0	0	0	0	0	1	0	0	0	1	2	1	1	1	1
414	2021	1	M	4	0	0	0	0	1	0	0	0	0	0	1	0	0	0	0	1	1	1	1	1
415	2023	1	M	4	0	0	1	0	0	0	0	0	0	0	1	0	0	0	0	0	0	0	0	0
416	2023	6	F	4	0	0	1	0	0	0	0	0	0	0	1	0	0	0	0	0	0	0	0	0
417	2022	4	M	4	0	0	0	0	0	1	0	0	0	0	1	0	0	0	0	2	1	1	1	0
418	2022	5	F	4	0	0	0	0	1	0	0	0	0	0	1	1	0	0	0	2	0	1	0	0
419	2022	5	M	4	0	0	0	0	1	0	0	0	0	0	1	0	0	0	0	0	0	0	0	0
420	2022	10	F	4	0	0	0	0	1	0	0	0	0	0	1	1	1	0	0	0	0	0	0	0
421	2022	10	M	4	0	0	0	0	1	0	0	0	0	0	1	1	0	0	0	2	0	0	0	0
422	2022	10	M	4	0	0	0	0	1	0	0	0	0	0	1	0	0	0	0	2	1	0	1	1
423	2022	11	F	4	0	1	0	0	0	0	0	0	0	0	1	1	0	0	0	1	0	1	0	1
424	2022	11	M	4	0	0	0	0	1	0	0	0	0	0	1	0	1	0	0	2	0	1	1	0
425	2022	11	M	4	0	0	0	0	1	0	0	0	0	0	1	0	0	0	0	2	1	1	1	1
426	2022	12	M	4	0	0	0	1	0	0	0	0	0	0	1	0	1	0	0	1	0	1	0	0
427	2022	12	M	4	0	0	0	0	1	0	0	0	0	0	1	0	0	0	0	2	0	1	1	1
428	2023	3	M	4	0	0	0	0	1	0	0	0	0	0	0	0	0	0	1	2	0	1	0	0
429	2023	5	M	4	0	1	0	0	0	0	0	0	0	0	1	1	0	0	0	2	0	1	1	1
430	2023	8	M	4	0	0	0	0	0	1	0	0	0	0	1	0	0	0	0	1	0	0	0	0
431	2023	9	M	4	0	0	0	0	1	0	0	0	0	0	1	0	1	0	0	1	1	0	0	0
432	2023	11	M	4	0	0	0	0	0	0	1	0	0	0	1	0	0	0	0	2	1	1	1	1
433	2024	2	M	4	0	1	0	0	0	0	0	0	0	0	1	1	0	0	1	2	1	0	1	1
434	2024	6	M	4	0	0	0	0	0	1	0	0	0	0	1	0	0	0	0	0	0	0	0	0
435	2024	9	F	4	0	0	0	0	1	0	0	0	0	0	1	1	1	0	0	0	0	0	0	0
436	2021	9	M	4	0	0	0	0	1	0	0	0	0	1	1	0	0	1	0	1	0	1	0	0
437	2023	1	M	4	1	0	0	0	0	0	0	1	0	0	1	0	0	0	1	2	0	0	0	0
438	2020	2	M	4	0	0	0	1	0	0	1	0	0	0	1	0	0	0	0	2	0	1	1	0
439	2020	6	M	4	0	1	0	0	0	0	1	0	0	0	1	0	0	0	0	0	0	0	0	0
440	2020	7	F	4	0	1	0	0	1	0	0	0	0	0	1	0	1	0	0	2	1	1	1	1
441	2021	2	F	4	1	0	0	0	1	0	0	0	0	0	1	1	0	0	0	2	0	0	1	0

ID	Yr	Mo	Sex	Age	Tre	Grs	Rag	Mug	HDM	Dan	Mld	Food	Drg	Ven	Rhi	Cnj	Ast	AsE	Urt	Sev	Prt	Obs	Rho	Snz
442	2020	11	F	4	0	1	1	0	0	0	0	0	0	0	1	1	0	0	0	0	0	0	0	0
443	2021	3	M	4	0	0	1	0	1	0	0	0	0	0	1	1	0	0	0	0	0	0	0	0
444	2021	11	M	4	0	1	1	0	0	0	0	0	0	0	1	0	0	0	0	0	0	0	0	0
445	2021	11	F	4	0	0	1	0	0	0	1	0	0	0	1	0	1	0	0	0	1	1	1	1
446	2022	3	M	4	0	0	1	1	0	0	0	0	0	0	1	0	1	0	0	2	1	1	1	1
447	2023	2	F	4	0	0	1	0	1	0	0	0	0	0	1	0	0	0	1	0	0	0	0	0
448	2022	2	M	4	0	0	0	0	1	0	1	0	0	0	1	0	0	0	0	0	0	0	0	0
449	2022	5	M	4	0	1	0	0	1	0	0	0	0	0	1	1	0	0	0	2	1	1	1	1
450	2024	4	M	4	0	0	0	0	1	0	1	0	0	0	1	0	0	0	0	2	1	0	1	1
451	2024	11	M	4	0	0	0	0	1	0	1	0	0	0	1	1	1	0	0	0	0	0	0	0
452	2021	11	F	4	0	0	1	0	0	0	1	0	0	1	0	1	0	0	0	2	1	1	1	1
453	2021	10	M	4	1	0	1	0	0	1	0	0	0	0	1	1	0	0	0	2	1	0	1	1
454	2024	10	F	4	1	0	1	0	0	1	0	0	0	0	1	1	1	0	0	0	0	0	0	0
455	2022	11	M	4	0	1	0	0	1	1	0	0	0	0	1	0	0	0	0	2	1	1	1	1
456	2024	4	F	4	0	1	0	0	1	0	1	0	0	0	1	1	1	0	0	2	1	0	0	0
457	2020	6	F	4	1	1	1	0	0	0	0	1	0	0	1	1	0	0	0	2	0	0	0	0
458	2022	3	M	4	1	0	1	0	0	1	0	1	0	0	1	0	0	0	0	1	0	0	0	0
459	2024	11	M	4	0	1	1	0	0	0	1	1	0	0	1	1	0	0	0	0	0	0	0	0
460	2020	10	M	4	1	1	0	1	0	0	1	0	0	0	1	0	0	0	0	0	0	0	0	0
461	2020	9	F	4	0	0	1	0	1	1	1	0	0	0	1	0	1	1	0	1	0	0	0	0
462	2020	10	M	4	1	1	1	0	0	1	0	1	0	0	1	1	0	0	0	1	0	1	0	0
463	2021	9	M	4	1	1	1	0	0	0	1	0	0	1	1	0	0	0	0	0	0	0	0	0
464	2020	1	M	5	0	0	0	0	0	0	0	0	0	0	0	0	0	0	0	2	0	0	0	0
465	2020	2	F	5	0	0	0	0	0	0	0	0	0	0	0	0	0	0	0	0	0	0	0	0
466	2020	2	F	5	0	0	0	0	0	0	0	0	0	0	0	0	0	0	1	0	0	0	0	0
467	2020	6	F	5	0	0	0	0	0	0	0	0	0	0	1	0	1	0	0	2	1	1	1	1
468	2020	8	F	5	0	0	0	0	0	0	0	0	0	0	0	0	0	0	1	1	1	0	0	0
469	2020	8	F	5	0	0	0	0	0	0	0	0	0	0	1	0	0	0	1	0	0	0	0	0
470	2020	9	F	5	0	0	0	0	0	0	0	0	0	0	1	0	0	0	0	0	0	0	0	0
471	2020	9	M	5	0	0	0	0	0	0	0	0	0	0	1	0	0	0	0	1	1	1	0	0
472	2020	10	F	5	0	0	0	0	0	0	0	0	0	0	0	0	1	0	0	2	1	1	1	1
473	2021	2	M	5	0	0	0	0	0	0	0	0	0	0	0	0	0	0	0	0	0	0	0	0
474	2021	4	F	5	0	0	0	0	0	0	0	0	0	0	0	0	0	0	1	0	0	0	0	0
475	2021	6	F	5	0	0	0	0	0	0	0	0	0	0	0	0	0	0	1	0	0	0	0	0
476	2021	9	M	5	0	0	0	0	0	0	0	0	0	0	0	0	0	0	0	0	0	0	1	1
477	2021	9	F	5	0	0	0	0	0	0	0	0	0	0	1	0	0	0	0	1	0	1	0	0
478	2021	11	F	5	0	0	0	0	0	0	0	0	0	0	0	0	0	0	1	0	0	0	0	0
479	2021	11	F	5	0	0	0	0	0	0	0	0	0	0	0	0	1	0	0	1	0	0	0	0
480	2021	12	F	5	0	0	0	0	0	0	0	0	0	0	0	0	0	0	1	2	0	1	1	1
481	2021	6	F	5	0	0	0	0	0	0	0	0	0	0	0	0	0	1	0	2	1	1	1	1
482	2021	3	F	5	0	0	0	0	0	0	0	0	0	0	0	0	1	0	1	1	0	1	1	1
483	2021	5	F	5	0	0	0	0	0	0	0	0	0	0	0	0	0	1	0	2	0	1	0	0
484	2021	5	F	5	0	0	0	0	0	0	0	0	0	0	0	0	0	1	0	0	0	0	0	0
485	2021	2	F	5	0	0	0	0	0	0	0	0	0	0	0	0	1	0	1	0	0	0	0	0
486	2022	1	M	5	0	0	0	0	0	0	0	0	0	0	0	0	0	0	0	2	0	1	1	1
487	2022	1	M	5	0	0	0	0	0	0	0	0	0	0	0	0	0	0	0	2	0	1	1	0
488	2022	3	M	5	0	0	0	0	0	0	0	0	0	0	1	0	0	0	0	0	0	0	0	0
489	2022	3	F	5	0	0	0	0	0	0	0	0	0	0	0	0	0	0	1	1	0	1	0	0
490	2022	3	F	5	0	0	0	0	0	0	0	0	0	0	1	0	0	0	0	0	0	0	0	0
491	2022	4	M	5	0	0	0	0	0	0	0	0	0	0	1	0	0	0	0	2	1	1	1	1
492	2022	5	M	5	0	0	0	0	0	0	0	0	0	0	0	0	0	0	0	0	0	0	0	0
493	2022	5	M	5	0	0	0	0	0	0	0	0	0	0	0	0	0	0	0	0	0	0	0	0
494	2022	8	M	5	0	0	0	0	0	0	0	0	0	0	1	0	0	0	1	1	0	1	1	1
495	2022	8	F	5	0	0	0	0	0	0	0	0	0	0	0	0	1	0	0	1	0	0	0	0
496	2022	8	F	5	0	0	0	0	0	0	0	0	0	0	0	0	0	0	1	0	0	0	0	0
497	2022	9	M	5	0	0	0	0	0	0	0	0	0	0	1	0	0	0	0	2	0	1	0	0
498	2022	9	M	5	0	0	0	0	0	0	0	0	0	0	0	0	1	0	0	0	0	0	0	0
499	2022	9	F	5	0	0	0	0	0	0	0	0	0	0	1	0	1	0	1	2	1	1	1	1
500	2022	10	M	5	0	0	0	0	0	0	0	0	0	0	1	0	0	0	0	0	0	0	0	0
501	2022	11	F	5	0	0	0	0	0	0	0	0	0	0	1	0	0	0	0	0	0	0	0	0
502	2022	11	M	5	0	0	0	0	0	0	0	0	0	0	0	0	0	0	0	1	1	1	0	1
503	2022	11	F	5	0	0	0	0	0	0	0	0	0	0	1	0	0	0	0	0	0	0	0	0
504	2022	11	F	5	0	0	0	0	0	0	0	0	0	0	0	0	1	0	0	2	0	0	0	1

ID	Yr	Mo	Sex	Age	Tre	Grs	Rag	Mug	HDM	Dan	Mld	Food	Drg	Ven	Rhi	Cnj	Ast	AsE	Urt	Sev	Prt	Obs	Rho	Snz
505	2022	11	F	5	0	0	0	0	0	0	0	0	0	0	0	0	0	0	0	1	0	1	1	1
506	2022	11	F	5	0	0	0	0	0	0	0	0	0	0	1	0	1	0	1	2	1	1	1	1
507	2023	1	M	5	0	0	0	0	0	0	0	0	0	0	1	0	0	0	0	2	0	0	1	0
508	2023	1	F	5	0	0	0	0	0	0	0	0	0	0	0	0	1	0	0	2	1	1	1	1
509	2023	2	M	5	0	0	0	0	0	0	0	0	0	0	1	0	0	0	0	1	0	0	0	0
510	2023	3	F	5	0	0	0	0	0	0	0	0	0	0	1	0	1	0	0	0	0	0	0	0
511	2023	3	F	5	0	0	0	0	0	0	0	0	0	0	1	0	0	0	0	0	0	0	0	0
512	2023	9	F	5	0	0	0	0	0	0	0	0	0	0	0	0	0	0	1	1	0	0	0	0
513	2023	10	M	5	0	0	0	0	0	0	0	0	0	0	0	0	0	0	0	1	0	0	0	1
514	2023	10	F	5	0	0	0	0	0	0	0	0	0	0	0	0	0	0	1	0	0	0	0	0
515	2023	11	M	5	0	0	0	0	0	0	0	0	0	0	1	0	0	0	0	0	0	0	0	0
516	2023	11	F	5	0	0	0	0	0	0	0	0	0	0	0	0	1	0	0	0	0	0	0	0
517	2024	1	F	5	0	0	0	0	0	0	0	0	0	0	0	0	1	0	0	2	1	1	1	1
518	2024	1	M	5	0	0	0	0	0	0	0	0	0	0	1	0	0	0	0	2	0	0	1	0
519	2024	2	M	5	0	0	0	0	0	0	0	0	0	0	1	1	1	0	1	2	0	1	0	0
520	2024	2	F	5	0	0	0	0	0	0	0	0	0	0	0	0	1	0	0	0	0	0	0	0
521	2024	2	M	5	0	0	0	0	0	0	0	0	0	0	0	0	1	0	0	2	0	0	1	0
522	2024	3	F	5	0	0	0	0	0	0	0	0	0	0	1	0	0	0	0	0	0	0	0	0
523	2024	3	F	5	0	0	0	0	0	0	0	0	0	0	1	0	1	0	0	2	1	1	0	1
524	2024	4	F	5	0	0	0	0	0	0	0	0	0	0	0	0	0	0	0	2	1	0	1	1
525	2024	6	F	5	0	0	0	0	0	0	0	0	0	0	0	0	0	0	1	2	1	1	1	1
526	2024	7	F	5	0	0	0	0	0	0	0	0	0	0	0	0	0	0	1	2	0	0	0	0
527	2024	7	F	5	0	0	0	0	0	0	0	0	0	0	0	0	0	0	0	0	0	0	0	0
528	2024	7	M	5	0	0	0	0	0	0	0	0	0	0	0	0	0	0	1	2	0	0	0	0
529	2024	8	F	5	0	0	0	0	0	0	0	0	0	0	0	0	0	0	0	0	0	0	0	0
530	2024	10	F	5	0	0	0	0	0	0	0	0	0	0	1	0	0	0	0	0	0	0	0	0
531	2020	1	F	5	0	0	0	0	0	0	0	1	0	0	0	0	0	0	1	1	0	1	1	0
532	2020	10	M	5	0	0	0	0	0	0	0	1	0	0	1	0	0	0	0	1	0	1	0	0
533	2021	10	M	5	0	0	0	0	0	0	0	0	0	1	0	0	0	0	0	2	0	0	0	0
534	2021	12	M	5	0	0	0	0	0	0	0	0	0	1	0	1	0	1	0	2	0	1	1	0
535	2021	11	M	5	0	0	0	0	0	0	0	1	0	0	0	0	0	0	0	0	0	0	0	0
536	2022	1	F	5	0	0	0	0	0	0	0	0	1	0	1	0	1	0	0	1	1	1	1	1
537	2022	9	M	5	0	0	0	0	0	0	0	1	0	0	0	0	0	0	1	0	0	0	0	0
538	2023	3	M	5	0	0	0	0	0	0	0	1	0	0	0	0	0	0	1	2	0	0	0	0
539	2023	6	F	5	0	0	0	0	0	0	0	0	1	0	0	0	0	0	1	2	1	1	0	0
540	2024	4	M	5	0	0	0	0	0	0	0	1	0	0	0	0	0	0	0	0	0	0	0	0
541	2024	10	M	5	0	0	0	0	0	0	0	1	0	0	0	0	0	0	1	2	1	0	1	1
542	2020	2	M	5	0	1	0	0	0	0	0	0	0	0	1	0	0	0	0	2	0	0	1	1
543	2020	9	M	5	0	0	0	0	1	0	0	0	0	0	1	0	1	0	0	0	0	0	0	0
544	2020	9	M	5	0	0	0	0	0	0	1	0	0	0	1	0	0	0	0	0	0	0	0	0
545	2021	1	F	5	0	0	0	0	1	0	0	0	0	0	1	1	0	0	1	0	0	0	0	0
546	2021	6	M	5	0	0	0	0	1	0	0	0	0	0	0	0	1	0	0	1	0	1	1	1
547	2021	10	M	5	0	0	0	0	1	0	0	0	0	0	1	0	1	0	0	1	0	0	0	0
548	2022	3	M	5	0	1	0	0	0	0	0	0	0	0	1	0	1	0	0	0	0	0	0	0
549	2022	4	M	5	0	0	0	0	0	1	0	0	0	0	1	0	0	0	0	0	0	0	0	0
550	2022	5	M	5	0	0	0	0	1	0	0	0	0	0	1	0	1	0	0	1	0	1	1	0
551	2022	6	M	5	0	0	0	0	1	0	0	0	0	0	1	0	0	0	0	1	0	1	0	0
552	2022	8	M	5	0	0	0	0	1	0	0	0	0	0	1	0	1	0	0	1	0	0	0	0
553	2022	11	M	5	1	0	0	0	0	0	0	0	0	0	0	0	1	0	0	2	0	1	1	0
554	2023	6	F	5	0	0	0	0	1	0	0	0	0	0	1	0	1	0	0	2	1	1	1	1
555	2021	10	F	5	0	0	0	0	1	0	0	0	0	1	1	0	0	0	0	2	1	1	0	1
556	2022	5	F	5	0	0	0	0	1	0	0	1	0	0	1	0	0	0	0	1	0	1	1	0
557	2024	4	M	5	0	0	0	0	1	0	0	0	1	0	1	1	0	0	0	1	0	1	0	0
558	2024	7	M	5	0	0	0	0	1	0	0	0	1	0	1	1	0	0	0	0	0	0	0	0
559	2024	7	M	5	1	0	0	0	0	0	0	1	0	0	1	0	1	0	1	2	0	1	1	1
560	2024	10	F	5	0	0	0	0	0	1	0	1	0	0	1	0	0	0	0	0	0	0	0	0
561	2020	6	M	5	1	1	0	0	0	0	0	0	0	0	1	1	0	0	0	2	1	1	1	1
562	2020	7	M	5	0	0	0	0	1	0	1	0	0	0	1	0	0	0	0	2	1	1	1	1
563	2020	7	M	5	0	0	0	0	1	1	0	0	0	0	1	0	0	0	0	2	0	0	0	1
564	2021	11	F	5	0	0	1	0	1	0	0	0	0	0	1	0	0	0	0	1	0	1	0	0
565	2022	6	M	5	0	1	1	0	0	0	0	0	0	0	1	1	0	0	0	0	0	0	0	0
566	2022	4	M	5	0	1	0	0	0	0	1	0	0	0	1	0	1	0	0	2	0	1	0	0
567	2022	7	M	5	0	1	0	0	1	0	0	0	0	0	1	1	0	0	0	0	0	0	0	0

ID	Yr	Mo	Sex	Age	Tre	Grs	Rag	Mug	HDM	Dan	Mld	Food	Drg	Ven	Rhi	Cnj	Ast	AsE	Urt	Sev	Prt	Obs	Rho	Snz
568	2023	5	F	5	0	0	0	0	1	0	1	0	0	0	1	0	0	0	0	2	0	1	1	0
569	2023	6	M	5	1	1	0	0	0	0	0	0	0	0	1	1	0	0	0	1	0	1	1	0
570	2023	11	M	5	0	0	0	0	1	1	0	0	0	0	1	0	1	0	1	2	1	0	0	1
571	2021	4	M	5	0	0	0	1	1	0	0	1	0	0	1	0	1	0	1	1	0	1	1	0
572	2021	11	F	5	0	0	1	0	0	0	1	0	0	1	0	0	0	1	0	2	1	0	1	1
573	2020	10	M	5	0	0	0	0	1	1	1	0	0	0	1	0	0	0	0	2	0	1	0	0
574	2021	9	M	5	0	1	0	1	0	1	0	0	0	0	1	1	1	0	0	2	1	1	1	1
575	2020	12	M	5	0	1	1	0	1	0	0	0	0	0	1	0	0	0	1	1	0	0	0	0
576	2021	9	F	5	0	0	1	1	0	1	0	0	0	0	1	1	0	0	0	1	0	0	0	0
577	2022	12	M	5	1	0	1	0	0	1	0	0	0	0	1	1	1	0	0	1	0	1	0	1
578	2023	6	F	5	1	1	1	0	0	0	0	0	0	0	1	1	0	0	0	2	1	1	1	1
579	2023	7	M	5	0	1	1	0	0	0	1	0	0	0	1	0	1	0	0	0	0	0	0	0
580	2023	11	F	5	0	1	1	0	0	1	0	0	0	0	1	0	1	0	0	2	0	1	1	0
581	2022	6	M	5	1	1	0	0	1	0	0	0	0	0	1	0	1	0	0	0	0	0	0	0
582	2020	7	M	5	1	1	0	0	1	0	0	1	0	0	1	1	0	0	0	1	0	1	0	1
583	2023	8	M	5	0	1	0	1	1	0	0	1	0	0	1	0	0	0	1	0	0	0	0	0
584	2021	12	M	5	0	0	1	1	1	0	0	1	0	1	0	0	0	1	0	1	0	1	1	1
585	2022	4	M	5	1	1	0	0	0	0	1	1	1	0	1	1	0	0	0	1	1	0	0	0
586	2020	9	M	5	1	1	0	1	0	0	1	0	0	0	1	1	1	0	0	0	0	0	0	0
587	2020	11	F	5	0	1	1	0	0	1	1	0	0	0	1	1	0	0	0	0	0	0	0	0
588	2022	10	F	5	0	1	1	0	1	1	0	0	0	0	1	0	1	0	0	0	0	0	0	0
589	2020	11	M	5	1	1	0	1	1	1	1	0	0	0	1	0	0	0	0	1	1	0	1	1
590	2020	2	M	6	0	0	0	0	0	0	0	0	0	0	0	0	1	0	0	0	0	0	0	0
591	2020	3	M	6	0	0	0	0	0	0	0	0	0	0	0	0	0	0	0	2	1	1	1	1
592	2020	3	F	6	0	0	0	0	0	0	0	0	0	0	0	0	0	0	1	0	1	0	0	0
593	2020	7	M	6	0	0	0	0	0	0	0	0	0	0	1	0	1	0	0	1	0	0	1	0
594	2020	7	M	6	0	0	0	0	0	0	0	0	0	0	0	0	0	0	0	0	0	0	0	0
595	2020	9	M	6	0	0	0	0	0	0	0	0	0	0	0	0	1	0	0	2	0	1	1	1
596	2020	9	F	6	0	0	0	0	0	0	0	0	0	0	1	0	1	0	0	2	1	1	1	1
597	2021	1	M	6	0	0	0	0	0	0	0	0	0	0	0	0	0	0	0	1	0	1	0	1
598	2021	6	M	6	0	0	0	0	0	0	0	0	0	0	1	0	1	0	0	0	0	0	0	0
599	2021	7	F	6	0	0	0	0	0	0	0	0	0	0	0	0	1	0	1	0	0	0	0	0
600	2021	8	M	6	0	0	0	0	0	0	0	0	0	0	0	0	0	0	0	0	0	0	0	0
601	2021	8	M	6	0	0	0	0	0	0	0	0	0	0	0	0	0	0	0	2	0	1	0	0
602	2021	10	M	6	0	0	0	0	0	0	0	0	0	0	0	0	0	0	0	2	1	1	1	1
603	2021	10	F	6	0	0	0	0	0	0	0	0	0	0	0	0	0	0	0	2	1	1	1	1
604	2021	12	M	6	0	0	0	0	0	0	0	0	0	0	0	0	0	0	0	0	0	0	0	0
605	2021	12	M	6	0	0	0	0	0	0	0	0	0	0	0	0	0	0	0	0	0	0	0	0
606	2021	12	F	6	0	0	0	0	0	0	0	0	0	0	0	1	0	0	0	0	0	0	0	0
607	2021	10	M	6	0	0	0	0	0	0	0	0	0	0	0	0	0	0	1	0	0	0	0	0
608	2021	10	M	6	0	0	0	0	0	0	0	0	0	0	0	1	0	0	0	2	1	0	1	1
609	2021	5	M	6	0	0	0	0	0	0	0	0	0	0	0	0	0	1	0	1	1	0	1	1
610	2022	2	M	6	0	0	0	0	0	0	0	0	0	0	0	0	0	0	0	1	0	1	0	0
611	2022	3	F	6	0	0	0	0	0	0	0	0	0	0	1	0	0	0	0	1	1	1	0	0
612	2022	4	F	6	0	0	0	0	0	0	0	0	0	0	1	0	0	0	0	1	0	0	0	1
613	2022	4	F	6	0	0	0	0	0	0	0	0	0	0	1	0	0	0	0	0	0	0	0	0
614	2022	5	M	6	0	0	0	0	0	0	0	0	0	0	0	0	1	0	0	0	0	0	0	0
615	2022	8	M	6	0	0	0	0	0	0	0	0	0	0	1	0	1	0	0	1	1	1	1	1
616	2022	9	F	6	0	0	0	0	0	0	0	0	0	0	1	1	0	0	0	0	0	0	0	0
617	2022	9	M	6	0	0	0	0	0	0	0	0	0	0	0	0	0	0	1	0	0	0	0	0
618	2023	2	F	6	0	0	0	0	0	0	0	0	0	0	0	0	0	0	0	1	0	0	0	0
619	2023	3	F	6	0	0	0	0	0	0	0	0	0	0	1	0	0	0	1	0	0	0	0	0
620	2023	4	M	6	0	0	0	0	0	0	0	0	0	0	1	0	0	0	0	0	0	0	0	0
621	2023	5	F	6	0	0	0	0	0	0	0	0	0	0	1	0	0	0	0	1	1	0	0	1
622	2023	6	M	6	0	0	0	0	0	0	0	0	0	0	1	1	0	0	0	2	0	1	0	0
623	2023	6	F	6	0	0	0	0	0	0	0	0	0	0	0	0	0	0	1	0	0	0	0	0
624	2023	8	M	6	0	0	0	0	0	0	0	0	0	0	0	0	0	0	1	1	0	1	1	1
625	2023	8	F	6	0	0	0	0	0	0	0	0	0	0	0	0	0	0	1	1	1	0	1	1
626	2023	8	M	6	0	0	0	0	0	0	0	0	0	0	1	0	0	0	0	0	0	1	1	1
627	2023	11	F	6	0	0	0	0	0	0	0	0	0	0	1	0	0	0	0	1	0	1	0	0
628	2023	12	M	6	0	0	0	0	0	0	0	0	0	0	1	0	0	0	0	2	0	1	1	1
629	2024	3	M	6	0	0	0	0	0	0	0	0	0	0	0	0	0	1	0	0	0	0	0	0
630	2024	4	F	6	0	0	0	0	0	0	0	0	0	0	0	0	0	0	0	2	1	1	1	1

ID	Yr	Mo	Sex	Age	Tre	Grs	Rag	Mug	HDM	Dan	Mld	Food	Drg	Ven	Rhi	Cnj	Ast	AsE	Urt	Sev	Prt	Obs	Rho	Snz
631	2024	4	M	6	0	0	0	0	0	0	0	0	0	0	0	0	0	0	0	2	0	1	0	0
632	2024	5	M	6	0	0	0	0	0	0	0	0	0	0	0	0	0	0	1	0	0	0	0	0
633	2024	5	F	6	0	0	0	0	0	0	0	0	0	0	0	0	0	0	0	2	0	1	1	1
634	2024	7	M	6	0	0	0	0	0	0	0	0	0	0	0	0	0	0	1	2	0	1	0	0
635	2024	7	M	6	0	0	0	0	0	0	0	0	0	0	0	0	0	0	1	2	0	0	0	0
636	2024	8	F	6	0	0	0	0	0	0	0	0	0	0	0	0	0	0	1	1	0	1	0	0
637	2024	8	F	6	0	0	0	0	0	0	0	0	0	0	0	0	0	0	0	1	1	0	1	1
638	2024	9	M	6	0	0	0	0	0	0	0	0	0	0	0	0	0	0	1	2	1	1	1	1
639	2024	9	M	6	0	0	0	0	0	0	0	0	0	0	0	1	0	0	0	0	0	0	0	0
640	2024	10	M	6	0	0	0	0	0	0	0	0	0	0	0	0	0	0	0	2	0	1	0	0
641	2024	11	M	6	0	0	0	0	0	0	0	0	0	0	0	0	1	0	0	1	0	1	0	0
642	2020	1	M	6	0	0	0	0	0	0	0	1	0	0	0	0	0	0	0	1	1	1	1	1
643	2020	9	M	6	0	0	0	0	0	0	0	0	0	1	1	0	0	0	0	0	0	0	0	0
644	2021	10	F	6	0	0	0	0	0	0	0	0	0	1	0	0	0	0	0	2	0	1	1	0
645	2022	4	M	6	0	0	0	0	0	0	0	1	0	0	0	0	1	0	0	2	0	0	0	0
646	2023	9	M	6	0	0	0	0	0	0	0	0	0	1	0	0	0	0	0	2	1	1	1	1
647	2023	10	M	6	0	0	0	0	0	0	0	0	1	0	0	0	0	0	0	2	1	0	0	0
648	2020	1	M	6	0	0	0	0	1	0	0	0	0	0	1	0	1	0	1	0	0	0	0	0
649	2020	1	M	6	0	0	0	0	1	0	0	0	0	0	1	0	1	0	0	0	0	0	0	0
650	2020	6	M	6	0	0	0	0	1	0	0	0	0	0	1	0	1	0	0	0	0	0	0	0
651	2020	6	F	6	0	0	0	0	1	0	0	0	0	0	1	0	1	0	0	2	0	1	1	1
652	2021	3	M	6	0	0	0	0	1	0	0	0	0	0	1	0	0	0	0	0	0	0	0	0
653	2021	5	M	6	0	1	0	0	0	0	0	0	0	0	1	1	0	0	0	2	0	0	1	1
654	2021	6	M	6	0	1	0	0	0	0	0	0	0	0	1	1	0	0	0	2	0	1	0	0
655	2021	9	M	6	0	0	0	0	1	0	0	0	0	0	1	0	1	0	0	1	1	0	0	1
656	2020	10	M	6	0	0	1	0	0	0	0	0	0	0	1	0	1	0	0	0	0	0	0	0
657	2022	11	F	6	0	0	1	0	0	0	0	0	0	0	1	0	1	0	0	2	0	1	0	1
658	2022	4	F	6	0	0	0	0	1	0	0	0	0	0	1	1	0	0	0	1	0	0	0	0
659	2022	6	M	6	0	0	0	0	1	0	0	0	0	0	1	0	1	0	0	1	1	0	0	0
660	2023	4	F	6	0	0	0	0	1	0	0	0	0	0	1	0	1	0	0	0	0	0	0	0
661	2023	6	M	6	0	0	0	0	1	0	0	0	0	0	1	0	1	0	1	2	1	1	1	1
662	2023	6	M	6	0	0	0	0	1	0	0	0	0	0	1	0	1	0	0	0	0	0	0	0
663	2023	6	M	6	0	0	0	0	1	0	0	0	0	0	1	1	0	0	0	0	0	0	0	0
664	2023	10	M	6	0	1	0	0	0	0	0	0	0	0	1	0	1	0	0	1	1	0	1	1
665	2023	11	F	6	0	0	0	0	0	0	1	0	0	0	1	0	1	0	1	0	0	0	0	0
666	2024	3	M	6	0	1	0	0	0	0	0	0	0	0	1	0	1	0	1	2	0	0	0	0
667	2024	4	M	6	0	0	0	0	1	0	0	0	0	0	1	1	0	0	0	1	0	1	1	1
668	2024	8	M	6	0	0	0	0	0	0	1	0	0	0	1	0	1	0	0	2	1	1	1	1
669	2024	10	F	6	0	0	0	0	1	0	0	0	0	0	1	1	1	0	0	2	0	1	1	1
670	2021	4	F	6	1	0	0	0	0	0	0	1	0	0	1	1	0	0	1	2	0	0	0	0
671	2021	5	M	6	0	0	0	0	1	0	0	0	0	1	0	0	0	0	0	2	1	1	1	1
672	2023	1	F	6	0	0	0	0	1	0	0	1	1	0	1	0	1	0	1	2	0	1	0	1
673	2020	1	F	6	0	0	0	0	1	1	0	0	0	0	1	0	1	0	0	2	1	0	1	0
674	2020	1	F	6	0	0	0	0	1	1	0	0	0	0	1	0	1	0	0	0	0	0	0	0
675	2020	1	M	6	0	1	0	0	0	0	1	0	0	0	1	0	0	0	1	0	0	0	0	0
676	2020	9	M	6	0	1	0	0	0	1	0	0	0	0	1	1	0	0	0	0	0	0	0	0
677	2021	2	M	6	1	1	0	0	0	0	0	0	0	0	1	1	1	0	0	1	1	0	1	1
678	2022	5	M	6	0	0	1	0	1	0	0	0	0	0	1	0	1	0	0	0	0	0	0	0
679	2022	12	F	6	0	0	1	0	0	1	0	0	0	0	1	0	1	1	0	0	0	1	0	1
680	2024	10	M	6	0	1	1	0	0	0	0	0	0	0	1	0	1	0	0	0	0	0	0	0
681	2022	4	M	6	0	1	0	0	1	0	0	0	0	0	1	0	0	0	0	0	1	0	1	0
682	2022	7	M	6	0	0	0	0	1	1	0	0	0	0	0	0	1	1	1	0	0	0	0	0
683	2022	9	F	6	0	1	0	0	0	1	0	0	0	0	1	0	1	1	0	1	0	0	1	0
684	2023	5	F	6	0	0	0	0	1	0	1	0	0	0	1	0	1	0	0	0	0	0	0	0
685	2023	7	M	6	0	0	0	0	1	1	0	0	0	0	1	0	1	0	0	0	0	0	0	0
686	2023	11	M	6	1	0	0	0	1	0	0	0	0	0	1	0	0	0	0	1	1	0	1	1
687	2024	3	F	6	1	1	0	0	0	0	0	0	0	0	1	1	0	0	0	1	0	1	0	0
688	2024	4	M	6	0	1	0	0	1	0	0	0	0	0	1	1	1	0	0	0	0	1	1	1
689	2024	8	F	6	0	1	0	0	0	1	0	0	0	0	1	1	0	0	1	0	0	0	0	0
690	2021	5	M	6	0	1	0	0	1	0	0	0	0	1	0	0	0	0	0	0	0	0	0	0
691	2021	11	M	6	0	0	1	0	1	0	0	1	0	1	0	1	0	0	0	0	0	0	0	0
692	2020	9	M	6	1	1	0	1	0	0	0	0	0	0	1	1	0	0	0	2	1	1	1	1
693	2021	2	M	6	0	1	0	0	1	0	1	0	0	0	1	1	0	0	0	1	0	0	0	0

ID	Yr	Mo	Sex	Age	Tre	Grs	Rag	Mug	HDM	Dan	Mld	Food	Drg	Ven	Rhi	Cnj	Ast	AsE	Urt	Sev	Prt	Obs	Rho	Snz
694	2020	8	M	6	0	0	1	1	0	1	0	0	0	0	1	1	0	0	0	0	0	0	0	0
695	2022	9	F	6	1	0	1	1	0	0	0	0	0	0	1	0	1	0	0	2	0	1	0	1
696	2024	12	F	6	0	0	1	0	0	1	1	0	0	0	1	0	0	0	0	1	0	0	0	0
697	2023	5	F	6	0	1	0	0	1	1	0	0	0	0	1	0	1	0	1	1	0	0	0	0
698	2023	11	M	6	0	1	0	0	1	0	1	0	0	0	1	0	1	0	0	0	0	0	0	0
699	2024	1	M	6	0	1	0	0	1	1	0	1	0	0	1	0	0	0	0	2	1	0	1	1
700	2020	1	M	7	0	0	0	0	0	0	0	0	0	0	0	0	1	0	0	0	0	0	0	0
701	2020	1	M	7	0	0	0	0	0	0	0	0	0	0	0	0	0	0	1	2	0	1	1	1
702	2020	3	M	7	0	0	0	0	0	0	0	0	0	0	0	0	0	0	1	0	0	0	0	0
703	2020	5	F	7	0	0	0	0	0	0	0	0	0	0	1	0	0	0	0	2	0	0	0	0
704	2020	8	M	7	0	0	0	0	0	0	0	0	0	0	1	0	0	0	0	2	1	0	1	1
705	2021	5	M	7	0	0	0	0	0	0	0	0	0	0	1	1	0	0	0	2	0	1	0	1
706	2021	10	M	7	0	0	0	0	0	0	0	0	0	0	0	1	0	1	0	0	0	0	1	0
707	2021	2	F	7	0	0	0	0	0	0	0	0	0	0	0	0	0	0	0	0	2	0	1	1
708	2022	1	F	7	0	0	0	0	0	0	0	0	0	0	0	0	0	0	0	0	0	0	0	0
709	2022	2	M	7	0	0	0	0	0	0	0	0	0	0	0	0	0	0	0	0	0	0	0	0
710	2022	4	M	7	0	0	0	0	0	0	0	0	0	0	1	1	0	0	0	0	0	0	0	0
711	2022	4	M	7	0	0	0	0	0	0	0	0	0	0	1	0	0	0	0	2	0	1	1	0
712	2022	5	F	7	0	0	0	0	0	0	0	0	0	0	0	0	0	0	0	2	1	0	1	1
713	2022	5	F	7	0	0	0	0	0	0	0	0	0	0	1	0	0	0	0	0	0	0	0	0
714	2022	6	M	7	0	0	0	0	0	0	0	0	0	0	0	1	0	0	0	0	0	0	0	0
715	2022	9	F	7	0	0	0	0	0	0	0	0	0	0	1	0	0	0	0	0	0	0	1	1
716	2022	10	F	7	0	0	0	0	0	0	0	0	0	0	0	0	0	0	0	2	1	1	1	1
717	2022	10	F	7	0	0	0	0	0	0	0	0	0	0	0	0	0	0	0	0	0	0	0	0
718	2022	11	M	7	0	0	0	0	0	0	0	0	0	0	0	0	0	0	0	0	2	1	1	0
719	2023	2	F	7	0	0	0	0	0	0	0	0	0	0	0	0	0	0	1	0	0	0	0	0
720	2023	3	F	7	0	0	0	0	0	0	0	0	0	0	1	0	1	0	0	2	0	0	0	0
721	2023	5	F	7	0	0	0	0	0	0	0	0	0	0	0	0	0	0	1	0	0	0	0	0
722	2023	7	F	7	0	0	0	0	0	0	0	0	0	0	1	0	0	0	0	1	0	0	0	0
723	2023	10	M	7	0	0	0	0	0	0	0	0	0	0	0	0	0	0	0	2	0	1	0	1
724	2024	1	F	7	0	0	0	0	0	0	0	0	0	0	0	0	0	0	0	1	0	0	0	0
725	2024	2	M	7	0	0	0	0	0	0	0	0	0	0	1	0	1	0	0	2	0	0	1	1
726	2024	4	M	7	0	0	0	0	0	0	0	0	0	0	0	0	0	0	1	1	0	0	1	1
727	2024	5	F	7	0	0	0	0	0	0	0	0	0	0	0	0	1	0	0	2	1	1	1	1
728	2024	5	F	7	0	0	0	0	0	0	0	0	0	0	0	0	0	0	1	1	0	1	0	0
729	2024	8	M	7	0	0	0	0	0	0	0	0	0	0	0	0	0	0	0	0	1	0	1	0
730	2024	8	M	7	0	0	0	0	0	0	0	0	0	0	0	0	0	0	1	0	0	0	0	0
731	2024	9	M	7	0	0	0	0	0	0	0	0	0	0	0	0	0	0	0	1	0	0	0	0
732	2024	10	F	7	0	0	0	0	0	0	0	0	0	0	0	0	0	0	0	1	0	0	0	0
733	2024	12	F	7	0	0	0	0	0	0	0	0	0	0	0	0	1	0	0	2	1	0	1	1
734	2021	7	M	7	0	0	0	0	0	0	0	0	1	0	0	0	1	0	1	2	1	1	0	0
735	2021	10	F	7	0	0	0	0	0	0	0	0	0	1	0	0	0	0	0	0	2	0	1	1
736	2024	8	M	7	0	0	0	0	0	0	0	0	0	1	0	0	0	0	0	0	0	0	0	0
737	2020	1	M	7	0	0	0	0	1	0	0	0	0	0	1	0	1	0	0	2	1	1	0	1
738	2020	1	F	7	0	0	0	0	1	0	0	0	0	0	1	0	0	0	1	2	1	0	1	0
739	2020	2	M	7	0	0	0	0	1	0	0	0	0	0	1	0	1	0	0	0	0	0	0	0
740	2020	7	M	7	0	0	0	0	1	0	0	0	0	0	1	0	1	0	0	0	0	0	0	0
741	2020	7	M	7	0	0	0	0	1	0	0	0	0	0	1	0	1	0	0	0	0	0	0	0
742	2020	9	F	7	0	0	0	0	0	1	0	0	0	0	1	0	1	0	0	0	0	1	1	1
743	2020	11	F	7	0	0	0	0	1	0	0	0	0	0	1	0	1	0	0	0	0	0	0	0
744	2021	6	M	7	0	1	0	0	0	0	0	0	0	0	1	1	0	0	0	0	1	0	0	1
745	2021	6	F	7	1	0	0	0	0	0	0	0	0	0	1	0	0	0	0	1	0	0	0	0
746	2021	11	F	7	0	0	0	0	1	0	0	0	0	0	1	0	1	0	0	0	0	0	0	0
747	2020	10	M	7	0	0	1	0	0	0	0	0	0	0	0	0	0	0	0	0	0	0	0	0
748	2021	11	M	7	0	0	1	0	0	0	0	0	0	0	1	0	1	0	0	1	1	0	0	1
749	2022	10	M	7	0	0	1	0	0	0	0	0	0	0	1	1	0	0	0	2	0	1	0	1
750	2023	9	M	7	0	0	1	0	0	0	0	0	0	0	1	0	0	0	1	2	1	1	1	1
751	2024	2	M	7	0	0	1	0	0	0	0	0	0	0	1	1	0	0	1	2	0	1	1	1
752	2022	2	F	7	0	0	0	0	1	0	0	0	0	0	1	1	0	0	0	0	0	0	0	0
753	2022	9	M	7	0	0	0	0	1	0	0	0	0	0	1	0	1	0	0	0	0	0	1	0
754	2023	2	F	7	0	0	0	0	1	0	0	0	0	0	1	0	0	0	0	0	0	1	0	0
755	2023	5	M	7	0	1	0	0	0	0	0	0	0	0	1	1	0	0	0	0	0	1	1	0
756	2023	6	M	7	0	1	0	0	0	0	0	0	0	0	1	0	0	0	0	2	0	0	0	0

ID	Yr	Mo	Sex	Age	Tre	Grs	Rag	Mug	HDM	Dan	Mld	Food	Drg	Ven	Rhi	Cnj	Ast	AsE	Urt	Sev	Prt	Obs	Rho	Snz
757	2023	9	M	7	0	0	0	0	1	0	0	0	0	0	1	0	0	0	0	2	0	1	1	1
758	2023	9	M	7	0	0	0	0	1	0	0	0	0	0	1	0	1	0	0	2	0	0	1	1
759	2024	3	M	7	0	0	0	0	1	0	0	0	0	0	1	0	1	0	0	0	0	0	0	0
760	2024	6	M	7	0	0	0	0	1	0	0	0	0	0	1	0	1	0	0	2	1	0	1	1
761	2024	7	M	7	0	1	0	0	0	0	0	0	0	0	1	0	1	0	0	0	0	0	0	0
762	2024	11	F	7	0	1	0	0	0	0	0	0	0	0	0	0	1	0	0	0	0	0	0	0
763	2021	9	M	7	0	0	0	0	1	0	0	0	0	1	0	0	0	0	0	0	0	0	0	0
764	2021	11	F	7	0	0	0	0	1	0	0	0	0	1	0	0	0	1	0	1	0	1	0	1
765	2022	4	F	7	0	0	0	0	1	0	0	0	1	0	1	0	0	0	0	1	1	0	0	0
766	2023	12	M	7	0	0	0	0	1	0	0	1	0	0	1	0	0	0	0	2	1	0	1	1
767	2021	4	M	7	0	0	0	0	0	0	1	1	0	1	0	1	0	1	0	0	0	0	0	0
768	2022	10	M	7	0	1	1	0	0	0	0	0	0	0	1	1	0	0	0	0	0	0	0	0
769	2024	2	F	7	1	0	1	0	0	0	0	0	0	0	1	0	0	0	1	0	0	0	0	0
770	2024	3	M	7	0	0	1	0	0	0	1	0	0	0	1	1	0	0	1	0	0	0	0	0
771	2022	5	M	7	1	1	0	0	0	0	0	0	0	0	1	1	0	0	0	1	0	0	0	0
772	2022	10	F	7	0	1	0	1	0	0	0	0	0	0	1	0	0	0	1	0	0	0	0	0
773	2023	5	M	7	0	1	0	0	1	0	0	0	0	0	1	0	0	0	1	2	0	1	1	1
774	2024	10	M	7	0	1	0	0	0	1	0	0	0	0	1	1	1	0	0	0	0	0	0	0
775	2021	9	M	7	1	1	0	1	0	0	0	0	0	0	1	0	1	0	0	0	0	0	0	0
776	2020	1	F	7	0	1	1	0	0	1	0	0	0	0	1	0	1	0	0	2	0	0	1	1
777	2020	7	F	7	0	1	1	1	0	0	0	0	0	0	1	1	0	0	0	0	0	0	0	0
778	2020	9	F	7	1	1	1	0	0	0	0	0	0	0	0	0	0	0	0	0	0	0	0	0
779	2022	10	M	7	0	1	1	1	0	0	0	0	0	0	1	1	0	0	0	2	0	0	0	0
780	2022	10	M	7	0	1	1	0	1	0	0	0	0	0	1	0	1	0	0	2	1	1	1	1
781	2023	2	M	7	1	1	1	0	0	0	0	0	0	0	0	0	0	0	1	0	0	1	0	0
782	2022	1	M	7	0	1	0	0	1	1	0	0	0	0	1	0	0	0	0	0	0	0	0	0
783	2023	3	M	7	0	0	0	0	1	1	1	0	0	0	1	0	0	0	0	0	0	0	0	0
784	2024	2	F	7	0	1	0	0	1	1	0	0	0	0	1	0	0	0	1	2	1	1	1	1
785	2022	10	M	7	0	0	1	0	0	1	1	1	0	0	1	0	0	0	1	0	1	0	1	0
786	2020	10	M	7	0	1	1	0	1	1	0	0	0	0	1	1	1	0	0	0	1	0	1	0
787	2021	9	F	7	0	1	1	0	0	1	1	0	0	0	1	0	0	0	0	2	1	0	1	1
788	2023	7	F	7	1	1	1	0	0	1	0	0	0	0	1	0	0	0	0	1	0	0	0	0
789	2023	8	M	7	1	1	0	1	0	1	0	0	0	0	1	0	1	0	1	0	2	0	1	1
790	2024	11	M	7	1	1	0	1	0	1	0	0	0	0	1	0	1	0	0	0	0	0	0	0
791	2022	11	M	7	0	1	1	0	1	1	0	1	0	1	1	0	0	0	0	1	0	0	0	0
792	2021	10	M	7	1	1	1	1	0	1	1	0	0	0	1	1	0	0	0	1	1	0	1	1
793	2020	1	M	8	0	0	0	0	0	0	0	0	0	0	0	0	0	0	1	0	0	0	0	0
794	2020	1	M	8	0	0	0	0	0	0	0	0	0	0	1	0	1	0	0	2	1	1	1	1
795	2020	3	M	8	0	0	0	0	0	0	0	0	0	0	0	0	0	0	0	0	0	1	1	0
796	2020	6	M	8	0	0	0	0	0	0	0	0	0	0	1	0	1	0	1	0	0	0	0	0
797	2020	7	M	8	0	0	0	0	0	0	0	0	0	0	1	0	0	0	0	2	1	1	1	1
798	2020	7	M	8	0	0	0	0	0	0	0	0	0	0	1	0	0	0	0	0	0	0	0	0
799	2020	8	M	8	0	0	0	0	0	0	0	0	0	0	0	0	0	0	1	0	0	0	0	0
800	2020	9	F	8	0	0	0	0	0	0	0	0	0	0	1	1	0	0	0	0	0	0	0	0
801	2020	9	F	8	0	0	0	0	0	0	0	0	0	0	1	0	1	0	0	1	0	0	0	0
802	2020	9	F	8	0	0	0	0	0	0	0	0	0	0	0	0	0	0	0	0	1	0	1	0
803	2020	12	F	8	0	0	0	0	0	0	0	0	0	0	0	0	0	0	1	0	0	0	0	0
804	2020	12	M	8	0	0	0	0	0	0	0	0	0	0	1	0	0	0	0	2	1	1	1	1
805	2021	1	M	8	0	0	0	0	0	0	0	0	0	0	0	0	0	0	0	0	0	0	0	0
806	2021	6	F	8	0	0	0	0	0	0	0	0	0	0	0	0	0	0	1	0	0	0	0	0
807	2021	6	F	8	0	0	0	0	0	0	0	0	0	0	0	0	0	0	0	0	0	0	0	0
808	2021	7	F	8	0	0	0	0	0	0	0	0	0	0	0	0	0	0	0	0	0	0	0	0
809	2021	11	F	8	0	0	0	0	0	0	0	0	0	0	0	0	0	0	0	0	0	0	0	0
810	2022	4	M	8	0	0	0	0	0	0	0	0	0	0	0	0	0	0	1	2	0	1	0	0
811	2022	5	M	8	0	0	0	0	0	0	0	0	0	0	1	1	0	0	0	0	1	0	1	0
812	2022	7	F	8	0	0	0	0	0	0	0	0	0	0	1	0	0	0	0	0	0	0	0	0
813	2022	7	F	8	0	0	0	0	0	0	0	0	0	0	0	0	0	0	1	1	0	0	0	0
814	2022	8	F	8	0	0	0	0	0	0	0	0	0	0	1	1	1	1	0	0	0	1	1	0
815	2022	10	F	8	0	0	0	0	0	0	0	0	0	0	1	1	0	0	0	0	0	1	0	1
816	2022	9	F	8	0	0	0	0	0	0	0	0	0	0	1	0	1	0	0	0	0	0	0	0
817	2022	12	M	8	0	0	0	0	0	0	0	0	0	0	0	0	0	0	1	2	1	1	1	1
818	2022	12	M	8	0	0	0	0	0	0	0	0	0	0	0	0	0	0	0	1	0	0	0	0
819	2023	2	F	8	0	0	0	0	0	0	0	0	0	0	0	0	0	0	1	2	1	1	1	1

ID	Yr	Mo	Sex	Age	Tre	Grs	Rag	Mug	HDM	Dan	Mld	Food	Drg	Ven	Rhi	Cnj	Ast	AsE	Urt	Sev	Prt	Obs	Rho	Snz
820	2023	4	M	8	0	0	0	0	0	0	0	0	0	0	0	0	0	0	1	2	0	0	1	1
821	2023	10	M	8	0	0	0	0	0	0	0	0	0	0	0	0	0	0	1	1	0	0	0	0
822	2023	11	F	8	0	0	0	0	0	0	0	0	0	0	0	0	1	0	0	2	1	1	1	1
823	2023	11	M	8	0	0	0	0	0	0	0	0	0	0	0	0	1	0	0	2	0	1	1	1
824	2024	1	M	8	0	0	0	0	0	0	0	0	0	0	0	0	0	0	1	0	0	0	0	0
825	2024	1	F	8	0	0	0	0	0	0	0	0	0	0	0	0	0	0	0	0	3	1	1	1
826	2024	2	F	8	0	0	0	0	0	0	0	0	0	0	1	1	0	0	1	2	0	1	0	0
827	2024	3	F	8	0	0	0	0	0	0	0	0	0	0	0	0	0	0	1	0	0	1	1	1
828	2024	6	F	8	0	0	0	0	0	0	0	0	0	0	0	0	0	0	0	0	0	0	0	0
829	2024	7	F	8	0	0	0	0	0	0	0	0	0	0	0	0	0	0	0	2	0	1	1	0
830	2024	10	F	8	0	0	0	0	0	0	0	0	0	0	0	0	0	0	1	2	0	0	0	0
831	2024	11	M	8	0	0	0	0	0	0	0	0	0	0	0	0	1	0	0	2	0	1	1	1
832	2024	11	M	8	0	0	0	0	0	0	0	0	0	0	0	0	1	0	1	2	0	0	1	1
833	2020	5	F	8	0	0	0	0	0	0	0	0	1	0	1	0	1	0	0	2	0	1	1	1
834	2021	10	M	8	0	0	0	0	0	0	0	0	0	1	0	0	0	0	0	0	0	0	0	0
835	2021	5	F	8	0	0	0	0	0	0	0	0	0	1	0	0	0	0	0	2	1	1	0	0
836	2021	10	M	8	0	0	0	0	0	0	0	0	0	1	0	1	0	1	0	0	1	1	1	1
837	2023	3	M	8	0	0	0	0	0	0	0	0	1	0	0	0	0	0	0	2	0	1	1	1
838	2020	2	M	8	0	0	0	0	1	0	0	0	0	0	0	0	1	0	0	0	0	0	0	0
839	2020	8	M	8	0	1	0	0	0	0	0	0	0	0	1	1	0	0	0	0	0	0	0	0
840	2020	12	M	8	1	0	0	0	0	0	0	0	0	0	1	0	0	0	0	0	0	0	0	0
841	2021	8	M	8	0	0	0	0	1	0	0	0	0	0	1	0	1	0	1	0	3	1	1	1
842	2021	12	F	8	0	0	1	0	0	0	0	0	0	0	1	1	0	0	0	0	0	0	0	0
843	2022	6	M	8	0	0	1	0	0	0	0	0	0	0	1	1	0	0	0	0	0	0	0	0
844	2022	7	M	8	0	0	1	0	0	0	0	0	0	0	1	1	0	0	0	0	0	0	0	0
845	2023	9	M	8	0	0	1	0	0	0	0	0	0	0	1	0	1	1	0	0	0	0	0	0
846	2022	1	M	8	0	1	0	0	0	0	0	0	0	0	1	1	1	0	1	2	0	0	0	0
847	2022	8	M	8	0	0	0	0	1	0	0	0	0	0	1	1	1	0	0	0	0	0	0	0
848	2022	9	F	8	0	1	0	0	0	0	0	0	0	0	1	0	1	0	0	1	0	1	1	1
849	2023	3	M	8	0	0	0	0	0	1	0	0	0	0	1	0	1	0	0	1	0	1	0	0
850	2023	8	M	8	0	0	0	0	1	0	0	0	0	0	1	0	1	0	0	0	0	0	0	0
851	2023	9	M	8	0	0	0	0	0	1	0	0	0	0	1	0	1	0	0	2	0	1	1	1
852	2023	10	M	8	0	0	0	0	1	0	0	0	0	0	1	0	0	0	0	0	0	1	1	1
853	2023	10	F	8	0	1	0	0	0	0	0	0	0	0	1	0	0	0	0	0	0	0	0	0
854	2024	3	M	8	0	0	0	0	1	0	0	0	0	0	1	0	0	0	1	0	0	0	0	0
855	2024	9	M	8	0	1	0	0	0	0	0	0	0	0	1	0	1	0	0	1	0	1	0	1
856	2024	11	F	8	0	0	0	0	1	0	0	0	0	0	1	0	1	0	0	0	0	0	0	0
857	2024	1	M	8	0	0	0	0	0	0	1	1	0	0	1	0	1	0	1	0	1	0	0	1
858	2021	11	M	8	0	0	1	0	0	0	0	1	0	1	1	1	0	0	0	2	1	1	1	1
859	2020	1	F	8	0	0	1	0	0	0	1	0	0	0	1	0	0	0	0	1	0	1	0	0
860	2022	8	M	8	0	0	1	0	0	0	1	0	0	0	1	1	0	0	0	2	1	1	1	1
861	2024	11	M	8	0	0	1	0	1	0	0	0	0	0	1	1	0	0	0	2	1	1	1	1
862	2023	11	F	8	0	0	0	0	1	1	0	0	0	0	1	0	1	1	0	2	1	1	1	1
863	2024	4	F	8	0	1	0	0	0	1	0	0	0	0	1	0	1	0	1	0	0	1	0	1
864	2024	9	F	8	0	1	0	0	1	0	0	0	0	0	1	0	0	0	0	0	0	0	0	0
865	2024	9	M	8	0	1	0	1	0	0	0	0	0	0	0	0	1	0	0	1	0	0	0	0
866	2021	10	M	8	0	1	1	0	0	0	0	0	0	1	1	1	0	0	0	2	1	1	1	1
867	2022	6	M	8	1	0	0	0	0	0	1	1	0	0	1	1	0	0	0	0	0	0	0	0
868	2020	7	F	8	0	1	0	0	1	0	1	0	0	0	1	1	0	0	0	0	0	0	0	0
869	2020	1	F	8	0	1	1	0	1	0	0	0	0	0	1	0	0	0	0	2	1	1	1	1
870	2020	7	M	8	1	1	1	0	0	0	0	0	0	0	1	1	0	0	0	0	0	0	0	0
871	2022	10	M	8	0	1	1	1	0	0	0	0	0	0	1	1	0	0	0	1	1	0	1	0
872	2022	10	M	8	1	0	1	1	0	0	0	0	0	0	1	1	0	0	1	2	1	1	1	1
873	2022	11	F	8	0	1	1	0	0	0	1	0	0	0	1	0	0	0	0	1	0	0	0	0
874	2023	8	M	8	0	1	1	0	1	0	0	0	0	0	1	0	0	0	1	2	1	1	0	1
875	2022	1	F	8	0	1	0	0	1	1	0	0	0	0	1	0	1	0	0	0	0	1	0	0
876	2024	11	F	8	1	1	0	0	0	1	0	0	0	0	1	0	0	0	0	0	0	0	0	0
877	2021	9	F	8	1	0	0	1	0	1	0	0	0	1	1	0	0	0	0	0	0	0	0	0
878	2021	5	M	8	1	1	0	1	0	0	0	0	0	1	1	0	0	0	0	2	1	1	1	1
879	2021	8	M	8	0	1	1	1	0	0	1	0	0	0	1	1	0	0	0	2	0	1	1	1
880	2024	9	M	8	0	1	1	0	0	1	1	0	0	0	1	1	0	0	0	0	0	0	0	0
881	2020	8	M	8	1	1	1	0	1	0	1	0	0	0	1	1	0	0	0	0	1	0	1	1
882	2023	7	M	8	1	1	0	0	1	1	1	0	0	0	1	0	0	0	1	1	0	0	0	0

ID	Yr	Mo	Sex	Age	Tre	Grs	Rag	Mug	HDM	Dan	Mld	Food	Drg	Ven	Rhi	Cnj	Ast	AsE	Urt	Sev	Prt	Obs	Rho	Snz
883	2021	9	M	8	1	1	1	1	0	1	0	0	0	1	1	1	0	0	0	2	0	1	1	1
884	2022	12	F	8	1	1	1	1	0	0	1	1	0	0	1	0	0	0	0	0	0	0	0	0
885	2023	11	F	8	1	1	0	0	1	1	1	1	0	0	1	1	1	0	0	2	0	1	0	0
886	2020	2	F	9	0	0	0	0	0	0	0	0	0	0	0	0	0	0	1	0	0	0	0	0
887	2020	2	M	9	0	0	0	0	0	0	0	0	0	0	0	0	0	0	1	0	0	0	0	0
888	2020	3	M	9	0	0	0	0	0	0	0	0	0	0	1	0	0	0	0	2	0	1	1	1
889	2020	9	M	9	0	0	0	0	0	0	0	0	0	0	0	0	1	0	0	0	0	0	0	0
890	2020	9	F	9	0	0	0	0	0	0	0	0	0	0	0	0	1	0	0	2	1	1	1	1
891	2020	10	F	9	0	0	0	0	0	0	0	0	0	0	1	0	0	0	1	0	0	0	0	0
892	2020	11	F	9	0	0	0	0	0	0	0	0	0	0	0	0	0	0	0	0	0	0	0	0
893	2021	1	M	9	0	0	0	0	0	0	0	0	0	0	0	0	0	0	0	0	0	0	0	0
894	2021	2	F	9	0	0	0	0	0	0	0	0	0	0	1	0	0	0	0	2	0	1	1	0
895	2021	9	F	9	0	0	0	0	0	0	0	0	0	0	1	0	0	0	0	1	0	0	1	1
896	2021	10	M	9	0	0	0	0	0	0	0	0	0	0	0	1	0	0	0	1	0	0	0	1
897	2022	1	F	9	0	0	0	0	0	0	0	0	0	0	0	0	0	0	0	1	1	0	0	1
898	2022	3	M	9	0	0	0	0	0	0	0	0	0	0	1	1	1	0	0	0	0	0	0	0
899	2022	5	M	9	0	0	0	0	0	0	0	0	0	0	1	0	1	0	0	2	0	1	1	1
900	2022	5	F	9	0	0	0	0	0	0	0	0	0	0	0	0	0	0	0	2	0	0	1	1
901	2022	7	F	9	0	0	0	0	0	0	0	0	0	0	0	0	0	0	0	0	0	1	1	1
902	2022	8	M	9	0	0	0	0	0	0	0	0	0	0	1	0	1	0	0	1	0	1	1	1
903	2022	9	M	9	0	0	0	0	0	0	0	0	0	0	1	1	0	0	0	2	1	1	0	0
904	2022	12	M	9	0	0	0	0	0	0	0	0	0	0	1	0	1	0	0	0	0	0	0	0
905	2023	1	F	9	0	0	0	0	0	0	0	0	0	0	0	0	1	0	0	2	0	1	0	0
906	2023	4	F	9	0	0	0	0	0	0	0	0	0	0	0	0	0	0	1	1	0	0	0	0
907	2023	5	F	9	0	0	0	0	0	0	0	0	0	0	1	0	0	0	0	0	0	0	0	0
908	2023	6	F	9	0	0	0	0	0	0	0	0	0	0	0	0	0	0	0	2	0	1	0	1
909	2023	10	M	9	0	0	0	0	0	0	0	0	0	0	1	0	1	0	0	0	0	0	1	0
910	2023	10	M	9	0	0	0	0	0	0	0	0	0	0	1	0	0	0	0	0	0	0	0	0
911	2024	1	M	9	0	0	0	0	0	0	0	0	0	0	1	0	0	0	1	1	1	0	1	0
912	2024	3	M	9	0	0	0	0	0	0	0	0	0	0	0	0	0	0	0	0	0	0	0	0
913	2024	3	F	9	0	0	0	0	0	0	0	0	0	0	0	0	0	0	0	0	0	0	0	0
914	2024	4	F	9	0	0	0	0	0	0	0	0	0	0	0	0	0	0	1	1	2	1	1	0
915	2024	5	M	9	0	0	0	0	0	0	0	0	0	0	0	0	0	0	1	0	0	0	0	0
916	2024	5	M	9	0	0	0	0	0	0	0	0	0	0	0	0	0	0	0	0	0	0	0	0
917	2024	5	M	9	0	0	0	0	0	0	0	0	0	0	1	1	0	0	0	2	1	0	1	1
918	2024	8	F	9	0	0	0	0	0	0	0	0	0	0	0	0	0	0	0	0	0	0	0	0
919	2021	10	F	9	0	0	0	0	0	0	0	0	1	0	0	0	0	0	0	0	0	0	0	0
920	2022	5	F	9	0	0	0	0	0	0	0	0	1	0	1	0	0	0	1	2	0	0	0	1
921	2023	5	F	9	0	0	0	0	0	0	0	1	0	0	1	0	0	0	0	1	0	0	0	0
922	2023	7	M	9	0	0	0	0	0	0	0	1	0	0	0	0	0	0	1	0	0	0	0	0
923	2020	7	F	9	0	0	0	0	1	0	0	0	0	0	1	0	0	0	0	1	0	0	0	0
924	2021	1	M	9	0	0	0	0	1	0	0	0	0	0	1	0	0	0	0	2	1	1	0	1
925	2020	6	M	9	0	0	1	0	0	0	0	0	0	0	1	1	1	0	0	0	0	0	1	0
926	2021	4	F	9	0	0	1	0	0	0	0	0	0	0	1	1	0	0	1	1	0	0	0	0
927	2022	1	M	9	0	0	0	0	1	0	0	0	0	0	1	0	0	0	0	0	1	1	1	1
928	2023	4	M	9	0	1	0	0	0	0	0	0	0	0	1	0	0	0	0	2	1	0	0	1
929	2023	8	F	9	0	0	0	0	0	0	1	0	0	0	1	0	1	0	0	0	0	0	0	0
930	2023	9	M	9	0	1	0	0	0	0	0	0	0	0	1	0	0	0	0	2	1	1	1	1
931	2023	10	M	9	0	0	0	0	1	0	0	0	0	0	1	0	0	0	0	0	0	1	1	1
932	2024	4	F	9	0	0	0	0	1	0	0	0	0	0	1	0	1	1	0	2	0	1	0	0
933	2024	4	M	9	0	1	0	0	0	0	0	0	0	0	1	0	0	0	0	0	0	0	0	0
934	2024	6	M	9	0	0	0	0	1	0	0	0	0	0	1	0	1	0	0	1	0	0	0	0
935	2024	10	F	9	0	0	0	0	1	0	0	0	0	0	1	0	1	0	0	0	0	0	0	0
936	2024	10	M	9	0	0	0	0	1	0	0	0	0	0	1	1	0	0	0	0	2	1	1	1
937	2021	10	F	9	0	0	0	0	1	0	0	0	0	1	1	0	0	0	0	0	0	0	0	0
938	2020	1	M	9	0	1	0	0	1	0	0	0	0	0	1	1	1	0	1	0	1	1	1	1
939	2020	7	F	9	0	1	0	0	1	0	0	0	0	0	1	1	0	0	0	0	2	0	1	1
940	2021	3	M	9	0	0	0	0	1	1	0	0	0	0	1	0	1	1	0	0	0	0	0	0
941	2021	1	F	9	0	0	0	0	1	1	0	0	0	0	1	1	0	0	0	2	1	0	1	1
942	2020	7	M	9	0	1	1	0	0	0	0	0	0	0	1	1	0	0	0	2	0	1	1	1
943	2020	8	F	9	1	0	1	0	0	0	0	0	0	0	0	1	0	0	0	2	1	1	1	1
944	2022	6	F	9	0	1	1	0	0	0	0	0	0	0	1	1	1	0	0	1	0	0	1	0
945	2022	10	F	9	0	0	1	0	1	0	0	0	0	0	1	0	1	0	0	2	1	1	0	1

ID	Yr	Mo	Sex	Age	Tre	Grs	Rag	Mug	HDM	Dan	Mld	Food	Drg	Ven	Rhi	Cnj	Ast	AsE	Urt	Sev	Prt	Obs	Rho	Snz
946	2023	5	M	9	0	1	1	0	0	0	0	0	0	0	1	1	0	0	0	0	0	0	0	0
947	2023	9	F	9	0	0	1	0	1	0	0	0	0	0	1	1	0	0	0	0	0	0	0	0
948	2023	1	M	9	0	1	0	0	1	0	0	0	0	0	1	0	1	0	0	2	1	1	1	1
949	2023	7	F	9	0	0	0	0	1	1	0	0	0	0	1	0	1	0	0	0	0	0	0	0
950	2024	6	F	9	0	0	0	0	0	1	1	0	0	0	1	0	1	1	0	2	0	1	1	1
951	2024	9	F	9	0	0	0	0	0	1	1	0	0	0	1	0	1	0	0	0	2	1	1	0
952	2020	6	F	9	1	1	1	0	0	0	0	0	0	0	1	1	0	0	0	2	1	1	1	1
953	2022	5	F	9	0	1	0	0	1	0	1	0	0	0	1	1	0	0	0	1	0	0	0	0
954	2022	6	F	9	0	1	0	0	1	0	1	0	0	0	1	1	0	0	0	2	0	1	1	1
955	2021	10	M	9	0	1	0	0	1	1	0	0	0	1	0	1	1	0	0	1	0	1	0	1
956	2020	9	F	9	1	1	1	0	0	1	0	0	0	0	1	1	0	0	0	2	0	1	1	1
957	2024	3	M	9	1	1	1	1	0	0	0	0	0	0	1	1	0	0	0	0	0	0	0	0
958	2021	8	M	9	1	1	0	0	1	1	1	0	0	0	1	1	0	0	0	2	1	0	1	1
959	2020	1	F	10	0	0	0	0	0	0	0	0	0	0	0	0	0	0	1	1	0	0	1	0
960	2020	3	M	10	0	0	0	0	0	0	0	0	0	0	0	0	0	0	0	2	0	1	1	1
961	2020	6	F	10	0	0	0	0	0	0	0	0	0	0	1	0	0	0	0	0	2	1	1	0
962	2020	6	F	10	0	0	0	0	0	0	0	0	0	0	1	1	0	0	0	1	0	0	0	0
963	2020	9	M	10	0	0	0	0	0	0	0	0	0	0	1	0	0	0	0	0	0	0	0	0
964	2020	10	M	10	0	0	0	0	0	0	0	0	0	0	1	0	1	0	0	0	2	0	1	1
965	2020	12	F	10	0	0	0	0	0	0	0	0	0	0	1	0	1	0	0	2	1	0	1	1
966	2021	1	F	10	0	0	0	0	0	0	0	0	0	0	0	0	0	0	1	2	1	1	1	1
967	2021	6	F	10	0	0	0	0	0	0	0	0	0	0	1	1	0	0	0	2	0	1	1	1
968	2021	9	M	10	0	0	0	0	0	0	0	0	0	0	1	1	0	0	0	1	2	0	1	1
969	2021	2	F	10	0	0	0	0	0	0	0	0	0	0	0	0	1	0	1	2	1	1	1	1
970	2022	1	M	10	0	0	0	0	0	0	0	0	0	0	0	0	0	0	0	0	1	0	0	0
971	2022	4	F	10	0	0	0	0	0	0	0	0	0	0	0	0	0	0	1	1	0	0	0	0
972	2022	5	M	10	0	0	0	0	0	0	0	0	0	0	0	0	0	0	1	0	0	0	0	0
973	2022	8	F	10	0	0	0	0	0	0	0	0	0	0	0	0	0	0	1	0	0	0	0	0
974	2022	8	M	10	0	0	0	0	0	0	0	0	0	0	1	0	0	0	1	0	0	0	0	0
975	2022	9	F	10	0	0	0	0	0	0	0	0	0	0	0	0	0	0	1	0	0	0	0	0
976	2022	9	M	10	0	0	0	0	0	0	0	0	0	0	1	0	0	0	0	0	0	0	0	0
977	2022	10	F	10	0	0	0	0	0	0	0	0	0	0	0	0	1	0	0	1	0	0	0	0
978	2022	11	M	10	0	0	0	0	0	0	0	0	0	0	0	0	1	0	0	0	0	0	0	0
979	2023	1	F	10	0	0	0	0	0	0	0	0	0	0	0	0	0	0	0	2	0	1	1	1
980	2023	4	F	10	0	0	0	0	0	0	0	0	0	0	1	0	0	0	0	2	1	1	0	1
981	2023	8	M	10	0	0	0	0	0	0	0	0	0	0	1	0	1	0	1	0	0	0	0	0
982	2024	2	M	10	0	0	0	0	0	0	0	0	0	0	0	0	0	0	1	1	1	1	1	1
983	2024	2	F	10	0	0	0	0	0	0	0	0	0	0	0	0	0	0	1	0	0	0	1	0
984	2024	3	M	10	0	0	0	0	0	0	0	0	0	0	1	0	0	0	0	1	0	0	0	1
985	2024	8	M	10	0	0	0	0	0	0	0	0	0	0	0	0	0	0	0	2	1	1	0	0
986	2020	6	F	10	0	0	0	0	0	0	0	1	0	0	1	0	1	0	0	0	0	0	0	0
987	2020	7	M	10	0	0	0	0	0	0	0	1	0	0	0	0	0	0	1	0	0	0	0	0
988	2022	11	F	10	0	0	0	0	0	0	0	0	1	0	0	0	0	0	0	2	1	1	1	1
989	2020	1	F	10	0	0	0	0	1	0	0	0	0	0	1	0	1	0	0	0	0	0	0	0
990	2020	1	M	10	0	0	0	0	1	0	0	0	0	0	1	0	0	0	0	0	0	0	0	0
991	2021	4	M	10	0	0	0	0	1	0	0	0	0	0	1	0	1	0	0	2	1	1	1	1
992	2021	12	F	10	0	0	0	0	1	0	0	0	0	0	1	0	1	0	0	2	1	1	1	1
993	2023	8	F	10	0	0	1	0	0	0	0	0	0	0	1	0	0	0	0	1	0	0	0	0
994	2024	9	M	10	0	0	1	0	0	0	0	0	0	0	1	1	0	0	0	0	0	0	0	0
995	2022	3	M	10	0	0	0	0	1	0	0	0	0	0	0	0	0	0	1	2	1	1	1	1
996	2022	9	F	10	1	0	0	0	0	0	0	0	0	0	1	0	0	0	1	0	0	0	0	0
997	2023	11	M	10	0	0	0	0	1	0	0	0	0	0	0	0	1	0	0	0	0	0	0	0
998	2024	4	M	10	0	0	0	0	1	0	0	0	0	0	0	0	1	0	0	0	0	0	0	0
999	2024	4	M	10	0	0	0	0	1	0	0	0	0	0	1	0	0	0	0	0	0	0	0	0
1000	2024	7	M	10	0	0	0	0	1	0	0	0	0	0	1	0	0	0	0	2	1	1	0	0
1001	2024	10	M	10	0	0	0	0	1	0	0	0	0	0	1	0	0	0	0	0	0	0	0	0
1002	2024	11	F	10	0	0	0	0	1	0	0	0	0	0	1	0	0	0	0	0	0	0	0	0
1003	2024	11	F	10	0	0	0	0	1	0	0	0	0	0	1	0	1	0	0	2	0	0	0	0
1004	2024	12	M	10	0	1	0	0	0	0	0	0	0	0	1	0	0	0	0	0	0	0	0	0
1005	2021	12	M	10	0	0	0	0	1	0	0	0	0	1	0	1	0	0	0	2	0	1	0	0
1006	2020	7	M	10	0	1	0	0	0	0	1	0	0	0	1	0	1	0	0	0	0	0	0	0
1007	2021	6	M	10	1	1	0	0	0	0	0	0	0	0	1	1	0	0	1	2	0	1	0	0
1008	2022	11	M	10	0	0	1	0	0	0	1	0	0	0	1	0	1	0	0	0	0	1	0	0

ID	Yr	Mo	Sex	Age	Tre	Grs	Rag	Mug	HDM	Dan	Mld	Food	Drg	Ven	Rhi	Cnj	Ast	AsE	Urt	Sev	Prt	Obs	Rho	Snz
1009	2024	4	M	10	0	0	1	0	0	1	0	0	0	0	1	1	1	0	0	0	0	0	0	0
1010	2024	10	F	10	0	0	1	0	1	0	0	0	0	0	1	1	0	0	0	2	1	1	0	1
1011	2021	12	F	10	0	0	1	0	0	1	0	0	0	0	1	0	0	1	0	0	0	0	0	0
1012	2023	3	M	10	0	0	0	0	1	0	1	0	0	0	1	0	1	0	0	2	1	1	1	1
1013	2021	9	F	10	0	1	0	0	1	0	0	0	0	1	0	1	0	1	1	0	0	0	0	0
1014	2021	12	M	10	0	0	0	0	1	1	0	0	0	1	0	1	0	0	0	0	0	0	0	0
1015	2022	8	M	10	0	0	0	0	1	1	0	1	0	0	1	0	1	0	1	0	0	0	0	0
1016	2024	2	F	10	0	1	0	0	1	0	0	1	0	0	1	0	1	0	0	0	0	0	0	0
1017	2021	5	F	10	0	1	0	0	1	0	1	0	0	0	1	1	0	0	0	0	1	0	1	0
1018	2021	1	M	10	0	1	0	0	1	1	0	0	0	0	1	0	1	0	0	1	0	0	0	0
1019	2020	9	M	10	1	1	1	0	0	0	0	0	0	0	1	1	0	0	0	2	0	0	1	1
1020	2020	11	F	10	0	1	1	1	0	0	0	0	0	0	1	1	0	0	0	2	0	1	1	1
1021	2021	9	M	10	0	1	1	0	0	0	1	0	0	0	1	1	1	0	0	2	0	1	1	1
1022	2022	5	M	10	1	1	1	0	0	0	0	0	0	0	1	1	0	0	0	2	1	0	1	1
1023	2022	9	M	10	1	1	1	0	0	0	0	0	0	0	1	0	0	0	0	2	0	0	1	1
1024	2023	1	M	10	0	1	1	0	1	0	0	0	0	0	1	0	0	0	0	1	0	0	0	0
1025	2023	8	F	10	0	1	1	0	0	0	1	0	0	0	1	0	1	0	0	2	0	0	1	1
1026	2023	9	M	10	0	0	1	0	1	1	0	0	0	0	1	1	0	0	0	0	0	0	0	0
1027	2024	2	F	10	0	1	1	0	1	0	0	0	0	0	1	1	0	0	1	0	1	0	1	0
1028	2024	9	M	10	0	1	1	0	1	0	0	0	0	0	1	1	0	0	1	0	0	0	0	0
1029	2024	10	F	10	0	0	1	0	1	1	0	0	0	0	1	0	0	0	1	0	0	0	0	0
1030	2023	4	F	10	0	0	0	1	1	1	0	0	0	0	1	0	1	0	0	2	0	1	1	1
1031	2021	11	M	10	1	1	0	0	1	0	0	0	0	1	0	0	0	0	0	0	0	0	0	0
1032	2020	5	M	10	1	1	1	0	0	0	1	0	0	0	1	1	0	0	0	0	0	0	0	0
1033	2023	6	M	10	1	1	1	0	1	0	0	0	0	0	1	0	0	0	0	0	0	0	0	0
1034	2023	11	M	10	1	1	1	0	1	0	0	0	0	0	1	1	0	0	0	0	0	0	0	0
1035	2023	7	F	10	0	1	0	0	1	1	1	0	0	0	1	0	0	0	0	0	0	0	0	0
1036	2020	10	F	10	1	0	1	0	1	1	1	0	0	0	1	0	1	0	0	0	0	0	0	0
1037	2021	9	M	10	0	1	1	1	1	1	0	0	0	0	1	1	0	0	0	0	0	1	1	1
1038	2023	11	M	10	1	1	1	0	1	1	0	0	0	0	1	0	1	0	0	2	1	0	1	1
1039	2020	6	M	10	1	1	1	1	1	1	0	0	0	0	1	1	0	0	0	0	1	0	1	0
1040	2021	11	F	10	1	1	1	1	1	0	1	0	0	0	1	1	0	0	1	0	0	0	0	0
1041	2020	7	F	11	0	0	0	0	0	0	0	0	0	0	0	0	0	0	1	0	0	0	0	0
1042	2020	9	F	11	0	0	0	0	0	0	0	0	0	0	1	0	1	0	0	0	0	0	0	0
1043	2020	9	M	11	0	0	0	0	0	0	0	0	0	0	1	0	1	0	0	0	0	0	0	0
1044	2021	3	M	11	0	0	0	0	0	0	0	0	0	0	0	0	0	0	0	1	0	0	0	0
1045	2021	9	M	11	0	0	0	0	0	0	0	0	0	0	0	0	0	0	0	0	0	0	0	0
1046	2021	9	M	11	0	0	0	0	0	0	0	0	0	0	0	0	0	0	1	1	0	0	0	0
1047	2021	11	F	11	0	0	0	0	0	0	0	0	0	0	0	0	0	0	1	2	1	0	1	1
1048	2022	1	F	11	0	0	0	0	0	0	0	0	0	0	0	0	0	0	1	0	1	0	0	0
1049	2022	1	M	11	0	0	0	0	0	0	0	0	0	0	0	0	0	0	0	0	0	0	0	0
1050	2022	4	F	11	0	0	0	0	0	0	0	0	0	0	1	0	1	0	0	1	0	1	0	1
1051	2022	9	M	11	0	0	0	0	0	0	0	0	0	0	0	0	0	0	0	0	0	0	0	0
1052	2022	10	M	11	0	0	0	0	0	0	0	0	0	0	1	0	1	0	0	1	0	0	0	0
1053	2022	11	F	11	0	0	0	0	0	0	0	0	0	0	1	0	0	0	0	0	0	0	0	0
1054	2022	11	F	11	0	0	0	0	0	0	0	0	0	0	0	0	0	0	1	2	1	1	1	1
1055	2022	12	F	11	0	0	0	0	0	0	0	0	0	0	0	0	0	0	1	2	0	1	1	1
1056	2023	5	M	11	0	0	0	0	0	0	0	0	0	0	1	0	1	0	0	2	0	1	1	1
1057	2023	5	M	11	0	0	0	0	0	0	0	0	0	0	1	0	1	0	0	2	1	1	1	1
1058	2023	6	F	11	0	0	0	0	0	0	0	0	0	0	0	0	0	0	0	2	0	1	1	1
1059	2023	6	M	11	0	0	0	0	0	0	0	0	0	0	1	0	0	0	0	0	0	0	0	0
1060	2023	6	F	11	0	0	0	0	0	0	0	0	0	0	1	0	1	0	0	2	0	1	1	1
1061	2023	11	F	11	0	0	0	0	0	0	0	0	0	0	1	0	1	0	0	1	0	0	0	0
1062	2024	2	F	11	0	0	0	0	0	0	0	0	0	0	1	0	1	0	0	2	1	1	1	1
1063	2024	8	F	11	0	0	0	0	0	0	0	0	0	0	0	0	0	0	1	2	1	1	1	1
1064	2024	11	F	11	0	0	0	0	0	0	0	0	0	0	0	0	1	0	0	0	0	0	0	0
1065	2021	11	M	11	0	0	0	0	0	0	0	1	0	0	0	0	0	0	0	1	2	1	1	0
1066	2021	9	F	11	0	0	0	0	0	0	0	1	0	0	0	1	0	1	0	0	0	1	0	1
1067	2022	1	M	11	0	0	0	0	0	0	0	1	0	0	1	1	0	0	0	0	0	0	0	0
1068	2022	5	M	11	0	0	0	0	0	0	0	1	0	0	0	0	0	0	1	0	0	0	0	0
1069	2022	9	M	11	0	0	0	0	0	0	0	1	0	0	0	1	0	0	1	1	0	0	1	1
1070	2020	7	F	11	0	1	0	0	0	0	0	0	0	0	1	1	0	0	0	0	0	0	0	0
1071	2020	9	M	11	0	0	0	0	0	1	0	0	0	0	1	0	0	0	0	0	0	0	0	0

ID	Yr	Mo	Sex	Age	Tre	Grs	Rag	Mug	HDM	Dan	Mld	Food	Drg	Ven	Rhi	Cnj	Ast	AsE	Urt	Sev	Prt	Obs	Rho	Snz
1072	2020	9	M	11	0	0	0	0	0	1	0	0	0	0	1	1	0	0	0	2	0	1	0	0
1073	2020	9	M	11	0	0	0	0	1	0	0	0	0	0	1	0	1	0	0	0	0	0	0	0
1074	2020	10	F	11	0	0	0	0	1	0	0	0	0	0	1	1	1	0	0	0	0	0	0	0
1075	2021	6	F	11	0	1	0	0	0	0	0	0	0	0	0	0	1	0	0	0	0	0	0	0
1076	2021	3	M	11	0	0	0	0	1	0	0	0	0	0	1	1	0	0	0	0	0	0	0	0
1077	2020	10	M	11	0	0	1	0	0	0	0	0	0	0	1	0	0	0	0	1	0	0	0	0
1078	2023	8	M	11	0	0	1	0	0	0	0	0	0	0	1	0	1	0	0	2	1	1	1	0
1079	2021	1	M	11	0	0	1	0	0	0	0	0	0	0	1	1	0	0	1	0	0	0	0	0
1080	2022	4	M	11	0	0	0	0	1	0	0	0	0	0	1	0	0	0	0	0	1	0	1	1
1081	2022	5	M	11	0	0	0	0	1	0	0	0	0	0	1	0	0	0	0	0	0	0	0	0
1082	2022	5	M	11	0	1	0	0	0	0	0	0	0	0	1	1	0	0	0	2	1	1	1	1
1083	2022	8	M	11	0	0	0	0	1	0	0	0	0	0	1	0	0	0	0	0	0	0	0	0
1084	2022	11	M	11	0	0	0	0	1	0	0	0	0	0	1	0	1	0	0	0	0	0	0	0
1085	2023	11	M	11	0	0	0	0	1	0	0	0	0	0	0	0	1	1	0	1	0	1	1	1
1086	2024	4	M	11	0	0	0	0	0	0	1	0	0	0	1	0	1	0	0	0	0	0	0	0
1087	2024	8	M	11	0	0	0	0	1	0	0	0	0	0	1	0	1	0	0	0	0	0	0	0
1088	2021	9	M	11	0	1	0	0	0	0	0	0	0	1	1	0	0	0	0	2	0	1	0	0
1089	2021	9	F	11	0	0	1	0	0	0	0	0	0	1	1	0	0	0	0	2	0	1	1	0
1090	2020	2	M	11	0	0	0	0	1	0	1	0	0	0	1	0	0	0	0	0	1	0	1	0
1091	2020	2	F	11	0	0	0	0	1	0	1	0	0	0	1	1	1	0	0	0	0	0	0	0
1092	2020	7	M	11	0	0	0	0	1	1	0	0	0	0	1	1	1	0	0	2	1	1	1	1
1093	2020	7	M	11	0	1	0	0	0	0	1	0	0	0	1	1	0	0	0	0	1	0	1	1
1094	2020	9	F	11	0	0	0	0	1	1	0	0	0	0	1	1	1	0	0	1	1	1	1	1
1095	2020	9	M	11	0	0	0	0	1	1	0	0	0	0	1	0	1	0	0	0	0	1	0	0
1096	2021	6	M	11	0	1	0	0	1	0	0	0	0	0	1	0	0	0	0	2	0	1	1	1
1097	2021	5	F	11	0	0	0	0	1	1	0	0	0	0	0	0	0	1	0	2	0	1	1	0
1098	2020	7	M	11	0	1	1	0	0	0	0	0	0	0	1	1	1	0	0	0	0	0	0	0
1099	2022	5	F	11	0	0	1	0	0	1	0	0	0	0	1	1	0	0	0	0	0	0	0	0
1100	2022	7	M	11	0	1	1	0	0	0	0	0	0	0	1	1	0	0	1	0	0	0	0	0
1101	2022	12	M	11	0	0	0	0	1	1	0	0	0	0	1	0	1	0	0	0	0	1	0	0
1102	2024	7	M	11	0	0	0	0	1	1	0	0	0	0	1	0	0	0	0	3	0	1	1	1
1103	2024	8	F	11	0	1	0	0	0	0	1	0	0	0	1	0	0	0	0	0	0	0	0	0
1104	2021	9	M	11	0	1	1	0	0	0	0	0	0	1	1	1	0	0	0	0	0	0	0	0
1105	2024	9	M	11	0	1	1	0	0	0	0	0	0	1	0	0	0	0	2	2	0	1	1	1
1106	2021	4	M	11	0	0	0	0	1	1	1	0	0	0	1	0	0	0	0	0	0	0	0	0
1107	2020	9	F	11	0	1	1	0	1	0	0	0	0	0	1	0	1	0	0	1	0	0	0	0
1108	2022	3	M	11	0	1	1	1	0	0	0	0	0	0	1	1	0	0	0	1	0	1	0	0
1109	2022	5	M	11	0	0	1	0	1	1	0	0	0	0	1	1	0	0	1	0	0	0	0	0
1110	2022	11	M	11	1	1	0	0	0	1	0	0	0	0	1	0	0	0	0	1	0	1	0	0
1111	2024	5	M	11	1	1	0	0	1	0	0	0	0	0	1	0	0	0	0	1	0	0	0	0
1112	2021	11	M	11	0	0	1	0	1	0	1	0	0	1	0	0	0	0	0	0	0	0	0	0
1113	2020	6	M	11	0	1	1	1	0	0	1	0	0	0	1	1	1	0	0	2	1	1	1	1
1114	2021	11	F	11	0	1	1	1	1	0	0	0	0	0	1	1	0	0	1	0	0	0	0	0
1115	2024	8	F	11	1	0	1	0	0	1	1	0	0	0	1	0	0	0	0	0	1	1	1	1
1116	2023	10	F	11	1	1	0	0	1	1	0	0	0	0	1	0	1	0	1	2	1	1	1	1
1117	2023	10	M	11	0	1	1	0	0	1	1	1	0	0	1	0	0	0	0	1	0	0	0	0
1118	2024	9	M	11	1	1	0	1	0	0	1	1	0	0	1	0	0	0	0	0	0	0	0	0
1119	2020	2	F	12	0	0	0	0	0	0	0	0	0	0	0	0	0	0	0	0	0	0	0	0
1120	2021	1	M	12	0	0	0	0	0	0	0	0	0	0	0	0	0	0	1	1	0	0	0	0
1121	2021	6	F	12	0	0	0	0	0	0	0	0	0	0	0	0	0	0	1	0	0	0	0	0
1122	2021	6	F	12	0	0	0	0	0	0	0	0	0	0	1	0	1	0	0	0	0	1	1	1
1123	2021	8	M	12	0	0	0	0	0	0	0	0	0	0	1	0	0	0	0	0	0	0	0	0
1124	2021	8	M	12	0	0	0	0	0	0	0	0	0	0	0	0	0	0	0	0	0	0	0	0
1125	2021	8	M	12	0	0	0	0	0	0	0	0	0	0	1	0	0	0	0	0	0	0	0	0
1126	2021	1	F	12	0	0	0	0	0	0	0	0	0	0	0	0	0	0	1	1	0	0	0	0
1127	2021	5	M	12	0	0	0	0	0	0	0	0	0	0	0	0	0	1	0	2	0	1	1	1
1128	2022	6	M	12	0	0	0	0	0	0	0	0	0	0	1	0	1	0	0	2	1	1	1	1
1129	2022	12	F	12	0	0	0	0	0	0	0	0	0	0	0	0	1	0	0	0	0	0	0	0
1130	2023	1	M	12	0	0	0	0	0	0	0	0	0	0	0	0	0	0	0	2	0	1	1	0
1131	2023	3	F	12	0	0	0	0	0	0	0	0	0	0	0	0	0	0	1	0	0	0	0	0
1132	2023	6	F	12	0	0	0	0	0	0	0	0	0	0	0	0	0	0	1	0	0	0	1	0
1133	2023	6	F	12	0	0	0	0	0	0	0	0	0	0	0	0	0	0	1	0	0	0	0	0
1134	2023	6	M	12	0	0	0	0	0	0	0	0	0	0	0	0	0	0	0	0	1	0	1	0

ID	Yr	Mo	Sex	Age	Tre	Grs	Rag	Mug	HDM	Dan	Mld	Food	Drg	Ven	Rhi	Cnj	Ast	AsE	Urt	Sev	Prt	Obs	Rho	Snz
1135	2023	6	F	12	0	0	0	0	0	0	0	0	0	0	0	0	0	0	0	2	0	0	0	0
1136	2023	9	F	12	0	0	0	0	0	0	0	0	0	0	0	0	0	0	1	2	1	1	1	1
1137	2023	10	M	12	0	0	0	0	0	0	0	0	0	0	1	0	1	0	0	1	0	0	1	1
1138	2023	11	M	12	0	0	0	0	0	0	0	0	0	0	1	0	1	0	0	0	0	0	0	0
1139	2024	1	M	12	0	0	0	0	0	0	0	0	0	0	1	0	0	0	0	0	0	0	0	0
1140	2024	1	M	12	0	0	0	0	0	0	0	0	0	0	0	0	0	0	1	0	0	0	0	0
1141	2024	1	M	12	0	0	0	0	0	0	0	0	0	0	0	0	0	0	0	2	1	1	1	1
1142	2024	2	F	12	0	0	0	0	0	0	0	0	0	0	0	0	0	0	0	2	1	1	1	1
1143	2024	3	F	12	0	0	0	0	0	0	0	0	0	0	0	0	0	0	0	0	0	0	0	0
1144	2024	4	F	12	0	0	0	0	0	0	0	0	0	0	0	0	0	0	1	2	0	1	0	0
1145	2024	7	M	12	0	0	0	0	0	0	0	0	0	0	0	0	1	1	0	0	0	0	0	0
1146	2024	8	M	12	0	0	0	0	0	0	0	0	0	0	1	0	0	0	1	0	0	0	0	0
1147	2024	10	F	12	0	0	0	0	0	0	0	0	0	0	1	0	0	0	0	0	0	0	0	0
1148	2021	5	F	12	0	0	0	0	0	0	0	0	0	1	0	0	0	0	0	0	0	0	0	0
1149	2022	1	F	12	0	0	0	0	0	0	0	0	0	1	0	0	0	0	0	2	0	1	0	0
1150	2023	4	F	12	0	0	0	0	0	0	0	0	1	0	0	0	0	0	1	0	0	0	0	0
1151	2020	1	M	12	0	0	0	0	1	0	0	0	0	0	1	1	1	0	0	1	0	0	0	0
1152	2020	7	M	12	0	1	0	0	0	0	0	0	0	0	1	1	1	0	0	0	0	0	0	0
1153	2020	10	M	12	0	0	0	0	0	0	1	0	0	0	1	0	1	0	0	0	0	0	0	0
1154	2021	9	F	12	0	0	1	0	0	0	0	0	0	0	1	1	0	0	0	2	1	1	1	1
1155	2023	2	M	12	0	0	1	0	0	0	0	0	0	0	1	0	0	0	0	1	0	0	0	0
1156	2023	4	M	12	0	0	1	0	0	0	0	0	0	0	1	1	0	0	0	2	0	0	1	1
1157	2023	11	M	12	0	0	1	0	0	0	0	0	0	0	1	1	0	0	0	0	0	0	0	0
1158	2023	11	M	12	0	0	1	0	0	0	0	0	0	0	1	0	1	0	0	1	0	0	0	0
1159	2024	9	M	12	0	0	1	0	0	0	0	0	0	0	1	1	0	0	0	0	1	0	1	0
1160	2024	11	M	12	0	0	1	0	0	0	0	0	0	0	1	1	0	0	0	0	0	1	1	1
1161	2022	6	M	12	0	0	0	0	1	0	0	0	0	0	1	0	0	0	0	0	0	0	0	0
1162	2023	2	M	12	0	0	0	0	1	0	0	0	0	0	1	0	0	0	0	2	0	0	0	0
1163	2023	2	M	12	0	0	0	0	1	0	0	0	0	0	1	0	1	0	0	0	0	0	0	0
1164	2023	11	F	12	0	0	0	0	1	0	0	0	0	0	1	0	0	0	0	0	0	0	0	0
1165	2024	5	F	12	0	0	0	0	0	0	1	0	0	0	1	0	1	0	0	2	0	0	1	1
1166	2024	8	F	12	0	0	0	0	1	0	0	0	0	0	1	0	1	0	0	2	0	1	1	1
1167	2020	1	F	12	0	0	0	0	1	1	0	0	0	0	1	1	0	0	0	1	0	0	0	0
1168	2021	4	F	12	0	0	0	0	1	0	1	0	0	0	1	0	1	0	0	0	1	0	1	0
1169	2021	6	F	12	0	1	0	0	1	0	0	0	0	0	1	0	1	0	0	0	0	1	1	0
1170	2020	7	F	12	0	1	1	0	0	0	0	0	0	0	1	1	0	0	0	0	0	0	0	0
1171	2022	5	F	12	0	0	1	0	1	0	0	0	0	0	1	1	0	0	0	0	0	1	0	0
1172	2023	5	F	12	0	1	1	0	0	0	0	0	0	0	1	0	0	0	0	1	1	1	1	1
1173	2024	5	F	12	0	0	1	0	0	1	0	0	0	0	1	1	1	0	0	0	3	1	1	1
1174	2022	9	M	12	0	0	0	0	1	1	0	0	0	0	1	1	1	0	0	0	0	0	0	0
1175	2022	10	M	12	0	0	0	0	1	1	0	0	0	0	1	0	1	0	0	1	0	1	0	0
1176	2023	3	F	12	1	0	0	0	1	0	0	0	0	0	1	0	0	0	1	0	0	0	0	0
1177	2021	5	M	12	1	1	0	0	0	0	0	0	0	1	1	0	0	0	0	0	0	0	0	0
1178	2021	5	M	12	0	1	1	0	0	0	0	0	0	1	1	1	0	0	1	2	1	0	1	1
1179	2020	11	F	12	0	1	1	0	0	1	0	0	0	0	1	0	0	0	0	1	0	0	1	0
1180	2021	7	M	12	1	1	1	0	0	0	0	0	0	0	1	1	0	0	1	1	0	1	0	0
1181	2021	8	F	12	0	0	1	1	1	0	0	0	0	0	1	1	1	0	1	2	1	1	1	1
1182	2021	11	M	12	1	1	1	0	0	0	0	0	0	0	1	1	0	0	0	2	1	1	1	1
1183	2023	9	M	12	0	1	1	1	0	0	0	0	0	0	1	1	1	0	0	2	1	1	1	1
1184	2024	2	M	12	0	1	1	0	1	0	0	0	0	0	1	1	0	0	0	0	0	0	0	0
1185	2024	10	F	12	0	1	1	0	1	0	0	0	0	0	1	1	0	0	0	0	0	0	0	0
1186	2024	10	F	12	0	1	1	0	1	0	0	0	0	0	1	1	0	0	1	2	1	1	0	0
1187	2024	10	M	12	1	1	0	0	1	0	0	0	0	0	1	0	0	0	0	0	0	0	0	0
1188	2021	9	M	12	0	1	1	0	1	1	0	0	0	0	1	1	0	0	0	0	0	0	0	0
1189	2022	3	M	12	1	1	1	0	1	0	0	0	0	0	1	1	0	0	0	2	0	0	1	0
1190	2022	6	M	12	1	1	1	0	1	0	0	0	0	0	1	1	1	0	0	0	0	0	0	0
1191	2023	1	M	12	1	1	1	0	0	0	1	0	0	0	1	1	0	0	0	2	0	0	1	1
1192	2024	6	M	12	1	1	1	0	1	0	0	0	0	0	1	0	1	0	0	2	1	1	1	1
1193	2023	11	M	12	0	1	0	0	1	1	1	0	0	0	1	0	1	0	0	2	1	1	1	1
1194	2023	10	F	12	1	1	1	0	0	1	0	1	0	0	1	0	0	0	0	0	0	0	0	0
1195	2024	8	M	12	1	1	1	0	0	0	1	0	0	1	1	1	0	0	1	1	0	0	0	0
1196	2021	9	M	12	1	1	1	1	0	0	0	0	0	1	1	0	0	0	0	1	0	1	0	0
1197	2020	2	F	12	0	1	1	1	1	1	0	0	0	0	1	1	0	0	0	0	0	0	0	0

ID	Yr	Mo	Sex	Age	Tre	Grs	Rag	Mug	HDM	Dan	Mld	Food	Drg	Ven	Rhi	Cnj	Ast	AsE	Urt	Sev	Prt	Obs	Rho	Snz
1198	2021	9	F	12	1	1	1	1	0	1	0	0	0	0	1	1	0	0	0	2	1	0	1	1
1199	2023	8	M	12	1	1	1	0	1	0	1	0	0	0	1	1	0	0	0	1	0	1	0	0
1200	2023	11	F	12	1	1	1	0	1	1	0	0	0	0	1	0	0	0	0	2	0	1	1	1
1201	2022	4	F	12	1	1	1	1	1	1	0	0	0	0	1	1	0	0	0	0	0	0	0	0
1202	2020	1	F	13	0	0	0	0	0	0	0	0	0	0	0	0	1	0	0	1	0	0	0	0
1203	2020	2	F	13	0	0	0	0	0	0	0	0	0	0	0	0	0	0	0	0	0	0	0	0
1204	2020	10	M	13	0	0	0	0	0	0	0	0	0	0	0	0	0	0	0	1	0	0	0	0
1205	2020	10	F	13	0	0	0	0	0	0	0	0	0	0	1	1	0	0	0	0	0	0	0	0
1206	2021	5	F	13	0	0	0	0	0	0	0	0	0	0	1	1	0	0	0	2	1	1	0	1
1207	2021	7	F	13	0	0	0	0	0	0	0	0	0	0	0	0	1	0	0	0	0	1	0	0
1208	2022	1	F	13	0	0	0	0	0	0	0	0	0	0	1	0	0	0	1	0	0	0	0	0
1209	2022	3	F	13	0	0	0	0	0	0	0	0	0	0	1	1	0	0	0	2	0	1	1	1
1210	2022	4	M	13	0	0	0	0	0	0	0	0	0	0	0	0	0	0	0	2	1	1	0	1
1211	2022	10	F	13	0	0	0	0	0	0	0	0	0	0	0	0	0	0	0	0	0	0	0	0
1212	2022	12	M	13	0	0	0	0	0	0	0	0	0	0	0	0	0	0	1	2	0	0	1	1
1213	2023	3	F	13	0	0	0	0	0	0	0	0	0	0	0	0	0	0	1	2	1	1	1	1
1214	2023	6	M	13	0	0	0	0	0	0	0	0	0	0	1	0	0	0	0	0	0	1	1	0
1215	2023	12	F	13	0	0	0	0	0	0	0	0	0	0	1	0	0	0	0	2	0	1	0	1
1216	2024	1	F	13	0	0	0	0	0	0	0	0	0	0	0	0	0	0	1	2	1	0	1	1
1217	2024	3	M	13	0	0	0	0	0	0	0	0	0	0	0	0	1	0	0	0	0	0	0	0
1218	2024	4	M	13	0	0	0	0	0	0	0	0	0	0	0	0	0	0	1	0	2	0	1	1
1219	2024	4	F	13	0	0	0	0	0	0	0	0	0	0	1	0	0	0	0	1	0	0	0	0
1220	2021	9	F	13	0	0	0	0	0	0	0	0	0	1	1	0	0	0	0	0	0	0	0	0
1221	2021	11	F	13	0	0	0	0	0	0	0	0	0	1	0	0	0	0	0	2	1	1	1	1
1222	2021	11	F	13	0	0	0	0	0	0	0	0	0	1	0	0	0	0	0	0	0	0	0	0
1223	2022	4	F	13	0	0	0	0	0	0	0	0	1	0	1	0	0	0	1	2	0	1	1	0
1224	2023	4	M	13	0	0	0	0	0	0	0	0	1	0	1	0	1	0	0	2	1	0	1	1
1225	2020	9	F	13	0	0	0	0	1	0	0	0	0	0	1	0	1	0	0	0	0	0	0	0
1226	2021	9	F	13	0	1	0	0	0	0	0	0	0	0	1	0	0	0	0	0	0	0	0	0
1227	2023	8	M	13	0	0	1	0	0	0	0	0	0	0	1	1	0	0	0	1	0	1	1	1
1228	2023	10	M	13	0	0	1	0	0	0	0	0	0	0	1	1	0	0	0	0	0	0	0	0
1229	2022	10	F	13	0	0	0	0	1	0	0	0	0	0	1	0	1	0	0	0	0	1	1	0
1230	2022	12	M	13	0	0	0	0	1	0	0	0	0	0	1	0	1	0	0	2	0	1	0	0
1231	2023	5	F	13	0	0	0	0	1	0	0	0	0	0	1	0	0	0	1	2	0	1	1	1
1232	2023	6	F	13	0	0	0	0	1	0	0	0	0	0	1	0	1	0	0	0	0	0	0	0
1233	2023	6	F	13	0	0	0	0	1	0	0	0	0	0	1	0	1	0	0	0	3	1	1	1
1234	2023	7	M	13	0	0	0	0	1	0	0	0	0	0	1	0	1	0	0	2	1	0	0	1
1235	2023	10	F	13	0	0	0	0	1	0	0	0	0	0	1	0	1	0	0	0	1	0	1	0
1236	2024	4	F	13	0	0	0	0	1	0	0	0	0	0	1	0	1	0	0	2	1	1	1	1
1237	2024	5	M	13	0	0	0	0	1	0	0	0	0	0	1	0	1	0	0	1	0	0	0	0
1238	2024	8	M	13	0	0	0	0	1	0	0	0	0	0	1	0	0	0	0	2	1	0	0	1
1239	2024	7	M	13	0	0	0	0	1	0	0	1	0	0	1	0	1	0	0	0	0	0	0	0
1240	2020	3	F	13	0	0	0	0	1	1	0	0	0	0	1	1	0	0	0	1	0	0	0	0
1241	2020	7	F	13	0	1	0	0	0	0	1	0	0	0	1	1	0	0	0	2	1	1	0	0
1242	2020	11	F	13	1	0	1	0	0	0	0	0	0	0	1	1	0	0	0	1	0	0	0	0
1243	2023	2	F	13	0	1	1	0	0	0	0	0	0	0	1	0	1	0	0	0	3	1	1	1
1244	2023	8	F	13	0	0	1	0	1	0	0	0	0	0	1	0	0	0	0	1	0	0	0	0
1245	2024	1	F	13	0	1	1	0	0	0	0	0	0	0	1	1	0	0	0	0	3	1	1	1
1246	2024	3	M	13	0	1	1	0	0	0	0	0	0	0	1	1	0	0	0	0	0	0	0	0
1247	2024	9	M	13	0	1	1	0	0	0	0	0	0	0	1	1	0	0	0	2	0	1	1	1
1248	2022	10	M	13	0	1	0	0	0	0	1	0	0	0	1	0	1	0	0	2	1	0	1	1
1249	2023	5	M	13	0	1	0	0	0	1	0	0	0	0	1	1	1	0	0	2	0	1	0	0
1250	2024	3	M	13	0	0	0	0	1	1	0	0	0	0	1	0	1	0	0	0	2	1	1	0
1251	2022	4	F	13	0	0	1	0	1	1	0	0	0	0	1	0	1	0	0	2	1	1	1	1
1252	2022	6	F	13	0	0	1	1	0	1	0	0	0	0	1	0	0	0	0	1	0	0	0	0
1253	2023	5	F	13	1	1	1	0	0	0	0	0	0	0	1	1	0	0	0	2	1	0	1	1
1254	2024	9	M	13	0	1	1	0	1	0	0	0	0	0	1	1	1	0	0	2	1	1	0	1
1255	2024	9	F	13	0	0	1	0	1	0	1	0	0	0	1	0	0	0	0	0	0	0	0	0
1256	2021	6	M	13	1	1	0	1	0	1	0	0	0	0	1	1	0	0	0	1	0	0	0	0
1257	2022	6	M	13	0	1	1	1	1	0	0	0	0	0	1	0	0	0	0	2	1	0	1	1
1258	2024	3	M	13	0	1	1	1	0	1	0	0	0	0	1	0	0	0	0	0	0	0	0	0
1259	2024	7	M	13	1	1	1	0	0	0	1	0	0	0	1	0	0	0	0	0	0	0	0	0
1260	2024	8	M	13	1	1	1	0	1	0	1	0	0	0	1	1	0	0	0	0	0	0	0	0

ID	Yr	Mo	Sex	Age	Tre	Grs	Rag	Mug	HDM	Dan	Mld	Food	Drg	Ven	Rhi	Cnj	Ast	AsE	Urt	Sev	Prt	Obs	Rho	Snz
1261	2020	7	M	14	0	0	0	0	0	0	0	0	0	0	1	0	0	0	0	2	1	1	0	1
1262	2020	8	F	14	0	0	0	0	0	0	0	0	0	0	0	0	0	0	1	2	1	1	1	1
1263	2020	9	F	14	0	0	0	0	0	0	0	0	0	0	0	0	1	0	0	1	0	1	0	0
1264	2021	2	F	14	0	0	0	0	0	0	0	0	0	0	0	0	0	0	0	2	1	1	0	0
1265	2021	6	F	14	0	0	0	0	0	0	0	0	0	0	0	0	0	0	1	2	0	1	1	1
1266	2021	9	F	14	0	0	0	0	0	0	0	0	0	0	1	1	0	0	0	0	0	0	0	0
1267	2021	11	F	14	0	0	0	0	0	0	0	0	0	0	1	0	0	0	0	1	0	0	0	0
1268	2021	11	F	14	0	0	0	0	0	0	0	0	0	0	0	0	0	1	0	2	0	1	1	1
1269	2021	1	F	14	0	0	0	0	0	0	0	0	0	0	1	0	0	0	0	1	0	0	0	0
1270	2022	1	M	14	0	0	0	0	0	0	0	0	0	0	0	0	0	0	1	0	0	0	0	0
1271	2022	1	F	14	0	0	0	0	0	0	0	0	0	0	0	0	1	0	0	0	0	0	0	0
1272	2022	8	M	14	0	0	0	0	0	0	0	0	0	0	0	0	0	0	1	1	0	0	1	1
1273	2022	9	F	14	0	0	0	0	0	0	0	0	0	0	0	0	0	0	1	0	0	1	0	0
1274	2022	9	M	14	0	0	0	0	0	0	0	0	0	0	1	0	0	0	0	0	1	0	1	0
1275	2023	1	M	14	0	0	0	0	0	0	0	0	0	0	0	0	0	0	1	1	0	0	0	1
1276	2023	6	F	14	0	0	0	0	0	0	0	0	0	0	0	0	0	0	1	0	0	0	0	0
1277	2023	9	M	14	0	0	0	0	0	0	0	0	0	0	1	0	0	0	0	0	0	0	0	0
1278	2024	10	F	14	0	0	0	0	0	0	0	0	0	0	0	0	0	0	0	0	0	0	0	0
1279	2024	11	M	14	0	0	0	0	0	0	0	0	0	0	0	0	0	0	0	0	0	0	0	0
1280	2023	5	M	14	0	0	0	0	0	0	0	0	0	1	0	0	0	0	0	2	1	1	1	1
1281	2024	5	F	14	0	0	0	0	0	0	0	0	1	0	0	0	0	0	1	1	1	1	1	1
1282	2021	11	F	14	0	0	0	0	0	0	0	1	0	1	0	1	0	1	0	2	0	0	1	1
1283	2022	6	F	14	0	0	0	0	0	0	0	1	1	0	0	0	0	0	0	0	3	1	1	1
1284	2020	7	M	14	0	1	0	0	0	0	0	0	0	0	1	0	0	0	0	2	1	1	1	1
1285	2020	9	M	14	0	0	0	0	1	0	0	0	0	0	1	0	1	0	0	1	0	0	0	0
1286	2021	2	F	14	0	0	0	0	1	0	0	0	0	0	1	0	0	0	1	2	0	1	1	1
1287	2021	2	M	14	0	0	0	0	1	0	0	0	0	0	1	1	0	0	0	0	0	0	0	0
1288	2021	11	F	14	0	0	0	1	0	0	0	0	0	0	1	0	0	0	0	2	1	1	0	0
1289	2021	4	M	14	0	0	1	0	0	0	0	0	0	0	1	0	0	0	0	0	0	0	1	1
1290	2022	4	M	14	0	0	0	0	1	0	0	0	0	0	1	1	1	0	0	1	0	0	0	0
1291	2022	6	M	14	0	0	0	0	1	0	0	0	0	0	1	1	0	0	0	2	1	0	1	1
1292	2022	7	M	14	0	1	0	0	0	0	0	0	0	0	1	0	0	0	0	0	1	0	0	1
1293	2023	5	F	14	0	0	0	0	0	1	0	0	0	0	1	0	0	0	0	0	0	0	1	0
1294	2023	10	F	14	0	0	0	0	1	0	0	0	0	0	1	0	1	0	0	1	0	0	0	0
1295	2023	11	M	14	0	0	0	0	1	0	0	0	0	0	1	0	0	0	0	0	0	0	0	0
1296	2024	4	M	14	0	0	0	0	1	0	0	0	0	0	1	0	0	0	0	2	0	1	1	0
1297	2024	10	M	14	0	0	0	0	1	0	0	0	0	0	1	1	0	0	0	2	0	1	1	1
1298	2024	10	F	14	0	0	0	0	1	0	0	0	0	0	1	0	1	0	0	0	0	0	0	0
1299	2021	9	M	14	0	0	0	0	1	0	0	0	0	1	1	0	0	1	1	0	0	0	0	0
1300	2020	9	M	14	0	1	1	0	0	0	0	0	0	0	1	0	0	0	0	2	1	1	1	1
1301	2020	9	M	14	0	1	1	0	0	0	0	0	0	0	1	0	0	0	0	1	0	1	1	1
1302	2021	10	M	14	0	0	1	0	1	0	0	0	0	0	1	1	0	0	0	1	0	1	1	0
1303	2022	3	F	14	0	1	1	0	0	0	0	0	0	0	1	1	0	0	0	0	0	0	0	0
1304	2022	4	F	14	1	0	1	0	0	0	0	0	0	0	1	0	1	0	0	0	0	0	0	0
1305	2022	5	F	14	0	0	1	0	0	1	0	0	0	0	1	1	0	0	0	1	1	1	0	1
1306	2024	2	F	14	0	0	1	0	1	0	0	0	0	0	1	0	0	0	0	2	0	1	1	0
1307	2022	2	F	14	0	0	0	0	1	0	1	0	0	0	1	0	0	0	0	0	0	0	0	0
1308	2022	4	F	14	0	0	0	0	1	0	1	0	0	0	1	1	0	0	0	0	0	0	0	0
1309	2024	5	M	14	0	1	0	0	1	0	0	0	0	0	1	1	0	0	0	0	0	0	0	0
1310	2021	10	M	14	0	1	0	0	1	0	0	0	0	1	0	1	0	0	0	2	1	1	1	1
1311	2020	9	M	14	0	1	0	0	1	1	0	0	0	0	1	0	1	0	0	0	0	0	0	0
1312	2022	3	F	14	1	1	1	0	0	0	0	0	0	0	1	0	1	0	0	2	1	1	0	1
1313	2022	5	M	14	1	1	1	0	0	0	0	0	0	0	1	1	0	0	0	2	1	1	1	1
1314	2023	2	M	14	1	0	1	0	0	0	1	0	0	0	1	0	1	0	0	2	0	1	0	0
1315	2021	9	F	14	0	1	1	1	0	0	0	0	0	1	1	0	0	0	0	2	0	0	0	0
1316	2020	9	M	14	0	1	1	0	0	1	1	0	0	0	1	0	0	0	0	1	0	0	0	0
1317	2020	11	F	14	1	1	1	0	1	0	0	0	0	0	1	1	0	0	0	0	0	0	0	0
1318	2023	11	F	14	1	1	1	0	1	0	0	0	0	0	1	1	1	0	0	0	0	0	0	0
1319	2021	2	M	14	1	1	1	0	0	0	1	0	1	0	1	0	0	0	0	0	1	1	1	1
1320	2023	3	F	14	1	1	1	1	1	1	1	0	0	0	1	0	1	0	0	0	0	0	0	0
1321	2020	9	F	15	0	0	0	0	0	0	0	0	0	0	0	0	0	0	1	0	0	0	0	0
1322	2020	10	F	15	0	0	0	0	0	0	0	0	0	0	1	0	1	0	0	2	1	0	1	1
1323	2020	10	M	15	0	0	0	0	0	0	0	0	0	0	0	0	0	0	1	0	0	0	0	0

ID	Yr	Mo	Sex	Age	Tre	Grs	Rag	Mug	HDM	Dan	Mld	Food	Drg	Ven	Rhi	Cnj	Ast	AsE	Urt	Sev	Prt	Obs	Rho	Snz
1324	2020	12	M	15	0	0	0	0	0	0	0	0	0	0	0	0	0	0	0	0	0	0	0	0
1325	2021	3	M	15	0	0	0	0	0	0	0	0	0	0	0	0	0	0	0	2	0	1	0	1
1326	2021	5	M	15	0	0	0	0	0	0	0	0	0	0	1	1	0	0	0	0	0	0	0	0
1327	2021	9	F	15	0	0	0	0	0	0	0	0	0	0	0	0	0	1	0	1	1	0	0	1
1328	2021	9	F	15	0	0	0	0	0	0	0	0	0	0	1	0	0	0	1	1	1	1	1	1
1329	2022	1	F	15	0	0	0	0	0	0	0	0	0	0	0	0	0	0	1	2	1	1	0	0
1330	2023	2	M	15	0	0	0	0	0	0	0	0	0	0	1	0	1	0	0	0	0	0	0	0
1331	2022	2	F	15	0	0	0	0	0	0	0	0	0	0	0	0	0	0	0	1	1	0	1	0
1332	2022	6	M	15	0	0	0	0	0	0	0	0	0	0	0	0	0	0	1	2	1	0	1	1
1333	2022	7	F	15	0	0	0	0	0	0	0	0	0	0	1	0	0	0	0	1	0	0	0	0
1334	2023	2	F	15	0	0	0	0	0	0	0	0	0	0	0	0	0	0	1	0	2	0	1	1
1335	2023	5	F	15	0	0	0	0	0	0	0	0	0	0	0	0	0	0	0	0	0	0	0	0
1336	2023	8	F	15	0	0	0	0	0	0	0	0	0	0	1	0	0	0	1	2	0	0	0	0
1337	2023	9	F	15	0	0	0	0	0	0	0	0	0	0	1	0	1	0	0	2	0	1	1	0
1338	2024	1	F	15	0	0	0	0	0	0	0	0	0	0	0	0	0	0	0	1	2	0	1	1
1339	2024	5	M	15	0	0	0	0	0	0	0	0	0	0	0	0	0	0	0	0	0	0	0	0
1340	2024	8	M	15	0	0	0	0	0	0	0	0	0	0	0	0	0	0	0	1	0	0	0	0
1341	2020	9	M	15	0	0	0	0	0	0	0	1	0	0	1	0	1	0	0	2	0	0	0	0
1342	2024	8	F	15	0	0	0	0	0	0	0	0	1	0	0	0	0	0	0	2	0	1	1	1
1343	2020	7	M	15	0	0	0	0	1	0	0	0	0	0	1	0	1	0	0	0	0	0	0	0
1344	2020	7	M	15	0	1	0	0	0	0	0	0	0	0	1	1	0	0	1	1	0	1	0	0
1345	2020	8	M	15	0	0	0	0	1	0	0	0	0	0	1	0	1	0	0	0	0	1	0	0
1346	2020	9	M	15	0	0	0	0	1	0	0	0	0	0	1	0	1	0	0	2	0	1	0	0
1347	2023	5	F	15	0	0	1	0	0	0	0	0	0	0	1	0	0	0	0	0	0	0	0	0
1348	2022	3	M	15	0	0	0	0	1	0	0	0	0	0	1	0	1	0	0	0	2	1	1	0
1349	2022	6	M	15	0	1	0	0	0	0	0	0	0	0	1	1	0	0	0	1	0	0	0	0
1350	2023	2	M	15	0	0	0	0	1	0	0	0	0	0	0	0	0	0	0	2	1	1	1	1
1351	2023	9	F	15	0	1	0	0	0	0	0	0	0	0	1	0	0	0	0	0	0	0	0	1
1352	2023	11	M	15	0	0	0	0	1	0	0	0	0	0	1	0	1	0	0	0	0	0	0	0
1353	2024	6	M	15	0	1	0	0	0	0	0	0	0	0	1	0	0	0	0	0	0	0	0	0
1354	2024	9	M	15	0	0	0	0	1	0	0	0	0	0	1	0	1	0	0	2	1	0	0	1
1355	2023	3	M	15	0	1	0	0	0	0	0	0	1	0	1	0	0	0	1	0	0	0	0	0
1356	2020	6	M	15	0	1	0	0	1	0	0	0	0	0	1	1	1	0	0	0	0	0	0	0
1357	2021	8	M	15	0	1	1	0	0	0	0	0	0	0	1	0	0	0	0	0	0	0	0	0
1358	2022	6	M	15	0	0	1	0	1	0	0	0	0	0	1	0	1	0	0	2	0	0	1	1
1359	2022	4	M	15	0	1	0	0	1	0	0	0	0	0	1	0	1	0	0	0	0	0	0	0
1360	2022	8	F	15	0	0	0	0	1	1	0	0	0	0	1	0	1	0	0	0	0	0	0	0
1361	2020	10	F	15	0	1	0	1	0	0	1	0	0	0	1	1	1	0	0	0	0	0	0	0
1362	2020	11	M	15	1	0	0	0	1	0	1	0	0	0	1	1	0	0	0	0	0	0	0	0
1363	2023	6	M	15	0	1	1	0	1	0	0	0	0	0	1	0	0	0	0	0	0	0	0	0
1364	2023	9	M	15	0	1	1	0	0	0	1	0	0	0	1	0	0	0	0	0	0	0	0	0
1365	2023	9	M	15	1	0	0	0	1	1	0	0	0	0	1	0	0	0	0	2	0	1	1	1
1366	2021	5	F	15	0	1	1	1	0	0	0	0	0	1	1	0	0	0	0	0	0	1	1	0
1367	2023	6	F	15	1	1	1	0	0	1	1	0	0	0	1	0	0	0	1	0	0	0	0	0
1368	2020	6	F	15	1	1	1	1	1	1	1	0	0	0	1	0	1	0	0	2	1	1	1	1
1369	2020	1	F	16	0	0	0	0	0	0	0	0	0	0	1	0	0	0	0	2	0	0	0	0
1370	2021	6	M	16	0	0	0	0	0	0	0	0	0	0	1	1	0	0	1	0	0	0	0	0
1371	2021	7	F	16	0	0	0	0	0	0	0	0	0	0	0	0	0	0	1	0	0	0	0	0
1372	2021	7	M	16	0	0	0	0	0	0	0	0	0	0	1	0	0	0	0	1	0	0	0	0
1373	2021	11	F	16	0	0	0	0	0	0	0	0	0	0	1	0	0	0	0	0	0	0	0	0
1374	2022	2	F	16	0	0	0	0	0	0	0	0	0	0	0	0	0	0	0	1	1	0	1	1
1375	2022	2	F	16	0	0	0	0	0	0	0	0	0	0	0	0	0	0	0	1	0	0	0	0
1376	2022	4	F	16	0	0	0	0	0	0	0	0	0	0	0	0	1	0	1	2	0	1	0	0
1377	2022	9	F	16	0	0	0	0	0	0	0	0	0	0	0	0	1	0	0	0	0	0	0	0
1378	2022	11	F	16	0	0	0	0	0	0	0	0	0	0	0	0	0	0	0	0	0	0	0	0
1379	2022	11	F	16	0	0	0	0	0	0	0	0	0	0	0	0	0	0	0	2	0	1	1	1
1380	2023	1	F	16	0	0	0	0	0	0	0	0	0	0	0	0	0	0	1	0	0	0	0	0
1381	2023	2	F	16	0	0	0	0	0	0	0	0	0	0	0	0	0	0	1	1	0	0	0	0
1382	2023	5	F	16	0	0	0	0	0	0	0	0	0	0	0	0	0	0	1	0	0	0	0	0
1383	2023	8	F	16	0	0	0	0	0	0	0	0	0	0	0	0	0	0	1	2	1	1	1	1
1384	2024	2	M	16	0	0	0	0	0	0	0	0	0	0	1	0	0	0	0	0	0	0	0	0
1385	2024	7	M	16	0	0	0	0	0	0	0	0	0	0	0	0	0	0	0	2	0	0	1	1
1386	2024	9	F	16	0	0	0	0	0	0	0	0	0	0	0	0	1	0	0	0	0	0	0	0

ID	Yr	Mo	Sex	Age	Tre	Grs	Rag	Mug	HDM	Dan	Mld	Food	Drg	Ven	Rhi	Cnj	Ast	AsE	Urt	Sev	Prt	Obs	Rho	Snz
1387	2020	9	M	16	0	0	0	0	0	0	0	0	0	1	1	1	0	0	0	1	0	1	0	0
1388	2021	9	F	16	0	0	0	0	0	0	0	0	0	1	0	0	0	1	0	0	0	0	0	0
1389	2024	11	M	16	0	0	0	0	0	0	0	0	1	0	0	0	0	0	0	0	0	0	0	0
1390	2020	2	F	16	0	0	0	0	1	0	0	0	0	0	1	0	1	0	0	0	0	0	0	0
1391	2020	7	M	16	0	1	0	0	0	0	0	0	0	0	1	0	0	0	0	0	0	0	0	0
1392	2021	1	M	16	0	0	0	0	0	0	1	0	0	0	1	0	0	0	0	0	0	0	0	0
1393	2021	9	F	16	0	0	0	0	1	0	0	0	0	0	1	0	0	0	1	2	1	1	1	0
1394	2021	1	F	16	0	0	0	0	1	0	0	0	0	0	1	0	0	0	0	0	0	0	0	0
1395	2021	11	F	16	0	0	1	0	0	0	0	0	0	0	1	0	1	0	0	0	0	1	0	0
1396	2023	2	M	16	0	0	1	0	0	0	0	0	0	0	1	1	0	0	0	0	0	0	0	0
1397	2023	10	M	16	0	0	1	0	0	0	0	0	0	0	1	1	0	0	0	0	0	0	0	0
1398	2024	9	M	16	0	0	1	0	0	0	0	0	0	0	1	1	0	0	0	0	0	1	0	0
1399	2022	5	F	16	0	0	0	0	1	0	0	0	0	0	0	0	0	0	0	0	0	0	0	0
1400	2023	4	F	16	0	0	0	0	1	0	0	0	0	0	1	0	0	0	0	2	1	1	1	1
1401	2023	7	M	16	0	1	0	0	0	0	0	0	0	0	1	0	0	0	0	0	0	0	0	0
1402	2023	8	M	16	0	1	0	0	0	0	0	0	0	0	1	0	0	0	0	0	0	0	0	0
1403	2023	11	M	16	0	0	0	0	1	0	0	0	0	0	1	0	1	0	0	2	1	1	1	1
1404	2024	6	F	16	0	0	0	0	1	0	0	0	0	0	1	1	0	0	0	1	0	0	0	0
1405	2021	10	M	16	0	0	0	0	1	0	0	0	0	1	0	0	0	0	0	0	1	0	1	0
1406	2021	9	M	16	0	0	1	0	0	0	0	0	0	1	0	0	0	1	0	0	0	0	0	0
1407	2020	12	M	16	0	0	0	0	1	0	1	0	0	0	1	1	1	1	0	1	0	1	0	0
1408	2020	10	M	16	0	0	1	0	1	0	0	0	0	0	1	1	1	0	0	1	3	1	1	1
1409	2022	7	F	16	0	1	1	0	0	0	0	0	0	0	1	0	0	0	0	1	0	0	0	0
1410	2023	9	M	16	0	1	1	0	0	0	0	0	0	0	1	1	0	0	0	0	0	0	0	0
1411	2022	12	M	16	0	0	0	0	1	1	0	0	0	0	1	0	1	1	0	0	1	0	1	0
1412	2023	3	F	16	0	1	0	0	1	0	0	0	0	0	1	0	0	0	1	0	1	0	1	0
1413	2024	7	F	16	0	1	0	0	0	1	0	0	0	0	1	1	0	0	0	2	1	1	1	1
1414	2024	8	F	16	0	0	0	0	1	0	1	0	0	0	1	0	0	0	0	2	0	1	1	1
1415	2021	6	M	16	1	1	0	1	0	0	0	0	0	0	1	1	0	0	0	0	0	0	0	0
1416	2020	10	M	16	1	0	1	0	0	1	0	0	0	0	1	1	1	0	0	0	0	0	0	0
1417	2022	11	M	16	0	1	1	0	0	0	1	0	0	0	1	0	1	0	0	2	1	0	1	1
1418	2022	11	M	16	0	1	1	0	0	0	1	0	0	0	1	0	1	0	0	0	0	0	0	0
1419	2023	11	M	16	0	1	1	0	1	0	0	0	0	0	1	0	0	0	0	0	0	0	0	0
1420	2022	1	F	16	0	1	0	0	1	0	1	0	0	0	1	0	0	0	0	2	0	1	1	1
1421	2022	7	F	16	0	1	0	0	1	1	0	0	0	0	1	1	1	0	0	2	1	1	0	0
1422	2022	11	F	16	0	1	1	1	0	0	1	0	0	0	1	1	0	0	0	1	0	0	0	0
1423	2021	5	F	16	0	1	0	0	1	1	1	0	0	1	1	1	0	0	0	2	0	0	1	1
1424	2020	7	M	16	1	1	1	1	0	0	0	1	0	0	1	1	0	0	0	0	0	0	0	0
1425	2020	1	F	16	1	1	1	1	1	0	0	0	0	0	1	1	0	0	1	0	0	0	0	0
1426	2023	1	F	16	1	1	1	1	1	1	0	0	0	1	1	1	0	0	0	0	0	0	0	0
1427	2020	9	M	17	0	0	0	0	0	0	0	0	0	0	1	0	1	0	0	0	3	1	1	1
1428	2020	10	M	17	0	0	0	0	0	0	0	0	0	0	0	0	0	0	0	0	0	0	0	0
1429	2021	2	F	17	0	0	0	0	0	0	0	0	0	0	1	0	0	0	1	2	1	1	1	1
1430	2022	3	F	17	0	0	0	0	0	0	0	0	0	0	0	0	0	0	1	1	0	1	1	1
1431	2022	4	F	17	0	0	0	0	0	0	0	0	0	0	1	0	0	0	1	0	0	1	0	0
1432	2022	6	F	17	0	0	0	0	0	0	0	0	0	0	1	0	0	0	1	2	0	1	0	1
1433	2022	7	F	17	0	0	0	0	0	0	0	0	0	0	0	0	0	0	1	2	1	0	1	1
1434	2022	8	F	17	0	0	0	0	0	0	0	0	0	0	0	0	0	0	1	2	1	1	1	1
1435	2022	9	F	17	0	0	0	0	0	0	0	0	0	0	0	0	0	0	1	2	0	0	0	0
1436	2022	11	F	17	0	0	0	0	0	0	0	0	0	0	1	0	0	0	0	2	1	1	1	1
1437	2022	11	F	17	0	0	0	0	0	0	0	0	0	0	1	0	0	0	0	0	2	0	1	1
1438	2023	8	F	17	0	0	0	0	0	0	0	0	0	0	0	0	0	0	1	1	0	0	0	0
1439	2023	9	F	17	0	0	0	0	0	0	0	0	0	0	0	0	0	0	0	0	1	1	1	1
1440	2023	11	F	17	0	0	0	0	0	0	0	0	0	0	1	0	0	0	0	2	1	0	1	1
1441	2024	5	F	17	0	0	0	0	0	0	0	0	0	0	0	0	0	0	1	0	0	0	0	0
1442	2020	9	F	17	0	0	0	0	1	0	0	0	0	0	1	0	1	0	0	0	0	0	0	0
1443	2020	9	F	17	0	0	0	0	1	0	0	0	0	0	1	0	0	0	0	2	1	1	1	1
1444	2020	10	M	17	0	0	0	0	0	0	1	0	0	0	1	1	1	0	0	1	0	0	0	0
1445	2020	12	F	17	0	0	0	0	0	1	0	0	0	0	0	0	0	0	0	0	0	0	0	0
1446	2022	9	F	17	0	0	1	0	0	0	0	0	0	0	1	1	0	0	0	2	1	1	1	1
1447	2022	9	F	17	0	0	1	0	0	0	0	0	0	0	1	1	0	0	0	0	0	0	0	0
1448	2024	9	F	17	0	0	1	0	0	0	0	0	0	0	1	1	0	0	0	1	0	0	0	0
1449	2022	5	M	17	1	0	0	0	0	0	0	0	0	0	1	0	0	0	0	2	0	1	1	1

ID	Yr	Mo	Sex	Age	Tre	Grs	Rag	Mug	HDM	Dan	Mld	Food	Drg	Ven	Rhi	Cnj	Ast	AsE	Urt	Sev	Prt	Obs	Rho	Snz
1450	2023	1	F	17	0	0	0	0	1	0	0	0	0	0	1	0	0	0	0	0	0	0	0	0
1451	2023	3	M	17	0	0	0	0	1	0	0	0	0	0	1	0	0	0	0	1	0	1	0	0
1452	2023	5	M	17	0	0	0	0	1	0	0	0	0	0	1	1	0	0	0	2	0	1	0	0
1453	2023	11	M	17	0	0	0	0	1	0	0	0	0	0	1	0	0	0	0	1	0	0	0	0
1454	2024	10	M	17	0	0	0	0	0	1	0	0	0	0	1	0	1	0	0	1	2	0	1	1
1455	2020	12	M	17	0	0	1	0	1	0	0	0	0	0	1	1	0	0	1	2	0	1	1	1
1456	2022	9	F	17	0	0	1	0	0	1	0	0	0	0	1	0	0	0	0	2	0	1	1	1
1457	2022	10	F	17	0	0	1	0	0	1	0	0	0	0	0	0	1	0	1	0	0	0	0	0
1458	2022	6	F	17	0	1	0	0	1	0	0	0	0	0	1	1	0	0	0	1	0	0	0	0
1459	2023	6	M	17	0	1	0	0	1	0	0	0	0	0	1	0	0	0	1	0	0	0	0	0
1460	2023	7	M	17	0	0	0	0	1	1	0	0	0	0	1	0	0	0	0	2	0	1	1	1
1461	2024	3	M	17	0	0	0	0	0	1	1	0	0	0	1	0	0	0	0	2	1	1	0	1
1462	2024	5	M	17	0	1	0	0	0	1	0	0	0	0	1	1	1	0	0	2	0	1	1	1
1463	2022	2	F	17	0	1	1	0	0	0	0	1	0	0	1	0	0	0	0	2	1	1	0	1
1464	2024	1	F	17	0	0	1	0	0	1	0	1	0	0	1	1	0	0	0	0	0	1	0	0
1465	2022	4	F	17	0	1	1	0	0	1	0	0	0	0	1	1	0	0	0	1	0	1	0	0
1466	2022	6	F	17	1	0	1	0	0	1	0	0	0	0	1	1	0	0	0	0	0	0	0	0
1467	2023	9	F	17	0	1	1	0	1	0	0	0	0	0	1	0	0	0	0	0	0	0	0	0
1468	2024	6	F	17	1	0	1	0	0	0	1	0	0	0	1	1	0	0	0	2	1	0	0	1
1469	2022	3	F	17	0	1	0	0	1	1	1	0	0	0	1	0	1	0	1	2	0	1	0	1
1470	2022	12	M	17	1	1	0	0	1	1	0	0	0	0	1	0	0	0	0	0	0	0	0	0
1471	2020	8	M	17	1	1	1	0	0	1	1	0	0	0	1	1	0	0	0	1	0	0	0	0
1472	2022	12	M	17	1	1	1	0	0	1	1	0	0	0	1	1	0	0	1	2	0	1	0	1
1473	2024	7	M	17	1	1	1	0	1	1	0	0	0	0	1	1	0	0	0	2	1	1	1	1
1474	2024	8	M	17	0	1	1	1	1	0	1	0	0	0	1	1	0	0	0	1	0	0	0	0
1475	2024	10	M	17	1	1	1	1	1	0	0	0	0	0	1	1	0	0	0	0	0	1	0	0
1476	2021	8	F	17	1	1	1	1	1	1	1	0	0	0	1	1	0	0	0	1	0	0	0	0
1477	2020	1	M	18	0	0	0	0	0	0	0	0	0	0	0	0	0	0	0	2	1	1	1	1
1478	2020	2	M	18	0	0	0	0	0	0	0	0	0	0	0	0	0	0	0	0	0	0	0	0
1479	2020	2	F	18	0	0	0	0	0	0	0	0	0	0	1	0	0	0	0	2	1	0	1	1
1480	2020	3	F	18	0	0	0	0	0	0	0	0	0	0	0	0	1	0	0	2	1	1	1	1
1481	2020	3	M	18	0	0	0	0	0	0	0	0	0	0	0	0	0	0	0	2	0	1	0	1
1482	2020	7	M	18	0	0	0	0	0	0	0	0	0	0	0	0	0	0	0	0	1	1	1	1
1483	2020	10	F	18	0	0	0	0	0	0	0	0	0	0	0	0	0	0	1	0	0	0	0	0
1484	2020	10	M	18	0	0	0	0	0	0	0	0	0	0	1	0	0	0	0	0	0	0	0	0
1485	2020	12	M	18	0	0	0	0	0	0	0	0	0	0	1	0	1	0	0	0	0	0	0	0
1486	2021	8	M	18	0	0	0	0	0	0	0	0	0	0	1	1	0	0	0	2	1	0	0	1
1487	2021	10	F	18	0	0	0	0	0	0	0	0	0	0	0	0	0	0	0	2	1	1	1	1
1488	2022	1	M	18	0	0	0	0	0	0	0	0	0	0	0	0	0	0	0	2	1	0	1	1
1489	2022	5	M	18	0	0	0	0	0	0	0	0	0	0	0	0	0	0	1	0	0	0	0	0
1490	2022	10	F	18	0	0	0	0	0	0	0	0	0	0	0	0	0	0	1	0	0	1	0	1
1491	2022	11	M	18	0	0	0	0	0	0	0	0	0	0	1	0	0	0	0	0	0	0	0	0
1492	2023	2	M	18	0	0	0	0	0	0	0	0	0	0	0	0	0	0	0	2	0	1	1	1
1493	2023	8	F	18	0	0	0	0	0	0	0	0	0	0	0	0	0	0	1	0	0	0	0	0
1494	2024	3	M	18	0	0	0	0	0	0	0	0	0	0	0	0	1	0	0	2	0	0	1	1
1495	2024	8	M	18	0	0	0	0	0	0	0	0	0	0	0	0	0	0	0	1	0	0	1	1
1496	2024	8	F	18	0	0	0	0	0	0	0	0	0	0	0	0	0	0	1	2	0	1	1	0
1497	2024	12	F	18	0	0	0	0	0	0	0	0	0	0	0	0	0	0	1	0	0	0	0	0
1498	2022	1	F	18	0	0	1	0	0	0	0	0	0	0	1	1	1	0	0	0	0	0	0	0
1499	2023	9	F	18	0	0	1	0	0	0	0	0	0	0	1	1	1	1	0	0	0	0	0	0
1500	2023	10	F	18	0	0	1	0	0	0	0	0	0	0	1	1	0	0	0	2	1	1	1	1
1501	2022	9	M	18	0	0	0	0	1	0	0	0	0	0	1	0	0	0	0	0	0	1	0	1
1502	2024	7	M	18	0	1	0	0	0	0	0	0	0	0	1	1	0	0	0	0	2	0	1	1
1503	2024	11	M	18	0	0	0	0	1	0	0	0	0	0	1	1	1	0	0	0	1	0	1	0
1504	2021	5	F	18	0	0	0	0	0	0	1	0	0	1	0	0	1	0	0	2	0	0	1	1
1505	2022	2	M	18	0	0	1	1	0	0	0	0	0	0	1	1	0	0	0	0	0	0	0	0
1506	2022	11	M	18	1	0	0	0	1	0	0	0	0	0	1	0	1	0	1	0	0	0	0	0
1507	2021	9	M	18	0	1	1	0	0	0	0	1	0	0	1	1	0	0	0	2	1	1	0	1
1508	2022	3	F	18	1	0	0	0	0	0	1	1	0	0	1	1	0	0	0	1	0	1	0	0
1509	2020	7	F	18	0	1	0	1	0	1	0	0	0	0	1	1	0	0	0	2	0	0	1	1
1510	2020	9	M	18	0	1	0	0	0	1	1	0	0	0	1	1	0	0	0	0	2	2	1	1
1511	2020	8	F	18	1	1	1	0	0	0	0	0	0	0	1	1	0	0	0	0	0	0	0	0
1512	2023	10	F	18	0	1	1	0	1	1	0	0	0	0	1	0	0	0	0	0	0	0	0	0

ID	Yr	Mo	Sex	Age	Tre	Grs	Rag	Mug	HDM	Dan	Mld	Food	Drg	Ven	Rhi	Cnj	Ast	AsE	Urt	Sev	Prt	Obs	Rho	Snz
1513	2022	1	F	18	0	1	1	0	1	1	1	0	0	0	1	0	1	0	0	2	1	1	1	0
1514	2024	5	M	18	1	1	1	1	0	1	0	0	0	0	1	1	0	0	0	0	0	0	0	0
1515	2020	5	F	18	1	1	1	0	1	1	0	0	0	0	1	1	0	0	1	0	0	0	0	0
1516	2020	2	F	19	0	0	0	0	0	0	0	0	0	0	0	0	0	0	1	2	1	1	1	1
1517	2020	8	F	19	0	0	0	0	0	0	0	0	0	0	0	0	0	0	0	2	0	1	1	1
1518	2020	11	F	19	0	0	0	0	0	0	0	0	0	0	1	0	0	0	0	0	0	1	0	1
1519	2021	12	F	19	0	0	0	0	0	0	0	0	0	0	1	0	1	0	0	2	1	1	1	1
1520	2021	12	F	19	0	0	0	0	0	0	0	0	0	0	0	0	0	1	0	0	0	1	1	1
1521	2022	4	M	19	0	0	0	0	0	0	0	0	0	0	0	0	0	0	1	0	0	1	1	0
1522	2022	7	F	19	0	0	0	0	0	0	0	0	0	0	0	0	0	0	1	0	0	0	0	0
1523	2022	12	M	19	0	0	0	0	0	0	0	0	0	0	1	0	0	0	0	2	0	1	0	0
1524	2022	12	M	19	0	0	0	0	0	0	0	0	0	0	0	0	0	0	0	0	0	0	0	0
1525	2022	12	M	19	0	0	0	0	0	0	0	0	0	0	0	0	0	0	0	2	0	1	1	0
1526	2022	12	M	19	0	0	0	0	0	0	0	0	0	0	1	0	0	0	0	0	0	0	0	0
1527	2023	4	F	19	0	0	0	0	0	0	0	0	0	0	0	0	0	0	0	0	0	0	0	0
1528	2023	5	M	19	0	0	0	0	0	0	0	0	0	0	1	0	0	0	0	2	1	1	1	1
1529	2023	6	F	19	0	0	0	0	0	0	0	0	0	0	0	0	0	0	1	1	0	0	1	1
1530	2023	8	F	19	0	0	0	0	0	0	0	0	0	0	1	0	1	0	0	0	0	0	0	0
1531	2023	10	F	19	0	0	0	0	0	0	0	0	0	0	1	0	0	0	0	0	0	0	0	0
1532	2024	9	M	19	0	0	0	0	0	0	0	0	0	0	0	0	1	0	0	2	1	1	1	1
1533	2024	12	M	19	0	0	0	0	0	0	0	0	0	0	0	0	0	0	0	0	0	0	0	0
1534	2021	9	M	19	0	0	0	0	0	0	1	0	0	0	1	0	1	0	0	2	0	1	1	1
1535	2021	12	F	19	0	0	0	0	1	0	0	0	0	0	1	0	1	0	0	0	0	0	0	0
1536	2020	8	M	19	0	0	1	0	0	0	0	0	0	0	1	0	0	0	0	2	1	0	1	1
1537	2022	11	M	19	0	0	1	0	0	0	0	0	0	0	1	1	0	0	0	0	1	0	0	1
1538	2023	9	F	19	0	0	1	0	0	0	0	0	0	0	1	0	1	0	0	2	1	1	0	0
1539	2022	9	F	19	0	0	0	0	1	0	0	0	0	0	1	0	0	0	1	0	0	0	0	0
1540	2023	10	M	19	0	1	0	0	0	0	0	0	0	0	1	0	0	0	0	2	1	1	1	1
1541	2024	4	M	19	0	0	0	0	1	0	0	0	0	0	0	1	0	0	1	2	0	1	1	1
1542	2021	4	M	19	0	0	1	0	0	0	0	0	0	1	0	1	0	0	0	2	0	1	1	1
1543	2022	4	F	19	0	0	0	0	0	0	1	1	0	0	1	1	0	0	0	2	0	1	1	1
1544	2020	6	M	19	0	1	0	0	1	0	0	0	0	0	1	0	1	0	0	2	0	1	0	0
1545	2021	10	F	19	0	1	0	1	0	0	0	0	0	0	1	1	1	0	0	0	0	0	0	0
1546	2020	5	M	19	0	1	0	0	1	0	0	0	0	0	1	1	0	0	1	2	1	1	1	1
1547	2020	7	M	19	0	0	1	1	0	0	0	0	0	0	1	1	0	0	0	1	0	0	1	1
1548	2022	10	F	19	0	0	1	0	1	0	0	0	0	0	1	0	1	0	0	0	0	0	0	0
1549	2022	11	F	19	0	1	1	0	0	0	0	0	0	0	1	1	0	0	0	0	0	0	0	0
1550	2022	1	F	19	0	0	0	0	1	1	0	0	0	0	1	0	1	0	0	0	0	1	0	0
1551	2024	11	F	19	1	0	0	0	0	1	0	0	0	0	1	0	0	0	0	0	0	0	0	0
1552	2020	3	F	19	1	1	0	0	0	0	0	0	0	1	1	1	0	0	0	0	1	0	1	0
1553	2021	12	F	19	0	1	0	0	0	0	1	0	0	1	0	1	0	0	0	0	0	0	0	0
1554	2020	6	F	19	1	1	0	0	0	0	1	0	0	0	1	0	0	0	0	2	1	1	1	1
1555	2020	6	F	19	0	0	1	0	1	1	0	0	0	0	1	1	0	0	0	0	0	0	0	0
1556	2023	2	F	19	1	0	0	0	1	0	1	0	0	0	1	0	0	0	0	0	0	0	0	0
1557	2020	10	F	20	0	0	0	0	0	0	0	0	0	0	1	0	1	0	0	0	0	0	0	0
1558	2020	11	F	20	0	0	0	0	0	0	0	0	0	0	0	0	0	0	1	0	0	0	0	0
1559	2021	1	F	20	0	0	0	0	0	0	0	0	0	0	0	0	0	0	0	0	0	0	0	0
1560	2021	6	F	20	0	0	0	0	0	0	0	0	0	0	0	0	0	0	0	1	0	0	1	1
1561	2023	2	F	20	0	0	0	0	0	0	0	0	0	0	1	0	0	0	0	1	1	1	1	1
1562	2023	3	F	20	0	0	0	0	0	0	0	0	0	0	1	0	1	0	0	2	1	1	1	1
1563	2023	9	F	20	0	0	0	0	0	0	0	0	0	0	0	0	0	0	1	0	0	0	0	0
1564	2024	5	M	20	0	0	0	0	0	0	0	0	0	0	0	0	0	0	1	2	1	1	0	0
1565	2024	10	F	20	0	0	0	0	0	0	0	0	0	0	0	0	0	0	1	2	1	0	1	1
1566	2024	10	F	20	0	0	0	0	0	0	0	0	0	0	0	0	0	0	0	1	1	1	1	1
1567	2024	11	F	20	0	0	0	0	0	0	0	0	0	0	0	0	0	0	1	2	0	0	1	1
1568	2024	12	F	20	0	0	0	0	0	0	0	0	0	0	0	0	0	0	0	0	0	0	0	0
1569	2021	2	F	20	0	0	0	0	0	0	0	0	1	0	0	0	0	0	0	0	0	0	0	0
1570	2022	9	F	20	0	0	0	0	0	0	0	0	0	1	0	0	0	0	1	2	1	1	1	1
1571	2020	9	F	20	1	0	0	0	0	0	0	0	0	0	1	1	0	0	0	2	1	1	1	1
1572	2022	3	M	20	0	0	1	0	0	0	0	0	0	0	1	0	0	0	0	2	0	1	1	1
1573	2022	5	F	20	1	0	0	0	0	0	0	0	0	0	1	0	0	0	1	2	0	1	1	1
1574	2023	3	M	20	0	0	0	0	1	0	0	0	0	0	1	0	0	0	0	0	0	1	0	1
1575	2023	10	F	20	0	1	0	0	0	0	0	0	0	0	1	0	0	0	0	0	0	0	0	0

ID	Yr	Mo	Sex	Age	Tre	Grs	Rag	Mug	HDM	Dan	Mld	Food	Drg	Ven	Rhi	Cnj	Ast	AsE	Urt	Sev	Prt	Obs	Rho	Snz
1576	2022	7	M	20	0	1	0	0	1	0	0	0	0	0	1	1	0	0	0	0	0	0	0	0
1577	2024	1	F	20	0	1	0	0	1	0	0	0	0	0	1	1	0	0	0	2	0	0	1	1
1578	2024	5	M	20	1	1	0	0	0	0	0	0	0	0	1	1	0	0	0	2	1	1	1	1
1579	2024	6	F	20	0	0	1	1	0	0	0	1	0	0	1	1	0	0	1	2	0	0	1	1
1580	2023	2	M	20	0	1	1	1	0	0	0	0	0	0	1	0	0	0	0	0	0	0	1	1
1581	2023	6	M	20	0	1	1	0	0	0	1	0	0	0	1	0	1	0	0	0	0	0	0	0
1582	2023	7	F	20	0	1	0	0	1	1	0	0	0	0	1	0	1	0	0	0	0	0	1	1
1583	2024	2	F	20	1	1	0	1	0	0	0	0	0	0	1	1	0	0	0	0	0	0	1	1
1584	2024	7	F	20	1	0	0	0	1	1	0	0	0	0	1	1	1	0	0	0	0	1	0	0
1585	2023	4	F	20	1	1	1	0	1	0	0	0	0	0	1	0	1	0	0	0	0	0	0	0
1586	2023	11	F	20	1	1	1	1	0	0	0	0	0	0	1	1	0	0	0	2	1	1	1	1
1587	2024	5	F	20	1	0	1	0	1	1	0	0	0	0	1	1	0	0	0	0	0	1	1	0
1588	2024	8	F	20	1	1	1	0	1	0	0	0	0	0	1	1	1	0	0	2	1	1	0	0
1589	2021	11	M	20	1	1	1	0	0	1	1	0	0	0	1	0	1	0	0	0	0	0	0	0
1590	2021	4	F	20	0	1	1	1	1	1	0	1	0	0	1	0	0	0	1	0	0	0	0	0
1591	2023	2	F	20	1	1	1	1	1	1	1	0	0	0	1	1	0	0	0	0	0	0	0	0
1592	2020	3	F	21	0	0	0	0	0	0	0	0	0	0	0	0	0	0	0	2	1	0	1	1
1593	2020	5	F	21	0	0	0	0	0	0	0	0	0	0	1	0	0	0	0	0	0	0	0	0
1594	2021	2	F	21	0	0	0	0	0	0	0	0	0	0	0	0	1	0	0	NA	0	0	0	0
1595	2021	9	F	21	0	0	0	0	0	0	0	0	0	0	1	0	1	0	0	NA	0	0	0	0
1596	2021	10	M	21	0	0	0	0	0	0	0	0	0	0	0	0	1	0	0	2	0	1	1	0
1597	2021	9	M	21	0	0	0	0	0	0	0	0	0	0	0	0	0	0	0	NA	0	0	0	0
1598	2021	3	F	21	0	0	0	0	0	0	0	0	0	0	1	1	0	1	1	2	1	1	1	1
1599	2021	11	F	21	0	0	0	0	0	0	0	0	0	0	0	0	0	0	0	NA	0	0	0	0
1600	2022	4	F	21	0	0	0	0	0	0	0	0	0	0	0	0	0	0	0	NA	0	0	0	0
1601	2022	5	M	21	0	0	0	0	0	0	0	0	0	0	0	0	0	0	0	2	1	1	1	1
1602	2022	6	F	21	0	0	0	0	0	0	0	0	0	0	0	0	0	0	0	NA	0	0	0	0
1603	2022	9	M	21	0	0	0	0	0	0	0	0	0	0	1	0	1	0	0	NA	0	0	0	0
1604	2022	10	F	21	0	0	0	0	0	0	0	0	0	0	1	0	1	0	0	NA	0	0	0	0
1605	2022	11	F	21	0	0	0	0	0	0	0	0	0	0	0	0	0	0	1	2	1	0	1	1
1606	2023	2	F	21	0	0	0	0	0	0	0	0	0	0	0	0	0	0	1	2	0	1	1	0
1607	2024	1	F	21	0	0	0	0	0	0	0	0	0	0	0	0	0	0	1	NA	0	0	0	0
1608	2024	10	F	21	0	0	0	0	0	0	0	0	0	0	0	0	0	0	1	NA	0	0	0	0
1609	2020	10	M	21	0	0	0	0	0	0	0	0	0	1	0	0	0	0	0	NA	0	1	0	0
1610	2021	9	F	21	0	0	0	0	0	0	0	0	0	1	0	1	0	0	0	NA	0	1	1	0
1611	2021	10	F	21	0	0	0	0	0	0	0	0	1	0	0	0	0	0	0	2	1	1	1	1
1612	2021	5	F	21	0	0	0	0	0	0	0	0	0	1	1	0	0	1	0	2	1	1	1	1
1613	2022	1	F	21	0	0	0	0	0	0	0	0	1	0	0	0	0	0	0	2	0	0	1	0
1614	2024	6	M	21	0	0	0	0	0	0	0	0	1	0	1	0	0	0	0	2	0	1	1	0
1615	2020	1	M	21	0	0	0	0	1	0	0	0	0	0	1	0	1	0	0	2	1	1	1	1
1616	2022	3	F	21	0	0	1	0	0	0	0	0	0	0	1	0	0	0	0	NA	0	0	0	0
1617	2022	9	F	21	0	0	1	0	0	0	0	0	0	0	1	1	0	0	1	2	0	1	0	1
1618	2022	12	F	21	0	0	1	0	0	0	0	0	0	0	1	0	0	0	0	NA	0	0	0	0
1619	2022	3	F	21	0	0	0	0	0	1	0	0	0	0	1	0	0	0	0	2	1	1	1	1
1620	2022	11	F	21	0	0	0	0	1	0	0	0	0	0	1	0	1	0	0	NA	0	0	0	0
1621	2020	3	M	21	1	1	0	0	0	0	0	0	0	0	1	1	0	0	0	NA	0	0	0	0
1622	2021	5	F	21	0	0	0	0	1	1	0	0	0	0	1	0	1	0	0	2	0	0	1	1
1623	2022	5	M	21	0	0	1	0	1	0	0	0	0	0	1	1	1	0	0	NA	0	0	0	0
1624	2023	9	M	21	0	1	1	0	0	0	0	0	0	0	1	0	0	0	0	NA	0	1	1	0
1625	2024	4	M	21	0	0	1	0	0	1	0	0	0	0	1	0	0	0	0	NA	0	0	0	0
1626	2022	10	M	21	0	1	0	0	1	0	0	0	0	0	1	0	1	0	0	NA	0	0	0	0
1627	2024	3	F	21	0	0	0	0	0	1	1	0	0	0	1	0	0	0	0	2	1	0	0	1
1628	2021	3	F	21	1	1	0	0	1	0	0	0	0	0	1	1	0	0	1	2	0	1	0	0
1629	2021	2	M	21	0	0	1	0	1	1	0	0	0	0	1	1	1	0	0	2	1	1	1	1
1630	2022	10	M	21	0	0	1	0	1	1	0	0	0	0	1	1	0	0	1	NA	0	0	0	0
1631	2022	3	F	21	1	1	0	0	1	0	0	0	0	0	1	1	0	0	0	2	0	1	0	1
1632	2022	10	F	21	1	1	0	0	1	0	0	0	0	0	1	1	1	0	0	NA	0	0	0	0
1633	2023	8	F	21	1	1	0	1	0	0	0	0	0	0	1	0	0	0	1	NA	0	0	0	0
1634	2024	7	F	21	0	0	0	1	0	1	1	0	0	0	1	0	0	0	0	NA	0	0	0	0
1635	2024	1	F	21	0	1	1	1	0	1	1	0	0	0	1	1	1	0	0	NA	0	0	0	0
1636	2020	1	M	22	0	0	0	0	0	0	0	0	0	0	0	0	0	0	1	NA	0	0	0	0
1637	2020	3	F	22	0	0	0	0	0	0	0	0	0	0	0	0	1	0	0	NA	0	0	0	0
1638	2020	7	F	22	0	0	0	0	0	0	0	0	0	0	0	0	0	0	1	2	1	1	1	1

ID	Yr	Mo	Sex	Age	Tre	Grs	Rag	Mug	HDM	Dan	Mld	Food	Drg	Ven	Rhi	Cnj	Ast	AsE	Urt	Sev	Prt	Obs	Rho	Snz
1639	2020	9	F	22	0	0	0	0	0	0	0	0	0	0	0	0	0	0	0	2	1	1	1	1
1640	2020	12	F	22	0	0	0	0	0	0	0	0	0	0	1	0	0	0	0	NA	0	0	0	0
1641	2021	1	F	22	0	0	0	0	0	0	0	0	0	0	0	0	0	0	0	NA	0	1	0	1
1642	2021	5	F	22	0	0	0	0	0	0	0	0	0	0	1	0	0	0	0	2	0	0	0	0
1643	2021	6	F	22	0	0	0	0	0	0	0	0	0	0	1	1	0	0	0	2	0	1	1	1
1644	2021	3	F	22	0	0	0	0	0	0	0	0	0	0	0	0	0	1	0	NA	0	0	0	0
1645	2021	3	F	22	0	0	0	0	0	0	0	0	0	0	0	1	0	1	1	2	0	1	1	1
1646	2021	1	F	22	0	0	0	0	0	0	0	0	0	0	0	0	0	0	1	2	0	0	0	0
1647	2021	10	F	22	0	0	0	0	0	0	0	0	0	0	0	0	0	1	0	2	0	0	0	0
1648	2022	6	M	22	0	0	0	0	0	0	0	0	0	0	0	0	0	0	1	2	1	1	0	0
1649	2022	8	F	22	0	0	0	0	0	0	0	0	0	0	1	1	0	0	0	NA	0	0	0	0
1650	2022	8	F	22	0	0	0	0	0	0	0	0	0	0	1	1	0	0	0	1	0	1	1	0
1651	2022	10	F	22	0	0	0	0	0	0	0	0	0	0	0	0	0	0	0	2	1	1	1	1
1652	2023	5	F	22	0	0	0	0	0	0	0	0	0	0	0	0	0	0	1	NA	0	0	0	0
1653	2023	5	F	22	0	0	0	0	0	0	0	0	0	0	0	0	0	0	1	NA	0	0	0	0
1654	2023	9	M	22	0	0	0	0	0	0	0	0	0	0	0	0	0	0	1	NA	0	0	0	0
1655	2023	11	M	22	0	0	0	0	0	0	0	0	0	0	0	0	0	0	1	2	0	1	0	0
1656	2023	11	F	22	0	0	0	0	0	0	0	0	0	0	0	0	0	0	1	2	0	1	1	0
1657	2024	5	F	22	0	0	0	0	0	0	0	0	0	0	0	0	1	0	0	NA	0	0	0	0
1658	2024	7	F	22	0	0	0	0	0	0	0	0	0	0	0	0	0	0	1	NA	0	1	0	0
1659	2021	6	F	22	0	0	0	0	0	0	0	0	1	0	0	0	0	0	0	2	1	1	0	1
1660	2022	10	F	22	0	0	0	0	0	0	0	0	1	0	0	0	0	0	1	NA	0	0	0	0
1661	2024	3	M	22	0	0	0	0	0	0	0	0	0	1	1	0	0	0	0	NA	0	0	0	0
1662	2024	4	F	22	0	0	0	0	0	0	0	0	0	1	1	1	0	0	0	2	0	0	0	0
1663	2021	2	M	22	0	0	0	0	1	0	0	0	0	0	1	0	0	0	0	2	0	1	0	0
1664	2024	8	M	22	0	0	1	0	0	0	0	0	0	0	1	1	0	0	0	2	0	1	0	0
1665	2022	4	F	22	0	0	0	0	0	0	1	0	0	0	1	0	1	0	0	2	0	0	1	1
1666	2022	10	F	22	0	1	0	0	0	0	0	0	0	0	1	1	0	0	0	2	0	1	0	0
1667	2023	3	M	22	0	0	0	0	1	0	0	0	0	0	1	0	1	0	0	NA	0	0	0	0
1668	2023	11	F	22	0	0	0	0	1	0	0	0	0	0	1	0	1	0	0	2	1	1	1	0
1669	2024	1	F	22	0	0	0	0	1	0	0	0	0	0	1	0	0	0	0	NA	0	0	0	0
1670	2021	10	M	22	0	0	0	0	1	0	0	0	0	1	1	0	0	0	0	NA	0	0	0	0
1671	2023	8	F	22	0	0	0	0	1	0	0	1	0	0	1	0	0	0	1	2	0	0	0	0
1672	2024	11	F	22	0	0	0	0	1	0	0	0	1	0	1	0	1	0	0	2	0	0	0	1
1673	2020	12	M	22	1	0	0	0	1	0	0	0	0	0	1	0	0	0	0	2	0	0	1	0
1674	2020	2	F	22	0	1	1	0	0	0	0	0	0	0	1	1	0	0	0	NA	0	0	0	0
1675	2020	9	F	22	0	0	1	1	0	0	0	0	0	0	1	1	0	0	0	NA	0	0	0	0
1676	2020	11	M	22	0	0	1	0	0	1	0	0	0	0	1	0	0	0	0	2	0	1	1	0
1677	2023	4	M	22	0	0	1	0	1	0	0	0	0	0	1	0	0	0	0	2	1	1	1	1
1678	2024	7	M	22	0	0	1	0	1	0	0	0	0	0	1	1	0	0	0	2	0	1	1	1
1679	2022	9	F	22	0	1	0	0	0	1	0	0	0	0	1	1	0	0	0	2	1	1	1	1
1680	2023	2	M	22	0	0	0	0	1	1	0	0	0	0	1	0	0	0	0	NA	0	0	0	0
1681	2020	5	F	22	0	0	1	1	0	0	0	1	0	0	1	1	1	0	0	2	1	0	1	1
1682	2020	6	M	22	1	1	0	0	1	0	0	0	0	0	1	1	0	0	0	2	1	1	1	0
1683	2021	11	F	22	0	1	0	0	0	1	1	0	0	0	1	0	0	0	0	NA	0	0	0	0
1684	2023	10	F	22	0	1	0	0	0	1	1	0	0	0	1	0	0	0	0	2	0	1	0	0
1685	2022	3	F	22	0	1	1	1	0	0	0	1	0	0	1	1	0	0	0	2	1	1	1	1
1686	2021	9	F	22	0	1	1	0	0	1	0	0	0	1	1	0	0	0	0	2	1	1	0	0
1687	2020	9	M	22	0	1	1	1	1	0	0	0	0	0	1	0	1	0	0	2	0	1	0	0
1688	2023	1	F	22	1	1	1	0	0	1	0	0	0	0	1	1	0	0	0	2	0	0	1	1
1689	2022	9	F	22	0	1	0	0	1	1	1	0	0	0	1	1	1	0	0	NA	0	1	0	0
1690	2024	5	M	22	1	1	1	1	1	0	0	0	0	0	1	1	0	0	0	NA	0	0	0	0
1691	2020	2	M	23	0	0	0	0	0	0	0	0	0	0	0	0	0	0	0	NA	0	0	0	0
1692	2020	3	F	23	0	0	0	0	0	0	0	0	0	0	1	1	0	0	0	2	0	1	0	0
1693	2020	8	F	23	0	0	0	0	0	0	0	0	0	0	0	0	0	0	0	NA	0	0	0	0
1694	2020	9	F	23	0	0	0	0	0	0	0	0	0	0	1	1	1	0	0	2	1	0	0	1
1695	2020	9	F	23	0	0	0	0	0	0	0	0	0	0	0	0	0	0	1	2	1	1	1	1
1696	2021	1	M	23	0	0	0	0	0	0	0	0	0	0	0	0	1	0	0	NA	0	0	0	0
1697	2021	11	M	23	0	0	0	0	0	0	0	0	0	0	0	0	0	0	0	NA	0	0	0	0
1698	2021	3	M	23	0	0	0	0	0	0	0	0	0	0	1	0	0	0	0	NA	0	0	0	0
1699	2021	3	F	23	0	0	0	0	0	0	0	0	0	0	1	0	0	0	0	2	0	1	1	0
1700	2021	5	F	23	0	0	0	0	0	0	0	0	0	0	0	0	0	1	0	NA	0	0	0	0
1701	2022	1	F	23	0	0	0	0	0	0	0	0	0	0	0	0	0	0	1	NA	0	0	0	0

ID	Yr	Mo	Sex	Age	Tre	Grs	Rag	Mug	HDM	Dan	Mld	Food	Drg	Ven	Rhi	Cnj	Ast	AsE	Urt	Sev	Prt	Obs	Rho	Snz
1702	2022	2	F	23	0	0	0	0	0	0	0	0	0	0	1	0	0	0	0	2	0	1	1	1
1703	2022	2	F	23	0	0	0	0	0	0	0	0	0	0	0	0	0	0	1	NA	0	0	0	0
1704	2022	3	F	23	0	0	0	0	0	0	0	0	0	0	1	0	0	0	1	NA	0	0	0	0
1705	2022	6	F	23	0	0	0	0	0	0	0	0	0	0	0	0	0	0	1	NA	0	0	0	0
1706	2022	10	F	23	0	0	0	0	0	0	0	0	0	0	0	0	0	0	1	2	1	0	1	1
1707	2022	12	F	23	0	0	0	0	0	0	0	0	0	0	0	0	0	0	0	2	0	1	1	0
1708	2023	4	M	23	0	0	0	0	0	0	0	0	0	0	0	0	0	0	1	NA	0	0	0	0
1709	2023	5	M	23	0	0	0	0	0	0	0	0	0	0	0	0	0	0	1	2	0	1	0	0
1710	2024	1	F	23	0	0	0	0	0	0	0	0	0	0	0	0	0	0	0	2	1	1	1	0
1711	2024	3	F	23	0	0	0	0	0	0	0	0	0	0	0	0	0	0	0	NA	0	0	0	0
1712	2024	3	M	23	0	0	0	0	0	0	0	0	0	0	0	0	1	0	0	NA	0	0	0	0
1713	2024	9	F	23	0	0	0	0	0	0	0	0	0	0	0	0	0	0	0	2	1	1	1	1
1714	2024	10	F	23	0	0	0	0	0	0	0	0	0	0	1	0	1	0	1	NA	0	0	0	0
1715	2024	11	F	23	0	0	0	0	0	0	0	0	0	0	0	0	0	0	0	2	0	1	1	0
1716	2024	11	F	23	0	0	0	0	0	0	0	0	0	0	0	0	1	0	1	NA	0	1	1	0
1717	2024	12	F	23	0	0	0	0	0	0	0	0	0	0	0	0	0	0	1	NA	0	0	0	0
1718	2024	12	M	23	0	0	0	0	0	0	0	0	0	0	0	0	0	0	1	NA	0	0	0	0
1719	2021	11	M	23	0	0	0	0	0	0	0	0	0	1	0	0	0	0	0	2	0	1	0	0
1720	2021	10	M	23	0	0	0	0	0	0	0	0	0	1	0	0	0	0	0	NA	0	0	1	1
1721	2021	11	M	23	0	0	0	0	0	0	0	0	0	1	0	1	0	0	0	NA	0	0	0	0
1722	2024	8	M	23	0	0	0	0	0	0	0	0	1	0	1	0	1	0	0	NA	0	0	0	0
1723	2020	2	M	23	0	0	0	0	1	0	0	0	0	0	1	0	0	0	0	2	0	1	1	1
1724	2021	4	M	23	0	0	0	0	0	0	1	0	0	0	1	0	0	0	0	NA	0	0	0	0
1725	2021	8	M	23	0	0	0	0	1	0	0	0	0	0	1	0	1	0	0	2	1	1	1	1
1726	2021	9	M	23	0	0	0	0	1	0	0	0	0	0	1	1	0	0	0	2	0	1	1	1
1727	2020	2	M	23	0	0	1	0	0	0	0	0	0	0	1	1	0	0	0	2	0	0	1	0
1728	2022	9	F	23	0	0	1	0	0	0	0	0	0	0	1	0	1	0	0	NA	0	0	0	0
1729	2022	10	M	23	0	0	1	0	0	0	0	0	0	0	1	1	0	0	0	2	0	1	0	0
1730	2023	11	F	23	0	0	1	0	0	0	0	0	0	0	1	0	1	0	1	NA	0	0	0	0
1731	2024	9	F	23	0	0	1	0	0	0	0	0	0	0	1	1	1	0	0	NA	0	0	0	0
1732	2022	3	M	23	0	1	0	0	0	0	0	0	0	0	1	1	0	0	0	NA	0	0	0	0
1733	2023	9	M	23	0	0	0	0	0	0	1	0	0	0	1	0	1	0	0	2	0	1	1	1
1734	2023	10	F	23	0	1	0	0	0	0	0	0	0	0	1	1	1	0	0	NA	0	0	0	0
1735	2021	9	F	23	0	0	1	0	0	0	0	0	0	1	1	0	0	0	0	2	1	1	1	1
1736	2022	3	M	23	0	0	0	0	1	0	0	1	0	0	1	0	1	0	0	2	1	1	1	1
1737	2021	10	F	23	0	1	0	0	0	0	0	1	0	1	0	0	0	0	0	2	0	1	1	1
1738	2020	10	M	23	1	0	0	0	1	0	0	0	0	0	1	1	1	0	0	NA	0	0	0	0
1739	2022	5	M	23	0	1	1	0	0	0	0	0	0	0	1	1	0	0	0	2	0	1	0	0
1740	2023	5	F	23	0	1	1	0	0	0	0	0	0	0	1	0	0	0	0	2	0	1	0	0
1741	2023	7	M	23	0	1	1	0	0	0	0	0	0	0	1	1	0	0	0	1	0	1	0	0
1742	2024	5	F	23	0	1	1	0	0	0	0	0	0	0	1	0	0	0	0	2	0	0	1	0
1743	2023	9	M	23	0	0	0	0	1	1	0	0	0	0	1	0	0	0	1	2	0	0	0	0
1744	2021	8	F	23	0	0	1	0	0	1	0	0	0	1	1	1	1	0	0	2	1	0	0	1
1745	2020	9	F	23	0	0	1	0	1	1	0	0	0	0	1	0	1	0	0	2	1	1	1	1
1746	2022	11	M	23	0	0	1	0	1	0	1	0	0	0	1	0	0	0	0	2	1	1	1	1
1747	2024	10	M	23	0	0	1	0	1	0	1	0	0	0	1	0	0	0	0	NA	0	0	0	0
1748	2022	5	M	23	1	1	0	0	0	1	0	0	0	0	1	1	0	0	0	2	0	1	1	1
1749	2022	3	F	23	1	0	1	0	1	0	0	1	0	0	1	1	0	0	1	2	1	1	1	1
1750	2020	10	M	23	1	1	0	0	1	0	1	0	0	0	1	0	1	0	0	2	1	1	1	1
1751	2023	3	F	23	0	1	1	1	1	0	1	0	0	0	1	1	0	0	0	2	0	1	0	0
1752	2020	2	F	24	0	0	0	0	0	0	0	0	0	0	0	0	0	0	1	2	0	1	1	0
1753	2020	6	F	24	0	0	0	0	0	0	0	0	0	0	1	0	1	0	0	2	0	0	1	1
1754	2020	7	F	24	0	0	0	0	0	0	0	0	0	0	0	0	0	0	1	NA	0	0	0	0
1755	2020	8	M	24	0	0	0	0	0	0	0	0	0	0	1	0	0	0	0	2	1	1	1	1
1756	2021	5	F	24	0	0	0	0	0	0	0	0	0	0	1	1	0	0	0	NA	0	0	0	0
1757	2021	6	F	24	0	0	0	0	0	0	0	0	0	0	0	0	0	0	1	2	1	0	1	1
1758	2021	6	M	24	0	0	0	0	0	0	0	0	0	0	1	1	0	0	1	1	1	1	1	0
1759	2021	10	F	24	0	0	0	0	0	0	0	0	0	0	0	1	0	0	0	NA	0	0	0	0
1760	2021	10	M	24	0	0	0	0	0	0	0	0	0	0	1	1	0	0	0	2	0	1	0	0
1761	2021	11	F	24	0	0	0	0	0	0	0	0	0	0	0	0	0	1	0	2	1	1	0	0
1762	2022	3	M	24	0	0	0	0	0	0	0	0	0	0	1	0	0	0	0	NA	0	0	0	0
1763	2022	3	M	24	0	0	0	0	0	0	0	0	0	0	1	0	0	0	0	NA	0	0	0	0
1764	2022	11	F	24	0	0	0	0	0	0	0	0	0	0	1	0	0	0	0	NA	0	0	0	0

ID	Yr	Mo	Sex	Age	Tre	Grs	Rag	Mug	HDM	Dan	Mld	Food	Drg	Ven	Rhi	Cnj	Ast	AsE	Urt	Sev	Prt	Obs	Rho	Snz
1765	2022	11	F	24	0	0	0	0	0	0	0	0	0	0	0	0	0	0	0	2	1	1	1	1
1766	2023	4	F	24	0	0	0	0	0	0	0	0	0	0	1	0	0	0	0	NA	0	0	0	0
1767	2023	4	F	24	0	0	0	0	0	0	0	0	0	0	1	0	0	0	0	NA	0	0	0	0
1768	2023	8	M	24	0	0	0	0	0	0	0	0	0	0	0	0	0	0	0	NA	0	0	0	0
1769	2023	11	F	24	0	0	0	0	0	0	0	0	0	0	0	0	1	0	0	2	1	1	1	1
1770	2024	4	F	24	0	0	0	0	0	0	0	0	0	0	0	0	0	0	0	NA	0	0	0	0
1771	2024	9	F	24	0	0	0	0	0	0	0	0	0	0	1	1	0	0	0	2	1	1	1	1
1772	2024	9	F	24	0	0	0	0	0	0	0	0	0	0	1	0	0	0	0	NA	0	0	0	0
1773	2024	12	M	24	0	0	0	0	0	0	0	0	0	0	0	0	0	0	0	NA	0	0	0	0
1774	2020	9	F	24	0	1	0	0	0	0	0	0	0	0	1	1	1	0	0	2	0	0	1	1
1775	2020	9	F	24	0	0	0	0	1	0	0	0	0	0	1	0	1	0	0	2	1	1	0	1
1776	2021	3	F	24	1	0	0	0	0	0	0	0	0	0	1	0	0	0	0	NA	0	0	0	0
1777	2022	2	F	24	0	0	0	0	0	1	0	0	0	0	1	0	1	0	0	2	1	0	1	1
1778	2022	11	M	24	0	0	0	0	1	0	0	0	0	0	1	0	1	0	0	NA	0	0	0	0
1779	2023	4	F	24	0	0	0	0	1	0	0	0	0	0	1	0	0	0	0	2	1	1	1	1
1780	2023	4	F	24	0	1	0	0	0	0	0	0	0	0	1	1	1	0	0	NA	0	0	0	0
1781	2021	5	F	24	0	0	0	1	0	0	0	0	0	1	1	0	0	0	0	NA	0	0	0	0
1782	2021	2	M	24	0	0	1	0	0	0	0	1	0	0	1	1	1	0	1	NA	0	0	0	0
1783	2024	8	F	24	0	0	0	0	1	0	0	0	1	0	1	1	1	0	0	2	0	1	1	0
1784	2021	9	M	24	0	1	1	0	0	0	0	0	0	0	1	1	0	0	0	NA	0	0	0	0
1785	2022	6	M	24	0	1	1	0	0	0	0	0	0	0	1	0	0	0	0	2	0	1	0	1
1786	2024	4	F	24	0	1	1	0	0	0	0	0	0	0	1	1	0	0	0	2	1	1	1	1
1787	2024	9	F	24	0	0	1	0	0	1	0	0	0	0	1	0	0	0	0	NA	0	0	0	0
1788	2024	4	F	24	0	1	0	0	0	0	1	0	0	0	1	0	0	0	0	2	1	0	1	1
1789	2024	7	M	24	0	1	0	0	0	0	1	0	0	0	1	1	0	0	1	NA	0	0	0	0
1790	2021	4	M	24	0	1	0	1	0	0	0	0	0	1	1	0	0	0	0	2	1	1	1	1
1791	2023	5	M	24	1	1	1	0	0	0	0	0	0	0	1	0	0	0	0	NA	1	0	0	1
1792	2021	2	M	24	0	1	1	0	1	0	0	0	1	0	1	1	0	0	0	2	0	1	1	0
1793	2021	6	F	24	1	1	1	0	0	0	1	0	0	0	1	1	0	0	0	NA	0	0	0	0
1794	2023	3	M	24	1	1	1	1	0	0	0	0	0	0	1	1	0	0	0	NA	0	0	0	0
1795	2023	9	F	24	0	1	1	0	1	1	0	0	0	0	1	1	1	0	0	2	1	1	1	0
1796	2021	10	F	24	0	0	1	0	1	1	1	0	1	0	1	1	0	0	0	2	0	1	0	0
1797	2020	3	M	25	0	0	0	0	0	0	0	0	0	0	0	0	0	0	1	NA	0	0	0	0
1798	2020	7	F	25	0	0	0	0	0	0	0	0	0	0	1	1	0	0	0	2	1	1	0	0
1799	2020	7	F	25	0	0	0	0	0	0	0	0	0	0	0	0	0	0	0	NA	0	0	0	0
1800	2020	7	F	25	0	0	0	0	0	0	0	0	0	0	0	0	0	0	0	NA	0	0	0	0
1801	2020	9	F	25	0	0	0	0	0	0	0	0	0	0	0	0	0	0	0	NA	0	0	0	0
1802	2020	10	M	25	0	0	0	0	0	0	0	0	0	0	0	0	0	0	0	2	1	1	1	1
1803	2020	9	F	25	0	0	0	0	0	0	0	0	0	0	1	0	0	0	0	2	0	1	0	1
1804	2020	11	F	25	0	0	0	0	0	0	0	0	0	0	1	0	1	0	0	2	0	1	1	1
1805	2021	2	F	25	0	0	0	0	0	0	0	0	0	0	0	0	0	0	0	NA	0	0	0	0
1806	2021	2	F	25	0	0	0	0	0	0	0	0	0	0	0	0	0	0	0	2	0	1	0	1
1807	2021	5	F	25	0	0	0	0	0	0	0	0	0	0	0	0	0	0	1	NA	0	0	0	0
1808	2021	6	M	25	0	0	0	0	0	0	0	0	0	0	0	0	1	0	0	NA	0	0	0	0
1809	2021	6	M	25	0	0	0	0	0	0	0	0	0	0	1	1	0	0	0	2	1	0	0	1
1810	2021	7	F	25	0	0	0	0	0	0	0	0	0	0	1	0	0	0	1	2	0	0	1	0
1811	2021	11	F	25	0	0	0	0	0	0	0	0	0	0	0	0	1	0	0	NA	0	0	0	0
1812	2021	5	F	25	0	0	0	0	0	0	0	0	0	0	0	1	0	0	0	2	1	1	1	1
1813	2021	3	F	25	0	0	0	0	0	0	0	0	0	0	0	0	1	0	1	2	0	1	1	0
1814	2021	8	F	25	0	0	0	0	0	0	0	0	0	0	0	0	0	1	0	2	1	1	1	1
1815	2022	2	F	25	0	0	0	0	0	0	0	0	0	0	2	0	0	0	0	2	1	1	1	1
1816	2022	2	F	25	0	0	0	0	0	0	0	0	0	0	1	0	0	0	1	1	0	0	0	0
1817	2022	3	F	25	0	0	0	0	0	0	0	0	0	0	1	1	0	0	0	NA	0	0	0	0
1818	2022	5	F	25	0	0	0	0	0	0	0	0	0	0	0	0	0	0	1	NA	0	0	0	0
1819	2022	6	F	25	0	0	0	0	0	0	0	0	0	0	1	0	0	0	0	1	1	0	0	0
1820	2022	9	M	25	0	0	0	0	0	0	0	0	0	0	0	0	0	0	0	NA	0	0	0	0
1821	2022	11	M	25	0	0	0	0	0	0	0	0	0	0	1	0	0	0	0	2	0	1	1	1
1822	2023	2	F	25	0	0	0	0	0	0	0	0	0	0	1	0	0	0	0	NA	0	0	0	0
1823	2023	3	F	25	0	0	0	0	0	0	0	0	0	0	0	0	0	0	1	NA	0	0	0	0
1824	2023	3	F	25	0	0	0	0	0	0	0	0	0	0	0	0	0	0	1	NA	0	0	0	0
1825	2023	3	F	25	0	0	0	0	0	0	0	0	0	0	0	0	0	0	1	2	0	1	1	1
1826	2023	3	F	25	0	0	0	0	0	0	0	0	0	0	0	0	0	0	1	NA	0	0	0	0
1827	2023	8	F	25	0	0	0	0	0	0	0	0	0	0	1	1	0	0	0	NA	0	0	0	0

ID	Yr	Mo	Sex	Age	Tre	Grs	Rag	Mug	HDM	Dan	Mld	Food	Drg	Ven	Rhi	Cnj	Ast	AsE	Urt	Sev	Prt	Obs	Rho	Snz
1828	2023	9	F	25	0	0	0	0	0	0	0	0	0	0	0	0	0	0	1	2	1	1	1	1
1829	2023	10	F	25	0	0	0	0	0	0	0	0	0	0	0	0	0	0	1	1	1	0	0	0
1830	2023	11	F	25	0	0	0	0	0	0	0	0	0	0	0	0	0	0	1	NA	0	0	0	0
1831	2023	11	F	25	0	0	0	0	0	0	0	0	0	0	1	0	0	0	0	NA	0	0	0	0
1832	2024	4	F	25	0	0	0	0	0	0	0	0	0	0	0	0	0	0	0	2	0	1	1	0
1833	2024	4	M	25	0	0	0	0	0	0	0	0	0	0	0	0	0	0	0	NA	0	0	1	1
1834	2024	4	F	25	0	0	0	0	0	0	0	0	0	0	1	1	0	0	0	NA	0	0	0	0
1835	2024	6	F	25	0	0	0	0	0	0	0	0	0	0	0	0	0	0	0	NA	0	0	0	0
1836	2024	8	F	25	0	0	0	0	0	0	0	0	0	0	0	1	0	0	0	1	0	1	0	0
1837	2024	10	F	25	0	0	0	0	0	0	0	0	0	0	1	1	0	0	0	NA	0	1	0	0
1838	2024	10	F	25	0	0	0	0	0	0	0	0	0	0	1	0	0	0	0	2	0	1	1	1
1839	2020	1	F	25	0	0	0	0	0	0	0	0	1	0	1	0	1	0	0	NA	0	0	0	0
1840	2021	10	F	25	0	0	0	0	0	0	0	1	0	0	1	1	0	0	0	2	1	1	1	1
1841	2022	9	F	25	0	0	0	0	0	0	0	0	1	1	0	0	0	0	0	1	0	1	1	0
1842	2021	7	F	25	0	0	0	0	1	0	0	0	0	0	1	0	0	0	0	NA	0	0	0	0
1843	2021	9	F	25	0	0	0	0	0	0	1	0	0	0	0	0	0	0	1	2	0	1	1	0
1844	2020	1	M	25	0	0	1	0	0	0	0	0	0	0	1	1	1	0	0	2	1	0	1	1
1845	2023	8	M	25	0	0	1	0	0	0	0	0	0	0	1	1	0	0	0	NA	0	0	0	0
1846	2024	9	M	25	0	0	1	0	0	0	0	0	0	0	1	1	0	0	0	NA	0	0	0	0
1847	2022	1	M	25	0	0	0	0	1	0	0	0	0	0	1	0	0	0	0	2	1	1	0	1
1848	2022	6	M	25	0	0	0	0	1	0	0	0	0	0	1	1	0	0	0	2	1	1	1	1
1849	2022	6	M	25	0	0	0	0	1	0	0	0	0	0	1	1	0	0	0	2	1	1	1	1
1850	2023	6	M	25	0	0	0	0	1	0	0	0	0	0	1	0	0	0	1	2	1	1	1	1
1851	2024	3	F	25	1	0	0	0	0	0	0	0	0	0	1	1	0	0	0	NA	0	0	0	0
1852	2024	12	F	25	0	0	0	0	0	0	1	0	0	0	1	1	0	0	0	NA	0	0	0	0
1853	2021	5	F	25	0	0	0	0	1	0	0	0	0	1	0	1	0	0	0	2	1	1	1	1
1854	2020	7	M	25	0	1	0	0	1	0	0	0	0	0	1	0	1	0	0	NA	0	0	0	0
1855	2020	11	F	25	0	0	0	0	1	1	0	0	0	0	1	1	0	0	0	NA	0	0	0	0
1856	2021	5	F	25	0	1	0	0	1	0	0	0	0	0	1	1	0	0	0	NA	0	0	0	0
1857	2020	7	M	25	0	1	1	0	0	0	0	0	0	0	1	1	0	0	0	2	1	1	0	1
1858	2020	9	F	25	0	1	1	0	0	0	0	0	0	0	1	1	0	0	0	NA	0	0	0	0
1859	2022	11	F	25	0	0	0	0	1	1	0	0	0	0	1	0	0	0	0	NA	0	0	0	0
1860	2023	8	F	25	1	1	0	0	0	0	0	0	0	0	1	0	0	0	0	2	0	1	0	0
1861	2021	10	M	25	0	0	0	0	1	0	1	0	0	1	1	0	0	0	0	NA	0	0	0	0
1862	2021	9	M	25	0	1	1	0	0	0	0	0	0	1	1	0	0	0	0	2	0	1	1	0
1863	2020	3	M	25	0	1	1	1	0	0	0	0	0	0	1	1	0	0	0	2	1	1	1	1
1864	2020	12	M	25	0	1	1	0	1	0	0	0	0	0	1	1	0	0	0	NA	0	0	0	0
1865	2022	3	M	25	1	1	1	0	0	0	0	0	0	0	1	1	0	0	0	2	0	1	0	0
1866	2024	8	M	25	0	1	1	0	1	0	0	0	0	0	1	1	0	0	1	NA	0	0	0	0
1867	2024	7	F	25	1	0	0	1	1	0	0	0	0	0	0	0	0	0	0	NA	0	0	0	0
1868	2021	11	F	25	1	1	0	0	1	0	0	0	0	1	0	1	0	0	0	NA	0	0	0	0
1869	2022	5	F	25	1	1	1	0	1	1	0	0	0	0	1	1	1	0	0	NA	0	0	0	0
1870	2020	2	M	26	0	0	0	0	0	0	0	0	0	0	0	0	1	0	0	NA	0	0	0	0
1871	2020	3	F	26	0	0	0	0	0	0	0	0	0	0	1	0	0	0	0	2	1	1	1	1
1872	2020	7	M	26	0	0	0	0	0	0	0	0	0	0	1	0	0	0	0	NA	0	0	0	0
1873	2020	8	M	26	0	0	0	0	0	0	0	0	0	0	1	0	0	0	0	NA	0	0	0	0
1874	2020	9	M	26	0	0	0	0	0	0	0	0	0	0	1	1	0	0	0	NA	0	0	0	0
1875	2021	8	F	26	0	0	0	0	0	0	0	0	0	0	1	1	0	0	1	2	1	0	1	1
1876	2021	11	F	26	0	0	0	0	0	0	0	0	0	0	0	0	0	0	0	2	1	1	1	1
1877	2021	10	F	26	0	0	0	0	0	0	0	0	0	0	0	0	0	0	0	NA	0	0	0	0
1878	2022	1	M	26	0	0	0	0	0	0	0	0	0	0	0	0	0	0	1	NA	0	0	0	0
1879	2022	3	F	26	0	0	0	0	0	0	0	0	0	0	0	0	0	0	0	NA	0	0	0	0
1880	2022	5	M	26	0	0	0	0	0	0	0	0	0	0	1	1	0	0	0	2	1	1	1	1
1881	2022	6	F	26	0	0	0	0	0	0	0	0	0	0	0	0	0	0	1	2	1	0	1	1
1882	2022	6	F	26	0	0	0	0	0	0	0	0	0	0	1	0	0	0	0	2	1	1	1	1
1883	2022	9	F	26	0	0	0	0	0	0	0	0	0	0	1	1	0	0	0	NA	0	0	0	0
1884	2022	11	M	26	0	0	0	0	0	0	0	0	0	0	1	0	0	0	0	NA	0	0	0	0
1885	2023	2	F	26	0	0	0	0	0	0	0	0	0	0	0	0	0	0	1	2	1	1	1	1
1886	2023	3	F	26	0	0	0	0	0	0	0	0	0	0	0	0	0	0	1	NA	0	0	0	0
1887	2023	5	F	26	0	0	0	0	0	0	0	0	0	0	0	0	0	0	1	NA	0	0	0	0
1888	2023	5	F	26	0	0	0	0	0	0	0	0	0	0	0	0	0	0	1	1	1	0	0	1
1889	2023	7	F	26	0	0	0	0	0	0	0	0	0	0	0	0	0	0	1	NA	0	0	0	0
1890	2023	11	M	26	0	0	0	0	0	0	0	0	0	0	0	0	0	0	1	NA	0	0	1	0

ID	Yr	Mo	Sex	Age	Tre	Grs	Rag	Mug	HDM	Dan	Mld	Food	Drg	Ven	Rhi	Cnj	Ast	AsE	Urt	Sev	Prt	Obs	Rho	Snz
1891	2024	3	F	26	0	0	0	0	0	0	0	0	0	0	0	0	0	0	1	2	0	1	0	0
1892	2024	4	F	26	0	0	0	0	0	0	0	0	0	0	0	0	0	0	0	NA	0	0	0	0
1893	2024	4	F	26	0	0	0	0	0	0	0	0	0	0	1	0	1	0	1	NA	0	0	0	0
1894	2024	6	F	26	0	0	0	0	0	0	0	0	0	0	0	0	0	0	1	NA	0	0	0	0
1895	2024	6	M	26	0	0	0	0	0	0	0	0	0	0	0	0	0	0	0	NA	0	0	0	0
1896	2024	6	F	26	0	0	0	0	0	0	0	0	0	0	0	0	0	0	1	NA	0	0	0	0
1897	2024	7	M	26	0	0	0	0	0	0	0	0	0	0	0	0	0	0	0	NA	0	0	0	0
1898	2024	7	F	26	0	0	0	0	0	0	0	0	0	0	0	0	0	0	0	2	1	1	1	1
1899	2024	7	F	26	0	0	0	0	0	0	0	0	0	0	0	0	0	0	0	NA	0	1	1	1
1900	2024	10	F	26	0	0	0	0	0	0	0	0	0	0	1	0	1	0	0	NA	0	0	0	0
1901	2024	12	M	26	0	0	0	0	0	0	0	0	0	0	1	1	0	0	0	NA	0	0	0	0
1902	2021	9	F	26	0	0	0	0	0	0	0	0	0	1	1	0	0	0	0	NA	0	0	0	0
1903	2021	12	F	26	0	0	0	0	0	0	0	0	1	0	0	0	0	0	0	NA	0	0	0	0
1904	2021	11	F	26	0	0	0	0	0	0	0	0	0	1	0	0	0	1	0	NA	0	0	0	0
1905	2023	4	M	26	0	0	0	0	0	0	0	0	1	0	0	0	0	0	0	NA	0	0	0	0
1906	2020	9	F	26	0	0	0	1	0	0	0	0	0	0	1	0	1	0	0	2	1	1	1	1
1907	2021	10	F	26	1	0	0	0	0	0	0	0	0	0	0	0	0	0	1	2	0	1	1	1
1908	2020	1	M	26	0	0	1	0	0	0	0	0	0	0	1	1	0	0	0	1	1	1	1	1
1909	2020	10	M	26	0	0	1	0	0	0	0	0	0	0	1	1	0	0	0	NA	0	0	0	0
1910	2022	5	M	26	0	0	1	0	0	0	0	0	0	0	1	1	0	0	0	NA	0	0	0	0
1911	2022	8	M	26	0	0	1	0	0	0	0	0	0	0	1	0	0	0	0	1	1	0	0	0
1912	2024	10	M	26	0	0	1	0	0	0	0	0	0	0	1	0	0	0	1	NA	0	0	0	0
1913	2022	2	F	26	1	0	0	0	0	0	0	0	0	0	1	1	0	0	1	2	0	1	1	1
1914	2022	3	M	26	0	0	0	0	1	0	0	0	0	0	1	0	0	0	0	2	0	0	1	1
1915	2022	4	F	26	0	0	0	0	0	1	0	0	0	0	1	0	0	0	1	NA	0	0	0	0
1916	2022	5	M	26	0	1	0	0	0	0	0	0	0	0	1	1	0	0	0	NA	0	0	0	0
1917	2023	4	M	26	0	1	0	0	0	0	0	0	0	0	1	1	0	0	0	NA	0	0	0	0
1918	2021	8	M	26	0	1	0	0	0	0	0	0	0	1	1	0	0	0	0	2	0	1	1	0
1919	2021	11	M	26	0	0	1	0	0	0	0	0	0	1	1	0	0	0	0	NA	0	0	0	0
1920	2022	6	M	26	0	0	0	0	1	0	0	1	0	0	1	0	1	0	0	NA	0	0	0	0
1921	2023	8	M	26	0	1	0	0	0	0	0	0	1	0	1	0	1	0	0	2	0	1	1	1
1922	2024	10	M	26	0	1	0	0	0	0	0	1	0	0	0	0	0	0	0	NA	0	0	0	0
1923	2021	9	F	26	0	0	1	0	0	0	1	0	0	0	1	1	0	0	0	NA	0	0	0	0
1924	2022	2	M	26	0	0	1	1	0	0	0	0	0	0	1	0	0	0	0	NA	0	1	0	0
1925	2022	4	F	26	0	1	1	0	0	0	0	0	0	0	1	1	1	0	0	NA	0	0	0	0
1926	2023	2	F	26	0	0	1	0	0	1	0	0	0	0	1	0	1	0	0	NA	0	0	0	0
1927	2023	6	F	26	0	0	1	0	1	0	0	0	0	0	1	0	0	0	0	NA	0	0	0	0
1928	2022	11	F	26	0	0	0	0	1	1	0	0	0	0	1	1	0	0	1	2	1	1	0	0
1929	2024	9	M	26	0	1	0	1	0	0	0	0	0	0	1	0	0	0	1	NA	0	0	0	0
1930	2021	5	F	26	1	0	0	0	0	1	0	0	0	1	1	0	0	0	0	NA	0	0	0	0
1931	2020	9	F	26	0	0	1	0	1	1	0	0	0	0	1	0	0	0	0	NA	0	0	0	0
1932	2023	6	M	26	0	1	1	0	0	0	1	0	0	0	1	0	0	0	0	NA	0	1	0	0
1933	2021	9	F	26	0	0	1	0	1	1	1	0	0	1	1	0	0	0	0	2	1	0	1	1
1934	2021	5	F	26	1	1	1	1	1	0	0	0	0	0	1	1	0	0	1	2	1	0	0	1
1935	2021	3	F	26	0	1	1	1	0	1	1	0	0	0	1	1	0	0	0	NA	0	0	0	0
1936	2020	1	F	27	0	0	0	0	0	0	0	0	0	0	0	0	0	0	1	NA	0	0	0	0
1937	2020	1	F	27	0	0	0	0	0	0	0	0	0	0	0	0	1	0	1	NA	0	0	0	0
1938	2020	2	F	27	0	0	0	0	0	0	0	0	0	0	0	0	0	0	1	NA	0	0	0	0
1939	2020	3	F	27	0	0	0	0	0	0	0	0	0	0	0	0	1	0	1	NA	0	0	0	0
1940	2020	8	M	27	0	0	0	0	0	0	0	0	0	0	1	0	0	0	0	NA	1	1	0	0
1941	2020	8	F	27	0	0	0	0	0	0	0	0	0	0	0	0	0	0	1	2	1	1	1	1
1942	2020	9	M	27	0	0	0	0	0	0	0	0	0	0	0	0	0	0	0	NA	0	0	0	0
1943	2020	9	F	27	0	0	0	0	0	0	0	0	0	0	1	0	0	0	0	2	0	1	0	1
1944	2020	10	F	27	0	0	0	0	0	0	0	0	0	0	0	0	0	0	0	NA	0	0	0	0
1945	2021	1	F	27	0	0	0	0	0	0	0	0	0	0	1	0	0	0	0	NA	0	0	0	0
1946	2021	2	M	27	0	0	0	0	0	0	0	0	0	0	1	0	0	0	0	2	1	1	1	1
1947	2021	4	F	27	0	0	0	0	0	0	0	0	0	0	0	0	0	0	1	2	1	1	1	1
1948	2021	4	F	27	0	0	0	0	0	0	0	0	0	0	1	0	0	0	1	2	0	1	1	1
1949	2021	9	F	27	0	0	0	0	0	0	0	0	0	0	1	0	0	0	0	2	0	1	0	0
1950	2021	10	F	27	0	0	0	0	0	0	0	0	0	0	0	0	0	1	0	NA	0	0	0	0
1951	2021	2	F	27	0	0	0	0	0	0	0	0	0	0	1	0	0	0	0	2	1	1	1	1
1952	2022	2	F	27	0	0	0	0	0	0	0	0	0	0	0	0	0	0	0	2	0	1	0	0
1953	2022	3	F	27	0	0	0	0	0	0	0	0	0	0	0	0	0	0	1	1	1	1	1	1

ID	Yr	Mo	Sex	Age	Tre	Grs	Rag	Mug	HDM	Dan	Mld	Food	Drg	Ven	Rhi	Cnj	Ast	AsE	Urt	Sev	Prt	Obs	Rho	Snz
1954	2022	5	F	27	0	0	0	0	0	0	0	0	0	0	1	0	0	0	0	NA	0	0	0	0
1955	2022	5	F	27	0	0	0	0	0	0	0	0	0	0	0	0	0	0	1	NA	0	0	0	0
1956	2023	2	F	27	0	0	0	0	0	0	0	0	0	0	0	0	1	0	0	2	1	1	1	0
1957	2023	3	M	27	0	0	0	0	0	0	0	0	0	0	1	0	0	0	0	NA	0	0	0	0
1958	2023	4	F	27	0	0	0	0	0	0	0	0	0	0	0	0	0	0	1	2	0	1	1	1
1959	2023	4	F	27	0	0	0	0	0	0	0	0	0	0	1	0	1	0	0	2	0	1	1	0
1960	2023	6	F	27	0	0	0	0	0	0	0	0	0	0	0	0	0	0	0	NA	0	0	0	0
1961	2023	6	F	27	0	0	0	0	0	0	0	0	0	0	0	0	0	0	1	NA	0	0	0	0
1962	2023	6	F	27	0	0	0	0	0	0	0	0	0	0	0	0	0	0	1	1	0	1	0	0
1963	2023	6	F	27	0	0	0	0	0	0	0	0	0	0	1	0	0	0	0	NA	0	0	0	0
1964	2024	2	F	27	0	0	0	0	0	0	0	0	0	0	0	0	0	0	1	NA	0	0	0	0
1965	2024	2	F	27	0	0	0	0	0	0	0	0	0	0	0	0	0	0	1	1	0	1	0	0
1966	2024	2	F	27	0	0	0	0	0	0	0	0	0	0	0	0	0	0	1	NA	0	0	0	0
1967	2024	3	M	27	0	0	0	0	0	0	0	0	0	0	0	0	0	0	1	2	1	0	0	1
1968	2024	3	F	27	0	0	0	0	0	0	0	0	0	0	0	0	0	0	0	NA	0	0	0	0
1969	2024	3	F	27	0	0	0	0	0	0	0	0	0	0	0	0	0	0	0	NA	0	0	0	0
1970	2024	10	F	27	0	0	0	0	0	0	0	0	0	0	0	0	0	0	0	2	1	1	1	1
1971	2024	12	M	27	0	0	0	0	0	0	0	0	0	0	0	0	1	0	0	NA	0	0	0	0
1972	2020	1	F	27	0	0	0	0	0	0	0	0	0	1	1	0	1	0	1	NA	0	0	0	0
1973	2021	7	F	27	0	0	0	0	0	0	0	1	0	0	0	0	0	0	1	NA	0	0	0	0
1974	2021	10	M	27	0	0	0	0	0	0	0	0	0	1	0	1	0	0	0	2	1	1	1	1
1975	2023	4	F	27	0	0	0	0	0	0	0	0	1	0	0	0	0	0	0	2	0	0	1	0
1976	2023	11	F	27	0	0	0	0	0	0	0	1	0	0	0	0	0	0	1	NA	0	0	0	0
1977	2024	1	M	27	0	0	0	0	0	0	0	1	0	0	0	0	0	0	1	2	1	1	0	1
1978	2024	3	F	27	0	0	0	0	0	0	0	1	0	0	1	1	0	0	0	2	0	1	0	0
1979	2024	11	M	27	0	0	0	0	0	0	0	0	0	1	0	0	0	0	1	NA	0	0	0	0
1980	2020	7	F	27	0	0	0	0	0	1	0	0	0	0	1	1	0	0	0	1	0	0	0	0
1981	2020	9	F	27	0	0	0	0	1	0	0	0	0	0	1	1	0	0	0	NA	0	0	0	0
1982	2021	5	M	27	0	1	0	0	0	0	0	0	0	0	1	1	0	0	0	NA	0	0	0	0
1983	2020	8	F	27	0	0	1	0	0	0	0	0	0	0	1	1	1	0	0	NA	0	0	0	0
1984	2020	9	F	27	0	0	1	0	0	0	0	0	0	0	1	1	0	0	0	1	1	1	1	1
1985	2022	3	M	27	0	0	1	0	0	0	0	0	0	0	1	1	0	0	0	NA	0	0	0	0
1986	2023	9	M	27	0	0	1	0	0	0	0	0	0	0	1	1	0	0	0	NA	0	0	0	0
1987	2024	10	F	27	0	0	1	0	0	0	0	0	0	0	1	0	0	0	0	2	1	1	0	1
1988	2023	2	F	27	0	1	0	0	0	0	0	0	0	0	1	1	0	0	0	NA	0	0	0	0
1989	2024	4	M	27	0	1	0	0	0	0	0	0	0	0	1	1	0	0	0	2	1	0	0	1
1990	2024	5	F	27	0	0	0	1	0	0	0	0	0	0	1	0	1	0	1	2	1	1	1	1
1991	2021	5	M	27	0	1	0	0	0	0	0	0	0	1	1	0	0	0	0	2	0	1	1	0
1992	2021	9	F	27	0	0	1	0	0	0	0	0	0	1	1	1	0	0	0	NA	0	0	0	0
1993	2021	11	F	27	0	0	1	0	0	0	0	0	0	1	1	0	0	0	0	NA	0	0	0	0
1994	2021	12	F	27	0	0	1	0	0	0	0	0	0	1	1	0	0	1	1	2	0	1	1	1
1995	2022	2	M	27	0	0	0	0	1	0	0	0	1	0	1	1	0	0	0	2	1	1	1	1
1996	2021	3	M	27	0	0	0	0	1	0	0	1	1	0	1	0	1	0	1	1	1	1	1	1
1997	2020	3	F	27	1	1	0	0	0	1	0	0	0	0	1	1	1	0	0	2	0	0	1	1
1998	2020	7	M	27	0	1	1	0	0	1	0	0	0	0	1	1	0	0	0	2	1	1	1	1
1999	2020	10	M	27	1	1	1	0	0	0	0	0	0	0	1	1	1	0	0	2	1	1	1	1
2000	2022	1	F	27	0	1	1	0	0	1	0	0	0	0	1	1	1	0	0	NA	0	0	0	0
2001	2023	2	F	27	1	1	1	0	0	0	0	0	0	0	1	0	0	0	0	NA	0	0	0	0
2002	2023	11	F	27	1	0	1	0	1	0	0	0	0	0	0	0	0	0	1	2	0	1	0	0
2003	2024	4	F	27	1	0	1	0	0	1	0	0	0	0	1	1	0	0	0	2	1	1	1	1
2004	2023	10	F	27	0	1	0	0	1	1	0	0	0	0	1	0	0	0	1	2	0	1	0	0
2005	2021	10	F	27	0	1	1	0	0	0	1	0	0	1	1	0	0	0	0	2	1	0	0	1
2006	2023	4	F	27	1	1	1	1	0	0	0	0	0	0	1	1	0	0	0	NA	0	0	0	0
2007	2023	8	F	27	1	1	1	1	0	0	0	0	0	0	1	0	0	0	0	NA	0	0	0	0
2008	2023	2	M	27	1	0	1	0	1	1	0	1	0	0	1	1	0	0	1	2	1	0	1	1
2009	2021	6	F	27	0	1	1	0	1	1	0	0	0	1	1	0	0	0	0	NA	0	0	0	0
2010	2022	2	F	27	1	1	0	0	1	0	1	1	0	0	1	1	1	0	0	NA	0	0	0	0
2011	2021	5	F	27	1	1	1	0	1	1	0	0	0	0	1	1	1	0	0	2	0	1	1	1
2012	2021	5	M	27	0	1	1	1	1	0	1	0	0	1	1	0	0	0	0	NA	0	0	0	0
2013	2020	1	F	28	0	0	0	0	0	0	0	0	0	0	0	0	0	0	0	NA	0	0	0	0
2014	2020	2	F	28	0	0	0	0	0	0	0	0	0	0	0	0	0	0	0	NA	0	0	0	0
2015	2020	2	F	28	0	0	0	0	0	0	0	0	0	0	1	0	1	0	0	NA	0	0	0	0
2016	2020	7	F	28	0	0	0	0	0	0	0	0	0	0	0	0	0	0	1	NA	0	0	0	0

ID	Yr	Mo	Sex	Age	Tre	Grs	Rag	Mug	HDM	Dan	Mld	Food	Drg	Ven	Rhi	Cnj	Ast	AsE	Urt	Sev	Prt	Obs	Rho	Snz
2017	2020	7	F	28	0	0	0	0	0	0	0	0	0	0	0	0	0	0	1	NA	0	0	0	0
2018	2020	7	M	28	0	0	0	0	0	0	0	0	0	0	0	0	1	0	0	2	0	1	0	0
2019	2020	9	M	28	0	0	0	0	0	0	0	0	0	0	1	1	0	0	0	NA	0	0	0	0
2020	2020	9	F	28	0	0	0	0	0	0	0	0	0	0	1	0	0	0	0	NA	0	0	0	0
2021	2020	10	F	28	0	0	0	0	0	0	0	0	0	0	1	0	0	0	0	NA	0	0	0	0
2022	2020	11	M	28	0	0	0	0	0	0	0	0	0	0	1	0	1	0	0	NA	0	0	0	0
2023	2020	12	M	28	0	0	0	0	0	0	0	0	0	0	1	0	0	0	0	NA	0	0	0	0
2024	2021	2	F	28	0	0	0	0	0	0	0	0	0	0	0	0	0	0	0	2	1	1	1	0
2025	2021	4	M	28	0	0	0	0	0	0	0	0	0	0	1	0	0	0	0	2	0	1	0	0
2026	2021	6	M	28	0	0	0	0	0	0	0	0	0	0	1	0	0	0	0	2	1	1	1	0
2027	2021	11	M	28	0	0	0	0	0	0	0	0	0	0	0	0	0	0	1	2	1	1	1	0
2028	2021	10	F	28	0	0	0	0	0	0	0	0	0	0	0	0	0	1	0	NA	0	0	0	0
2029	2022	2	M	28	0	0	0	0	0	0	0	0	0	0	1	0	0	0	1	NA	0	0	0	0
2030	2022	4	F	28	0	0	0	0	0	0	0	0	0	0	0	0	0	0	1	NA	0	0	0	0
2031	2022	5	F	28	0	0	0	0	0	0	0	0	0	0	0	0	0	0	1	NA	0	0	0	0
2032	2022	5	F	28	0	0	0	0	0	0	0	0	0	0	0	0	0	0	1	NA	0	0	0	0
2033	2022	10	M	28	0	0	0	0	0	0	0	0	0	0	0	0	0	0	1	2	1	1	1	1
2034	2022	10	M	28	0	0	0	0	0	0	0	0	0	0	0	0	0	0	1	NA	0	0	0	0
2035	2022	10	F	28	0	0	0	0	0	0	0	0	0	0	0	0	0	0	1	NA	0	0	0	0
2036	2023	2	F	28	0	0	0	0	0	0	0	0	0	0	1	0	0	0	1	NA	0	0	0	0
2037	2023	2	F	28	0	0	0	0	0	0	0	0	0	0	1	0	0	0	0	2	1	1	0	1
2038	2023	4	F	28	0	0	0	0	0	0	0	0	0	0	1	0	1	0	0	NA	0	0	0	0
2039	2023	5	M	28	0	0	0	0	0	0	0	0	0	0	0	0	0	0	1	2	0	1	0	0
2040	2023	6	M	28	0	0	0	0	0	0	0	0	0	0	0	0	0	0	1	2	1	1	1	1
2041	2023	8	M	28	0	0	0	0	0	0	0	0	0	0	1	0	0	0	0	NA	0	0	0	0
2042	2023	11	F	28	0	0	0	0	0	0	0	0	0	0	1	0	0	0	0	NA	0	0	0	0
2043	2024	1	F	28	0	0	0	0	0	0	0	0	0	0	1	0	0	0	1	2	1	1	1	1
2044	2024	1	M	28	0	0	0	0	0	0	0	0	0	0	1	0	0	0	0	2	0	1	1	0
2045	2024	1	M	28	0	0	0	0	0	0	0	0	0	0	1	0	0	0	0	NA	0	0	0	0
2046	2024	1	F	28	0	0	0	0	0	0	0	0	0	0	0	0	0	0	1	NA	0	0	0	0
2047	2024	2	M	28	0	0	0	0	0	0	0	0	0	0	0	0	0	0	0	NA	0	0	0	0
2048	2024	4	F	28	0	0	0	0	0	0	0	0	0	0	0	0	0	0	0	NA	0	0	0	0
2049	2024	4	F	28	0	0	0	0	0	0	0	0	0	0	0	0	0	0	0	2	0	1	0	0
2050	2024	5	F	28	0	0	0	0	0	0	0	0	0	0	0	0	0	0	0	NA	0	0	0	0
2051	2024	6	F	28	0	0	0	0	0	0	0	0	0	0	0	0	0	0	1	2	0	1	0	0
2052	2024	8	F	28	0	0	0	0	0	0	0	0	0	0	0	0	0	0	1	NA	0	0	0	0
2053	2024	8	F	28	0	0	0	0	0	0	0	0	0	0	0	0	0	0	0	2	0	0	0	1
2054	2024	9	F	28	0	0	0	0	0	0	0	0	0	0	1	1	0	0	0	2	0	0	1	0
2055	2020	7	M	28	0	0	0	0	0	0	0	0	1	0	0	0	0	0	1	2	0	1	0	1
2056	2021	11	M	28	0	0	0	0	0	0	0	0	0	1	0	0	0	0	0	NA	0	0	0	0
2057	2021	4	M	28	0	0	0	0	0	0	0	0	0	1	0	0	0	0	0	NA	0	0	0	0
2058	2023	3	F	28	0	0	0	0	0	0	0	1	0	0	0	0	0	0	1	NA	0	0	0	0
2059	2020	1	M	28	0	0	1	0	0	0	0	0	0	0	1	1	1	0	1	2	0	1	1	1
2060	2022	3	M	28	0	0	1	0	0	0	0	0	0	0	1	1	0	0	0	2	1	1	1	0
2061	2022	8	F	28	0	0	1	0	0	0	0	0	0	0	1	1	1	0	0	2	0	1	0	1
2062	2022	9	F	28	0	0	1	0	0	0	0	0	0	0	1	1	0	0	0	NA	0	0	0	0
2063	2022	12	F	28	0	0	1	0	0	0	0	0	0	0	1	0	0	0	0	2	0	1	0	1
2064	2023	6	M	28	0	0	1	0	0	0	0	0	0	0	1	0	0	0	1	NA	0	0	0	0
2065	2023	8	F	28	0	0	1	0	0	0	0	0	0	0	1	0	0	0	0	NA	0	0	0	0
2066	2023	9	M	28	0	0	1	0	0	0	0	0	0	0	1	0	1	1	0	NA	0	0	0	0
2067	2022	1	M	28	0	0	0	0	1	0	0	0	0	0	0	0	1	0	0	2	0	0	0	0
2068	2022	2	F	28	0	0	0	0	1	0	0	0	0	0	1	0	0	0	0	NA	0	0	0	0
2069	2022	11	F	28	0	0	0	0	1	0	0	0	0	0	1	0	1	0	0	NA	0	0	0	0
2070	2022	12	M	28	0	1	0	0	0	0	0	0	0	0	1	1	1	0	0	NA	0	0	0	0
2071	2023	5	M	28	0	0	0	0	1	0	0	0	0	0	1	0	0	0	0	NA	0	1	0	0
2072	2024	1	F	28	0	0	0	0	0	1	0	0	0	0	1	1	1	0	0	NA	1	1	1	0
2073	2024	3	F	28	1	0	0	0	0	0	0	0	0	0	1	0	0	0	0	NA	0	0	0	0
2074	2024	7	M	28	0	1	0	0	0	0	0	0	0	0	1	0	0	0	0	NA	0	0	0	0
2075	2021	9	F	28	0	0	1	0	0	0	0	0	0	1	1	0	0	0	0	2	0	1	1	1
2076	2021	5	F	28	0	0	1	0	0	0	0	0	0	1	1	1	0	0	0	NA	0	0	0	0
2077	2020	7	M	28	0	1	0	0	1	0	0	0	0	0	1	0	1	0	0	2	0	0	0	1
2078	2020	7	M	28	0	1	0	0	1	0	0	0	0	0	1	1	0	0	0	NA	0	0	0	0
2079	2021	3	F	28	0	0	0	0	0	1	1	0	0	0	1	0	0	0	0	NA	0	0	0	0

ID	Yr	Mo	Sex	Age	Tre	Grs	Rag	Mug	HDM	Dan	Mld	Food	Drg	Ven	Rhi	Cnj	Ast	AsE	Urt	Sev	Prt	Obs	Rho	Snz
2080	2021	5	M	28	0	0	1	0	1	0	0	0	0	0	1	1	0	0	0	2	1	1	1	1
2081	2024	4	M	28	0	1	1	0	0	0	0	0	0	0	1	0	0	0	0	2	0	1	0	0
2082	2024	11	F	28	0	0	0	0	1	0	1	0	0	0	1	0	0	0	0	2	0	1	0	0
2083	2021	9	F	28	1	1	0	0	0	0	0	0	0	1	1	0	0	0	0	2	0	1	0	0
2084	2021	5	M	28	0	0	0	0	1	0	1	0	0	1	0	1	0	1	1	2	0	1	0	0
2085	2022	3	F	28	1	1	1	0	0	0	0	0	0	0	1	1	0	0	0	2	1	1	0	1
2086	2022	6	M	28	0	0	1	0	1	1	0	0	0	0	1	1	0	0	0	NA	0	0	0	0
2087	2023	9	F	28	0	0	1	0	1	1	0	0	0	0	1	1	0	0	0	2	1	1	1	1
2088	2023	10	M	28	0	1	1	0	0	1	0	0	0	0	1	0	0	0	1	NA	0	0	0	0
2089	2023	10	M	28	1	1	1	0	0	0	0	0	0	0	1	0	1	0	0	NA	0	0	0	0
2090	2024	12	F	28	1	0	1	0	1	0	0	0	0	0	1	1	0	0	1	NA	0	0	0	0
2091	2020	8	F	28	0	1	1	1	1	1	0	0	0	0	1	1	0	0	0	2	1	1	1	1
2092	2022	3	F	28	0	1	1	1	1	1	0	0	0	0	1	1	0	0	0	2	1	0	1	0
2093	2023	6	M	28	1	1	1	0	1	0	1	0	0	0	1	1	0	0	0	NA	0	0	0	0
2094	2022	11	F	28	1	1	1	0	1	1	1	0	0	0	1	0	0	0	0	NA	0	0	0	0
2095	2022	11	F	28	1	1	1	0	1	1	1	0	0	0	1	0	0	0	0	NA	0	0	0	0
2096	2020	1	F	29	0	0	0	0	0	0	0	0	0	0	0	0	0	0	1	NA	0	0	0	0
2097	2020	1	M	29	0	0	0	0	0	0	0	0	0	0	0	0	0	0	0	2	1	1	1	1
2098	2020	1	F	29	0	0	0	0	0	0	0	0	0	0	0	0	0	0	1	NA	0	0	0	0
2099	2020	2	F	29	0	0	0	0	0	0	0	0	0	0	0	0	0	0	1	NA	0	0	0	0
2100	2020	6	M	29	0	0	0	0	0	0	0	0	0	0	1	0	0	0	0	2	1	1	1	1
2101	2020	9	M	29	0	0	0	0	0	0	0	0	0	0	1	1	0	0	0	2	0	0	0	0
2102	2020	9	F	29	0	0	0	0	0	0	0	0	0	0	1	1	0	0	0	2	1	1	1	1
2103	2021	4	F	29	0	0	0	0	0	0	0	0	0	0	1	0	0	0	1	NA	1	1	1	1
2104	2021	7	M	29	0	0	0	0	0	0	0	0	0	0	0	0	0	0	1	NA	0	0	0	0
2105	2021	8	F	29	0	0	0	0	0	0	0	0	0	0	1	0	0	0	0	2	1	1	1	1
2106	2021	9	F	29	0	0	0	0	0	0	0	0	0	0	0	0	0	0	1	2	0	1	1	1
2107	2021	11	F	29	0	0	0	0	0	0	0	0	0	0	1	0	1	0	0	NA	0	0	0	0
2108	2021	12	M	29	0	0	0	0	0	0	0	0	0	0	0	0	0	0	1	NA	0	0	0	0
2109	2020	5	M	29	0	0	0	0	0	0	0	0	0	0	1	1	0	0	0	NA	0	0	0	0
2110	2022	1	F	29	0	0	0	0	0	0	0	0	0	0	0	0	0	0	1	2	0	1	1	1
2111	2022	1	M	29	0	0	0	0	0	0	0	0	0	0	0	0	0	0	1	NA	0	0	0	0
2112	2022	4	M	29	0	0	0	0	0	0	0	0	0	0	0	0	1	0	0	NA	0	0	0	0
2113	2022	4	M	29	0	0	0	0	0	0	0	0	0	0	1	0	1	0	0	2	1	1	1	1
2114	2022	5	M	29	0	0	0	0	0	0	0	0	0	0	1	0	0	0	0	2	1	1	1	1
2115	2022	10	F	29	0	0	0	0	0	0	0	0	0	0	1	0	1	0	0	2	0	1	0	1
2116	2022	12	F	29	0	0	0	0	0	0	0	0	0	0	0	0	1	0	0	NA	0	0	0	0
2117	2022	12	F	29	0	0	0	0	0	0	0	0	0	0	1	0	0	0	1	NA	0	1	1	0
2118	2022	12	F	29	0	0	0	0	0	0	0	0	0	0	0	0	0	0	1	NA	0	0	0	0
2119	2022	12	F	29	0	0	0	0	0	0	0	0	0	0	0	0	0	0	0	2	1	0	1	1
2120	2023	3	F	29	0	0	0	0	0	0	0	0	0	0	1	0	0	0	0	NA	0	0	0	0
2121	2023	6	F	29	0	0	0	0	0	0	0	0	0	0	1	0	0	0	1	2	1	1	1	1
2122	2023	6	F	29	0	0	0	0	0	0	0	0	0	0	1	0	0	0	0	2	0	1	1	1
2123	2023	10	F	29	0	0	0	0	0	0	0	0	0	0	0	0	0	0	1	2	0	0	1	1
2124	2023	11	F	29	0	0	0	0	0	0	0	0	0	0	0	0	0	0	1	NA	0	0	0	0
2125	2024	1	F	29	0	0	0	0	0	0	0	0	0	0	1	0	0	0	0	NA	0	1	0	0
2126	2023	2	F	29	0	0	0	0	0	0	0	0	0	0	0	0	0	0	1	NA	0	0	0	0
2127	2024	4	F	29	0	0	0	0	0	0	0	0	0	0	0	0	0	0	1	NA	0	0	0	0
2128	2024	5	M	29	0	0	0	0	0	0	0	0	0	0	0	0	0	0	1	2	0	1	0	1
2129	2024	5	F	29	0	0	0	0	0	0	0	0	0	0	0	0	0	0	0	2	0	1	1	1
2130	2024	9	F	29	0	0	0	0	0	0	0	0	0	0	1	1	0	0	0	2	0	1	0	0
2131	2024	10	M	29	0	0	0	0	0	0	0	0	0	0	1	0	0	0	0	2	1	1	1	0
2132	2024	10	M	29	0	0	0	0	0	0	0	0	0	0	1	0	0	0	0	NA	0	0	0	0
2133	2024	12	F	29	0	0	0	0	0	0	0	0	0	0	0	0	0	0	0	NA	0	0	0	0
2134	2021	9	M	29	0	0	0	0	0	0	0	0	0	1	0	0	0	0	0	NA	1	1	1	1
2135	2021	11	F	29	0	0	0	0	0	0	0	0	0	1	0	1	0	0	0	NA	0	0	0	0
2136	2020	3	F	29	0	0	0	0	1	0	0	0	0	0	1	0	0	0	0	NA	0	0	0	0
2137	2021	2	F	29	1	0	0	0	0	0	0	0	0	0	1	0	0	0	0	NA	1	1	1	1
2138	2021	9	F	29	0	0	1	0	0	0	0	0	0	0	1	1	0	0	0	NA	0	0	0	0
2139	2021	11	M	29	0	0	1	0	0	0	0	0	0	0	1	1	0	0	0	2	0	1	1	0
2140	2022	11	M	29	0	0	1	0	0	0	0	0	0	0	1	0	1	0	0	NA	0	1	1	0
2141	2022	11	M	29	0	0	1	0	0	0	0	0	0	0	1	0	0	0	0	NA	0	0	0	0
2142	2023	5	F	29	0	0	1	0	0	0	0	0	0	0	1	0	0	0	0	NA	0	1	1	1

ID	Yr	Mo	Sex	Age	Tre	Grs	Rag	Mug	HDM	Dan	Mld	Food	Drg	Ven	Rhi	Cnj	Ast	AsE	Urt	Sev	Prt	Obs	Rho	Snz
2143	2024	5	M	29	0	0	1	0	0	0	0	0	0	0	1	1	0	0	0	2	1	1	0	1
2144	2024	8	F	29	0	0	1	0	0	0	0	0	0	0	1	1	1	0	0	2	0	1	1	1
2145	2024	9	M	29	0	0	1	0	0	0	0	0	0	0	1	1	1	0	0	2	1	1	1	1
2146	2022	6	M	29	0	1	0	0	0	0	0	0	0	0	1	0	0	0	0	2	1	0	1	0
2147	2024	10	M	29	0	0	0	0	1	0	0	0	0	0	1	1	1	0	0	NA	0	0	0	0
2148	2024	11	F	29	0	0	0	0	1	0	0	0	0	0	1	1	0	0	0	NA	0	0	0	0
2149	2021	10	F	29	0	0	0	0	1	0	0	0	1	0	1	0	1	0	0	NA	0	0	0	0
2150	2021	5	F	29	0	1	0	0	0	0	0	0	0	1	1	0	0	0	0	NA	0	0	0	0
2151	2020	2	M	29	0	0	0	1	0	0	1	0	0	0	1	0	1	1	0	1	0	1	0	0
2152	2021	10	M	29	1	1	0	0	0	0	0	0	0	0	1	1	0	0	0	1	0	1	0	0
2153	2020	3	M	29	0	0	1	0	0	0	1	0	0	0	1	1	0	0	0	NA	0	0	0	0
2154	2020	3	M	29	0	0	1	1	0	0	0	0	0	0	1	1	0	0	0	NA	0	0	0	0
2155	2022	2	M	29	0	0	1	0	1	0	0	0	0	0	1	1	0	0	0	2	1	1	1	1
2156	2022	4	M	29	0	0	1	0	1	0	0	0	0	0	1	0	0	0	0	NA	0	0	0	0
2157	2022	5	M	29	1	0	1	0	0	0	0	0	0	0	1	1	0	0	0	NA	0	0	0	0
2158	2023	2	F	29	0	1	1	0	0	0	0	0	0	0	1	0	0	0	0	NA	0	0	0	0
2159	2023	8	M	29	0	1	1	0	0	0	0	0	0	0	1	1	0	0	0	2	0	1	1	0
2160	2023	8	M	29	0	1	1	0	0	0	0	0	0	0	1	0	0	0	0	NA	0	0	0	0
2161	2024	7	F	29	0	1	1	0	0	0	0	0	0	0	1	1	1	0	0	2	0	1	1	0
2162	2024	9	M	29	0	1	1	0	0	0	0	0	0	0	1	1	0	0	0	NA	0	0	0	0
2163	2020	8	F	29	0	0	0	0	1	0	1	0	0	1	1	1	1	0	0	2	0	1	1	0
2164	2021	9	M	29	0	1	1	0	0	0	0	0	0	1	1	1	0	0	0	2	0	1	1	1
2165	2021	6	M	29	1	0	1	0	0	0	0	0	0	1	1	0	0	0	0	2	0	0	0	0
2166	2023	5	F	29	0	1	1	0	1	0	0	0	0	0	1	0	0	0	0	NA	0	0	0	0
2167	2023	5	M	29	0	1	1	0	0	1	0	0	0	0	1	1	1	0	1	1	0	1	0	0
2168	2022	9	F	29	0	1	0	0	0	1	1	0	0	0	1	1	0	0	0	NA	0	0	0	0
2169	2024	5	M	29	1	1	0	0	0	0	1	0	0	0	1	1	0	0	0	1	0	1	1	0
2170	2021	5	M	29	1	1	0	1	0	0	0	0	0	1	1	0	0	0	0	NA	0	0	0	0
2171	2021	5	F	29	1	1	0	1	0	0	0	0	0	1	1	0	0	0	0	NA	0	0	0	0
2172	2023	3	F	29	0	1	0	0	1	0	1	0	1	0	1	1	0	0	0	NA	0	0	0	0
2173	2021	1	M	29	0	1	0	1	1	1	0	0	0	0	1	1	0	0	0	1	0	1	0	0
2174	2020	12	F	29	1	1	1	0	0	1	0	0	0	0	1	0	0	0	0	NA	0	0	0	0
2175	2021	11	M	29	1	1	1	0	0	1	0	0	0	1	1	1	0	0	0	2	1	1	1	1
2176	2020	1	M	30	0	0	0	0	0	0	0	0	0	0	1	0	0	0	1	NA	0	0	0	0
2177	2020	1	F	30	0	0	0	0	0	0	0	0	0	0	0	0	0	0	1	2	0	1	0	1
2178	2020	2	M	30	0	0	0	0	0	0	0	0	0	0	0	0	1	0	0	2	1	1	0	1
2179	2020	2	F	30	0	0	0	0	0	0	0	0	0	0	0	0	0	0	0	NA	0	0	0	0
2180	2020	2	F	30	0	0	0	0	0	0	0	0	0	0	0	0	0	0	1	NA	0	0	0	0
2181	2020	3	M	30	0	0	0	0	0	0	0	0	0	0	1	1	0	0	0	NA	0	0	0	0
2182	2020	6	F	30	0	0	0	0	0	0	0	0	0	0	1	1	1	0	0	NA	0	0	0	0
2183	2020	6	M	30	0	0	0	0	0	0	0	0	0	0	1	0	0	0	0	NA	0	0	0	0
2184	2020	6	F	30	0	0	0	0	0	0	0	0	0	0	0	0	0	0	0	2	1	1	1	1
2185	2020	7	F	30	0	0	0	0	0	0	0	0	0	0	0	0	0	0	0	2	0	1	0	1
2186	2020	8	M	30	0	0	0	0	0	0	0	0	0	0	1	1	1	0	0	2	1	1	0	0
2187	2020	10	M	30	0	0	0	0	0	0	0	0	0	0	1	0	0	0	0	NA	0	0	0	0
2188	2020	11	M	30	0	0	0	0	0	0	0	0	0	0	1	0	1	0	0	2	0	0	0	0
2189	2021	2	M	30	0	0	0	0	0	0	0	0	0	0	1	0	0	0	0	2	1	1	1	1
2190	2021	6	M	30	0	0	0	0	0	0	0	0	0	0	1	1	0	0	0	2	1	1	1	1
2191	2021	7	M	30	0	0	0	0	0	0	0	0	0	0	0	0	0	0	0	2	1	1	0	0
2192	2021	8	F	30	0	0	0	0	0	0	0	0	0	0	0	0	0	0	1	NA	0	0	0	0
2193	2021	9	M	30	0	0	0	0	0	0	0	0	0	0	0	0	0	0	1	NA	0	0	0	0
2194	2021	5	F	30	0	0	0	0	0	0	0	0	0	0	0	0	0	1	0	NA	0	1	1	0
2195	2022	2	F	30	0	0	0	0	0	0	0	0	0	0	0	0	0	0	1	2	0	1	0	0
2196	2022	2	F	30	0	0	0	0	0	0	0	0	0	0	1	0	1	0	0	NA	0	0	0	0
2197	2022	2	F	30	0	0	0	0	0	0	0	0	0	0	0	0	0	0	1	NA	0	0	0	0
2198	2022	2	M	30	0	0	0	0	0	0	0	0	0	0	1	1	0	0	0	NA	0	0	0	0
2199	2022	3	F	30	0	0	0	0	0	0	0	0	0	0	0	0	0	0	1	2	1	1	1	1
2200	2022	3	M	30	0	0	0	0	0	0	0	0	0	0	0	0	0	0	0	NA	0	0	0	0
2201	2022	4	M	30	0	0	0	0	0	0	0	0	0	0	0	0	0	0	1	NA	0	0	0	0
2202	2022	9	F	30	0	0	0	0	0	0	0	0	0	0	1	0	0	0	0	1	0	1	0	0
2203	2022	10	F	30	0	0	0	0	0	0	0	0	0	0	0	0	0	0	0	NA	0	0	0	0
2204	2022	11	F	30	0	0	0	0	0	0	0	0	0	0	0	0	0	0	1	2	0	1	1	0
2205	2022	11	M	30	0	0	0	0	0	0	0	0	0	0	1	0	0	0	0	NA	0	0	0	0

ID	Yr	Mo	Sex	Age	Tre	Grs	Rag	Mug	HDM	Dan	Mld	Food	Drg	Ven	Rhi	Cnj	Ast	AsE	Urt	Sev	Prt	Obs	Rho	Snz
2206	2022	11	F	30	0	0	0	0	0	0	0	0	0	0	1	0	0	0	0	2	1	1	1	1
2207	2023	3	F	30	0	0	0	0	0	0	0	0	0	0	0	0	0	0	1	NA	0	0	0	0
2208	2023	6	M	30	0	0	0	0	0	0	0	0	0	0	1	0	0	0	1	2	0	1	0	0
2209	2023	6	M	30	0	0	0	0	0	0	0	0	0	0	0	0	0	0	1	2	1	1	1	0
2210	2023	6	M	30	0	0	0	0	0	0	0	0	0	0	1	0	0	0	0	NA	0	0	0	0
2211	2023	11	F	30	0	0	0	0	0	0	0	0	0	0	1	0	0	0	0	NA	0	0	0	0
2212	2024	2	M	30	0	0	0	0	0	0	0	0	0	0	1	0	1	1	0	2	1	1	1	1
2213	2024	3	M	30	0	0	0	0	0	0	0	0	0	0	0	0	0	0	0	NA	0	0	0	0
2214	2024	3	M	30	0	0	0	0	0	0	0	0	0	0	0	0	1	0	0	NA	0	0	0	0
2215	2024	3	F	30	0	0	0	0	0	0	0	0	0	0	0	0	0	0	0	2	0	1	0	1
2216	2024	4	F	30	0	0	0	0	0	0	0	0	0	0	0	0	0	0	0	2	0	0	1	1
2217	2024	4	M	30	0	0	0	0	0	0	0	0	0	0	0	0	0	0	0	2	1	1	1	1
2218	2024	8	F	30	0	0	0	0	0	0	0	0	0	0	0	0	0	0	1	NA	0	0	0	0
2219	2024	8	F	30	0	0	0	0	0	0	0	0	0	0	0	0	0	0	0	NA	0	0	0	0
2220	2024	9	F	30	0	0	0	0	0	0	0	0	0	0	1	1	0	0	0	NA	0	0	0	0
2221	2024	12	M	30	0	0	0	0	0	0	0	0	0	0	0	0	0	0	1	NA	0	0	0	0
2222	2020	9	M	30	0	0	0	0	0	0	0	0	0	1	1	0	0	0	0	2	1	1	1	0
2223	2020	10	F	30	0	0	0	0	0	0	0	0	1	0	1	0	1	0	0	2	0	1	0	0
2224	2021	10	F	30	0	0	0	0	0	0	0	0	1	0	0	0	0	0	0	NA	0	0	0	0
2225	2021	11	M	30	0	0	0	0	0	0	0	0	0	1	0	1	0	0	0	NA	0	0	0	0
2226	2023	4	F	30	0	0	0	0	0	0	0	1	0	0	0	0	0	0	1	NA	0	0	0	0
2227	2023	11	M	30	0	0	0	0	0	0	0	0	1	0	0	0	0	0	1	NA	0	0	0	0
2228	2024	3	M	30	0	0	0	0	0	0	0	0	1	0	0	0	0	0	1	NA	0	0	0	0
2229	2024	7	F	30	0	0	0	0	0	0	0	0	1	0	0	0	0	0	0	2	1	1	1	1
2230	2020	2	F	30	0	0	0	0	1	0	0	0	0	0	1	0	1	0	0	2	0	1	1	0
2231	2020	5	F	30	0	0	0	0	1	0	0	0	0	0	1	0	1	0	0	1	0	1	0	0
2232	2021	4	M	30	0	1	0	0	0	0	0	0	0	0	1	1	0	0	1	NA	0	0	0	0
2233	2021	6	M	30	0	1	0	0	0	0	0	0	0	0	1	1	0	0	0	2	0	1	1	1
2234	2020	9	F	30	0	0	1	0	0	0	0	0	0	0	1	1	0	0	0	NA	0	0	0	0
2235	2020	9	M	30	0	0	1	0	0	0	0	0	0	0	0	0	0	0	0	NA	0	0	0	0
2236	2022	8	M	30	0	0	1	0	0	0	0	0	0	0	1	1	1	0	0	2	1	1	1	1
2237	2023	9	M	30	0	0	1	0	0	0	0	0	0	0	1	0	0	0	0	NA	0	0	1	0
2238	2023	11	F	30	0	0	1	0	0	0	0	0	0	0	1	0	1	0	1	NA	0	0	0	0
2239	2024	9	M	30	0	0	1	0	0	0	0	0	0	0	1	1	0	0	0	2	1	1	0	0
2240	2024	11	F	30	0	0	1	0	0	0	0	0	0	0	1	1	0	0	0	NA	0	0	0	0
2241	2022	9	F	30	0	0	0	1	0	0	0	0	0	0	1	1	0	0	0	NA	0	0	0	0
2242	2021	9	F	30	0	0	0	0	0	1	0	0	0	1	1	1	0	0	0	NA	0	0	0	0
2243	2021	5	M	30	0	1	0	0	0	0	0	0	0	1	1	0	0	0	0	NA	0	0	0	0
2244	2020	1	F	30	0	0	0	0	1	1	0	0	0	0	1	0	0	0	0	NA	0	0	0	0
2245	2020	9	M	30	0	1	0	0	1	0	0	0	0	0	1	0	1	0	0	NA	0	0	0	0
2246	2020	1	M	30	0	0	1	1	0	0	0	0	0	0	1	1	0	0	0	NA	0	0	0	0
2247	2020	2	M	30	0	0	1	0	1	0	0	0	0	0	1	1	0	0	0	NA	0	0	0	0
2248	2023	11	M	30	0	1	1	0	0	0	0	0	0	0	1	0	1	0	0	NA	0	0	0	0
2249	2021	3	F	30	0	0	1	0	1	0	0	0	0	0	1	1	0	0	0	2	0	1	0	0
2250	2022	9	F	30	0	1	0	0	1	0	0	0	0	0	1	1	1	0	1	2	1	1	1	1
2251	2022	3	F	30	0	0	1	0	1	0	0	0	1	0	1	1	1	0	1	2	1	1	1	1
2252	2020	8	F	30	0	1	1	0	1	0	0	0	0	0	1	1	1	0	0	2	0	1	0	0
2253	2022	5	M	30	0	0	1	0	1	1	0	0	0	0	1	1	1	0	0	NA	0	0	0	0
2254	2022	5	F	30	0	1	1	0	0	1	0	0	0	0	1	1	0	0	0	2	0	0	1	1
2255	2023	5	M	30	0	0	1	1	1	0	0	0	0	0	1	1	0	0	0	2	1	1	1	1
2256	2024	1	F	30	0	1	1	1	0	0	0	0	0	0	1	0	1	0	0	2	0	1	0	0
2257	2024	7	M	30	0	1	1	0	1	0	0	0	0	0	1	0	1	0	0	NA	0	0	0	0
2258	2024	9	M	30	0	1	1	1	0	0	0	0	0	0	1	0	1	0	0	1	0	1	0	0
2259	2024	10	F	30	0	1	1	0	1	0	0	0	0	0	1	1	0	0	0	2	0	1	1	0
2260	2024	11	M	30	0	0	1	0	0	1	1	0	0	0	1	1	0	0	0	2	0	0	1	1
2261	2022	4	M	30	1	1	0	0	1	0	0	0	0	0	1	1	0	0	0	NA	0	0	0	0
2262	2024	8	F	30	0	1	0	0	1	1	0	0	1	0	0	1	1	2	0	NA	0	0	0	0
2263	2020	8	M	30	1	1	1	0	0	1	0	0	0	0	1	1	1	0	0	2	0	1	0	0
2264	2022	9	M	30	1	1	1	1	0	0	0	0	0	0	1	1	0	0	0	2	1	1	1	1
2265	2024	4	M	30	0	0	1	0	1	1	1	0	0	0	1	0	1	0	0	NA	0	0	0	0
2266	2024	8	F	30	0	1	1	1	1	0	0	0	0	0	1	0	0	0	0	2	1	1	1	1
2267	2022	4	F	30	1	1	0	0	1	1	0	0	0	0	1	1	0	0	0	NA	0	0	0	0
2268	2020	12	M	30	1	1	1	0	1	1	0	0	0	0	1	1	0	0	0	2	1	0	1	1

ID	Yr	Mo	Sex	Age	Tre	Grs	Rag	Mug	HDM	Dan	Mld	Food	Drg	Ven	Rhi	Cnj	Ast	AsE	Urt	Sev	Prt	Obs	Rho	Snz
2269	2020	1	F	31	0	0	0	0	0	0	0	0	0	0	1	0	0	0	0	2	1	1	1	1
2270	2020	2	M	31	0	0	0	0	0	0	0	0	0	0	0	0	0	0	0	2	1	1	1	1
2271	2020	2	F	31	0	0	0	0	0	0	0	0	0	0	1	0	0	0	1	2	0	1	1	0
2272	2020	3	M	31	0	0	0	0	0	0	0	0	0	0	0	0	0	0	1	NA	0	1	0	0
2273	2020	9	M	31	0	0	0	0	0	0	0	0	0	0	1	0	1	0	0	1	1	1	1	1
2274	2020	9	F	31	0	0	0	0	0	0	0	0	0	0	1	0	1	0	1	NA	0	0	0	0
2275	2020	9	F	31	0	0	0	0	0	0	0	0	0	0	1	0	0	0	0	2	0	1	1	1
2276	2020	11	F	31	0	0	0	0	0	0	0	0	0	0	1	0	0	0	0	NA	0	0	0	0
2277	2021	1	M	31	0	0	0	0	0	0	0	0	0	0	0	0	0	0	1	NA	0	0	0	0
2278	2021	1	F	31	0	0	0	0	0	0	0	0	0	0	0	0	0	0	0	2	0	0	1	1
2279	2021	5	F	31	0	0	0	0	0	0	0	0	0	0	0	0	0	0	1	2	0	1	1	1
2280	2021	9	M	31	0	0	0	0	0	0	0	0	0	0	1	0	0	0	0	NA	0	0	0	0
2281	2021	3	F	31	0	0	0	0	0	0	0	0	0	0	1	0	0	0	0	1	0	1	0	0
2282	2021	5	F	31	0	0	0	0	0	0	0	0	0	0	0	0	0	0	1	2	1	0	1	1
2283	2021	10	F	31	0	0	0	0	0	0	0	0	0	0	0	0	0	0	0	NA	0	0	0	0
2284	2022	1	M	31	0	0	0	0	0	0	0	0	0	0	0	0	0	0	0	1	0	1	0	0
2285	2022	1	M	31	0	0	0	0	0	0	0	0	0	0	0	0	0	0	0	NA	0	0	0	0
2286	2022	1	F	31	0	0	0	0	0	0	0	0	0	0	1	0	1	0	0	1	0	1	0	0
2287	2022	1	F	31	0	0	0	0	0	0	0	0	0	0	0	0	0	0	0	NA	0	0	0	0
2288	2022	5	F	31	0	0	0	0	0	0	0	0	0	0	0	0	0	0	1	NA	0	0	0	0
2289	2022	9	F	31	0	0	0	0	0	0	0	0	0	0	1	1	0	0	0	NA	0	0	0	0
2290	2022	10	F	31	0	0	0	0	0	0	0	0	0	0	0	0	0	0	1	2	1	1	1	1
2291	2022	11	F	31	0	0	0	0	0	0	0	0	0	0	0	0	0	0	1	NA	0	0	0	0
2292	2023	1	F	31	0	0	0	0	0	0	0	0	0	0	0	0	0	0	1	NA	0	0	0	0
2293	2023	2	F	31	0	0	0	0	0	0	0	0	0	0	0	0	1	0	0	NA	0	0	0	0
2294	2023	3	M	31	0	0	0	0	0	0	0	0	0	0	0	0	0	0	1	NA	0	0	0	0
2295	2023	3	F	31	0	0	0	0	0	0	0	0	0	0	1	0	0	0	0	NA	0	0	0	0
2296	2023	3	M	31	0	0	0	0	0	0	0	0	0	0	1	0	0	0	0	NA	0	0	0	0
2297	2023	5	F	31	0	0	0	0	0	0	0	0	0	0	1	0	0	0	0	NA	0	0	0	0
2298	2023	6	M	31	0	0	0	0	0	0	0	0	0	0	0	0	0	0	1	2	0	0	1	1
2299	2023	6	F	31	0	0	0	0	0	0	0	0	0	0	0	0	0	0	1	2	1	1	1	1
2300	2023	8	M	31	0	0	0	0	0	0	0	0	0	0	0	0	0	0	1	NA	0	0	1	1
2301	2023	8	F	31	0	0	0	0	0	0	0	0	0	0	0	0	0	0	1	2	0	0	1	0
2302	2024	2	F	31	0	0	0	0	0	0	0	0	0	0	0	0	0	0	0	2	0	0	0	0
2303	2024	3	F	31	0	0	0	0	0	0	0	0	0	0	1	1	0	0	0	NA	0	0	0	0
2304	2024	3	M	31	0	0	0	0	0	0	0	0	0	0	0	0	0	0	0	2	0	0	0	0
2305	2024	8	F	31	0	0	0	0	0	0	0	0	0	0	1	1	0	0	0	NA	0	0	0	0
2306	2024	9	F	31	0	0	0	0	0	0	0	0	0	0	0	0	0	0	1	NA	0	0	0	0
2307	2024	10	M	31	0	0	0	0	0	0	0	0	0	0	0	0	0	0	1	NA	0	0	0	0
2308	2021	10	F	31	0	0	0	0	0	0	0	0	0	1	1	1	0	0	0	1	1	0	0	1
2309	2024	9	M	31	0	0	0	0	0	0	0	0	0	1	1	0	0	0	0	2	1	1	1	1
2310	2020	9	F	31	0	0	0	0	0	0	1	0	0	0	0	0	1	0	0	1	0	1	0	1
2311	2020	11	M	31	0	0	0	0	0	0	1	0	0	0	1	0	1	0	0	NA	0	0	0	0
2312	2020	1	F	31	0	0	1	0	0	0	0	0	0	0	1	1	0	0	0	1	0	1	0	0
2313	2020	2	M	31	0	0	1	0	0	0	0	0	0	0	1	1	0	0	0	NA	0	0	0	0
2314	2020	7	M	31	0	0	1	0	0	0	0	0	0	0	1	0	0	0	0	NA	0	0	0	0
2315	2020	9	F	31	0	0	1	0	0	0	0	0	0	0	1	0	0	0	0	2	0	1	0	0
2316	2022	7	M	31	0	0	1	0	0	0	0	0	0	0	1	0	0	0	0	NA	0	0	0	0
2317	2022	7	M	31	0	0	1	0	0	0	0	0	0	0	1	0	1	0	0	NA	0	0	0	0
2318	2022	8	F	31	0	0	1	0	0	0	0	0	0	0	1	1	0	0	1	1	0	0	0	0
2319	2022	9	M	31	0	0	1	0	0	0	0	0	0	0	1	1	1	0	0	1	0	0	1	0
2320	2023	1	F	31	0	0	1	0	0	0	0	0	0	0	1	0	0	0	0	1	1	0	0	0
2321	2024	9	F	31	0	0	1	0	0	0	0	0	0	0	1	1	0	0	0	2	0	0	0	0
2322	2024	9	M	31	0	0	1	0	0	0	0	0	0	0	1	1	0	0	0	2	0	1	0	0
2323	2022	9	F	31	0	0	0	0	0	1	0	0	0	0	1	1	0	0	0	NA	0	0	0	0
2324	2023	2	M	31	0	0	0	0	1	0	0	0	0	0	1	0	0	0	1	2	0	1	1	0
2325	2023	5	M	31	0	0	0	0	1	0	0	0	0	0	1	1	0	0	0	2	0	1	1	0
2326	2023	8	F	31	0	0	0	0	0	1	0	0	0	0	1	0	0	0	0	2	1	1	1	1
2327	2021	5	M	31	0	1	0	0	0	0	0	0	0	1	1	0	0	0	0	2	1	1	1	1
2328	2021	12	M	31	0	0	0	0	1	0	0	0	0	1	0	1	0	0	0	2	1	1	1	1
2329	2021	10	F	31	0	0	1	0	0	0	0	0	0	1	1	0	0	0	0	NA	2	0	0	0
2330	2021	8	F	31	0	0	1	0	0	0	0	0	0	1	1	0	0	0	0	2	1	1	1	1
2331	2020	12	M	31	0	0	0	0	1	0	1	0	0	0	1	0	1	0	0	1	1	1	0	0

ID	Yr	Mo	Sex	Age	Tre	Grs	Rag	Mug	HDM	Dan	Mld	Food	Drg	Ven	Rhi	Cnj	Ast	AsE	Urt	Sev	Prt	Obs	Rho	Snz
2332	2021	11	F	31	0	0	0	0	0	1	1	0	0	0	1	0	1	0	0	1	0	0	0	0
2333	2020	1	M	31	0	1	1	0	0	0	0	0	0	0	1	0	0	0	0	NA	0	0	0	0
2334	2020	2	F	31	0	1	1	0	0	0	0	0	0	0	1	1	1	0	0	1	0	1	1	0
2335	2020	3	M	31	0	0	1	0	0	1	0	0	0	0	1	1	0	0	0	2	0	0	0	0
2336	2020	9	M	31	0	0	1	1	0	0	0	0	0	0	1	1	0	0	0	NA	0	0	0	0
2337	2021	10	M	31	0	0	1	0	0	1	0	0	0	0	1	1	1	0	0	NA	1	0	1	1
2338	2023	3	M	31	1	0	1	0	0	0	0	0	0	0	1	0	0	0	0	NA	0	0	0	0
2339	2023	6	M	31	0	0	1	0	1	0	0	0	0	0	1	1	0	0	0	2	0	1	0	0
2340	2023	8	F	31	0	0	1	0	1	0	0	0	0	0	1	1	1	0	0	1	0	0	0	0
2341	2024	7	M	31	0	0	1	1	0	0	0	0	0	0	1	0	0	0	0	2	0	0	0	0
2342	2023	6	M	31	0	1	0	0	0	1	0	0	0	0	1	0	0	0	0	3	0	0	0	0
2343	2024	5	M	31	0	0	0	1	1	0	0	0	0	0	1	1	0	0	0	2	0	0	1	1
2344	2021	9	F	31	0	0	1	0	0	1	0	0	0	1	1	0	0	0	0	2	0	1	0	0
2345	2021	9	M	31	0	1	0	0	1	1	0	0	0	0	0	1	0	0	0	1	0	0	1	1
2346	2020	8	F	31	0	1	1	0	0	0	1	0	0	0	1	1	0	0	0	NA	0	0	0	0
2347	2023	2	F	31	0	1	1	0	0	1	0	0	0	0	1	0	0	0	0	2	0	1	1	1
2348	2023	8	F	31	0	0	1	1	0	1	0	0	0	0	1	0	0	0	1	NA	0	0	0	0
2349	2024	9	M	31	1	1	1	0	0	0	0	0	0	0	1	1	0	0	0	4	0	1	1	0
2350	2020	10	F	31	1	1	1	1	0	0	0	0	0	0	1	0	0	0	0	1	0	0	1	1
2351	2024	2	F	31	1	1	1	1	0	0	0	0	0	0	1	1	0	0	0	NA	0	0	0	0
2352	2024	10	M	31	0	0	1	0	1	1	1	0	0	0	1	1	1	0	0	NA	0	0	0	0
2353	2023	6	M	31	1	1	1	0	1	1	1	0	0	0	1	0	0	0	0	2	1	1	0	1
2354	2021	3	F	31	1	1	1	1	1	1	0	0	0	0	1	1	0	0	0	NA	0	0	0	0
2355	2020	2	F	32	0	0	0	0	0	0	0	0	0	0	0	0	0	0	1	2	0	1	0	1
2356	2020	2	M	32	0	0	0	0	0	0	0	0	0	0	0	0	0	0	1	1	0	1	0	0
2357	2020	9	F	32	0	0	0	0	0	0	0	0	0	0	1	0	0	0	0	NA	0	0	0	0
2358	2020	11	F	32	0	0	0	0	0	0	0	0	0	0	0	0	0	0	1	2	0	1	1	1
2359	2020	12	F	32	0	0	0	0	0	0	0	0	0	0	0	0	0	0	1	3	0	1	0	0
2360	2021	2	F	32	0	0	0	0	0	0	0	0	0	0	0	0	0	0	1	2	0	1	1	0
2361	2021	5	F	32	0	0	0	0	0	0	0	0	0	0	0	0	0	0	1	2	0	0	0	0
2362	2021	9	F	32	0	0	0	0	0	0	0	0	0	0	0	0	0	1	0	3	0	0	0	0
2363	2021	9	M	32	0	0	0	0	0	0	0	0	0	0	1	1	0	0	0	1	0	0	1	1
2364	2021	9	F	32	0	0	0	0	0	0	0	0	0	0	0	0	0	0	0	1	1	1	1	0
2365	2021	10	F	32	0	0	0	0	0	0	0	0	0	0	0	0	0	0	1	1	0	1	0	0
2366	2021	2	M	32	0	0	0	0	0	0	0	0	0	0	0	0	1	0	1	2	0	1	0	0
2367	2021	1	F	32	0	0	0	0	0	0	0	0	0	0	1	0	0	0	0	NA	0	0	0	0
2368	2021	2	F	32	0	0	0	0	0	0	0	0	0	0	0	0	0	0	0	NA	0	0	0	0
2369	2021	10	F	32	0	0	0	0	0	0	0	0	0	0	0	0	0	1	0	NA	0	0	0	0
2370	2021	10	F	32	0	0	0	0	0	0	0	0	0	0	0	0	0	0	0	4	1	1	0	0
2371	2022	1	F	32	0	0	0	0	0	0	0	0	0	0	1	0	0	0	0	NA	0	0	0	0
2372	2022	1	F	32	0	0	0	0	0	0	0	0	0	0	0	0	0	0	1	NA	0	0	0	0
2373	2022	3	M	32	0	0	0	0	0	0	0	0	0	0	1	1	0	0	0	NA	0	0	0	0
2374	2022	5	M	32	0	0	0	0	0	0	0	0	0	0	1	1	0	0	0	NA	0	0	0	0
2375	2022	7	M	32	0	0	0	0	0	0	0	0	0	0	0	0	0	0	0	NA	0	0	0	0
2376	2022	9	M	32	0	0	0	0	0	0	0	0	0	0	1	1	0	0	0	2	1	1	1	1
2377	2022	11	F	32	0	0	0	0	0	0	0	0	0	0	0	0	0	0	1	NA	0	0	0	0
2378	2023	2	F	32	0	0	0	0	0	0	0	0	0	0	1	0	0	0	1	2	1	1	1	0
2379	2023	4	M	32	0	0	0	0	0	0	0	0	0	0	0	0	0	0	1	1	0	1	0	0
2380	2023	12	F	32	0	0	0	0	0	0	0	0	0	0	1	0	0	0	0	3	0	1	0	0
2381	2024	2	F	32	0	0	0	0	0	0	0	0	0	0	0	0	1	0	0	1	0	0	0	0
2382	2024	3	F	32	0	0	0	0	0	0	0	0	0	0	0	0	0	0	0	4	1	0	0	0
2383	2024	3	F	32	0	0	0	0	0	0	0	0	0	0	0	0	0	0	0	3	0	1	1	1
2384	2024	4	F	32	0	0	0	0	0	0	0	0	0	0	0	0	1	0	0	4	1	1	1	0
2385	2024	10	F	32	0	0	0	0	0	0	0	0	0	0	0	0	0	0	1	1	0	0	1	0
2386	2021	11	F	32	0	0	0	0	0	0	0	1	0	0	0	0	0	0	0	2	1	0	0	0
2387	2021	6	M	32	0	0	0	0	0	0	0	0	0	1	0	1	0	0	0	NA	0	0	0	0
2388	2021	9	M	32	0	0	0	0	0	0	0	0	0	1	1	0	0	0	0	2	1	1	1	1
2389	2021	5	F	32	0	0	0	0	0	0	0	0	0	1	0	0	0	0	0	2	0	1	1	1
2390	2022	2	M	32	0	0	0	0	0	0	0	0	0	1	0	0	0	0	1	NA	0	0	0	0
2391	2022	6	F	32	0	0	0	0	0	0	0	1	0	0	0	0	0	0	0	1	0	1	1	1
2392	2023	5	F	32	0	0	0	0	0	0	0	0	1	0	1	0	1	0	0	4	0	1	1	1
2393	2023	5	F	32	0	0	0	0	0	0	0	0	1	0	0	0	0	0	1	1	0	1	1	1
2394	2024	4	M	32	0	0	0	0	0	0	0	0	1	0	0	0	0	0	0	1	0	1	0	1

ID	Yr	Mo	Sex	Age	Tre	Grs	Rag	Mug	HDM	Dan	Mld	Food	Drg	Ven	Rhi	Cnj	Ast	AsE	Urt	Sev	Prt	Obs	Rho	Snz
2395	2021	4	F	32	0	0	0	0	0	0	0	1	0	1	1	0	0	1	1	2	0	0	0	1
2396	2020	7	M	32	0	0	0	1	0	0	0	0	0	0	1	1	0	0	0	NA	0	0	0	0
2397	2020	10	M	32	0	0	0	0	1	0	0	0	0	0	1	0	1	0	0	NA	0	1	0	0
2398	2020	12	M	32	0	0	0	0	1	0	0	0	0	0	1	0	1	0	0	NA	0	0	0	0
2399	2021	4	F	32	0	0	0	0	1	0	0	0	0	0	0	0	0	0	1	NA	0	0	0	0
2400	2021	3	F	32	0	0	0	0	1	0	0	0	0	0	0	0	1	0	1	NA	0	0	0	0
2401	2020	9	F	32	0	0	1	0	0	0	0	0	0	0	1	1	0	0	0	2	0	0	0	0
2402	2021	9	M	32	0	0	1	0	0	0	0	0	0	0	1	1	0	0	0	3	1	1	0	1
2403	2022	2	M	32	0	0	1	0	0	0	0	0	0	0	1	0	0	0	0	2	1	1	1	1
2404	2022	3	M	32	0	0	1	0	0	0	0	0	0	0	1	1	0	0	0	NA	0	0	0	0
2405	2022	5	M	32	0	0	1	0	0	0	0	0	0	0	1	1	0	0	0	2	0	1	1	0
2406	2022	8	F	32	0	0	1	0	0	0	0	0	0	0	1	0	0	0	0	4	0	1	0	1
2407	2022	11	F	32	0	0	1	0	0	0	0	0	0	0	1	0	0	0	1	3	0	0	1	0
2408	2023	3	M	32	0	0	1	0	0	0	0	0	0	0	1	0	0	0	0	1	1	1	0	0
2409	2023	5	M	32	0	0	1	0	0	0	0	0	0	0	1	1	0	0	0	2	1	1	1	1
2410	2023	10	M	32	0	0	1	0	0	0	0	0	0	0	1	1	0	0	0	2	1	1	1	1
2411	2024	8	F	32	0	0	1	0	0	0	0	0	0	0	1	1	1	0	0	2	1	0	1	1
2412	2024	8	F	32	0	0	1	0	0	0	0	0	0	0	1	1	0	0	0	NA	0	0	0	0
2413	2024	8	M	32	0	0	1	0	0	0	0	0	0	0	1	1	0	0	0	3	0	1	0	0
2414	2024	8	F	32	0	0	1	0	0	0	0	0	0	0	1	0	0	0	0	NA	0	0	0	0
2415	2024	9	M	32	0	0	1	0	0	0	0	0	0	0	1	1	0	0	0	2	1	1	1	1
2416	2024	11	F	32	0	0	1	0	0	0	0	0	0	0	1	1	0	0	0	2	0	1	1	1
2417	2022	4	F	32	0	1	0	0	0	0	0	0	0	0	1	1	0	0	0	1	1	0	0	0
2418	2022	4	M	32	0	0	0	0	1	0	0	0	0	0	1	0	1	0	0	4	0	0	0	0
2419	2022	6	M	32	0	1	0	0	0	0	0	0	0	0	1	1	1	0	0	2	0	1	0	0
2420	2022	7	M	32	0	1	0	0	0	0	0	0	0	0	1	1	0	0	0	NA	1	0	0	0
2421	2022	9	F	32	0	0	0	0	0	0	1	0	0	0	1	0	1	0	0	4	1	1	1	0
2422	2024	5	F	32	0	0	0	0	1	0	0	0	0	0	1	0	0	0	0	2	0	0	0	1
2423	2024	8	M	32	0	1	0	0	0	0	0	0	0	0	1	1	0	0	0	2	1	1	1	1
2424	2021	11	M	32	0	0	0	0	1	0	0	0	0	1	0	0	0	1	0	2	1	1	1	1
2425	2021	12	F	32	0	0	1	0	0	0	0	0	0	1	0	0	0	0	0	NA	0	0	0	0
2426	2022	2	M	32	0	0	0	0	1	0	0	0	1	0	1	1	0	0	0	2	0	1	1	1
2427	2022	11	M	32	0	0	0	0	0	0	1	1	0	0	1	0	0	0	0	NA	0	0	0	0
2428	2020	6	M	32	0	0	1	0	0	1	0	0	0	0	1	1	0	0	0	3	1	0	0	0
2429	2020	7	M	32	0	0	1	0	1	0	0	0	0	0	1	1	0	0	0	2	1	0	0	0
2430	2021	2	M	32	0	0	1	0	0	1	0	0	0	0	1	1	0	0	0	3	1	0	0	0
2431	2021	12	F	32	0	0	1	0	0	1	0	0	0	0	1	1	0	0	0	2	0	1	0	0
2432	2022	2	M	32	0	0	1	0	1	0	0	0	0	0	1	1	0	0	0	NA	0	0	0	0
2433	2023	2	F	32	0	0	1	1	0	0	0	0	0	0	1	0	0	0	0	1	0	0	0	0
2434	2021	3	F	32	1	1	0	0	0	1	0	0	0	0	1	1	0	0	0	1	0	0	1	0
2435	2021	1	M	32	0	1	1	0	1	0	0	0	0	0	1	0	0	0	1	NA	0	0	0	0
2436	2021	10	M	32	0	1	1	1	0	0	0	0	0	0	1	0	0	0	0	2	0	1	0	0
2437	2022	3	M	32	0	0	1	0	1	0	1	0	0	0	1	0	1	0	0	2	1	0	1	1
2438	2022	7	M	32	0	1	1	0	0	1	0	0	0	0	1	1	0	0	0	2	0	1	1	1
2439	2023	11	M	32	0	0	1	0	1	1	0	0	0	0	1	0	1	0	0	3	1	1	0	1
2440	2024	3	F	32	1	1	1	0	0	0	0	0	0	0	1	0	0	0	0	1	0	1	0	0
2441	2022	6	M	32	1	0	1	0	0	1	0	1	0	0	0	0	0	0	0	3	0	0	1	1
2442	2020	9	F	32	1	1	1	1	0	0	0	0	0	0	1	1	1	0	0	NA	0	0	0	0
2443	2022	7	F	32	0	1	1	1	0	1	0	0	0	0	1	1	1	0	0	1	0	1	0	1
2444	2023	5	F	32	1	1	1	0	0	1	0	0	0	0	1	1	0	0	0	2	1	0	1	1
2445	2024	11	M	32	1	1	1	1	0	0	0	0	0	0	1	1	0	0	0	2	0	1	0	1
2446	2023	3	M	32	1	1	1	1	1	0	0	0	0	0	1	1	0	0	0	2	0	1	1	0
2447	2023	6	M	32	1	1	1	0	1	1	0	0	0	0	1	0	0	0	0	NA	0	0	0	0
2448	2024	8	F	32	1	1	1	0	1	1	0	0	0	0	1	1	0	0	0	2	0	1	1	1
2449	2021	1	F	32	0	1	1	0	1	1	1	0	0	0	1	1	0	0	0	2	0	1	0	0
2450	2020	1	F	33	0	0	0	0	0	0	0	0	0	0	1	1	0	0	1	2	1	1	1	1
2451	2020	8	F	33	0	0	0	0	0	0	0	0	0	0	1	1	0	0	0	NA	0	0	0	0
2452	2020	9	F	33	0	0	0	0	0	0	0	0	0	0	1	0	0	0	0	2	1	1	1	1
2453	2020	10	F	33	0	0	0	0	0	0	0	0	0	0	1	1	0	0	0	2	0	0	0	1
2454	2020	11	F	33	0	0	0	0	0	0	0	0	0	0	0	0	1	0	0	NA	0	0	0	0
2455	2020	11	M	33	0	0	0	0	0	0	0	0	0	0	0	0	1	0	0	2	1	1	1	1
2456	2021	1	M	33	0	0	0	0	0	0	0	0	0	0	0	0	0	0	1	2	1	1	1	1
2457	2021	3	M	33	0	0	0	0	0	0	0	0	0	0	0	0	0	0	1	2	0	0	0	0

ID	Yr	Mo	Sex	Age	Tre	Grs	Rag	Mug	HDM	Dan	Mld	Food	Drg	Ven	Rhi	Cnj	Ast	AsE	Urt	Sev	Prt	Obs	Rho	Snz
2458	2021	3	F	33	0	0	0	0	0	0	0	0	0	0	0	0	0	0	1	2	0	1	0	0
2459	2021	5	M	33	0	0	0	0	0	0	0	0	0	0	0	0	1	1	0	2	0	1	0	0
2460	2021	11	F	33	0	0	0	0	0	0	0	0	0	0	0	1	0	0	0	3	0	1	0	1
2461	2021	1	M	33	0	0	0	0	0	0	0	0	0	0	0	0	1	0	0	3	0	1	1	1
2462	2022	1	M	33	0	0	0	0	0	0	0	0	0	0	0	0	0	0	1	NA	0	0	0	0
2463	2022	1	F	33	0	0	0	0	0	0	0	0	0	0	1	0	0	0	1	3	0	1	1	0
2464	2022	2	F	33	0	0	0	0	0	0	0	0	0	0	1	0	0	0	0	2	0	0	0	0
2465	2022	7	M	33	0	0	0	0	0	0	0	0	0	0	0	0	0	0	1	NA	0	0	0	0
2466	2022	7	M	33	0	0	0	0	0	0	0	0	0	0	1	1	0	0	0	2	0	1	1	1
2467	2022	7	F	33	0	0	0	0	0	0	0	0	0	0	1	0	0	0	0	NA	0	0	1	0
2468	2022	7	F	33	0	0	0	0	0	0	0	0	0	0	1	1	0	0	0	NA	0	1	0	0
2469	2022	9	F	33	0	0	0	0	0	0	0	0	0	0	1	0	0	0	0	2	0	1	1	1
2470	2022	9	F	33	0	0	0	0	0	0	0	0	0	0	1	0	1	0	1	2	1	1	1	0
2471	2022	9	M	33	0	0	0	0	0	0	0	0	0	0	1	1	0	0	0	2	0	1	0	0
2472	2022	10	F	33	0	0	0	0	0	0	0	0	0	0	0	0	0	0	0	1	0	0	0	0
2473	2022	11	M	33	0	0	0	0	0	0	0	0	0	0	0	0	0	0	0	2	1	0	0	0
2474	2023	3	F	33	0	0	0	0	0	0	0	0	0	0	0	0	1	0	0	NA	0	0	0	0
2475	2023	4	F	33	0	0	0	0	0	0	0	0	0	0	1	0	1	0	0	2	0	1	1	1
2476	2023	4	M	33	0	0	0	0	0	0	0	0	0	0	1	0	0	0	0	2	0	1	1	1
2477	2023	5	F	33	0	0	0	0	0	0	0	0	0	0	1	0	1	0	0	2	1	1	1	1
2478	2023	7	F	33	0	0	0	0	0	0	0	0	0	0	0	0	0	0	1	2	1	1	1	1
2479	2023	8	F	33	0	0	0	0	0	0	0	0	0	0	0	0	0	0	1	2	1	1	1	1
2480	2023	8	F	33	0	0	0	0	0	0	0	0	0	0	0	0	0	0	1	NA	0	0	0	0
2481	2023	8	F	33	0	0	0	0	0	0	0	0	0	0	1	0	0	0	0	2	1	1	1	1
2482	2023	12	F	33	0	0	0	0	0	0	0	0	0	0	0	0	0	0	1	NA	0	0	0	0
2483	2024	2	M	33	0	0	0	0	0	0	0	0	0	0	1	0	0	0	0	2	0	1	0	0
2484	2024	4	M	33	0	0	0	0	0	0	0	0	0	0	0	0	0	0	0	NA	0	0	0	0
2485	2024	5	F	33	0	0	0	0	0	0	0	0	0	0	0	0	0	0	1	2	0	1	1	1
2486	2024	7	F	33	0	0	0	0	0	0	0	0	0	0	0	0	0	0	1	2	1	0	1	1
2487	2024	8	F	33	0	0	0	0	0	0	0	0	0	0	1	0	0	0	0	1	0	0	0	0
2488	2024	9	F	33	0	0	0	0	0	0	0	0	0	0	1	0	1	0	0	NA	0	0	0	0
2489	2024	10	M	33	0	0	0	0	0	0	0	0	0	0	1	0	1	0	0	2	1	1	0	1
2490	2024	11	F	33	0	0	0	0	0	0	0	0	0	0	0	0	0	0	0	NA	0	0	0	0
2491	2024	12	F	33	0	0	0	0	0	0	0	0	0	0	0	0	0	0	1	NA	0	0	0	0
2492	2024	12	F	33	0	0	0	0	0	0	0	0	0	0	0	0	1	0	1	2	0	1	0	0
2493	2023	8	F	33	0	0	0	0	0	0	0	0	0	0	0	0	0	0	1	2	1	0	0	1
2494	2022	7	F	33	0	0	0	0	0	0	0	1	0	0	0	0	0	0	1	1	1	1	1	0
2495	2022	9	F	33	0	0	0	0	0	0	0	1	0	0	0	0	0	0	0	2	0	1	1	0
2496	2024	2	F	33	0	0	0	0	0	0	0	0	1	0	0	0	0	0	0	NA	0	0	0	0
2497	2024	11	M	33	0	0	0	0	0	0	0	0	1	0	0	0	0	0	0	NA	0	0	0	0
2498	2024	12	M	33	0	0	0	0	0	0	0	0	0	1	0	0	0	0	1	NA	0	0	0	0
2499	2020	3	M	33	0	1	0	0	0	0	0	0	0	0	1	0	1	0	0	NA	0	0	0	0
2500	2021	2	M	33	0	0	0	0	1	0	0	0	0	0	1	1	1	0	0	NA	1	1	0	0
2501	2021	8	F	33	0	0	0	0	1	0	0	0	0	0	1	1	1	0	0	2	0	1	0	1
2502	2020	8	M	33	0	0	1	0	0	0	0	0	0	0	1	1	0	0	0	NA	0	0	0	0
2503	2022	9	M	33	0	0	1	0	0	0	0	0	0	0	1	1	0	0	0	1	0	0	0	1
2504	2023	8	M	33	0	0	1	0	0	0	0	0	0	0	1	0	0	0	0	NA	0	0	0	0
2505	2023	9	F	33	0	0	1	0	0	0	0	0	0	0	1	0	0	0	0	1	0	1	0	0
2506	2024	1	F	33	0	0	1	0	0	0	0	0	0	0	1	1	0	0	0	NA	0	0	0	0
2507	2024	9	M	33	0	0	1	0	0	0	0	0	0	0	1	0	0	0	0	2	0	1	0	0
2508	2024	10	M	33	0	0	1	0	0	0	0	0	0	0	1	1	0	0	0	NA	0	0	0	0
2509	2024	10	F	33	0	0	1	0	0	0	0	0	0	0	1	0	0	0	0	NA	0	0	0	0
2510	2024	10	M	33	0	0	1	0	0	0	0	0	0	0	1	1	0	0	0	2	0	1	1	0
2511	2024	11	F	33	0	0	1	0	0	0	0	0	0	0	1	0	0	0	0	2	0	1	0	1
2512	2021	3	F	33	0	0	1	0	0	0	0	0	0	0	1	0	0	0	0	NA	0	0	0	0
2513	2022	8	M	33	0	0	0	0	1	0	0	0	0	0	0	0	0	0	1	2	0	0	1	1
2514	2023	1	F	33	0	0	0	0	1	0	0	0	0	0	1	0	0	0	0	NA	0	0	0	0
2515	2023	3	F	33	0	0	0	0	0	1	0	0	0	0	1	0	0	0	1	2	0	1	1	0
2516	2023	4	M	33	0	0	0	0	1	0	0	0	0	0	1	0	1	0	0	NA	0	0	0	0
2517	2024	4	F	33	0	0	0	0	1	0	0	0	0	0	1	1	0	0	0	2	0	1	0	0
2518	2024	5	F	33	1	0	0	0	0	0	0	0	0	0	1	1	0	0	0	1	1	1	1	1
2519	2024	5	M	33	0	0	0	0	1	0	0	0	0	0	1	0	0	0	0	1	1	1	1	1
2520	2020	11	F	33	1	0	0	0	0	0	0	0	0	1	1	0	0	0	0	NA	0	0	0	0

ID	Yr	Mo	Sex	Age	Tre	Grs	Rag	Mug	HDM	Dan	Mld	Food	Drg	Ven	Rhi	Cnj	Ast	AsE	Urt	Sev	Prt	Obs	Rho	Snz
2521	2021	12	M	33	0	0	0	0	1	0	0	0	0	1	0	0	0	0	0	1	1	1	1	1
2522	2022	3	F	33	0	0	1	0	0	0	0	0	1	0	1	0	0	0	1	NA	0	0	0	0
2523	2024	4	M	33	0	0	1	0	0	0	0	0	0	1	1	0	0	0	0	2	0	1	1	1
2524	2020	8	M	33	1	0	1	0	0	0	0	0	0	0	1	1	0	0	0	NA	0	0	0	0
2525	2020	12	M	33	0	0	1	1	0	0	0	0	0	0	1	1	0	0	0	1	1	1	1	1
2526	2022	1	F	33	0	0	1	0	1	0	0	0	0	0	1	0	0	0	0	2	0	1	0	0
2527	2022	4	F	33	0	1	1	0	0	0	0	0	0	0	1	0	0	0	0	2	0	1	0	1
2528	2024	11	M	33	1	0	1	0	0	0	0	0	0	0	1	0	0	0	1	2	1	1	1	1
2529	2024	12	F	33	0	0	1	0	1	0	0	0	0	0	1	0	1	0	0	2	0	0	1	0
2530	2022	5	M	33	1	1	0	0	0	0	0	0	0	0	1	1	0	0	0	2	0	1	0	0
2531	2023	4	F	33	0	0	1	1	0	0	0	0	1	0	1	0	0	0	0	NA	0	0	0	0
2532	2021	3	M	33	0	1	1	1	0	0	0	0	0	0	1	1	1	0	0	2	0	1	0	0
2533	2021	6	M	33	0	1	1	1	0	0	0	0	0	0	1	1	0	0	0	2	1	1	0	1
2534	2022	1	M	33	0	1	1	0	1	0	0	0	0	0	1	0	1	0	0	NA	0	0	0	0
2535	2023	3	F	33	0	1	1	0	0	1	0	0	0	0	1	0	0	0	0	2	1	1	0	1
2536	2023	3	F	33	0	0	1	0	0	1	1	0	0	0	1	0	0	0	0	NA	0	0	0	0
2537	2024	8	M	33	0	1	1	0	1	0	0	0	0	0	1	0	0	0	0	2	0	1	1	1
2538	2022	8	F	33	1	1	0	0	1	0	0	0	0	0	1	1	0	0	0	NA	0	0	0	0
2539	2021	12	M	33	0	1	1	1	0	0	0	0	0	1	0	0	0	0	0	1	0	1	1	1
2540	2022	12	F	33	0	1	1	0	0	0	1	1	1	0	1	1	1	0	0	0	1	1	1	1
2541	2022	3	F	33	1	1	1	0	0	1	0	0	0	0	1	1	0	0	0	NA	0	0	0	0
2542	2023	10	M	33	1	1	1	0	0	1	0	0	0	0	1	0	1	0	0	NA	0	0	0	0
2543	2020	1	F	34	0	0	0	0	0	0	0	0	0	0	1	0	0	0	1	NA	0	0	0	0
2544	2020	3	F	34	0	0	0	0	0	0	0	0	0	0	0	0	0	0	1	2	0	1	1	0
2545	2020	3	M	34	0	0	0	0	0	0	0	0	0	0	1	1	0	0	0	NA	0	0	0	0
2546	2020	6	F	34	0	0	0	0	0	0	0	0	0	0	0	0	0	0	1	NA	0	0	0	0
2547	2020	9	M	34	0	0	0	0	0	0	0	0	0	0	1	1	0	0	0	NA	0	0	0	0
2548	2020	11	F	34	0	0	0	0	0	0	0	0	0	0	0	0	0	0	1	1	0	0	1	0
2549	2020	11	F	34	0	0	0	0	0	0	0	0	0	0	1	0	1	0	0	2	0	1	0	0
2550	2021	5	F	34	0	0	0	0	0	0	0	0	0	0	0	0	0	0	1	0	1	1	1	1
2551	2021	6	M	34	0	0	0	0	0	0	0	0	0	0	0	0	0	0	1	1	0	1	0	0
2552	2021	9	F	34	0	0	0	0	0	0	0	0	0	0	0	0	0	0	0	2	0	0	1	0
2553	2021	10	F	34	0	0	0	0	0	0	0	0	0	0	0	0	0	0	1	2	0	1	0	0
2554	2021	5	F	34	0	0	0	0	0	0	0	0	0	0	0	0	0	1	0	2	1	1	1	1
2555	2021	5	F	34	0	0	0	0	0	0	0	0	0	0	0	1	0	0	0	1	0	1	0	0
2556	2021	11	M	34	0	0	0	0	0	0	0	0	0	0	0	0	0	0	0	NA	0	0	0	0
2557	2021	2	F	34	0	0	0	0	0	0	0	0	0	0	0	0	0	1	0	NA	0	0	0	0
2558	2021	3	F	34	0	0	0	0	0	0	0	0	0	0	0	0	1	0	1	NA	0	0	0	0
2559	2022	1	F	34	0	0	0	0	0	0	0	0	0	0	0	0	0	0	0	NA	0	0	0	0
2560	2022	2	M	34	0	0	0	0	0	0	0	0	0	0	1	0	0	0	0	0	0	1	0	1
2561	2022	3	M	34	0	0	0	0	0	0	0	0	0	0	1	0	1	0	0	NA	0	1	1	1
2562	2022	3	F	34	0	0	0	0	0	0	0	0	0	0	1	0	0	0	0	1	1	0	0	1
2563	2022	5	F	34	0	0	0	0	0	0	0	0	0	0	0	0	0	0	0	0	1	1	0	0
2564	2022	9	M	34	0	0	0	0	0	0	0	0	0	0	1	1	0	0	0	NA	0	0	0	0
2565	2022	9	M	34	0	0	0	0	0	0	0	0	0	0	0	0	0	0	1	2	1	0	0	0
2566	2022	10	F	34	0	0	0	0	0	0	0	0	0	0	0	0	0	0	1	1	1	1	1	1
2567	2023	2	F	34	0	0	0	0	0	0	0	0	0	0	0	0	0	0	1	NA	0	0	0	0
2568	2023	4	F	34	0	0	0	0	0	0	0	0	0	0	0	0	0	0	1	NA	0	0	0	0
2569	2023	5	F	34	0	0	0	0	0	0	0	0	0	0	0	0	0	0	1	1	1	1	1	1
2570	2023	8	F	34	0	0	0	0	0	0	0	0	0	0	0	0	0	0	0	NA	0	0	0	0
2571	2023	9	F	34	0	0	0	0	0	0	0	0	0	0	1	0	0	0	0	NA	0	0	0	0
2572	2023	12	F	34	0	0	0	0	0	0	0	0	0	0	0	0	0	0	0	NA	0	0	0	0
2573	2024	1	F	34	0	0	0	0	0	0	0	0	0	0	1	0	0	0	0	NA	0	0	0	0
2574	2024	2	F	34	0	0	0	0	0	0	0	0	0	0	1	0	0	0	0	2	0	1	0	0
2575	2024	3	M	34	0	0	0	0	0	0	0	0	0	0	0	0	0	0	1	2	1	1	0	0
2576	2024	5	F	34	0	0	0	0	0	0	0	0	0	0	0	0	0	0	1	1	1	1	1	1
2577	2024	7	F	34	0	0	0	0	0	0	0	0	0	0	0	0	0	0	1	2	1	1	1	1
2578	2024	7	F	34	0	0	0	0	0	0	0	0	0	0	0	0	0	0	1	1	1	1	1	1
2579	2024	7	F	34	0	0	0	0	0	0	0	0	0	0	0	0	0	0	1	NA	0	0	0	0
2580	2024	9	M	34	0	0	0	0	0	0	0	0	0	0	0	0	0	0	0	NA	0	0	0	0
2581	2024	10	M	34	0	0	0	0	0	0	0	0	0	0	0	1	0	0	0	2	0	0	1	1
2582	2024	12	F	34	0	0	0	0	0	0	0	0	0	0	0	0	0	0	0	0	1	0	0	0
2583	2021	4	F	34	0	0	0	0	0	0	0	0	0	1	0	0	0	0	1	NA	0	0	0	0

ID	Yr	Mo	Sex	Age	Tre	Grs	Rag	Mug	HDM	Dan	Mld	Food	Drg	Ven	Rhi	Cnj	Ast	AsE	Urt	Sev	Prt	Obs	Rho	Snz
2584	2021	10	F	34	0	0	0	0	0	0	0	0	1	0	0	0	0	0	0	2	0	1	0	1
2585	2021	10	F	34	0	0	0	0	0	0	0	0	1	0	0	0	0	0	0	1	1	1	1	1
2586	2021	12	F	34	0	0	0	0	0	0	0	0	1	0	1	1	0	0	0	NA	0	0	0	0
2587	2021	12	M	34	0	0	0	0	0	0	0	0	1	0	0	0	0	0	0	2	1	1	1	1
2588	2023	3	M	34	0	0	0	0	0	0	0	0	1	0	0	0	0	0	0	1	1	1	1	1
2589	2023	10	F	34	0	0	0	0	0	0	0	0	1	0	0	0	0	0	0	0	1	1	1	1
2590	2024	4	F	34	0	0	0	0	0	0	0	0	1	0	0	0	0	0	0	2	0	0	1	0
2591	2020	6	F	34	0	0	0	0	1	0	0	0	0	0	1	1	0	0	0	1	1	1	1	1
2592	2021	5	F	34	0	0	0	0	1	0	0	0	0	0	0	0	0	0	0	1	1	1	1	1
2593	2021	8	F	34	0	0	0	0	0	0	1	0	0	0	1	0	0	0	0	1	1	1	0	0
2594	2021	11	F	34	0	0	0	0	1	0	0	0	0	0	1	0	0	0	0	2	1	1	1	1
2595	2020	2	M	34	0	0	1	0	0	0	0	0	0	0	1	1	0	0	0	1	0	1	1	1
2596	2020	9	M	34	0	0	1	0	0	0	0	0	0	0	1	1	0	0	0	NA	0	0	0	0
2597	2021	3	F	34	0	0	1	0	0	0	0	0	0	0	0	0	1	0	0	1	0	1	1	1
2598	2022	8	F	34	0	0	1	0	0	0	0	0	0	0	1	1	0	0	0	NA	0	0	0	0
2599	2023	9	M	34	0	0	1	0	0	0	0	0	0	0	1	1	0	0	0	2	0	1	0	0
2600	2023	9	F	34	0	0	1	0	0	0	0	0	0	0	1	1	0	0	0	1	0	1	0	0
2601	2024	5	M	34	0	0	1	0	0	0	0	0	0	0	1	1	0	0	0	1	1	0	0	0
2602	2022	5	F	34	0	0	0	0	1	0	0	0	0	0	1	0	0	0	0	2	1	0	1	1
2603	2023	3	F	34	0	0	0	0	1	0	0	0	0	0	1	0	1	0	0	2	0	0	1	1
2604	2024	1	M	34	0	0	0	0	1	0	0	0	0	0	1	1	0	0	0	1	0	1	0	0
2605	2024	3	M	34	0	1	0	0	0	0	0	0	0	0	1	1	1	0	0	1	1	1	1	1
2606	2024	3	M	34	0	0	0	0	1	0	0	0	0	0	1	0	1	0	0	2	0	1	1	1
2607	2021	12	F	34	0	0	1	0	0	0	0	0	0	1	0	0	0	0	0	2	0	1	0	0
2608	2020	2	F	34	0	0	1	0	1	0	0	0	0	0	1	1	0	0	0	0	1	1	1	1
2609	2020	2	F	34	0	0	1	0	0	1	0	0	0	0	1	1	0	0	0	1	1	1	1	1
2610	2021	3	M	34	0	0	1	0	1	0	0	0	0	0	1	1	1	1	0	NA	0	0	0	0
2611	2021	5	F	34	0	0	1	1	0	0	0	0	0	0	1	0	0	0	0	NA	0	0	0	0
2612	2022	1	M	34	1	0	1	0	0	0	0	0	0	0	1	1	0	0	0	NA	0	0	0	0
2613	2022	6	M	34	0	1	1	0	0	0	0	0	0	0	1	1	0	0	0	NA	0	0	0	0
2614	2022	7	F	34	0	0	1	0	0	1	0	0	0	0	1	1	0	0	0	2	0	1	0	0
2615	2023	8	M	34	0	0	1	0	1	0	0	0	0	0	1	0	0	0	0	1	1	1	1	1
2616	2023	8	M	34	0	0	1	0	1	0	0	0	0	0	1	1	0	0	0	1	0	1	0	0
2617	2023	11	F	34	0	0	1	0	1	0	0	0	0	0	1	0	0	0	1	NA	0	0	0	0
2618	2024	1	M	34	0	0	1	0	0	1	0	0	0	0	1	1	0	0	0	2	1	0	1	1
2619	2024	9	F	34	0	0	1	0	1	0	0	0	0	0	1	1	1	0	0	1	1	1	0	0
2620	2024	9	M	34	0	0	1	1	0	0	0	0	0	0	1	1	1	0	0	1	0	1	0	0
2621	2023	11	F	34	0	1	0	0	1	0	0	0	0	0	1	0	0	0	0	1	1	1	0	1
2622	2020	3	F	34	0	1	1	0	0	0	0	0	1	0	1	1	1	0	0	2	1	1	0	0
2623	2021	12	M	34	0	0	1	0	1	0	0	0	0	1	0	1	0	0	0	1	1	1	1	1
2624	2021	8	M	34	0	0	1	1	0	0	0	0	0	1	1	0	0	0	0	1	0	1	0	0
2625	2020	7	F	34	0	1	1	0	0	1	0	0	0	0	1	1	0	0	1	NA	0	0	0	0
2626	2022	3	M	34	0	1	1	0	1	0	0	0	0	0	1	1	0	0	0	1	1	0	0	0
2627	2022	10	M	34	1	1	1	0	0	0	0	0	0	0	1	1	1	0	0	2	1	1	1	1
2628	2023	2	F	34	0	0	1	1	0	1	0	0	0	0	1	0	0	0	0	2	0	1	1	1
2629	2023	3	M	34	1	1	1	0	0	0	0	0	0	0	1	0	0	0	0	1	1	1	1	1
2630	2021	3	M	34	0	1	1	1	0	0	0	0	0	0	1	1	0	0	0	NA	0	0	0	0
2631	2021	3	F	34	0	1	1	1	0	0	0	0	0	0	1	0	1	0	1	2	0	1	0	0
2632	2023	10	F	34	0	0	0	0	1	1	1	0	0	0	1	0	0	0	0	NA	0	0	0	0
2633	2020	8	M	34	1	1	1	0	1	0	0	0	0	0	1	0	0	0	0	1	0	1	0	1
2634	2022	8	F	34	1	1	1	0	0	0	1	0	0	0	1	1	1	0	0	1	1	0	0	1
2635	2023	10	F	34	1	1	1	0	1	0	0	0	0	0	1	0	0	0	0	NA	0	0	0	0
2636	2024	3	F	34	0	1	1	1	0	1	0	0	0	1	1	1	0	0	0	2	1	0	0	0
2637	2020	9	M	34	1	1	1	1	1	1	1	0	0	0	1	1	0	0	0	2	0	1	0	0
2638	2020	2	M	35	0	0	0	0	0	0	0	0	0	0	1	1	0	0	0	NA	0	0	0	0
2639	2020	2	M	35	0	0	0	0	0	0	0	0	0	0	1	0	0	0	0	NA	0	1	0	0
2640	2020	5	F	35	0	0	0	0	0	0	0	0	0	0	0	0	0	0	1	NA	0	0	0	0
2641	2020	8	M	35	0	0	0	0	0	0	0	0	0	0	1	1	0	0	0	NA	0	0	0	0
2642	2020	11	M	35	0	0	0	0	0	0	0	0	0	0	0	0	0	0	1	1	0	1	1	0
2643	2020	12	M	35	0	0	0	0	0	0	0	0	0	0	0	0	1	0	0	NA	0	0	0	0
2644	2021	4	F	35	0	0	0	0	0	0	0	0	0	0	0	0	0	0	1	2	0	1	1	1
2645	2021	9	M	35	0	0	0	0	0	0	0	0	0	0	1	1	0	0	0	1	0	1	1	0
2646	2021	6	F	35	0	0	0	0	0	0	0	0	0	0	0	0	0	1	0	2	1	1	0	0

ID	Yr	Mo	Sex	Age	Tre	Grs	Rag	Mug	HDM	Dan	Mld	Food	Drg	Ven	Rhi	Cnj	Ast	AsE	Urt	Sev	Prt	Obs	Rho	Snz
2647	2021	12	M	35	0	0	0	0	0	0	0	0	0	0	0	1	0	1	0	2	0	1	1	1
2648	2022	3	M	35	0	0	0	0	0	0	0	0	0	0	1	1	0	0	0	1	0	1	0	0
2649	2022	4	F	35	0	0	0	0	0	0	0	0	0	0	0	0	1	0	0	2	1	1	1	1
2650	2022	4	M	35	0	0	0	0	0	0	0	0	0	0	0	0	0	0	0	0	0	1	1	1
2651	2022	4	M	35	0	0	0	0	0	0	0	0	0	0	0	0	0	0	1	2	0	1	1	1
2652	2022	4	F	35	0	0	0	0	0	0	0	0	0	0	1	0	0	0	0	NA	0	0	0	0
2653	2022	5	M	35	0	0	0	0	0	0	0	0	0	0	1	1	0	0	0	NA	0	0	0	0
2654	2022	6	F	35	0	0	0	0	0	0	0	0	0	0	1	0	1	0	0	2	1	1	1	1
2655	2022	6	F	35	0	0	0	0	0	0	0	0	0	0	0	0	0	0	1	NA	0	0	0	0
2656	2022	6	F	35	0	0	0	0	0	0	0	0	0	0	0	0	0	0	1	NA	0	0	0	0
2657	2022	7	F	35	0	0	0	0	0	0	0	0	0	0	1	1	0	0	0	1	0	1	1	1
2658	2022	9	M	35	0	0	0	0	0	0	0	0	0	0	0	0	1	0	0	2	1	0	0	1
2659	2022	10	F	35	0	0	0	0	0	0	0	0	0	0	0	0	0	0	1	NA	0	0	0	0
2660	2022	11	M	35	0	0	0	0	0	0	0	0	0	0	0	0	1	0	0	NA	0	0	0	0
2661	2023	4	F	35	0	0	0	0	0	0	0	0	0	0	1	0	0	0	0	2	1	1	1	1
2662	2023	5	M	35	0	0	0	0	0	0	0	0	0	0	1	0	0	0	0	0	1	1	1	1
2663	2023	8	F	35	0	0	0	0	0	0	0	0	0	0	0	0	0	0	1	2	1	1	1	1
2664	2023	8	F	35	0	0	0	0	0	0	0	0	0	0	0	0	0	0	1	NA	0	0	0	0
2665	2023	10	F	35	0	0	0	0	0	0	0	0	0	0	0	0	0	0	1	0	1	1	1	1
2666	2023	11	M	35	0	0	0	0	0	0	0	0	0	0	0	0	1	0	0	2	0	1	1	0
2667	2023	11	F	35	0	0	0	0	0	0	0	0	0	0	1	0	0	0	0	NA	0	0	0	0
2668	2024	1	M	35	0	0	0	0	0	0	0	0	0	0	0	0	0	0	1	0	0	1	0	0
2669	2024	2	F	35	0	0	0	0	0	0	0	0	0	0	0	0	0	0	0	NA	0	0	0	0
2670	2024	2	F	35	0	0	0	0	0	0	0	0	0	0	0	0	0	0	1	2	0	1	1	1
2671	2024	4	M	35	0	0	0	0	0	0	0	0	0	0	1	0	1	1	0	1	0	1	0	0
2672	2024	8	F	35	0	0	0	0	0	0	0	0	0	0	0	0	0	0	0	1	1	1	1	1
2673	2024	9	M	35	0	0	0	0	0	0	0	0	0	0	0	0	0	0	1	2	1	1	0	1
2674	2024	10	F	35	0	0	0	0	0	0	0	0	0	0	0	0	0	0	0	2	1	1	1	0
2675	2021	8	F	35	0	0	0	0	0	0	0	0	1	0	0	0	0	0	1	NA	0	0	0	0
2676	2021	9	F	35	0	0	0	0	0	0	0	0	0	1	1	0	0	0	0	NA	0	0	0	0
2677	2021	10	F	35	0	0	0	0	0	0	0	0	1	0	0	0	0	0	0	2	1	1	0	0
2678	2022	9	F	35	0	0	0	0	0	0	0	1	0	0	1	1	0	0	0	NA	0	0	0	0
2679	2023	8	F	35	0	0	0	0	0	0	0	0	0	1	0	0	1	0	0	2	1	1	0	0
2680	2021	3	F	35	0	0	0	0	1	0	0	0	0	0	1	0	1	0	1	2	0	1	1	1
2681	2021	3	F	35	0	0	0	0	0	1	0	0	0	0	1	1	0	0	0	NA	0	0	0	0
2682	2020	7	M	35	0	0	1	0	0	0	0	0	0	0	1	1	1	0	0	1	1	0	0	1
2683	2022	8	M	35	0	0	1	0	0	0	0	0	0	0	1	1	1	0	0	0	1	1	1	1
2684	2023	2	F	35	0	0	1	0	0	0	0	0	0	0	1	0	0	0	1	2	1	1	1	1
2685	2023	8	F	35	0	0	1	0	0	0	0	0	0	0	1	0	1	0	0	NA	0	0	0	0
2686	2023	10	M	35	0	0	1	0	0	0	0	0	0	0	1	0	0	0	0	0	1	1	1	1
2687	2024	5	F	35	0	0	1	0	0	0	0	0	0	0	1	1	0	0	0	0	0	1	0	0
2688	2024	8	F	35	0	0	1	0	0	0	0	0	0	0	0	0	0	0	0	NA	0	0	0	0
2689	2021	1	M	35	0	0	1	0	0	0	0	0	0	0	1	1	1	0	0	0	1	1	0	1
2690	2022	1	M	35	0	0	0	0	1	0	0	0	0	0	1	0	0	0	0	NA	0	0	0	0
2691	2022	1	F	35	0	0	0	0	1	0	0	0	0	0	1	0	0	0	0	0	1	1	1	1
2692	2023	6	F	35	1	0	0	0	0	0	0	0	0	0	1	1	0	0	0	NA	0	0	0	0
2693	2021	9	F	35	0	0	1	0	0	0	0	0	1	0	1	1	0	0	0	0	1	0	0	0
2694	2022	8	M	35	0	0	1	0	0	0	0	1	0	0	1	1	1	0	0	NA	0	0	0	0
2695	2021	4	F	35	0	0	1	0	0	0	0	0	0	1	1	0	0	0	0	2	1	0	1	1
2696	2021	12	M	35	0	0	1	0	0	0	0	0	0	1	0	0	0	1	0	NA	0	0	0	0
2697	2020	1	F	35	0	1	0	0	1	0	0	0	0	0	1	1	1	0	1	2	1	0	1	0
2698	2020	9	F	35	1	1	0	0	0	0	0	0	0	0	1	0	1	0	0	1	1	1	1	1
2699	2021	5	M	35	0	0	0	0	1	1	0	0	0	0	1	0	1	0	0	1	0	0	0	0
2700	2020	1	F	35	0	0	1	0	1	0	0	0	0	0	1	1	0	0	0	2	1	0	1	1
2701	2022	3	F	35	0	0	1	1	0	0	0	0	0	0	1	0	0	0	1	NA	0	0	0	0
2702	2022	6	M	35	0	0	1	0	0	1	0	0	0	0	1	1	0	0	0	1	0	1	1	0
2703	2022	6	F	35	0	0	1	0	1	0	0	0	0	0	1	1	1	0	0	NA	0	0	0	0
2704	2023	4	M	35	1	0	1	0	0	0	0	0	0	0	1	0	0	0	1	NA	0	0	0	0
2705	2023	5	M	35	1	0	1	0	0	0	0	0	0	0	1	1	0	0	0	NA	0	0	0	0
2706	2023	6	M	35	0	1	1	0	0	0	0	0	0	0	1	0	0	0	0	2	1	0	1	0
2707	2023	11	F	35	0	0	1	0	1	0	0	0	0	0	1	0	1	1	0	1	0	0	1	1
2708	2021	1	M	35	0	0	1	1	0	0	0	0	0	0	1	1	0	0	0	NA	0	0	0	0
2709	2021	5	F	35	0	0	1	0	1	0	0	0	0	1	0	1	0	0	0	2	1	1	1	1

ID	Yr	Mo	Sex	Age	Tre	Grs	Rag	Mug	HDM	Dan	Mld	Food	Drg	Ven	Rhi	Cnj	Ast	AsE	Urt	Sev	Prt	Obs	Rho	Snz
2710	2020	8	F	35	0	1	1	0	1	0	0	0	0	0	1	1	1	0	0	1	1	1	1	1
2711	2020	9	M	35	1	1	1	0	0	0	0	0	0	0	1	0	0	0	0	2	1	1	1	1
2712	2020	10	F	35	0	0	1	0	1	0	1	0	0	0	1	1	0	0	0	1	1	1	0	1
2713	2023	6	M	35	0	1	1	0	0	1	0	0	0	0	1	1	1	0	0	NA	0	0	0	0
2714	2023	8	F	35	0	1	1	0	1	0	0	0	0	0	1	1	0	0	0	NA	0	0	0	0
2715	2024	3	M	35	0	1	1	0	0	1	0	0	0	0	1	1	1	0	0	1	1	1	1	1
2716	2020	5	F	35	1	1	1	0	0	0	0	0	0	0	1	0	0	0	0	1	1	1	1	1
2717	2024	7	F	35	0	0	1	0	1	1	0	0	0	0	1	1	0	0	0	NA	0	0	0	0
2718	2021	1	M	35	0	0	1	1	0	0	1	0	0	0	1	1	0	0	0	2	0	1	0	0
2719	2021	12	F	35	1	1	0	0	1	0	0	0	0	1	0	0	1	0	0	1	0	0	0	1
2720	2020	7	F	35	0	1	1	0	1	1	0	0	0	0	1	1	1	0	0	2	0	0	0	1
2721	2023	8	F	35	0	1	1	0	1	1	0	0	0	0	1	0	0	0	0	1	0	1	1	1
2722	2024	7	F	35	0	1	1	0	1	1	0	0	0	0	1	1	0	0	0	NA	0	0	0	0
2723	2020	8	M	35	0	1	1	1	1	0	0	1	0	0	1	0	0	0	0	2	1	0	0	0
2724	2021	10	M	35	0	1	1	0	1	1	0	0	0	1	1	0	0	0	0	0	1	1	1	1
2725	2022	5	F	35	1	1	1	1	0	0	1	0	0	0	1	1	0	0	0	2	0	1	0	1
2726	2022	10	M	35	1	1	1	1	0	0	1	0	0	0	1	1	1	0	0	1	1	0	0	0
2727	2020	7	F	35	1	1	1	1	1	1	0	0	0	0	1	1	0	0	0	NA	0	0	0	0
2728	2020	1	M	36	0	0	0	0	0	0	0	0	0	0	0	0	1	0	0	NA	0	0	0	0
2729	2020	6	F	36	0	0	0	0	0	0	0	0	0	0	0	0	0	0	0	2	0	1	1	1
2730	2020	7	F	36	0	0	0	0	0	0	0	0	0	0	1	0	0	0	0	NA	0	0	0	0
2731	2020	8	M	36	0	0	0	0	0	0	0	0	0	0	1	0	0	0	0	1	0	1	1	1
2732	2020	10	M	36	0	0	0	0	0	0	0	0	0	0	1	1	1	0	0	NA	0	0	0	0
2733	2020	12	M	36	1	1	0	0	1	0	0	0	0	0	1	1	0	0	0	2	0	1	0	0
2734	2021	1	M	36	0	0	0	0	0	0	0	0	0	0	0	0	0	0	0	2	1	1	1	1
2735	2021	3	M	36	0	0	0	0	0	0	0	0	0	0	0	0	1	0	0	NA	0	0	0	0
2736	2021	4	F	36	0	0	0	0	0	0	0	0	0	0	0	0	0	0	1	2	0	1	0	0
2737	2021	6	M	36	0	0	0	0	0	0	0	0	0	0	1	1	0	0	0	2	1	1	0	0
2738	2021	6	F	36	0	0	0	0	0	0	0	0	0	0	0	0	1	0	0	1	1	1	0	1
2739	2021	7	F	36	0	0	0	0	0	0	0	0	0	0	1	0	0	0	0	NA	0	0	0	0
2740	2021	8	F	36	0	0	0	0	0	0	0	0	0	0	0	0	1	0	1	2	1	1	0	0
2741	2021	9	F	36	0	0	0	0	0	0	0	0	0	0	0	0	0	1	0	0	0	1	0	0
2742	2021	12	M	36	0	0	0	0	0	0	0	0	0	0	0	0	0	0	0	NA	0	0	0	0
2743	2021	5	M	36	0	0	0	0	0	0	0	0	0	0	0	0	0	1	0	NA	0	0	0	0
2744	2022	2	M	36	0	0	0	0	0	0	0	0	0	0	0	0	0	0	0	0	0	1	0	0
2745	2022	4	F	36	0	0	0	0	0	0	0	0	0	0	1	1	1	0	1	1	1	1	0	0
2746	2022	5	F	36	0	0	0	0	0	0	0	0	0	0	1	1	0	0	0	NA	0	0	0	0
2747	2022	6	F	36	0	0	0	0	0	0	0	0	0	0	0	0	0	0	1	NA	0	0	0	0
2748	2022	11	M	36	0	0	0	0	0	0	0	0	0	0	0	0	0	0	1	2	1	0	1	1
2749	2023	1	F	36	0	0	0	0	0	0	0	0	0	0	0	0	0	0	1	2	1	1	0	0
2750	2023	1	F	36	0	0	0	0	0	0	0	0	0	0	0	0	1	0	0	NA	0	0	0	0
2751	2023	1	M	36	0	0	0	0	0	0	0	0	0	0	0	0	0	0	1	2	1	1	0	1
2752	2023	1	M	36	0	0	0	0	0	0	0	0	0	0	1	0	0	0	0	NA	0	0	0	0
2753	2023	8	M	36	0	0	0	0	0	0	0	0	0	0	1	0	0	0	0	2	1	0	1	0
2754	2024	1	F	36	0	0	0	0	0	0	0	0	0	0	1	0	1	0	0	NA	0	0	0	0
2755	2024	1	F	36	0	0	0	0	0	0	0	0	0	0	0	0	1	0	0	1	1	1	1	1
2756	2024	2	M	36	0	0	0	0	0	0	0	0	0	0	0	0	0	0	1	NA	0	0	0	0
2757	2024	2	F	36	0	0	0	0	0	0	0	0	0	0	0	0	0	0	0	1	0	1	0	0
2758	2024	2	F	36	0	0	0	0	0	0	0	0	0	0	1	1	0	0	0	NA	0	0	0	0
2759	2024	5	F	36	0	0	0	0	0	0	0	0	0	0	0	0	0	0	0	2	1	1	1	0
2760	2024	7	F	36	0	0	0	0	0	0	0	0	0	0	1	0	0	0	1	NA	0	0	0	0
2761	2024	8	M	36	0	0	0	0	0	0	0	0	0	0	0	0	0	0	1	2	0	1	1	1
2762	2021	12	M	36	0	0	0	0	0	0	0	0	1	0	0	0	0	0	0	1	0	0	1	0
2763	2021	3	F	36	0	0	0	0	0	0	0	0	1	0	0	0	0	0	0	2	1	1	1	1
2764	2021	10	F	36	0	0	0	0	0	0	0	0	0	1	0	0	0	0	0	1	1	0	0	0
2765	2022	6	F	36	0	0	0	0	0	0	0	1	0	0	0	0	0	0	0	1	1	1	1	1
2766	2022	9	F	36	0	0	0	0	0	0	0	0	1	0	0	0	0	0	1	NA	0	0	0	0
2767	2020	11	F	36	0	0	0	0	1	0	0	0	0	0	1	0	1	0	0	1	1	1	1	1
2768	2021	6	F	36	0	0	0	0	0	1	0	0	0	0	1	0	0	0	0	2	0	0	0	1
2769	2021	11	F	36	0	0	0	0	1	0	0	0	0	0	1	1	0	0	0	1	1	1	1	1
2770	2020	8	M	36	0	0	1	0	0	0	0	0	0	0	1	0	0	0	0	0	1	1	1	1
2771	2021	5	M	36	0	0	1	0	0	0	0	0	0	0	1	1	0	0	0	0	0	1	1	0
2772	2022	9	M	36	0	0	1	0	0	0	0	0	0	0	1	1	0	0	0	NA	0	0	0	0

ID	Yr	Mo	Sex	Age	Tre	Grs	Rag	Mug	HDM	Dan	Mld	Food	Drg	Ven	Rhi	Cnj	Ast	AsE	Urt	Sev	Prt	Obs	Rho	Snz
2773	2022	11	F	36	0	0	1	0	0	0	0	0	0	0	1	0	0	0	1	2	0	0	1	1
2774	2023	8	F	36	0	0	1	0	0	0	0	0	0	0	1	0	1	1	0	NA	0	1	1	0
2775	2023	10	F	36	0	0	1	0	0	0	0	0	0	0	1	0	0	0	0	2	1	1	1	1
2776	2024	2	F	36	0	0	1	0	0	0	0	0	0	0	1	0	0	0	0	NA	0	0	0	0
2777	2024	6	F	36	0	0	1	0	0	0	0	0	0	0	1	0	0	0	0	NA	0	0	0	0
2778	2024	9	M	36	0	0	1	0	0	0	0	0	0	0	1	1	0	0	0	1	0	1	0	1
2779	2024	10	F	36	0	0	1	0	0	0	0	0	0	0	1	1	0	0	0	NA	0	0	0	0
2780	2022	3	M	36	0	0	0	0	1	0	0	0	0	0	1	1	0	0	0	2	1	1	0	0
2781	2023	5	F	36	0	1	0	0	0	0	0	0	0	0	1	1	0	0	0	NA	0	0	0	0
2782	2023	8	F	36	0	0	0	0	1	0	0	0	0	0	1	0	0	0	0	NA	0	0	0	0
2783	2024	6	M	36	0	1	0	0	0	0	0	0	0	0	1	0	0	0	0	2	0	1	1	1
2784	2020	11	F	36	0	0	0	0	1	0	0	1	0	0	1	0	1	0	0	2	1	0	0	1
2785	2021	8	F	36	0	0	1	0	0	0	0	0	1	0	1	0	1	0	1	NA	0	0	0	0
2786	2021	10	M	36	0	0	1	0	0	0	0	0	0	1	1	0	0	0	0	NA	0	0	0	0
2787	2020	10	M	36	1	1	0	0	0	0	0	0	0	0	1	0	1	0	0	NA	0	0	0	0
2788	2020	6	F	36	0	0	1	0	0	1	0	0	0	0	1	0	0	0	0	1	1	0	1	1
2789	2020	9	M	36	0	0	1	0	0	1	0	0	0	0	1	1	0	0	0	NA	0	0	0	0
2790	2020	10	M	36	0	0	1	0	1	0	0	0	0	0	1	1	1	0	0	2	1	1	1	1
2791	2021	11	M	36	1	0	1	0	0	0	0	0	0	0	1	1	1	0	0	NA	0	0	0	0
2792	2022	8	M	36	0	1	1	0	0	0	0	0	0	0	1	1	0	0	0	NA	0	0	0	0
2793	2022	12	F	36	0	0	1	0	0	1	0	0	0	0	1	0	1	0	0	1	0	1	0	0
2794	2023	8	F	36	0	1	1	0	0	0	0	0	0	0	1	0	0	0	0	NA	0	0	0	0
2795	2024	4	F	36	1	1	0	0	0	0	0	0	0	0	1	0	0	0	0	NA	0	0	0	0
2796	2021	3	F	36	1	1	0	0	0	0	0	0	1	0	1	1	0	0	0	1	1	1	1	1
2797	2021	10	M	36	0	0	0	0	1	1	0	0	0	1	0	1	0	0	0	1	1	0	0	0
2798	2020	9	F	36	1	0	0	0	1	1	0	0	0	0	1	1	1	0	0	NA	0	0	0	0
2799	2021	2	M	36	1	1	0	0	1	0	0	0	0	0	1	1	0	0	0	NA	0	0	0	0
2800	2020	2	M	36	0	1	1	0	0	1	0	0	0	0	1	1	0	0	1	2	0	0	2	2
2801	2022	5	M	36	0	1	1	1	0	0	0	0	0	0	1	0	0	0	0	NA	0	0	0	0
2802	2022	10	F	36	0	1	1	0	1	0	0	0	0	0	1	1	0	0	0	NA	0	0	0	0
2803	2022	10	F	36	1	1	1	0	0	0	0	0	0	0	1	0	1	0	0	NA	0	0	0	0
2804	2023	3	M	36	1	1	1	0	0	0	0	0	0	0	1	1	0	0	0	NA	0	1	1	1
2805	2023	6	F	36	1	1	1	0	0	0	0	0	0	0	1	1	0	0	0	NA	0	0	0	0
2806	2023	8	M	36	1	1	1	0	0	0	0	0	0	0	1	0	1	0	0	0	0	0	1	1
2807	2023	8	F	36	1	1	1	0	0	0	0	0	0	0	1	0	0	0	0	NA	0	0	0	0
2808	2021	11	M	36	0	0	1	0	1	0	1	0	0	1	0	0	0	0	0	2	0	1	0	0
2809	2021	5	F	36	0	1	1	0	1	1	0	0	0	0	1	1	1	0	0	NA	0	0	0	0
2810	2021	6	M	36	1	1	1	1	0	0	0	0	0	0	1	1	1	0	0	0	1	0	0	1
2811	2022	9	M	36	1	1	1	0	0	1	0	0	0	0	1	1	0	0	0	NA	0	0	0	0
2812	2023	11	M	36	0	1	1	1	1	0	0	0	0	0	1	0	0	0	0	2	1	0	1	1
2813	2024	4	F	36	0	1	1	0	1	1	0	0	0	0	1	1	1	0	1	NA	0	0	0	0
2814	2021	4	M	36	0	1	1	0	1	1	0	0	0	1	1	0	1	0	0	2	1	1	0	1
2815	2021	5	F	36	1	0	1	1	1	0	0	0	0	1	1	1	0	0	0	2	0	1	0	1
2816	2021	8	F	36	0	1	1	1	1	1	0	0	0	0	1	1	0	0	0	NA	0	0	0	0
2817	2021	2	F	36	1	1	1	0	0	1	1	0	0	0	1	1	0	0	0	2	0	1	0	1
2818	2021	9	F	36	0	1	1	1	1	1	1	0	0	1	1	1	0	0	0	1	1	1	1	1
2819	2020	1	M	37	0	0	0	0	0	0	0	0	0	0	0	0	0	0	1	1	1	0	0	0
2820	2020	2	M	37	0	0	0	0	0	0	0	0	0	0	1	0	0	0	0	NA	0	0	0	0
2821	2020	3	M	37	0	0	0	0	0	0	0	0	0	0	0	0	0	0	1	NA	0	0	0	0
2822	2020	3	M	37	0	0	0	0	0	0	0	0	0	0	1	0	0	0	0	1	0	1	0	0
2823	2020	11	F	37	0	0	0	0	0	0	0	0	0	0	1	0	1	0	0	2	0	1	1	1
2824	2021	1	F	37	0	0	0	0	0	0	0	0	0	0	0	0	0	0	1	NA	0	0	0	0
2825	2021	2	M	37	0	0	0	0	0	0	0	0	0	0	0	0	0	0	0	NA	0	0	0	0
2826	2021	3	F	37	0	0	0	0	0	0	0	0	0	0	0	0	0	0	0	1	1	1	1	1
2827	2021	4	F	37	0	0	0	0	0	0	0	0	0	0	0	0	0	0	1	0	0	0	1	1
2828	2021	5	M	37	0	0	0	0	0	0	0	0	0	0	1	1	0	0	1	2	0	1	0	0
2829	2021	6	M	37	0	0	0	0	0	0	0	0	0	0	0	0	0	0	0	1	0	1	0	1
2830	2021	11	F	37	0	0	0	0	0	0	0	0	0	0	0	0	0	0	0	NA	0	0	0	0
2831	2021	11	M	37	0	0	0	0	0	0	0	0	0	0	0	0	0	0	0	0	1	0	0	0
2832	2021	12	F	37	0	0	0	0	0	0	0	0	0	0	0	0	0	0	0	2	0	1	1	1
2833	2021	2	M	37	0	0	0	0	0	0	0	0	0	0	0	0	0	0	0	2	0	1	1	0
2834	2022	1	M	37	0	0	0	0	0	0	0	0	0	0	1	0	0	0	0	0	1	1	1	1
2835	2022	1	F	37	0	0	0	0	0	0	0	0	0	0	0	0	0	0	0	1	1	1	1	1

ID	Yr	Mo	Sex	Age	Tre	Grs	Rag	Mug	HDM	Dan	Mld	Food	Drg	Ven	Rhi	Cnj	Ast	AsE	Urt	Sev	Prt	Obs	Rho	Snz
2836	2022	3	M	37	0	0	0	0	0	0	0	0	0	0	1	0	0	0	0	NA	0	0	0	0
2837	2022	3	F	37	0	0	0	0	0	0	0	0	0	0	0	1	0	0	0	2	1	0	1	1
2838	2022	3	F	37	0	0	0	0	0	0	0	0	0	0	1	0	1	0	0	0	0	1	0	0
2839	2022	4	M	37	0	0	0	0	0	0	0	0	0	0	0	0	0	0	0	2	1	1	0	0
2840	2022	7	F	37	0	0	0	0	0	0	0	0	0	0	0	0	0	0	0	2	1	1	1	1
2841	2022	7	F	37	0	0	0	0	0	0	0	0	0	0	0	0	0	0	1	NA	0	0	0	0
2842	2022	8	M	37	0	0	0	0	0	0	0	0	0	0	0	0	0	0	1	2	0	1	0	1
2843	2022	12	M	37	0	0	0	0	0	0	0	0	0	0	0	0	0	0	0	2	1	1	1	1
2844	2022	12	F	37	0	0	0	0	0	0	0	0	0	0	0	0	0	0	1	2	0	1	0	0
2845	2023	2	F	37	0	0	0	0	0	0	0	0	0	0	0	0	0	0	1	1	1	1	1	1
2846	2023	2	M	37	0	0	0	0	0	0	0	0	0	0	0	0	0	0	0	NA	0	0	0	0
2847	2023	2	F	37	0	0	0	0	0	0	0	0	0	0	0	0	0	0	1	2	1	1	1	1
2848	2023	4	F	37	0	0	0	0	0	0	0	0	0	0	0	0	0	0	0	2	1	1	0	0
2849	2023	4	F	37	0	0	0	0	0	0	0	0	0	0	1	0	1	0	0	NA	0	0	0	0
2850	2023	5	F	37	0	0	0	0	0	0	0	0	0	0	0	0	1	0	0	NA	0	0	0	0
2851	2023	6	F	37	0	0	0	0	0	0	0	0	0	0	0	0	0	0	0	NA	0	0	0	0
2852	2024	4	F	37	0	0	0	0	0	0	0	0	0	0	0	0	1	0	0	NA	0	0	0	0
2853	2024	4	F	37	0	0	0	0	0	0	0	0	0	0	0	0	0	0	1	NA	0	0	0	0
2854	2024	4	M	37	0	0	0	0	0	0	0	0	0	0	0	0	1	1	0	NA	0	0	0	0
2855	2024	10	M	37	0	0	0	0	0	0	0	0	0	0	1	0	0	0	0	NA	0	0	0	0
2856	2024	10	F	37	0	0	0	0	0	0	0	0	0	0	1	0	1	0	1	NA	0	0	0	0
2857	2021	10	F	37	0	0	0	0	0	0	0	0	0	1	1	0	0	0	0	NA	0	0	0	0
2858	2024	11	F	37	0	0	0	0	0	0	0	1	0	0	0	0	0	0	0	NA	0	0	0	0
2859	2021	3	F	37	0	0	0	0	0	0	0	1	0	1	0	0	0	0	0	NA	0	0	0	0
2860	2020	12	M	37	1	0	0	0	0	0	0	0	0	0	1	0	0	0	0	2	0	1	0	1
2861	2021	9	M	37	0	0	1	0	0	0	0	0	0	0	1	0	0	0	1	2	1	1	1	1
2862	2021	9	M	37	0	0	1	0	0	0	0	0	0	0	1	0	0	0	0	2	0	1	1	0
2863	2022	1	F	37	0	0	1	0	0	0	0	0	0	0	1	0	0	0	0	NA	0	0	0	0
2864	2022	1	F	37	0	0	1	0	0	0	0	0	0	0	1	1	1	0	0	2	0	1	0	1
2865	2022	9	M	37	0	0	1	0	0	0	0	0	0	0	1	1	0	0	0	2	1	1	1	0
2866	2022	10	M	37	0	0	1	0	0	0	0	0	0	0	1	1	0	0	0	NA	0	0	0	0
2867	2022	10	F	37	0	0	1	0	0	0	0	0	0	0	1	1	0	0	0	2	1	1	0	1
2868	2023	2	F	37	0	0	1	0	0	0	0	0	0	0	1	1	0	0	0	2	1	1	1	1
2869	2023	3	F	37	0	0	1	0	0	0	0	0	0	0	1	1	0	0	0	NA	0	0	0	0
2870	2023	4	M	37	0	0	1	0	0	0	0	0	0	0	1	0	0	0	0	NA	0	0	0	0
2871	2023	8	F	37	0	0	1	0	0	0	0	0	0	0	1	0	0	0	0	NA	0	0	0	0
2872	2023	8	F	37	0	0	1	0	0	0	0	0	0	0	1	1	1	1	0	NA	0	0	0	0
2873	2024	10	M	37	0	0	1	0	0	0	0	0	0	0	1	0	0	0	0	NA	0	0	0	0
2874	2022	1	M	37	0	0	0	0	1	0	0	0	0	0	1	0	1	0	0	NA	0	0	0	0
2875	2022	2	F	37	0	0	0	0	1	0	0	0	0	0	1	1	1	0	0	1	1	0	0	0
2876	2022	4	F	37	0	0	0	0	1	0	0	0	0	0	1	0	0	0	1	NA	0	0	0	0
2877	2022	5	M	37	1	0	0	0	0	0	0	0	0	0	1	1	1	0	0	2	0	1	0	0
2878	2024	1	F	37	0	0	0	0	1	0	0	0	0	0	1	0	1	1	0	2	0	1	0	0
2879	2024	7	M	37	0	0	0	0	1	0	0	0	0	0	1	0	0	0	0	0	1	1	1	1
2880	2021	12	F	37	0	0	1	0	0	0	0	0	0	1	0	1	0	0	0	1	1	1	1	1
2881	2024	7	F	37	0	0	0	0	1	0	0	0	1	0	0	0	1	1	0	0	0	0	0	1
2882	2020	3	M	37	0	0	0	1	1	0	0	0	0	0	1	1	0	0	0	2	0	1	1	0
2883	2020	9	F	37	0	0	1	0	1	0	0	0	0	0	1	1	0	0	0	NA	0	0	0	0
2884	2021	8	M	37	1	0	1	0	0	0	0	0	0	0	1	1	0	0	0	NA	0	0	0	0
2885	2022	2	M	37	0	0	1	0	1	0	0	0	0	0	1	0	0	0	0	NA	0	0	0	0
2886	2022	4	M	37	0	1	1	0	0	0	0	0	0	0	1	1	0	0	0	NA	0	0	0	0
2887	2022	4	F	37	0	0	1	0	1	0	0	0	0	0	1	0	1	0	1	NA	0	0	0	0
2888	2022	10	M	37	0	1	1	0	0	0	0	0	0	0	1	1	0	0	0	2	1	0	1	0
2889	2023	10	M	37	0	0	1	0	0	0	1	0	0	0	1	0	0	0	0	2	1	1	0	0
2890	2024	4	M	37	0	1	1	0	0	0	0	0	0	0	1	1	0	0	0	NA	0	0	0	0
2891	2024	8	M	37	0	1	1	0	0	0	0	0	0	0	1	1	0	0	0	2	1	1	1	1
2892	2024	9	F	37	1	0	1	0	0	0	0	0	0	0	1	1	0	0	0	NA	0	0	0	0
2893	2022	2	M	37	0	1	0	0	1	0	0	0	0	0	1	1	0	0	0	2	0	1	1	1
2894	2023	7	M	37	0	1	0	0	0	1	0	0	0	0	1	0	0	0	1	0	0	1	0	0
2895	2024	4	M	37	1	1	0	0	0	0	0	0	0	0	1	0	0	0	0	2	1	0	1	1
2896	2024	8	F	37	1	1	0	0	0	0	0	0	0	1	0	0	1	0	1	NA	0	0	0	0
2897	2020	7	M	37	1	1	1	0	0	0	0	0	0	0	0	0	1	0	1	NA	0	0	0	0
2898	2020	11	F	37	0	0	1	1	1	0	0	0	0	0	1	1	1	0	0	NA	0	0	0	0

ID	Yr	Mo	Sex	Age	Tre	Grs	Rag	Mug	HDM	Dan	Mld	Food	Drg	Ven	Rhi	Cnj	Ast	AsE	Urt	Sev	Prt	Obs	Rho	Snz
2899	2021	11	F	37	0	1	1	0	1	0	0	0	0	0	1	1	1	0	0	NA	0	0	0	0
2900	2022	3	M	37	0	0	1	0	1	1	0	0	0	0	1	1	1	0	0	NA	0	0	0	0
2901	2022	5	F	37	0	0	1	0	1	1	0	0	0	0	1	0	1	0	1	1	1	1	0	1
2902	2022	5	M	37	0	1	1	0	0	1	0	0	0	0	1	1	1	0	0	NA	0	0	0	0
2903	2022	6	F	37	0	1	1	0	1	0	0	0	0	0	1	1	1	0	0	1	1	1	1	1
2904	2022	10	F	37	0	1	1	1	0	0	0	0	0	0	1	0	0	0	0	NA	0	0	0	0
2905	2021	9	F	37	1	1	0	0	0	0	1	0	1	0	1	1	1	0	1	2	1	1	1	1
2906	2021	9	M	37	1	0	1	0	0	1	0	0	0	1	1	0	0	0	0	NA	0	0	0	0
2907	2020	9	F	37	0	0	1	1	1	1	0	0	0	0	1	1	0	0	0	NA	0	0	0	0
2908	2021	9	M	37	1	0	1	1	1	0	0	0	0	0	1	1	0	0	0	2	0	0	1	1
2909	2022	1	F	37	1	1	1	1	0	0	0	0	0	0	1	1	1	0	0	NA	0	0	0	0
2910	2022	1	M	37	1	1	1	1	0	0	0	0	0	0	1	1	0	0	0	NA	0	0	0	0
2911	2022	10	M	37	0	0	1	1	1	0	1	0	0	0	1	0	0	0	0	2	0	1	0	1
2912	2022	11	M	37	0	1	1	0	1	1	0	0	0	0	1	1	1	0	0	NA	0	0	0	0
2913	2023	4	M	37	0	1	1	1	1	0	0	0	0	0	1	0	0	0	0	0	0	1	0	0
2914	2023	5	M	37	1	1	1	1	0	0	0	0	0	0	1	0	1	0	0	1	1	1	0	1
2915	2022	2	F	37	1	1	0	1	1	0	0	0	0	0	1	1	1	0	0	NA	0	0	0	0
2916	2020	2	F	38	0	0	0	0	0	0	0	0	0	0	1	1	0	0	0	2	0	1	0	0
2917	2020	2	F	38	0	0	0	0	0	0	0	0	0	0	1	0	1	0	0	NA	0	0	0	0
2918	2020	2	F	38	0	0	0	0	0	0	0	0	0	0	0	0	1	0	1	NA	0	0	0	0
2919	2020	6	F	38	0	0	0	0	0	0	0	0	0	0	1	0	0	0	0	NA	0	0	0	0
2920	2020	7	F	38	0	0	0	0	0	0	0	0	0	0	1	0	0	0	0	NA	0	0	0	0
2921	2020	8	F	38	0	0	0	0	0	0	0	0	0	0	0	0	1	0	0	1	1	1	1	1
2922	2020	9	M	38	0	0	0	0	0	0	0	0	0	0	1	1	0	0	0	NA	0	0	0	0
2923	2021	1	F	38	0	0	0	0	0	0	0	0	0	0	0	0	1	0	0	2	0	1	0	0
2924	2021	4	M	38	0	0	0	0	0	0	0	0	0	0	0	0	0	0	1	0	1	1	1	1
2925	2021	9	M	38	0	0	0	0	0	0	0	0	0	0	1	1	0	0	0	NA	0	0	0	0
2926	2021	11	F	38	0	0	0	0	0	0	0	0	0	0	0	0	0	1	0	1	1	1	1	0
2927	2022	1	F	38	0	0	0	0	0	0	0	0	0	0	0	0	0	0	0	0	1	1	1	1
2928	2022	2	F	38	0	0	0	0	0	0	0	0	0	0	0	0	0	0	0	NA	0	0	0	0
2929	2022	3	M	38	0	0	0	0	0	0	0	0	0	0	0	0	1	0	0	1	1	1	1	1
2930	2022	9	F	38	0	0	0	0	0	0	0	0	0	0	0	0	0	0	1	2	0	1	1	0
2931	2022	9	M	38	0	0	0	0	0	0	0	0	0	0	0	0	0	0	1	2	0	0	1	1
2932	2022	9	M	38	0	0	0	0	0	0	0	0	0	0	0	0	0	0	1	2	1	1	1	1
2933	2022	11	F	38	0	0	0	0	0	0	0	0	0	0	0	0	0	0	0	NA	0	0	0	0
2934	2022	12	M	38	0	0	0	0	0	0	0	0	0	0	1	1	0	0	0	0	0	1	1	0
2935	2023	1	F	38	0	0	0	0	0	0	0	0	0	0	0	0	1	0	0	0	0	1	1	0
2936	2023	4	M	38	0	0	0	0	0	0	0	0	0	0	0	0	0	0	1	2	1	1	0	0
2937	2023	4	F	38	0	0	0	0	0	0	0	0	0	0	0	0	0	0	1	NA	0	0	0	0
2938	2023	10	F	38	0	0	0	0	0	0	0	0	0	0	0	0	0	0	0	2	1	1	0	0
2939	2024	1	F	38	0	0	0	0	0	0	0	0	0	0	0	0	0	0	1	2	0	1	1	1
2940	2024	3	F	38	0	0	0	0	0	0	0	0	0	0	0	0	0	0	0	NA	0	0	0	0
2941	2024	3	F	38	0	0	0	0	0	0	0	0	0	0	1	1	0	0	0	2	1	1	1	1
2942	2024	3	F	38	0	0	0	0	0	0	0	0	0	0	0	0	0	0	0	2	0	1	0	0
2943	2024	3	F	38	0	0	0	0	0	0	0	0	0	0	0	0	0	0	0	NA	0	0	0	0
2944	2024	7	M	38	0	0	0	0	0	0	0	0	0	0	0	0	0	0	1	NA	0	0	0	0
2945	2024	8	M	38	0	0	0	0	0	0	0	0	0	0	1	0	0	0	0	1	1	1	0	0
2946	2024	9	F	38	0	0	0	0	0	0	0	0	0	0	0	1	0	0	1	2	0	1	1	0
2947	2024	9	F	38	0	0	0	0	0	0	0	0	0	0	0	0	0	0	0	NA	0	1	1	1
2948	2024	11	F	38	0	0	0	0	0	0	0	0	0	0	0	0	0	0	0	NA	0	0	0	0
2949	2024	12	F	38	0	0	0	0	0	0	0	0	0	0	0	0	0	0	1	2	0	1	0	0
2950	2021	9	M	38	0	0	0	0	0	0	0	0	0	1	1	0	0	0	0	NA	0	0	0	0
2951	2021	11	F	38	0	0	0	0	0	0	0	0	0	1	0	0	0	0	0	NA	0	0	0	0
2952	2021	10	M	38	0	0	0	0	0	0	0	0	0	1	1	0	0	0	0	NA	0	0	0	0
2953	2020	1	F	38	0	0	0	0	1	0	0	0	0	0	1	0	1	0	0	NA	0	0	0	0
2954	2020	7	M	38	0	0	1	0	0	0	0	0	0	0	1	1	1	0	0	2	0	1	0	1
2955	2020	9	M	38	0	0	1	0	0	0	0	0	0	0	1	1	0	0	0	1	1	1	0	0
2956	2021	12	F	38	0	0	1	0	0	0	0	0	0	0	1	1	0	0	0	NA	0	0	0	0
2957	2022	3	M	38	0	0	1	0	0	0	0	0	0	0	1	1	0	0	0	NA	0	0	0	0
2958	2022	9	F	38	0	0	1	0	0	0	0	0	0	0	1	1	1	1	0	2	0	1	0	0
2959	2022	11	F	38	0	0	1	0	0	0	0	0	0	0	1	1	0	0	0	NA	0	0	0	0
2960	2023	8	M	38	0	0	1	0	0	0	0	0	0	0	1	0	0	0	0	1	1	0	1	0
2961	2023	8	M	38	0	0	1	0	0	0	0	0	0	0	1	0	0	0	0	NA	0	0	0	0

ID	Yr	Mo	Sex	Age	Tre	Grs	Rag	Mug	HDM	Dan	Mld	Food	Drg	Ven	Rhi	Cnj	Ast	AsE	Urt	Sev	Prt	Obs	Rho	Snz
2962	2023	8	F	38	0	0	1	0	0	0	0	0	0	0	1	0	0	0	0	1	1	0	1	0
2963	2023	9	F	38	0	0	1	0	0	0	0	0	0	0	1	1	1	0	0	2	1	1	0	0
2964	2023	9	F	38	0	0	1	0	0	0	0	0	0	0	1	0	1	0	0	NA	0	0	0	0
2965	2023	11	M	38	0	0	1	0	0	0	0	0	0	0	1	0	0	0	0	NA	0	0	0	0
2966	2024	2	M	38	0	0	1	0	0	0	0	0	0	0	1	1	0	0	0	NA	0	0	0	0
2967	2024	2	M	38	0	0	1	0	0	0	0	0	0	0	1	1	0	0	1	2	1	0	1	1
2968	2024	2	M	38	0	0	1	0	0	0	0	0	0	0	1	1	0	0	0	2	1	1	1	1
2969	2024	7	M	38	0	0	1	0	0	0	0	0	0	0	1	1	0	1	1	2	1	1	1	1
2970	2022	1	F	38	0	1	0	0	0	0	0	0	0	0	0	0	0	0	0	2	1	1	1	1
2971	2022	4	M	38	0	0	0	0	1	0	0	0	0	0	1	0	1	0	1	NA	0	0	0	0
2972	2023	3	F	38	0	0	0	0	0	1	0	0	0	0	1	0	1	0	0	NA	0	0	0	0
2973	2023	6	M	38	1	0	0	0	0	0	0	0	0	0	1	0	0	0	1	0	0	1	0	0
2974	2024	4	F	38	1	0	0	0	0	0	0	0	0	0	0	0	1	0	0	NA	0	0	0	0
2975	2024	11	F	38	0	0	0	0	1	0	0	0	0	0	1	0	1	0	0	NA	0	0	0	0
2976	2021	10	M	38	0	0	1	0	0	0	0	0	0	1	0	0	0	1	0	2	0	1	0	0
2977	2021	12	M	38	0	0	1	0	0	0	0	0	0	1	1	1	0	0	0	NA	0	0	0	0
2978	2022	12	F	38	0	0	0	0	1	0	0	0	0	1	0	0	0	0	1	2	0	1	0	0
2979	2021	5	F	38	0	0	0	1	1	0	0	0	0	0	1	1	1	0	0	1	1	1	1	1
2980	2020	1	M	38	0	1	1	0	0	0	0	0	0	0	1	0	0	0	1	NA	0	0	0	0
2981	2020	9	F	38	0	0	1	0	0	1	0	0	0	0	1	1	0	0	0	NA	0	0	0	0
2982	2020	9	M	38	0	0	1	0	0	1	0	0	0	0	1	1	0	0	0	NA	0	0	0	0
2983	2022	11	F	38	0	1	1	0	0	0	0	0	0	0	1	0	1	0	0	1	0	1	1	1
2984	2023	4	M	38	0	1	1	0	0	0	0	0	0	0	1	1	0	0	0	2	1	1	1	1
2985	2021	2	F	38	0	0	1	1	0	0	0	0	0	0	1	1	0	0	0	NA	0	0	0	0
2986	2023	10	M	38	0	0	0	0	1	0	1	0	0	0	1	0	0	0	1	NA	0	0	0	0
2987	2024	4	F	38	1	0	0	0	1	0	0	0	0	0	1	0	1	0	0	2	0	1	0	1
2988	2020	1	M	38	0	0	0	0	1	1	1	0	0	0	1	0	1	0	0	NA	0	0	0	0
2989	2020	10	F	38	1	1	0	1	0	0	0	0	0	0	1	0	1	0	0	1	0	1	0	0
2990	2020	2	M	38	0	1	1	0	1	0	0	0	0	0	1	1	1	0	0	2	0	0	1	1
2991	2020	7	M	38	0	1	1	0	1	0	0	0	0	0	1	1	0	0	0	1	1	1	1	1
2992	2020	12	F	38	0	1	1	0	1	0	0	0	0	0	1	1	1	0	0	2	1	1	1	1
2993	2021	10	F	38	0	0	1	0	1	1	0	0	0	0	1	0	1	0	0	0	1	0	1	0
2994	2022	2	F	38	0	1	1	0	1	0	0	0	0	0	1	1	1	0	0	NA	0	0	0	0
2995	2022	9	F	38	1	1	1	0	0	0	0	0	0	0	1	1	1	0	0	2	0	1	0	1
2996	2024	7	M	38	1	1	1	0	0	0	0	0	0	0	1	1	0	0	0	1	1	1	1	1
2997	2024	10	M	38	1	1	1	0	0	0	0	0	0	0	1	1	0	0	0	NA	0	0	0	0
2998	2023	8	F	38	1	1	1	0	0	0	0	1	0	0	1	0	1	0	0	2	0	1	1	1
2999	2020	6	F	38	0	1	1	0	1	1	0	0	0	0	1	1	0	0	0	1	1	1	1	1
3000	2020	1	F	39	0	0	0	0	0	0	0	0	0	0	0	0	0	0	1	1	0	1	1	1
3001	2020	3	M	39	0	0	0	0	0	0	0	0	0	0	1	0	0	0	0	NA	0	0	0	0
3002	2020	6	F	39	0	0	0	0	0	0	0	0	0	0	0	0	0	0	0	1	1	0	1	1
3003	2021	2	F	39	0	0	0	0	0	0	0	0	0	0	1	0	0	0	0	2	0	1	0	0
3004	2021	3	M	39	0	0	0	0	0	0	0	0	0	0	0	0	0	0	1	2	0	1	0	1
3005	2021	8	M	39	0	0	0	0	0	0	0	0	0	0	0	0	0	0	1	NA	0	0	0	0
3006	2021	8	F	39	0	0	0	0	0	0	0	0	0	0	0	0	0	0	0	1	0	1	1	0
3007	2021	9	F	39	0	0	0	0	0	0	0	0	0	0	0	0	0	1	0	2	1	0	0	1
3008	2021	10	F	39	0	0	0	0	0	0	0	0	0	0	1	1	0	0	0	NA	0	0	0	0
3009	2021	2	F	39	0	0	0	0	0	0	0	0	0	0	1	0	0	0	0	NA	0	0	0	0
3010	2022	1	M	39	0	0	0	0	0	0	0	0	0	0	0	0	0	0	0	2	0	1	1	0
3011	2022	1	M	39	0	0	0	0	0	0	0	0	0	0	0	0	1	0	0	2	0	1	0	0
3012	2022	1	M	39	0	0	0	0	0	0	0	0	0	0	0	0	0	0	0	2	1	1	1	1
3013	2022	2	M	39	0	0	0	0	0	0	0	0	0	0	0	0	0	0	1	2	0	1	1	1
3014	2022	3	F	39	0	0	0	0	0	0	0	0	0	0	1	0	0	0	0	1	0	1	0	0
3015	2022	4	F	39	0	0	0	0	0	0	0	0	0	0	0	0	0	0	0	1	0	1	0	0
3016	2022	6	F	39	0	0	0	0	0	0	0	0	0	0	1	0	0	0	0	NA	0	0	0	0
3017	2022	6	M	39	0	0	0	0	0	0	0	0	0	0	1	0	0	0	0	2	0	1	0	0
3018	2022	8	M	39	0	0	0	0	0	0	0	0	0	0	1	0	1	0	0	2	1	1	0	0
3019	2022	11	F	39	0	0	0	0	0	0	0	0	0	0	1	0	1	0	0	NA	0	0	0	0
3020	2022	11	F	39	0	0	0	0	0	0	0	0	0	0	1	0	0	0	0	NA	0	0	0	0
3021	2023	8	F	39	0	0	0	0	0	0	0	0	0	0	1	0	0	0	0	NA	0	0	0	0
3022	2023	9	M	39	0	0	0	0	0	0	0	0	0	0	0	0	0	0	1	NA	0	0	0	0
3023	2023	10	M	39	0	0	0	0	0	0	0	0	0	0	0	0	1	0	0	NA	0	0	0	0
3024	2024	3	F	39	0	0	0	0	0	0	0	0	0	0	1	1	0	0	0	NA	0	0	0	0

ID	Yr	Mo	Sex	Age	Tre	Grs	Rag	Mug	HDM	Dan	Mld	Food	Drg	Ven	Rhi	Cnj	Ast	AsE	Urt	Sev	Prt	Obs	Rho	Snz
3025	2024	3	F	39	0	0	0	0	0	0	0	0	0	0	0	0	0	0	0	NA	0	0	0	0
3026	2024	3	M	39	0	0	0	0	0	0	0	0	0	0	0	0	0	0	0	1	1	0	0	0
3027	2024	4	M	39	0	0	0	0	0	0	0	0	0	0	0	0	0	0	1	NA	0	0	0	0
3028	2024	4	M	39	0	0	0	0	0	0	0	0	0	0	0	0	0	0	1	2	1	1	1	1
3029	2024	6	F	39	0	0	0	0	0	0	0	0	0	0	0	0	0	0	1	2	1	1	1	1
3030	2024	7	F	39	0	0	0	0	0	0	0	0	0	0	0	0	0	0	0	1	1	1	1	1
3031	2024	11	F	39	0	0	0	0	0	0	0	0	0	0	0	0	0	0	0	0	1	1	1	1
3032	2022	12	F	39	0	0	0	0	0	0	0	0	1	0	0	0	0	0	0	NA	0	0	0	0
3033	2021	3	M	39	0	0	0	0	1	0	0	0	0	0	1	0	0	0	1	NA	0	0	0	0
3034	2020	6	F	39	0	0	1	0	0	0	0	0	0	0	1	1	0	0	0	1	1	1	1	1
3035	2020	7	M	39	0	0	1	0	0	0	0	0	0	0	1	1	1	0	0	0	1	1	1	1
3036	2020	8	F	39	0	0	1	0	0	0	0	0	0	0	1	0	1	0	0	NA	0	0	0	0
3037	2020	8	M	39	0	0	1	0	0	0	0	0	0	0	1	1	0	0	0	1	1	1	1	1
3038	2022	10	F	39	0	0	1	0	0	0	0	0	0	0	1	0	0	0	0	2	1	0	1	1
3039	2023	9	M	39	0	0	1	0	0	0	0	0	0	0	1	1	0	0	0	2	1	1	1	1
3040	2023	10	F	39	0	0	1	0	0	0	0	0	0	0	1	1	1	0	0	2	1	1	1	1
3041	2023	11	F	39	0	0	1	0	0	0	0	0	0	0	1	0	0	0	0	NA	0	0	0	0
3042	2024	2	F	39	0	0	1	0	0	0	0	0	0	0	1	1	0	0	0	2	0	1	1	0
3043	2022	3	M	39	1	0	0	0	0	0	0	0	0	0	1	1	0	0	0	NA	0	0	0	0
3044	2023	2	F	39	0	1	0	0	0	0	0	0	0	0	1	0	1	0	0	0	0	0	0	1
3045	2023	9	F	39	0	0	0	0	1	0	0	0	0	0	1	0	1	0	0	1	1	0	1	1
3046	2021	5	F	39	0	0	1	0	0	0	0	0	0	1	0	0	0	0	0	NA	0	0	0	0
3047	2024	2	F	39	0	0	0	0	1	0	0	1	1	0	0	0	1	0	0	1	1	0	0	0
3048	2020	6	F	39	0	0	1	0	1	0	0	0	0	0	1	1	0	0	0	2	0	1	1	1
3049	2020	10	M	39	0	0	1	0	0	1	0	0	0	0	1	0	1	0	0	1	0	1	0	1
3050	2021	8	M	39	0	0	1	1	0	0	0	0	0	0	1	1	1	0	0	NA	0	0	0	0
3051	2021	9	F	39	0	0	1	0	1	0	0	0	0	0	1	1	0	0	0	1	0	1	0	0
3052	2022	5	F	39	0	0	1	0	0	1	0	0	0	0	1	1	0	0	0	0	0	0	1	0
3053	2023	7	M	39	0	0	1	1	0	0	0	0	0	0	1	0	0	0	0	NA	0	0	0	0
3054	2024	4	M	39	0	0	1	0	0	1	0	0	0	0	1	1	0	0	0	NA	0	0	0	0
3055	2024	4	M	39	0	0	1	1	0	0	0	0	0	0	1	1	1	0	0	NA	0	0	0	0
3056	2024	12	F	39	0	0	1	0	1	0	0	0	0	0	1	1	0	0	0	2	1	1	1	1
3057	2023	2	F	39	0	0	0	0	1	0	1	0	0	0	1	0	0	0	1	2	0	1	1	0
3058	2023	8	M	39	1	1	0	0	0	0	0	0	0	0	1	1	0	0	0	2	1	1	0	0
3059	2021	9	F	39	1	0	1	0	0	0	0	1	0	0	1	1	0	0	0	0	0	1	1	1
3060	2020	1	F	39	1	1	0	0	0	1	0	0	0	0	1	1	1	0	0	NA	0	0	0	0
3061	2020	11	M	39	1	1	1	0	0	0	0	0	0	0	1	1	0	0	0	NA	0	0	0	0
3062	2021	2	F	39	0	0	1	0	1	1	0	0	0	0	1	0	1	0	0	1	1	1	1	1
3063	2021	8	M	39	0	0	1	1	0	1	0	0	0	0	1	0	0	0	0	0	1	1	1	1
3064	2023	7	M	39	1	0	1	0	0	1	0	0	0	0	1	0	1	0	0	NA	0	0	0	0
3065	2021	12	F	39	0	1	1	1	0	0	0	0	0	1	1	0	0	0	0	NA	0	0	0	0
3066	2021	4	M	39	1	1	0	0	1	1	0	0	0	0	1	0	0	0	1	NA	0	0	0	0
3067	2020	2	F	39	0	0	1	1	1	0	1	0	0	0	1	0	1	0	0	2	0	0	1	1
3068	2023	7	M	39	0	1	1	1	1	0	0	0	0	0	1	1	0	0	0	2	0	1	0	0
3069	2023	12	M	39	1	1	1	1	0	0	0	0	0	0	1	1	1	0	0	NA	0	0	0	0
3070	2023	5	M	39	1	1	1	1	0	1	0	0	0	0	1	0	0	0	0	NA	0	0	0	0
3071	2021	4	M	39	1	1	1	1	0	1	0	0	0	1	0	0	0	1	0	NA	0	0	0	0
3072	2020	7	F	39	1	1	1	1	1	1	0	0	0	0	1	1	0	0	0	NA	0	0	0	0
3073	2020	3	M	40	0	0	0	0	0	0	0	0	0	0	0	0	0	0	1	NA	0	0	0	0
3074	2020	5	M	40	0	0	0	0	0	0	0	0	0	0	1	0	0	0	0	0	1	0	1	1
3075	2020	6	F	40	0	0	0	0	0	0	0	0	0	0	0	0	0	0	1	NA	0	0	0	0
3076	2020	9	F	40	0	0	0	0	0	0	0	0	0	0	1	1	0	0	0	2	1	1	1	1
3077	2020	10	M	40	0	0	0	0	0	0	0	0	0	0	0	0	0	0	0	2	0	1	1	0
3078	2020	10	M	40	0	0	0	0	0	0	0	0	0	0	1	0	0	0	0	1	0	1	1	0
3079	2020	10	M	40	0	0	0	0	0	0	0	0	0	0	0	0	0	0	0	NA	0	0	0	0
3080	2020	11	M	40	0	0	0	0	0	0	0	0	0	0	1	0	0	0	0	NA	0	0	0	0
3081	2021	2	F	40	0	0	0	0	0	0	0	0	0	0	1	0	0	0	0	NA	0	0	0	0
3082	2021	2	F	40	0	0	0	0	0	0	0	0	0	0	0	0	0	0	1	NA	0	0	0	0
3083	2021	7	F	40	0	0	0	0	0	0	0	0	0	0	1	1	1	0	0	NA	0	0	0	0
3084	2021	7	F	40	0	0	0	0	0	0	0	0	0	0	0	0	0	0	1	NA	0	0	0	0
3085	2021	8	M	40	0	0	0	0	0	0	0	0	0	0	0	0	0	0	0	NA	0	0	0	0
3086	2021	9	F	40	0	0	0	0	0	0	0	0	0	0	0	0	0	0	0	0	0	1	1	0
3087	2021	12	F	40	0	0	0	0	0	0	0	0	0	0	1	0	0	0	0	2	0	1	0	1

ID	Yr	Mo	Sex	Age	Tre	Grs	Rag	Mug	HDM	Dan	Mld	Food	Drg	Ven	Rhi	Cnj	Ast	AsE	Urt	Sev	Prt	Obs	Rho	Snz
3088	2021	2	M	40	0	0	0	0	0	0	0	0	0	0	1	0	1	0	0	NA	0	0	0	0
3089	2021	11	F	40	0	0	0	0	0	0	0	0	0	0	0	0	0	0	0	NA	0	0	0	0
3090	2021	1	M	40	0	0	0	0	0	0	0	0	0	0	1	0	0	0	0	0	0	1	0	0
3091	2021	9	F	40	0	0	0	0	0	0	0	0	0	0	0	0	0	0	0	1	0	1	1	1
3092	2021	1	F	40	0	0	0	0	0	0	0	0	0	0	0	0	0	0	1	NA	0	0	0	0
3093	2022	1	M	40	0	0	0	0	0	0	0	0	0	0	1	0	0	0	0	2	0	1	0	1
3094	2022	1	M	40	0	0	0	0	0	0	0	0	0	0	1	0	0	0	0	NA	0	0	0	0
3095	2022	2	M	40	0	0	0	0	0	0	0	0	0	0	1	0	0	0	0	NA	0	0	0	0
3096	2022	2	M	40	0	0	0	0	0	0	0	0	0	0	0	0	0	0	1	NA	0	0	0	0
3097	2022	4	M	40	0	0	0	0	0	0	0	0	0	0	1	1	0	0	0	2	1	1	1	1
3098	2022	4	M	40	0	0	0	0	0	0	0	0	0	0	1	1	0	0	0	1	1	1	1	1
3099	2022	5	F	40	0	0	0	0	0	0	0	0	0	0	1	0	0	0	1	2	1	1	1	1
3100	2022	6	F	40	0	0	0	0	0	0	0	0	0	0	0	0	0	0	1	NA	0	0	0	0
3101	2022	7	F	40	0	0	0	0	0	0	0	0	0	0	0	0	0	0	1	0	1	1	1	1
3102	2022	8	F	40	0	0	0	0	0	0	0	0	0	0	1	0	0	0	0	2	1	1	1	1
3103	2022	10	F	40	0	0	0	0	0	0	0	0	0	0	0	0	0	0	1	2	1	1	1	1
3104	2022	10	F	40	0	0	0	0	0	0	0	0	0	0	0	0	0	0	0	NA	0	0	0	0
3105	2023	3	M	40	0	0	0	0	0	0	0	0	0	0	0	0	0	0	0	NA	0	0	0	0
3106	2023	5	F	40	0	0	0	0	0	0	0	0	0	0	0	0	0	0	1	NA	0	0	0	0
3107	2023	5	F	40	0	0	0	0	0	0	0	0	0	0	1	0	1	0	0	NA	0	0	0	0
3108	2023	8	F	40	0	0	0	0	0	0	0	0	0	0	1	0	0	0	1	NA	0	0	0	0
3109	2023	10	F	40	0	0	0	0	0	0	0	0	0	0	1	0	0	0	1	2	0	1	0	0
3110	2024	2	M	40	0	0	0	0	0	0	0	0	0	0	0	0	0	0	1	1	1	0	1	0
3111	2024	3	F	40	0	0	0	0	0	0	0	0	0	0	0	0	0	0	0	2	0	1	0	0
3112	2024	4	F	40	0	0	0	0	0	0	0	0	0	0	0	0	0	0	0	1	1	0	0	0
3113	2024	6	M	40	0	0	0	0	0	0	0	0	0	0	0	0	0	0	1	2	1	1	0	1
3114	2024	10	F	40	0	0	0	0	0	0	0	0	0	0	0	0	1	0	0	NA	0	0	0	0
3115	2024	11	M	40	0	0	0	0	0	0	0	0	0	0	1	0	1	0	0	1	1	0	0	0
3116	2024	12	F	40	0	0	0	0	0	0	0	0	0	0	0	0	0	0	1	1	0	1	1	1
3117	2021	3	F	40	0	0	0	0	0	0	0	0	1	0	0	0	0	0	0	NA	0	0	0	0
3118	2021	9	M	40	0	0	0	0	0	0	0	0	0	1	1	0	0	0	0	NA	0	0	0	0
3119	2020	8	M	40	0	0	0	0	0	0	1	0	0	0	1	0	1	0	0	2	0	1	1	1
3120	2021	6	M	40	0	0	0	0	0	0	1	0	0	0	1	0	0	0	0	NA	0	0	0	0
3121	2021	3	M	40	0	0	0	0	0	0	1	0	0	0	1	0	0	0	0	NA	0	0	0	0
3122	2020	6	M	40	0	0	1	0	0	0	0	0	0	0	1	1	0	0	0	2	0	1	0	1
3123	2020	7	M	40	0	0	1	0	0	0	0	0	0	0	1	1	1	0	0	NA	0	0	0	0
3124	2020	9	M	40	0	0	1	0	0	0	0	0	0	0	1	1	1	0	0	1	1	1	1	1
3125	2021	5	M	40	0	0	1	0	0	0	0	0	0	0	1	1	1	0	0	1	1	1	1	1
3126	2023	9	M	40	0	0	1	0	0	0	0	0	0	0	1	1	0	0	0	NA	0	0	0	0
3127	2023	11	M	40	0	0	1	0	0	0	0	0	0	0	1	1	0	0	0	1	1	0	0	0
3128	2023	9	M	40	0	1	0	0	0	0	0	0	0	0	0	0	1	0	0	NA	0	0	0	0
3129	2024	5	F	40	1	0	0	0	0	0	0	0	0	0	1	1	0	0	1	2	0	0	1	0
3130	2024	5	M	40	0	0	0	0	1	0	0	0	0	0	1	0	0	0	0	NA	0	0	0	0
3131	2021	9	M	40	0	0	1	0	0	0	0	0	0	1	1	0	0	0	0	NA	0	0	0	0
3132	2021	10	F	40	0	0	1	0	0	0	0	0	0	1	1	0	0	0	0	NA	0	0	0	0
3133	2024	10	M	40	0	0	1	0	0	0	0	0	1	1	1	1	0	0	1	2	0	1	1	1
3134	2020	6	M	40	1	0	0	0	1	0	0	0	0	0	1	1	0	0	0	NA	0	0	0	0
3135	2021	12	F	40	0	1	0	0	1	0	0	0	0	0	1	0	0	0	1	NA	0	0	0	0
3136	2020	8	M	40	0	0	1	1	0	0	0	0	0	0	1	1	0	0	0	NA	0	0	0	0
3137	2021	5	M	40	0	0	1	0	0	1	0	0	0	0	1	1	1	0	0	NA	0	0	0	0
3138	2022	11	M	40	0	0	1	0	1	0	0	0	0	0	1	0	0	0	0	2	0	1	0	0
3139	2022	12	M	40	0	1	1	0	0	0	0	0	0	0	1	0	0	0	0	2	0	2	2	0
3140	2023	10	M	40	1	0	1	0	0	0	0	0	0	0	1	0	0	0	0	NA	0	0	0	0
3141	2024	4	F	40	1	0	1	0	0	0	0	0	0	0	1	1	0	0	0	2	1	1	1	1
3142	2024	10	F	40	0	0	1	1	0	0	0	0	0	0	1	1	0	0	0	2	0	0	1	1
3143	2023	1	M	40	0	0	0	0	1	1	0	0	0	0	1	0	0	0	1	2	1	0	1	1
3144	2023	1	M	40	0	1	0	0	1	0	0	0	0	0	1	0	0	0	0	0	0	0	1	1
3145	2023	7	F	40	1	1	0	0	0	0	0	0	0	0	1	0	1	0	0	NA	0	0	0	0
3146	2021	9	F	40	0	0	0	0	1	1	0	0	0	1	1	0	0	0	0	NA	0	0	0	0
3147	2021	9	M	40	0	0	1	1	0	0	0	0	0	1	1	0	0	0	0	0	1	1	1	1
3148	2021	6	F	40	0	0	1	0	1	0	0	0	1	1	1	0	0	0	0	2	0	1	1	1
3149	2024	4	F	40	1	1	1	0	0	0	0	0	0	0	1	1	0	0	0	2	0	0	1	0
3150	2024	9	M	40	0	0	1	1	1	0	0	0	0	0	1	1	1	0	0	1	1	0	1	1

ID	Yr	Mo	Sex	Age	Tre	Grs	Rag	Mug	HDM	Dan	Mld	Food	Drg	Ven	Rhi	Cnj	Ast	AsE	Urt	Sev	Prt	Obs	Rho	Snz
3151	2024	7	F	40	1	1	0	0	0	1	0	0	0	0	1	0	0	0	1	NA	0	0	0	0
3152	2023	9	F	40	0	1	1	0	0	1	1	0	0	0	1	0	0	0	0	NA	0	0	0	0
3153	2022	10	F	40	1	0	0	0	1	1	1	0	0	0	1	0	1	0	0	NA	0	0	0	0
3154	2021	11	M	40	0	1	1	1	1	0	0	0	0	1	1	1	0	0	0	NA	0	0	0	0
3155	2021	12	M	40	0	1	1	1	0	1	1	0	0	1	0	0	0	0	0	NA	0	0	0	0
3156	2020	12	F	40	1	1	1	1	1	1	1	0	0	0	1	1	1	0	0	2	1	1	1	0
3157	2020	1	F	41	0	0	0	0	0	0	0	0	0	0	1	0	0	0	0	2	1	0	0	0
3158	2020	2	M	41	0	0	0	0	0	0	0	0	0	0	1	1	0	0	0	2	0	1	1	1
3159	2020	2	M	41	0	0	0	0	0	0	0	0	0	0	0	0	0	0	0	NA	0	0	0	0
3160	2020	6	M	41	0	0	0	0	0	0	0	0	0	0	0	0	1	0	0	1	1	1	1	1
3161	2020	7	F	41	0	0	0	0	0	0	0	0	0	0	0	0	0	0	1	2	0	1	0	1
3162	2020	7	F	41	0	0	0	0	0	0	0	0	0	0	1	0	0	0	1	NA	0	0	0	0
3163	2020	12	F	41	0	0	0	0	0	0	0	0	0	0	0	0	0	0	1	NA	0	0	0	0
3164	2021	5	F	41	0	0	0	0	0	0	0	0	0	0	1	1	0	0	0	2	0	0	1	0
3165	2021	9	F	41	0	0	0	0	0	0	0	0	0	0	1	1	0	0	0	0	1	1	1	1
3166	2021	9	F	41	0	0	0	0	0	0	0	0	0	0	0	0	0	0	0	1	0	1	0	1
3167	2021	12	F	41	0	0	0	0	0	0	0	0	0	0	1	0	0	0	0	NA	0	0	0	0
3168	2021	3	M	41	0	0	0	0	0	0	0	0	0	0	0	0	0	0	0	NA	0	0	0	0
3169	2022	1	F	41	0	0	0	0	0	0	0	0	0	0	1	0	1	0	0	0	1	1	1	1
3170	2022	1	M	41	0	0	0	0	0	0	0	0	0	0	0	0	0	0	0	2	0	1	0	1
3171	2022	3	F	41	0	0	0	0	0	0	0	0	0	0	0	0	0	0	1	NA	0	0	0	0
3172	2022	3	M	41	0	0	0	0	0	0	0	0	0	0	0	0	0	0	0	1	1	1	1	1
3173	2022	5	F	41	0	0	0	0	0	0	0	0	0	0	1	0	1	0	0	2	0	1	1	1
3174	2022	9	F	41	0	0	0	0	0	0	0	0	0	0	0	0	0	0	0	NA	0	0	0	0
3175	2022	9	F	41	0	0	0	0	0	0	0	0	0	0	0	0	0	0	1	NA	0	0	0	0
3176	2022	9	F	41	0	0	0	0	0	0	0	0	0	0	1	0	0	0	0	NA	0	0	0	0
3177	2022	10	M	41	0	0	0	0	0	0	0	0	0	0	0	0	1	0	0	2	1	1	0	0
3178	2023	1	M	41	0	0	0	0	0	0	0	0	0	0	0	0	1	0	0	NA	0	0	0	0
3179	2023	3	F	41	0	0	0	0	0	0	0	0	0	0	1	0	0	0	1	2	0	1	0	0
3180	2023	4	F	41	0	0	0	0	0	0	0	0	0	0	1	0	1	0	0	NA	0	0	0	0
3181	2023	5	M	41	0	0	0	0	0	0	0	0	0	0	1	0	0	0	0	0	0	0	1	0
3182	2023	5	M	41	0	0	0	0	0	0	0	0	0	0	1	0	0	0	0	2	1	1	1	1
3183	2023	8	F	41	0	0	0	0	0	0	0	0	0	0	1	0	0	0	1	2	1	1	0	1
3184	2023	8	F	41	0	0	0	0	0	0	0	0	0	0	1	0	1	0	0	2	0	1	1	1
3185	2023	12	F	41	0	0	0	0	0	0	0	0	0	0	0	0	0	0	1	1	1	0	1	0
3186	2024	1	M	41	0	0	0	0	0	0	0	0	0	0	1	0	0	0	0	NA	0	0	0	0
3187	2024	4	M	41	0	0	0	0	0	0	0	0	0	0	0	0	0	0	0	1	1	0	0	1
3188	2024	4	F	41	0	0	0	0	0	0	0	0	0	0	0	0	0	0	0	NA	0	0	0	0
3189	2024	5	F	41	0	0	0	0	0	0	0	0	0	0	0	0	0	0	0	NA	0	0	0	0
3190	2024	5	M	41	0	0	0	0	0	0	0	0	0	0	1	0	0	0	0	0	1	1	1	1
3191	2024	5	M	41	0	0	0	0	0	0	0	0	0	0	0	0	0	0	1	1	1	0	0	0
3192	2024	6	M	41	0	0	0	0	0	0	0	0	0	0	1	0	0	0	0	1	1	1	1	1
3193	2024	7	F	41	0	0	0	0	0	0	0	0	0	0	0	0	0	0	1	2	1	0	0	0
3194	2024	12	M	41	0	0	0	0	0	0	0	0	0	0	0	0	0	0	0	0	1	1	1	1
3195	2024	12	M	41	0	0	0	0	0	0	0	0	0	0	0	0	0	0	1	2	1	1	0	1
3196	2021	12	F	41	0	0	0	0	0	0	0	0	1	0	0	0	1	0	1	1	0	1	0	0
3197	2021	9	M	41	0	0	0	0	0	0	0	0	0	1	1	0	0	0	0	NA	0	0	0	0
3198	2021	12	M	41	0	0	0	0	0	0	0	0	0	1	0	0	0	0	0	2	1	1	0	1
3199	2021	10	F	41	0	0	0	0	0	0	0	0	0	1	0	0	0	1	0	1	1	1	0	0
3200	2021	4	F	41	0	0	0	0	0	0	0	0	1	0	0	0	0	1	0	0	0	1	1	1
3201	2023	8	F	41	0	0	0	0	0	0	0	0	0	1	1	0	0	0	0	1	0	1	0	0
3202	2020	1	F	41	0	0	1	0	0	0	0	0	0	0	1	1	1	0	0	NA	0	0	0	0
3203	2020	1	F	41	0	0	1	0	0	0	0	0	0	0	1	1	1	0	0	2	0	1	1	1
3204	2020	2	M	41	0	0	1	0	0	0	0	0	0	0	1	1	0	0	0	NA	0	0	0	0
3205	2021	4	M	41	0	0	1	0	0	0	0	0	0	0	0	0	0	0	1	1	0	1	1	0
3206	2021	5	M	41	0	0	1	0	0	0	0	0	0	0	1	1	1	0	0	0	1	0	0	1
3207	2022	8	M	41	0	0	1	0	0	0	0	0	0	0	1	0	0	0	0	NA	0	0	0	0
3208	2023	2	M	41	0	0	1	0	0	0	0	0	0	0	1	0	0	0	1	2	0	1	1	0
3209	2023	8	M	41	0	0	1	0	0	0	0	0	0	0	1	1	0	0	0	1	0	1	0	0
3210	2023	9	M	41	0	0	1	0	0	0	0	0	0	0	1	1	0	0	0	2	0	1	1	0
3211	2024	1	M	41	0	0	1	0	0	0	0	0	0	0	1	1	0	0	0	NA	0	0	0	0
3212	2021	1	F	41	0	0	1	0	0	0	0	0	0	0	1	0	1	0	0	2	1	0	0	1
3213	2021	2	M	41	0	0	1	0	0	0	0	0	0	0	1	1	0	0	0	1	0	1	0	0

ID	Yr	Mo	Sex	Age	Tre	Grs	Rag	Mug	HDM	Dan	Mld	Food	Drg	Ven	Rhi	Cnj	Ast	AsE	Urt	Sev	Prt	Obs	Rho	Snz
3214	2020	1	M	41	0	0	1	0	0	1	0	0	0	0	1	0	0	0	0	NA	0	0	0	0
3215	2021	9	M	41	0	0	1	0	1	0	0	0	0	0	1	1	0	0	0	2	0	1	1	1
3216	2022	4	F	41	0	1	1	0	0	0	0	0	0	0	1	1	0	0	0	2	0	0	1	0
3217	2024	11	M	41	0	1	1	0	0	0	0	0	0	0	1	1	1	0	0	2	0	1	1	1
3218	2022	5	M	41	0	1	0	0	1	0	0	0	0	0	1	1	0	0	0	NA	0	0	0	0
3219	2023	11	F	41	0	1	0	0	1	0	0	0	0	0	1	1	1	0	0	NA	0	0	0	0
3220	2024	10	M	41	0	1	1	0	0	0	0	1	0	0	1	1	1	0	0	NA	0	0	0	0
3221	2021	12	M	41	0	0	1	0	1	0	0	0	0	1	1	1	0	0	0	1	1	1	1	1
3222	2021	5	M	41	0	0	1	0	1	0	0	0	0	1	0	0	0	0	0	2	0	0	1	1
3223	2021	9	F	41	0	0	1	0	0	1	0	0	0	1	1	1	0	0	0	NA	0	0	0	0
3224	2020	2	M	41	0	1	1	0	1	0	0	0	0	0	1	0	0	0	0	1	0	1	1	0
3225	2021	6	F	41	0	1	1	0	0	0	1	0	0	0	1	1	0	0	0	NA	0	0	0	0
3226	2021	9	M	41	0	0	1	1	1	0	0	0	0	0	1	1	0	0	0	2	0	1	1	1
3227	2022	8	F	41	0	1	1	0	1	0	0	0	0	0	1	1	0	0	0	2	0	0	1	1
3228	2022	9	M	41	0	0	1	1	1	0	0	0	0	0	1	0	1	0	0	2	1	1	0	1
3229	2024	6	F	41	1	1	1	0	0	0	0	0	0	0	1	1	0	0	0	2	0	0	0	1
3230	2024	7	F	41	1	1	1	0	0	0	0	0	0	0	1	1	0	0	0	NA	0	0	0	0
3231	2022	5	F	41	1	0	0	0	1	1	0	0	0	0	1	0	0	0	1	NA	0	0	0	0
3232	2023	7	M	41	0	1	0	0	1	1	0	0	0	0	1	0	0	0	0	NA	0	0	0	0
3233	2021	2	M	41	1	1	0	1	0	0	0	0	1	0	1	0	1	0	0	NA	0	0	0	0
3234	2023	7	F	41	0	1	1	0	0	1	0	1	0	0	1	0	0	0	1	2	1	1	1	1
3235	2021	9	M	41	0	0	1	0	1	1	0	0	0	1	0	0	0	0	0	NA	0	0	0	0
3236	2020	2	M	41	0	1	0	0	1	1	1	0	0	0	1	0	1	0	0	2	1	1	0	1
3237	2021	10	M	41	1	1	1	1	1	1	0	0	0	1	1	1	0	0	0	1	1	1	1	0
3238	2020	1	F	42	0	0	0	0	0	0	0	0	0	0	0	0	1	0	1	1	0	1	1	1
3239	2020	2	M	42	0	0	0	0	0	0	0	0	0	0	0	0	0	0	0	2	0	1	0	0
3240	2020	2	F	42	0	0	0	0	0	0	0	0	0	0	1	0	0	0	1	NA	0	0	0	0
3241	2020	2	M	42	0	0	0	0	0	0	0	0	0	0	1	0	0	0	0	2	0	1	0	0
3242	2020	3	M	42	0	0	0	0	0	0	0	0	0	0	1	0	0	0	0	NA	0	0	0	0
3243	2020	6	F	42	0	0	0	0	0	0	0	0	0	0	1	0	1	0	0	NA	0	0	0	0
3244	2020	7	F	42	0	0	0	0	0	0	0	0	0	0	1	1	1	0	0	0	0	0	1	1
3245	2020	7	F	42	0	0	0	0	0	0	0	0	0	0	0	0	0	0	1	2	0	0	1	1
3246	2020	7	F	42	0	0	0	0	0	0	0	0	0	0	1	1	0	0	0	NA	0	0	0	0
3247	2020	11	F	42	0	0	0	0	0	0	0	0	0	0	0	0	1	0	0	2	0	1	1	1
3248	2020	11	M	42	0	0	0	0	0	0	0	0	0	0	1	0	0	0	0	NA	0	0	0	0
3249	2021	3	F	42	0	0	0	0	0	0	0	0	0	0	1	0	0	0	0	NA	0	0	0	0
3250	2021	4	F	42	0	0	0	0	0	0	0	0	0	0	0	0	0	0	1	NA	0	0	0	0
3251	2021	7	F	42	0	0	0	0	0	0	0	0	0	0	0	0	1	0	0	2	1	1	1	1
3252	2021	8	M	42	0	0	0	0	0	0	0	0	0	0	1	0	0	0	0	NA	0	0	0	0
3253	2021	11	F	42	0	0	0	0	0	0	0	0	0	0	0	0	0	0	1	2	0	1	0	0
3254	2022	1	F	42	0	0	0	0	0	0	0	0	0	0	1	0	0	0	0	NA	0	0	0	0
3255	2022	1	F	42	0	0	0	0	0	0	0	0	0	0	0	0	0	0	1	1	1	0	0	1
3256	2022	3	M	42	0	0	0	0	0	0	0	0	0	0	0	0	0	0	1	NA	0	0	0	0
3257	2022	6	F	42	0	0	0	0	0	0	0	0	0	0	0	0	0	0	1	NA	0	0	0	0
3258	2022	9	M	42	0	0	0	0	0	0	0	0	0	0	0	0	0	0	0	2	1	1	0	1
3259	2022	10	F	42	0	0	0	0	0	0	0	0	0	0	0	0	0	0	0	NA	0	0	0	0
3260	2023	2	F	42	0	0	0	0	0	0	0	0	0	0	0	0	0	0	0	1	0	1	0	0
3261	2023	5	M	42	0	0	0	0	0	0	0	0	0	0	1	0	1	0	0	1	0	0	1	1
3262	2023	6	F	42	0	0	0	0	0	0	0	0	0	0	1	0	0	0	0	0	1	1	1	1
3263	2023	8	F	42	0	0	0	0	0	0	0	0	0	0	1	0	0	0	0	2	0	1	1	1
3264	2023	8	F	42	0	0	0	0	0	0	0	0	0	0	0	0	0	0	1	2	0	1	1	0
3265	2023	10	F	42	0	0	0	0	0	0	0	0	0	0	0	0	0	0	1	NA	0	0	0	0
3266	2023	11	F	42	0	0	0	0	0	0	0	0	0	0	0	0	0	0	0	NA	0	0	0	0
3267	2024	3	F	42	0	0	0	0	0	0	0	0	0	0	0	0	0	0	0	NA	0	0	0	0
3268	2024	4	M	42	0	0	0	0	0	0	0	0	0	0	0	0	0	0	1	2	1	1	0	0
3269	2024	5	F	42	0	0	0	0	0	0	0	0	0	0	0	0	0	0	1	NA	0	0	0	0
3270	2024	7	F	42	0	0	0	0	0	0	0	0	0	0	0	0	0	0	1	NA	0	0	0	0
3271	2024	7	F	42	0	0	0	0	0	0	0	0	0	0	0	0	1	0	0	NA	0	0	0	0
3272	2024	10	M	42	0	0	0	0	0	0	0	0	0	0	0	0	0	0	1	1	0	1	1	0
3273	2024	10	M	42	0	0	0	0	0	0	0	0	0	0	0	0	0	0	1	1	1	0	0	0
3274	2024	11	F	42	0	0	0	0	0	0	0	0	0	0	0	0	0	0	1	2	1	0	1	1
3275	2020	1	M	42	0	0	0	0	0	0	0	0	1	0	0	0	0	0	0	NA	0	0	0	0
3276	2020	8	F	42	0	0	0	0	0	0	0	1	0	0	0	0	0	0	0	NA	0	0	0	0

ID	Yr	Mo	Sex	Age	Tre	Grs	Rag	Mug	HDM	Dan	Mld	Food	Drg	Ven	Rhi	Cnj	Ast	AsE	Urt	Sev	Prt	Obs	Rho	Snz
3277	2021	3	F	42	0	0	0	0	0	0	0	0	1	0	0	0	0	0	0	2	0	1	0	0
3278	2021	4	M	42	0	0	0	0	0	0	0	0	0	1	0	0	0	1	0	NA	0	0	0	0
3279	2022	9	F	42	0	0	0	0	0	0	0	1	0	0	1	1	0	0	0	2	1	1	1	0
3280	2022	6	F	42	0	0	0	0	0	0	0	0	1	1	0	0	0	0	1	1	1	1	1	1
3281	2020	2	M	42	0	0	0	0	0	0	1	0	0	0	1	0	0	0	0	NA	0	0	0	0
3282	2021	12	F	42	0	0	0	0	1	0	0	0	0	0	1	0	0	0	0	NA	0	0	0	0
3283	2020	3	M	42	0	0	1	0	0	0	0	0	0	0	1	1	1	0	0	2	1	1	0	0
3284	2020	9	F	42	0	0	1	0	0	0	0	0	0	0	1	1	0	0	0	1	1	1	1	1
3285	2022	7	F	42	0	0	1	0	0	0	0	0	0	0	1	0	0	0	0	0	1	1	1	1
3286	2022	8	M	42	0	0	1	0	0	0	0	0	0	0	1	1	0	0	0	2	1	1	1	1
3287	2022	8	M	42	0	0	1	0	0	0	0	0	0	0	1	0	0	0	0	0	1	1	1	1
3288	2023	8	F	42	0	0	1	0	0	0	0	0	0	0	1	0	0	0	0	NA	0	0	0	0
3289	2023	9	M	42	0	0	1	0	0	0	0	0	0	0	1	0	0	0	0	1	0	1	0	1
3290	2023	9	F	42	0	0	1	0	0	0	0	0	0	0	1	0	0	0	0	2	1	0	0	1
3291	2023	11	M	42	0	0	1	0	0	0	0	0	0	0	1	1	0	0	0	NA	0	0	0	0
3292	2024	8	F	42	0	0	1	0	0	0	0	0	0	0	1	1	0	0	0	1	1	1	1	1
3293	2021	2	M	42	0	0	1	0	0	0	0	0	0	0	1	0	1	0	1	NA	0	0	0	0
3294	2022	6	F	42	0	0	0	0	0	0	1	0	0	0	0	0	0	0	0	NA	0	0	0	0
3295	2022	6	F	42	0	0	0	0	0	0	1	0	0	0	1	0	0	0	0	NA	0	0	0	0
3296	2023	10	F	42	0	1	0	0	0	0	0	0	0	0	1	0	0	0	0	1	1	1	1	1
3297	2023	12	M	42	0	0	0	0	1	0	0	0	0	0	0	0	1	0	1	1	1	0	1	1
3298	2021	9	M	42	0	0	1	0	0	0	0	0	0	1	1	0	0	0	0	2	1	1	1	1
3299	2021	11	F	42	0	0	1	0	0	0	0	0	0	1	0	1	0	0	0	1	1	1	1	1
3300	2023	5	F	42	0	0	0	0	0	1	0	0	1	0	1	0	0	0	1	NA	0	0	0	0
3301	2021	11	F	42	1	1	0	0	0	0	0	0	0	0	0	1	1	0	1	1	1	1	1	1
3302	2020	6	F	42	0	0	1	0	1	0	0	0	0	0	1	1	0	0	0	1	1	1	1	1
3303	2021	4	M	42	0	0	1	0	1	0	0	0	0	0	1	1	0	0	0	2	0	1	0	0
3304	2022	9	M	42	0	1	1	0	0	0	0	0	0	0	1	0	0	0	0	0	1	1	1	1
3305	2022	3	F	42	0	0	0	0	0	1	1	0	0	0	1	0	0	0	1	1	1	1	1	1
3306	2021	6	M	42	0	1	0	0	1	0	0	0	1	1	0	0	0	0	1	2	1	1	1	1
3307	2024	10	F	42	1	0	1	0	0	0	0	0	1	1	1	1	1	0	0	0	1	1	1	1
3308	2020	3	M	42	1	1	0	0	1	0	0	0	0	0	1	1	0	0	0	2	0	1	0	0
3309	2021	11	F	42	0	1	0	0	1	1	0	0	0	0	1	0	1	0	0	0	1	1	1	1
3310	2023	6	M	42	0	1	1	0	0	0	1	0	0	0	1	0	1	0	1	NA	0	0	0	0
3311	2024	9	F	42	0	1	1	1	0	0	0	0	0	0	1	1	0	0	0	NA	0	0	0	0
3312	2024	9	F	42	1	0	1	1	0	0	0	0	0	0	1	0	0	0	0	NA	0	0	0	0
3313	2020	7	F	42	1	0	0	1	1	0	0	0	0	1	1	1	0	0	0	1	1	1	1	1
3314	2023	7	M	42	1	1	1	1	0	0	1	0	0	0	1	0	0	0	0	2	0	0	1	1
3315	2024	10	M	42	1	1	1	1	0	1	0	1	0	0	1	1	0	0	0	2	0	2	2	0
3316	2020	1	M	43	0	0	0	0	0	0	0	0	0	0	0	0	0	0	0	NA	0	0	0	0
3317	2020	1	F	43	0	0	0	0	0	0	0	0	0	0	1	0	0	0	0	NA	0	0	0	0
3318	2020	1	F	43	0	0	0	0	0	0	0	0	0	0	0	0	0	0	0	NA	0	0	0	0
3319	2020	2	F	43	0	0	0	0	0	0	0	0	0	0	0	1	0	0	0	2	0	1	0	0
3320	2020	3	F	43	0	0	0	0	0	0	0	0	0	0	1	0	0	0	0	2	1	1	1	1
3321	2020	7	F	43	0	0	0	0	0	0	0	0	0	0	0	0	0	0	0	2	0	0	1	1
3322	2020	7	F	43	0	0	0	0	0	0	0	0	0	0	0	0	0	0	0	NA	0	0	0	0
3323	2020	7	M	43	0	0	0	0	0	0	0	0	0	0	0	0	0	0	1	2	0	1	0	1
3324	2020	8	F	43	0	0	0	0	0	0	0	0	0	0	0	0	0	0	1	1	1	0	1	1
3325	2020	8	F	43	0	0	0	0	0	0	0	0	0	0	0	0	1	0	0	NA	0	0	0	0
3326	2020	9	F	43	0	0	0	0	0	0	0	0	0	0	1	0	0	0	0	NA	0	0	0	0
3327	2020	10	F	43	0	0	0	0	0	0	0	0	0	0	0	0	0	0	0	NA	0	0	0	0
3328	2020	10	F	43	0	0	0	0	0	0	0	0	0	0	0	0	0	0	0	NA	0	0	0	0
3329	2020	12	F	43	0	0	0	0	0	0	0	0	0	0	1	0	1	0	0	1	0	1	0	1
3330	2021	1	F	43	0	0	0	0	0	0	0	0	0	0	0	0	0	0	1	1	1	0	0	0
3331	2021	1	F	43	0	0	0	0	0	0	0	0	0	0	0	0	0	0	1	NA	0	0	0	0
3332	2021	7	M	43	0	0	0	0	0	0	0	0	0	0	0	0	0	0	1	1	1	1	1	1
3333	2021	7	F	43	0	0	0	0	0	0	0	0	0	0	0	0	1	0	0	2	1	0	1	1
3334	2021	10	F	43	0	0	0	0	0	0	0	0	0	0	0	0	0	0	0	NA	0	0	0	0
3335	2021	9	F	43	0	0	0	0	0	0	0	0	0	0	0	0	0	1	0	1	1	1	1	1
3336	2021	2	F	43	0	0	0	0	0	0	0	0	0	0	1	0	0	0	0	NA	0	0	0	0
3337	2021	11	F	43	0	0	0	0	0	0	0	0	0	0	0	0	0	1	0	0	1	1	1	1
3338	2021	5	F	43	0	0	0	0	0	0	0	0	0	0	0	0	0	1	0	2	0	0	1	0
3339	2021	5	F	43	0	0	0	0	0	0	0	0	0	0	0	0	0	1	0	1	0	1	1	1

ID	Yr	Mo	Sex	Age	Tre	Grs	Rag	Mug	HDM	Dan	Mld	Food	Drg	Ven	Rhi	Cnj	Ast	AsE	Urt	Sev	Prt	Obs	Rho	Snz
3340	2022	1	M	43	0	0	0	0	0	0	0	0	0	0	0	0	0	0	0	NA	0	0	0	0
3341	2022	4	M	43	0	0	0	0	0	0	0	0	0	0	0	0	0	0	0	2	0	1	1	0
3342	2023	2	F	43	0	0	0	0	0	0	0	0	0	0	1	0	1	0	1	NA	0	0	0	0
3343	2023	6	F	43	0	0	0	0	0	0	0	0	0	0	0	0	0	0	1	NA	0	0	0	0
3344	2023	10	M	43	0	0	0	0	0	0	0	0	0	0	1	0	0	0	0	0	1	0	1	0
3345	2023	10	F	43	0	0	0	0	0	0	0	0	0	0	0	0	1	0	0	2	0	1	0	1
3346	2024	4	F	43	0	0	0	0	0	0	0	0	0	0	0	0	0	0	0	NA	0	0	0	0
3347	2024	5	F	43	0	0	0	0	0	0	0	0	0	0	0	0	1	0	0	NA	0	0	0	0
3348	2024	7	F	43	0	0	0	0	0	0	0	0	0	0	0	0	0	0	1	2	0	1	1	1
3349	2024	9	F	43	0	0	0	0	0	0	0	0	0	0	0	0	0	0	1	NA	0	0	0	0
3350	2024	10	F	43	0	0	0	0	0	0	0	0	0	0	0	0	0	0	0	2	0	1	0	1
3351	2024	10	M	43	0	0	0	0	0	0	0	0	0	0	0	0	1	0	0	2	0	1	0	0
3352	2024	12	F	43	0	0	0	0	0	0	0	0	0	0	0	0	1	1	0	NA	0	0	0	0
3353	2020	9	M	43	0	0	0	0	0	0	0	0	0	1	0	0	0	0	0	NA	0	0	0	0
3354	2021	6	F	43	0	0	0	0	0	0	0	0	1	0	0	0	0	0	0	NA	0	0	0	0
3355	2022	2	M	43	0	0	0	0	0	0	0	0	1	0	0	0	0	0	1	2	0	1	0	0
3356	2023	5	F	43	0	0	0	0	0	0	0	0	0	1	0	0	0	0	1	1	1	1	1	1
3357	2023	5	F	43	0	0	0	0	0	0	0	0	1	0	0	0	0	0	1	2	1	1	1	1
3358	2024	5	F	43	0	0	0	0	0	0	0	0	1	0	0	0	0	0	0	NA	0	0	0	0
3359	2020	1	M	43	0	0	0	0	1	0	0	0	0	0	1	0	0	0	0	NA	0	0	0	0
3360	2021	2	M	43	0	0	0	0	1	0	0	0	0	0	0	0	0	0	1	1	1	1	1	1
3361	2021	5	F	43	0	0	0	0	1	0	0	0	0	0	1	0	0	0	0	NA	0	0	0	0
3362	2021	7	F	43	0	0	0	0	1	0	0	0	0	0	1	0	0	0	1	1	0	1	0	0
3363	2021	9	F	43	0	0	0	0	0	1	0	0	0	0	1	1	1	0	0	NA	0	0	0	0
3364	2021	3	M	43	1	0	0	0	0	0	0	0	0	0	1	1	0	0	0	NA	0	1	1	1
3365	2020	7	M	43	0	0	1	0	0	0	0	0	0	0	1	1	0	0	1	2	1	1	1	1
3366	2020	7	M	43	0	0	1	0	0	0	0	0	0	0	1	1	0	0	0	NA	0	0	0	0
3367	2020	8	F	43	0	0	1	0	0	0	0	0	0	0	1	0	0	0	1	1	1	1	0	1
3368	2022	7	M	43	0	0	1	0	0	0	0	0	0	0	1	1	1	0	0	2	0	1	1	1
3369	2022	10	M	43	0	0	1	0	0	0	0	0	0	0	1	1	0	0	0	NA	0	0	0	0
3370	2023	2	F	43	0	0	1	0	0	0	0	0	0	0	1	0	0	0	0	2	1	1	1	1
3371	2023	3	F	43	0	0	1	0	0	0	0	0	0	0	1	1	1	0	0	2	0	1	0	0
3372	2023	8	F	43	0	0	1	0	0	0	0	0	0	0	1	1	1	0	0	NA	0	0	0	0
3373	2024	2	F	43	0	0	1	0	0	0	0	0	0	0	1	1	0	0	1	1	0	1	0	0
3374	2024	6	F	43	0	0	1	0	0	0	0	0	0	0	1	1	0	0	0	NA	0	0	0	0
3375	2022	10	M	43	0	0	0	0	0	0	1	0	0	0	1	0	0	0	0	2	1	0	1	1
3376	2022	12	F	43	0	0	0	0	1	0	0	0	0	0	1	0	1	0	0	NA	0	0	0	0
3377	2021	5	M	43	0	1	0	0	0	0	0	0	0	1	1	0	0	0	0	2	1	1	1	1
3378	2020	8	M	43	0	0	0	1	0	1	0	0	0	0	1	1	0	0	0	NA	0	0	0	0
3379	2021	2	F	43	0	0	0	0	1	0	1	0	0	0	1	0	0	0	0	0	1	1	1	1
3380	2020	9	M	43	1	0	1	0	0	0	0	0	0	0	1	1	0	0	0	NA	0	0	0	0
3381	2023	6	F	43	0	1	1	0	0	0	0	0	0	0	1	0	1	0	0	NA	0	0	0	0
3382	2024	3	F	43	0	0	1	0	0	1	0	0	0	0	0	0	0	0	0	2	1	1	0	1
3383	2024	4	F	43	0	1	1	0	0	0	0	0	0	0	1	1	0	0	0	0	0	0	0	1
3384	2024	6	M	43	0	0	1	0	1	0	0	0	0	0	1	1	0	0	0	2	1	1	1	1
3385	2023	4	F	43	0	0	0	0	1	1	0	0	0	0	0	0	0	0	1	NA	0	0	0	0
3386	2024	7	F	43	0	1	0	0	0	1	0	0	0	0	1	1	0	0	0	NA	0	0	0	0
3387	2020	7	M	43	0	1	0	0	1	0	1	0	0	0	1	0	0	0	0	NA	0	1	0	0
3388	2020	5	F	43	0	0	1	1	1	0	0	0	0	0	1	0	1	0	0	NA	0	0	0	0
3389	2021	3	M	43	0	1	1	0	1	0	0	0	0	0	1	1	1	0	0	1	0	1	0	0
3390	2022	2	M	43	0	1	1	0	1	0	0	0	0	0	1	1	0	0	0	NA	0	0	0	0
3391	2022	9	F	43	1	1	1	0	0	0	0	0	0	0	1	1	0	0	0	2	1	1	1	0
3392	2023	5	M	43	1	1	1	0	0	0	0	0	0	0	1	0	0	0	0	2	0	1	1	0
3393	2023	8	F	43	0	1	1	0	1	0	0	0	0	0	1	1	0	0	0	NA	0	0	0	0
3394	2023	6	F	43	1	0	0	0	1	0	1	0	0	0	1	0	1	0	0	2	0	1	0	0
3395	2021	10	M	43	1	1	1	1	0	0	0	0	0	0	1	1	0	0	1	1	0	0	0	0
3396	2022	6	F	43	0	1	1	0	1	1	0	0	0	0	0	0	0	0	1	2	1	1	0	0
3397	2020	1	F	44	0	0	0	0	0	0	0	0	0	0	0	0	1	0	0	NA	0	0	0	0
3398	2020	2	F	44	0	0	0	0	0	0	0	0	0	0	0	0	0	0	1	2	0	1	1	0
3399	2020	2	M	44	0	0	0	0	0	0	0	0	0	0	1	0	0	0	1	2	0	1	0	0
3400	2020	6	M	44	0	0	0	0	0	0	0	0	0	0	0	0	0	0	0	NA	0	0	0	0
3401	2020	7	M	44	0	0	0	0	0	0	0	0	0	0	1	0	0	0	0	2	0	1	1	0
3402	2020	7	F	44	0	0	0	0	0	0	0	0	0	0	0	0	0	0	1	NA	0	0	0	0

ID	Yr	Mo	Sex	Age	Tre	Grs	Rag	Mug	HDM	Dan	Mld	Food	Drg	Ven	Rhi	Cnj	Ast	AsE	Urt	Sev	Prt	Obs	Rho	Snz
3403	2021	1	F	44	0	0	0	0	0	0	0	0	0	0	0	0	1	1	0	2	0	1	0	0
3404	2021	3	F	44	0	0	0	0	0	0	0	0	0	0	0	0	0	0	0	NA	0	0	0	0
3405	2021	9	F	44	0	0	0	0	0	0	0	0	0	0	1	1	1	0	0	2	0	1	1	1
3406	2021	2	F	44	0	0	0	0	0	0	0	0	0	0	0	0	1	0	1	NA	0	0	0	0
3407	2021	4	F	44	0	0	0	0	0	0	0	0	0	0	0	1	0	1	0	NA	0	0	0	0
3408	2022	1	F	44	0	0	0	0	0	0	0	0	0	0	0	0	0	0	1	NA	0	0	0	0
3409	2022	1	F	44	0	0	0	0	0	0	0	0	0	0	0	0	0	0	0	2	0	0	0	0
3410	2022	1	M	44	0	0	0	0	0	0	0	0	0	0	0	0	0	0	1	2	1	1	1	1
3411	2022	1	F	44	0	0	0	0	0	0	0	0	0	0	1	0	1	0	0	NA	0	1	1	1
3412	2022	1	F	44	0	0	0	0	0	0	0	0	0	0	0	0	0	0	1	NA	0	0	0	0
3413	2022	2	F	44	0	0	0	0	0	0	0	0	0	0	0	0	1	0	1	NA	0	0	0	0
3414	2022	3	F	44	0	0	0	0	0	0	0	0	0	0	1	0	0	0	0	NA	0	0	0	0
3415	2022	4	F	44	0	0	0	0	0	0	0	0	0	0	0	0	0	0	1	NA	0	0	0	0
3416	2022	6	F	44	0	0	0	0	0	0	0	0	0	0	0	0	0	0	1	NA	0	0	0	0
3417	2022	6	M	44	0	0	0	0	0	0	0	0	0	0	1	1	0	0	0	2	0	0	0	0
3418	2022	7	F	44	0	0	0	0	0	0	0	0	0	0	0	0	0	0	0	1	1	0	1	1
3419	2022	7	F	44	0	0	0	0	0	0	0	0	0	0	0	0	0	0	1	NA	0	0	0	0
3420	2022	10	F	44	0	0	0	0	0	0	0	0	0	0	0	0	1	0	0	1	0	1	0	0
3421	2022	10	F	44	0	0	0	0	0	0	0	0	0	0	0	0	1	0	1	2	0	1	1	1
3422	2023	5	F	44	0	0	0	0	0	0	0	0	0	0	0	0	0	0	1	1	0	1	0	0
3423	2023	8	M	44	0	0	0	0	0	0	0	0	0	0	0	0	1	0	1	NA	0	0	0	0
3424	2023	11	F	44	0	0	0	0	0	0	0	0	0	0	0	0	0	0	1	1	0	1	0	0
3425	2023	11	F	44	0	0	0	0	0	0	0	0	0	0	1	0	0	0	0	1	0	1	0	0
3426	2023	11	F	44	0	0	0	0	0	0	0	0	0	0	0	0	0	0	1	NA	0	0	0	0
3427	2024	3	F	44	0	0	0	0	0	0	0	0	0	0	0	0	0	0	0	NA	0	0	0	0
3428	2024	5	M	44	0	0	0	0	0	0	0	0	0	0	0	0	0	0	1	NA	0	0	0	0
3429	2024	7	M	44	0	0	0	0	0	0	0	0	0	0	0	0	0	0	0	2	0	1	0	0
3430	2024	8	F	44	0	0	0	0	0	0	0	0	0	0	0	0	0	0	0	NA	0	0	0	0
3431	2024	10	F	44	0	0	0	0	0	0	0	0	0	0	0	0	0	0	0	2	0	1	0	0
3432	2024	12	M	44	0	0	0	0	0	0	0	0	0	0	1	1	0	0	0	NA	0	0	0	0
3433	2024	12	F	44	0	0	0	0	0	0	0	0	0	0	0	0	0	0	0	1	0	1	0	0
3434	2021	9	M	44	0	0	0	0	0	0	0	0	0	1	1	0	0	0	0	2	0	1	0	0
3435	2021	10	F	44	0	0	0	0	0	0	0	0	0	1	1	0	0	0	0	2	1	0	1	1
3436	2021	10	M	44	0	0	0	0	0	0	0	0	0	1	0	0	0	0	0	2	1	1	1	1
3437	2021	12	F	44	0	0	0	0	0	0	0	0	1	0	0	0	0	0	0	NA	0	0	0	0
3438	2021	10	F	44	0	0	0	0	0	0	0	0	0	1	0	0	0	0	0	NA	0	0	0	0
3439	2021	4	F	44	0	0	0	0	0	0	0	0	0	1	0	0	0	0	0	NA	0	1	0	0
3440	2022	7	F	44	0	0	0	0	0	0	0	0	1	0	0	0	0	0	0	1	0	1	1	0
3441	2023	5	M	44	0	0	0	0	0	0	0	0	1	0	0	0	0	0	1	NA	0	0	0	0
3442	2024	1	M	44	0	0	0	0	0	0	0	0	1	0	0	0	0	0	1	NA	0	0	0	0
3443	2024	2	F	44	0	0	0	0	0	0	0	0	1	0	0	0	0	0	0	NA	0	0	0	0
3444	2020	7	F	44	0	0	1	0	0	0	0	0	0	0	1	1	1	0	0	NA	0	0	0	0
3445	2021	1	F	44	0	0	1	0	0	0	0	0	0	0	1	1	1	0	0	NA	0	0	0	0
3446	2022	8	M	44	0	0	1	0	0	0	0	0	0	0	1	0	0	0	0	NA	0	0	0	0
3447	2022	9	M	44	0	0	1	0	0	0	0	0	0	0	1	1	1	0	0	1	0	0	1	1
3448	2022	9	M	44	0	0	1	0	0	0	0	0	0	0	1	1	0	0	0	1	0	0	1	1
3449	2022	10	F	44	0	0	1	0	0	0	0	0	0	0	1	1	1	0	0	1	0	1	0	0
3450	2024	9	F	44	0	0	1	0	0	0	0	0	0	0	1	1	1	0	0	2	1	1	1	1
3451	2024	8	M	44	0	0	0	0	1	0	0	0	0	0	1	1	0	0	0	NA	0	0	0	0
3452	2021	2	F	44	0	1	0	0	0	1	0	0	0	0	1	1	0	0	0	NA	0	0	0	0
3453	2020	3	M	44	0	0	1	1	0	0	0	0	0	0	1	0	1	0	0	2	0	0	1	1
3454	2020	8	F	44	1	0	1	0	0	0	0	0	0	0	1	1	0	0	1	NA	0	0	0	0
3455	2023	8	M	44	0	0	1	1	0	0	0	0	0	0	1	1	0	0	0	2	0	1	0	0
3456	2024	10	M	44	0	0	1	0	1	0	0	0	0	0	1	0	0	0	0	NA	0	0	0	0
3457	2022	4	M	44	1	1	0	0	0	0	0	0	0	0	1	1	1	0	0	2	0	0	1	1
3458	2023	8	F	44	0	0	0	0	1	0	1	0	0	0	1	1	1	0	0	2	0	0	1	1
3459	2020	6	M	44	1	1	0	0	1	0	0	0	0	0	1	1	0	0	0	NA	0	0	0	0
3460	2021	4	M	44	1	1	1	0	0	0	0	0	0	0	1	1	0	0	0	NA	0	0	0	0
3461	2021	6	M	44	0	1	1	0	1	0	0	0	0	0	1	1	0	0	0	2	0	1	0	0
3462	2024	7	F	44	0	1	1	0	1	0	0	0	0	0	1	0	0	0	1	2	0	1	0	1
3463	2020	1	M	44	1	1	1	1	0	0	0	0	0	0	1	1	0	0	0	2	1	1	1	1
3464	2023	6	M	44	0	1	1	0	1	1	0	0	0	0	1	0	1	0	0	2	0	1	0	0
3465	2022	9	M	44	1	1	1	0	1	0	1	0	0	0	1	1	1	1	0	NA	0	0	0	0

ID	Yr	Mo	Sex	Age	Tre	Grs	Rag	Mug	HDM	Dan	Mld	Food	Drg	Ven	Rhi	Cnj	Ast	AsE	Urt	Sev	Prt	Obs	Rho	Snz
3466	2020	10	M	44	1	1	1	1	1	1	1	0	0	0	1	1	1	0	0	2	0	1	1	0
3467	2020	2	F	45	0	0	0	0	0	0	0	0	0	0	1	0	1	0	0	2	2	1	0	1
3468	2020	3	M	45	0	0	0	0	0	0	0	0	0	0	0	0	1	0	0	1	0	0	0	0
3469	2020	3	F	45	0	0	0	0	0	0	0	0	0	0	0	0	0	0	1	2	1	0	0	0
3470	2020	7	F	45	0	0	0	0	0	0	0	0	0	0	0	0	1	0	0	2	1	1	0	1
3471	2020	7	F	45	0	0	0	0	0	0	0	0	0	0	1	0	0	0	0	2	0	1	0	1
3472	2020	8	F	45	0	0	0	0	0	0	0	0	0	0	1	0	0	0	0	NA	0	0	0	0
3473	2020	9	F	45	0	0	0	0	0	0	0	0	0	0	1	1	0	0	0	2	1	1	1	1
3474	2020	9	F	45	0	0	0	0	0	0	0	0	0	0	1	0	1	0	0	2	0	1	0	0
3475	2020	11	M	45	0	0	0	0	0	0	0	0	0	0	1	0	1	0	0	2	1	1	0	0
3476	2020	12	F	45	0	0	0	0	0	0	0	0	0	0	0	0	0	0	1	2	1	1	1	1
3477	2021	9	M	45	0	0	0	0	0	0	0	0	0	0	1	1	0	0	0	NA	0	0	0	0
3478	2021	9	M	45	0	0	0	0	0	0	0	0	0	0	1	1	0	0	0	NA	0	0	0	0
3479	2021	10	F	45	0	0	0	0	0	0	0	0	0	0	0	0	0	0	0	2	0	1	1	0
3480	2021	11	F	45	0	0	0	0	0	0	0	0	0	0	0	0	0	0	0	2	1	1	1	1
3481	2021	2	M	45	0	0	0	0	0	0	0	0	0	0	0	0	0	0	1	2	0	0	0	0
3482	2021	12	F	45	0	0	0	0	0	0	0	0	0	0	1	0	0	0	0	2	0	0	1	1
3483	2022	2	M	45	0	0	0	0	0	0	0	0	0	0	0	0	0	0	1	2	0	0	0	0
3484	2022	6	M	45	0	0	0	0	0	0	0	0	0	0	0	0	0	0	1	1	0	0	0	0
3485	2022	8	M	45	0	0	0	0	0	0	0	0	0	0	1	0	0	0	0	1	0	0	0	0
3486	2023	1	M	45	0	0	0	0	0	0	0	0	0	0	0	0	0	0	1	NA	0	0	0	0
3487	2023	4	M	45	0	0	0	0	0	0	0	0	0	0	0	0	0	0	1	2	1	1	1	0
3488	2023	5	F	45	0	0	0	0	0	0	0	0	0	0	0	0	1	0	0	NA	0	0	0	0
3489	2023	7	F	45	0	0	0	0	0	0	0	0	0	0	0	0	0	0	1	NA	0	0	0	0
3490	2023	9	F	45	0	0	0	0	0	0	0	0	0	0	1	0	0	0	0	NA	0	0	0	0
3491	2023	10	F	45	0	0	0	0	0	0	0	0	0	0	1	0	1	0	0	2	1	1	0	1
3492	2023	11	F	45	0	0	0	0	0	0	0	0	0	0	0	0	0	0	1	2	0	0	0	0
3493	2024	4	F	45	0	0	0	0	0	0	0	0	0	0	0	0	0	0	1	NA	0	0	0	0
3494	2024	6	F	45	0	0	0	0	0	0	0	0	0	0	0	0	0	0	0	2	0	0	1	1
3495	2024	7	F	45	0	0	0	0	0	0	0	0	0	0	0	0	1	0	0	NA	0	0	0	0
3496	2024	8	M	45	0	0	0	0	0	0	0	0	0	0	0	0	0	0	0	2	0	0	1	1
3497	2024	10	F	45	0	0	0	0	0	0	0	0	0	0	0	0	0	0	1	2	0	1	0	1
3498	2021	6	M	45	0	0	0	0	0	0	0	1	0	0	0	0	0	0	1	2	0	1	0	0
3499	2021	7	F	45	0	0	0	0	0	0	0	1	0	0	0	0	0	0	1	2	1	0	1	1
3500	2021	10	F	45	0	0	0	0	0	0	0	0	1	0	0	0	0	0	1	2	0	1	1	1
3501	2021	6	F	45	0	0	0	0	0	0	0	0	0	1	1	0	0	0	0	NA	0	0	0	0
3502	2021	5	F	45	0	0	0	0	0	0	0	0	0	1	0	1	0	0	0	2	0	1	0	0
3503	2021	5	M	45	0	0	0	0	0	0	0	0	0	1	0	0	0	0	0	NA	0	0	0	0
3504	2021	12	F	45	0	0	0	0	0	0	0	0	0	1	0	1	0	0	0	2	0	1	0	0
3505	2022	6	M	45	0	0	0	0	0	0	0	1	0	0	1	0	0	0	0	2	0	1	0	0
3506	2020	1	F	45	0	0	0	1	0	0	0	0	0	0	1	0	0	0	0	2	0	0	0	0
3507	2020	2	M	45	0	0	1	0	0	0	0	0	0	0	1	1	1	0	0	2	0	1	0	0
3508	2020	2	M	45	0	0	1	0	0	0	0	0	0	0	1	0	0	0	0	NA	0	0	0	0
3509	2023	2	M	45	0	0	1	0	0	0	0	0	0	0	1	0	0	0	0	NA	0	0	0	0
3510	2023	6	M	45	0	0	1	0	0	0	0	0	0	0	1	0	1	0	0	NA	0	0	0	0
3511	2023	8	F	45	0	0	1	0	0	0	0	0	0	0	1	1	0	0	0	NA	0	0	0	0
3512	2023	8	M	45	0	0	1	0	0	0	0	0	0	0	1	1	0	0	0	2	0	0	0	0
3513	2023	10	M	45	0	0	1	0	0	0	0	0	0	0	1	0	1	0	0	2	1	1	1	1
3514	2024	3	M	45	0	0	1	0	0	0	0	0	0	0	1	1	1	0	0	2	1	1	0	1
3515	2024	10	M	45	0	0	1	0	0	0	0	0	0	0	1	1	0	0	0	NA	0	0	0	0
3516	2022	4	M	45	1	0	0	0	0	0	0	0	0	0	1	1	1	0	0	2	1	1	1	1
3517	2022	9	M	45	1	0	0	0	0	0	0	0	0	0	1	1	0	0	0	2	0	0	0	0
3518	2023	3	F	45	0	0	0	0	1	0	0	0	0	0	1	0	1	0	0	2	0	0	0	0
3519	2021	12	F	45	0	0	0	0	1	0	0	0	0	1	0	0	0	0	0	2	1	0	1	1
3520	2021	9	F	45	0	0	1	0	0	0	0	0	0	1	1	0	0	0	0	NA	0	0	0	0
3521	2023	8	M	45	0	0	1	0	0	0	0	1	0	1	1	1	0	0	0	2	1	1	1	1
3522	2021	8	F	45	0	0	0	0	1	0	1	0	0	0	1	0	1	0	0	2	0	1	0	0
3523	2020	9	M	45	0	0	1	0	1	0	0	0	0	0	1	0	1	1	0	NA	0	0	0	0
3524	2020	11	F	45	0	0	1	0	1	0	0	0	0	0	1	1	1	0	0	2	1	1	1	1
3525	2022	9	M	45	0	0	1	0	0	1	0	0	0	0	1	1	0	0	0	NA	0	0	0	0
3526	2023	5	M	45	0	1	1	0	0	0	0	0	0	0	1	0	0	0	0	2	0	1	0	0
3527	2023	4	M	45	0	0	0	0	1	0	1	0	0	0	1	1	0	0	0	2	0	1	0	0
3528	2024	1	F	45	0	1	0	0	1	0	0	0	0	0	1	1	0	0	0	NA	0	0	0	0

ID	Yr	Mo	Sex	Age	Tre	Grs	Rag	Mug	HDM	Dan	Mld	Food	Drg	Ven	Rhi	Cnj	Ast	AsE	Urt	Sev	Prt	Obs	Rho	Snz
3529	2020	3	M	45	0	1	1	0	1	0	0	0	0	0	1	0	1	0	0	2	1	0	1	1
3530	2023	10	F	45	0	0	1	0	1	0	1	0	0	0	1	0	1	1	0	NA	0	0	0	0
3531	2024	1	M	45	0	1	1	0	0	1	0	0	0	0	1	1	0	0	0	NA	0	0	0	0
3532	2022	1	F	45	0	0	1	1	1	0	0	1	0	0	1	1	1	0	0	2	0	1	1	1
3533	2021	9	M	45	1	1	1	0	0	0	0	0	0	1	1	0	0	0	0	NA	0	0	0	0
3534	2020	6	F	45	1	1	1	0	0	1	0	0	0	0	1	1	1	0	0	NA	0	0	0	0
3535	2020	2	F	46	0	0	0	0	0	0	0	0	0	0	1	0	1	0	0	2	1	1	1	1
3536	2020	2	M	46	0	0	0	0	0	0	0	0	0	0	1	0	0	0	0	2	0	1	0	0
3537	2020	10	F	46	0	0	0	0	0	0	0	0	0	0	0	0	0	0	1	NA	0	0	0	0
3538	2020	7	F	46	0	0	0	0	0	0	0	0	0	0	0	1	0	0	0	2	0	1	0	0
3539	2020	10	M	46	0	0	0	0	0	0	0	0	0	0	0	0	0	0	1	NA	0	0	0	0
3540	2020	12	M	46	0	0	0	0	0	0	0	0	0	0	0	0	0	0	0	NA	0	0	0	0
3541	2021	3	F	46	0	0	0	0	0	0	0	0	0	0	0	0	0	0	0	NA	0	0	0	0
3542	2021	9	M	46	0	0	0	0	0	0	0	0	0	0	1	1	0	0	0	2	0	1	0	0
3543	2021	11	F	46	0	0	0	0	0	0	0	0	0	0	1	0	0	0	0	2	1	1	0	1
3544	2021	9	M	46	0	0	0	0	0	0	0	0	0	0	0	0	0	1	0	2	0	1	0	1
3545	2021	3	F	46	0	0	0	0	0	0	0	0	0	0	0	0	0	1	1	2	0	1	0	0
3546	2021	10	M	46	0	0	0	0	0	0	0	0	0	0	0	0	0	1	0	NA	0	0	0	0
3547	2022	1	M	46	0	0	0	0	0	0	0	0	0	0	0	0	0	0	0	2	0	1	1	0
3548	2022	2	F	46	0	0	0	0	0	0	0	0	0	0	1	0	1	0	0	NA	0	0	0	0
3549	2022	3	F	46	0	0	0	0	0	0	0	0	0	0	0	0	0	0	0	2	0	1	0	0
3550	2022	3	M	46	0	0	0	0	0	0	0	0	0	0	0	0	0	0	1	NA	0	0	0	0
3551	2022	4	F	46	0	0	0	0	0	0	0	0	0	0	0	0	0	0	1	NA	0	0	0	0
3552	2022	11	M	46	0	0	0	0	0	0	0	0	0	0	0	0	1	0	0	NA	0	0	0	0
3553	2022	11	M	46	0	0	0	0	0	0	0	0	0	0	0	0	1	0	0	2	1	0	0	1
3554	2022	11	F	46	0	0	0	0	0	0	0	0	0	0	0	0	0	0	1	NA	0	0	0	0
3555	2023	3	F	46	0	0	0	0	0	0	0	0	0	0	0	0	1	0	0	NA	0	0	0	0
3556	2023	4	M	46	0	0	0	0	0	0	0	0	0	0	0	0	0	0	0	2	0	1	1	1
3557	2023	6	F	46	0	0	0	0	0	0	0	0	0	0	0	0	0	0	1	2	1	0	1	1
3558	2023	11	F	46	0	0	0	0	0	0	0	0	0	0	0	0	0	0	1	2	0	1	0	1
3559	2024	3	F	46	0	0	0	0	0	0	0	0	0	0	0	0	0	0	1	NA	0	0	0	0
3560	2024	5	M	46	0	0	0	0	0	0	0	0	0	0	0	0	0	0	0	1	0	1	1	0
3561	2024	5	F	46	0	0	0	0	0	0	0	0	0	0	0	0	0	0	1	NA	0	0	0	0
3562	2024	6	F	46	0	0	0	0	0	0	0	0	0	0	0	0	0	0	0	NA	0	0	0	0
3563	2024	7	F	46	0	0	0	0	0	0	0	0	0	0	1	0	0	0	0	NA	0	0	0	0
3564	2024	7	F	46	0	0	0	0	0	0	0	0	0	0	0	0	0	0	0	2	0	1	0	1
3565	2024	8	F	46	0	0	0	0	0	0	0	0	0	0	1	0	0	0	0	NA	0	0	0	0
3566	2024	9	M	46	0	0	0	0	0	0	0	0	0	0	0	0	0	0	1	2	0	0	1	1
3567	2024	11	M	46	0	0	0	0	0	0	0	0	0	0	1	0	0	0	0	NA	0	0	0	0
3568	2021	11	F	46	0	0	0	0	0	0	0	0	0	1	0	0	0	0	0	NA	0	0	0	0
3569	2021	11	F	46	0	0	0	0	0	0	0	0	0	1	0	1	0	0	0	2	0	0	0	0
3570	2022	9	M	46	0	0	0	0	0	0	0	0	0	1	0	0	0	0	0	2	0	1	0	0
3571	2023	2	F	46	0	0	0	0	0	0	0	0	1	0	1	0	1	0	0	2	0	0	1	1
3572	2023	11	F	46	0	0	0	0	0	0	0	0	0	1	0	0	0	0	0	2	0	1	0	1
3573	2024	10	F	46	0	0	0	0	0	0	0	0	1	0	0	0	0	0	1	2	0	0	1	0
3574	2020	11	F	46	0	0	0	0	1	0	0	0	0	0	1	1	1	0	0	2	0	1	1	1
3575	2020	2	F	46	0	0	1	0	0	0	0	0	0	0	1	0	0	0	1	2	1	1	0	1
3576	2020	12	F	46	0	0	1	0	0	0	0	0	0	0	1	1	0	0	0	NA	0	0	0	0
3577	2022	5	F	46	0	0	1	0	0	0	0	0	0	0	1	0	1	0	1	NA	0	0	0	0
3578	2022	8	M	46	0	0	1	0	0	0	0	0	0	0	1	1	1	0	0	NA	0	0	0	0
3579	2022	9	F	46	0	0	1	0	0	0	0	0	0	0	1	1	0	0	0	NA	0	0	0	0
3580	2023	4	F	46	0	0	1	0	0	0	0	0	0	0	1	0	0	0	0	NA	0	0	0	0
3581	2023	8	M	46	0	0	1	0	0	0	0	0	0	0	1	0	1	1	0	NA	0	0	0	0
3582	2023	9	F	46	0	0	1	0	0	0	0	0	0	0	1	1	1	0	0	NA	0	0	0	0
3583	2024	5	M	46	0	0	1	0	0	0	0	0	0	0	1	1	1	0	0	NA	0	0	0	0
3584	2024	7	F	46	0	0	1	0	0	0	0	0	0	0	1	1	0	0	0	NA	0	0	0	0
3585	2024	10	F	46	0	0	1	0	0	0	0	0	0	0	1	1	0	0	0	2	0	1	1	1
3586	2024	11	M	46	0	0	1	0	0	0	0	0	0	0	1	1	0	0	0	2	0	1	0	1
3587	2024	11	M	46	0	0	1	0	0	0	0	0	0	0	1	0	1	0	0	2	1	1	0	1
3588	2024	12	F	46	0	0	1	0	0	0	0	0	0	0	1	1	0	0	0	2	0	1	1	1
3589	2023	4	F	46	0	0	0	0	1	0	0	0	0	0	1	0	1	0	0	NA	0	0	0	0
3590	2023	4	F	46	0	0	0	0	1	0	0	0	0	0	1	0	0	0	0	2	1	1	0	1
3591	2023	5	M	46	1	0	0	0	0	0	0	0	0	0	1	0	0	0	0	NA	0	0	0	0

ID	Yr	Mo	Sex	Age	Tre	Grs	Rag	Mug	HDM	Dan	Mld	Food	Drg	Ven	Rhi	Cnj	Ast	AsE	Urt	Sev	Prt	Obs	Rho	Snz
3592	2023	10	M	46	0	0	0	0	1	0	0	0	0	0	1	0	0	0	0	NA	0	0	0	0
3593	2024	4	F	46	0	1	0	0	0	0	0	0	0	0	1	0	0	0	0	NA	0	0	0	0
3594	2021	8	F	46	0	0	1	0	0	0	0	0	0	1	1	1	0	0	0	NA	0	0	0	0
3595	2022	11	F	46	0	1	1	0	0	0	0	0	0	0	0	0	0	0	0	NA	0	0	0	0
3596	2023	4	M	46	0	0	1	0	1	0	0	0	0	0	1	0	1	0	0	2	1	1	1	1
3597	2024	3	M	46	0	0	1	0	0	1	0	0	0	0	1	0	1	0	0	2	1	1	0	1
3598	2022	8	M	46	0	1	0	0	0	0	1	0	0	0	1	0	1	0	0	2	0	1	1	0
3599	2024	3	F	46	0	0	0	0	1	1	0	0	0	0	1	0	0	0	0	2	1	0	0	1
3600	2021	12	F	46	0	0	0	0	1	1	0	0	0	1	0	1	0	0	0	NA	0	0	0	0
3601	2020	10	F	46	0	1	1	0	0	1	0	0	0	0	1	1	0	0	0	2	1	0	1	1
3602	2023	1	M	46	0	1	1	0	1	0	0	0	0	0	1	0	0	0	0	2	0	0	0	0
3603	2023	12	F	46	0	1	1	0	1	0	0	0	0	0	0	0	0	0	1	2	0	0	0	0
3604	2024	5	M	46	1	1	0	0	0	0	1	1	0	0	1	1	1	0	0	2	0	1	0	1
3605	2021	12	M	46	1	1	0	0	1	1	0	0	0	0	0	0	1	0	0	NA	0	0	0	0
3606	2020	1	M	47	0	0	0	0	0	0	0	0	0	0	0	0	0	0	0	NA	0	0	0	0
3607	2020	10	F	47	0	0	0	0	0	0	0	0	0	0	0	0	0	0	1	2	0	0	0	0
3608	2021	5	F	47	0	0	0	0	0	0	0	0	0	0	1	0	0	0	0	NA	0	0	0	0
3609	2021	10	M	47	0	0	0	0	0	0	0	0	0	0	0	0	0	0	1	NA	0	0	0	0
3610	2021	11	F	47	0	0	0	0	0	0	0	0	0	0	1	1	0	0	1	NA	0	0	0	0
3611	2021	9	F	47	0	0	0	0	0	0	0	0	0	0	0	0	0	1	0	2	0	0	0	1
3612	2022	3	F	47	0	0	0	0	0	0	0	0	0	0	0	0	0	0	0	2	0	0	0	0
3613	2022	5	F	47	0	0	0	0	0	0	0	0	0	0	1	0	0	0	0	NA	0	0	0	0
3614	2022	6	F	47	0	0	0	0	0	0	0	0	0	0	1	0	0	0	0	2	1	1	1	1
3615	2022	9	F	47	0	0	0	0	0	0	0	0	0	0	0	0	0	0	1	2	0	1	1	0
3616	2022	9	F	47	0	0	0	0	0	0	0	0	0	0	0	0	0	0	0	NA	0	0	0	0
3617	2022	9	M	47	0	0	0	0	0	0	0	0	0	0	0	0	1	0	0	NA	0	0	0	0
3618	2022	10	F	47	0	0	0	0	0	0	0	0	0	0	0	0	0	0	0	NA	0	0	0	0
3619	2023	3	M	47	0	0	0	0	0	0	0	0	0	0	1	0	0	0	1	NA	0	0	0	0
3620	2023	4	F	47	0	0	0	0	0	0	0	0	0	0	1	0	0	0	0	NA	0	0	0	0
3621	2023	5	F	47	0	0	0	0	0	0	0	0	0	0	0	0	0	0	1	NA	0	0	0	0
3622	2023	10	M	47	0	0	0	0	0	0	0	0	0	0	0	0	1	0	0	NA	0	0	0	0
3623	2023	11	M	47	0	0	0	0	0	0	0	0	0	0	0	0	0	0	1	2	1	1	1	1
3624	2024	2	F	47	0	0	0	0	0	0	0	0	0	0	1	1	0	0	0	2	0	1	0	0
3625	2024	3	F	47	0	0	0	0	0	0	0	0	0	0	0	0	0	0	0	2	1	1	0	1
3626	2024	3	F	47	0	0	0	0	0	0	0	0	0	0	1	0	1	0	0	NA	0	0	0	0
3627	2024	3	M	47	0	0	0	0	0	0	0	0	0	0	0	0	0	0	0	2	0	1	0	1
3628	2024	3	F	47	0	0	0	0	0	0	0	0	0	0	0	0	0	0	1	2	0	0	0	0
3629	2024	4	F	47	0	0	0	0	0	0	0	0	0	0	0	0	0	0	0	2	1	1	1	1
3630	2024	4	F	47	0	0	0	0	0	0	0	0	0	0	0	0	0	0	0	2	0	1	0	1
3631	2024	6	M	47	0	0	0	0	0	0	0	0	0	0	1	0	0	0	1	NA	0	0	0	0
3632	2024	7	F	47	0	0	0	0	0	0	0	0	0	0	1	0	1	0	0	2	0	0	0	0
3633	2024	7	M	47	0	0	0	0	0	0	0	0	0	0	0	0	0	0	1	2	0	1	1	1
3634	2024	7	F	47	0	0	0	0	0	0	0	0	0	0	0	0	0	0	1	NA	0	0	0	0
3635	2024	8	M	47	0	0	0	0	0	0	0	0	0	0	0	0	0	0	1	2	1	0	1	1
3636	2024	11	F	47	0	0	0	0	0	0	0	0	0	0	0	0	0	0	1	NA	0	0	0	0
3637	2020	9	M	47	0	0	0	0	0	0	0	0	0	1	0	0	0	0	0	NA	0	0	0	0
3638	2021	10	F	47	0	0	0	0	0	0	0	0	0	1	1	0	0	1	0	NA	0	0	0	0
3639	2023	11	F	47	0	0	0	0	0	0	0	1	0	0	0	0	0	0	1	2	1	1	1	0
3640	2024	7	F	47	0	0	0	0	0	0	0	0	1	0	0	0	0	0	0	2	1	1	0	1
3641	2024	7	M	47	0	0	0	0	0	0	0	0	1	1	0	0	0	0	0	2	0	0	1	0
3642	2020	7	F	47	0	1	0	0	0	0	0	0	0	0	1	1	0	0	0	2	1	1	0	1
3643	2023	2	M	47	0	0	1	0	0	0	0	0	0	0	1	0	1	0	0	2	1	1	1	0
3644	2023	8	M	47	0	0	1	0	0	0	0	0	0	0	1	1	1	0	0	NA	0	0	0	0
3645	2024	9	M	47	0	0	1	0	0	0	0	0	0	0	1	1	0	0	0	2	0	1	0	1
3646	2023	11	F	47	1	0	0	0	0	0	0	0	0	0	1	1	1	0	0	2	1	1	1	1
3647	2024	10	F	47	0	0	0	0	1	0	0	0	0	0	1	1	1	0	0	NA	0	0	0	0
3648	2021	9	F	47	0	0	1	0	0	0	0	0	0	1	1	0	0	0	0	2	1	1	0	1
3649	2023	3	M	47	0	0	1	0	0	0	0	0	0	1	1	0	1	0	0	2	0	1	0	1
3650	2021	10	F	47	0	1	0	0	0	0	1	0	0	0	1	0	0	0	0	2	0	0	0	0
3651	2020	7	M	47	0	0	1	1	0	0	0	0	0	0	1	1	0	0	1	2	0	0	1	1
3652	2021	6	M	47	0	1	1	0	0	0	0	0	0	0	1	0	0	0	0	NA	0	0	0	0
3653	2022	1	F	47	0	0	1	0	0	1	0	0	0	0	1	0	1	0	0	NA	0	0	0	0
3654	2022	5	F	47	0	0	1	0	0	1	0	0	0	0	1	1	1	0	0	2	0	1	1	0

ID	Yr	Mo	Sex	Age	Tre	Grs	Rag	Mug	HDM	Dan	Mld	Food	Drg	Ven	Rhi	Cnj	Ast	AsE	Urt	Sev	Prt	Obs	Rho	Snz
3655	2022	5	M	47	0	1	1	0	0	0	0	0	0	0	1	1	0	0	0	2	0	1	0	1
3656	2024	9	F	47	1	0	1	0	0	0	0	0	0	0	1	1	0	0	0	NA	0	0	0	0
3657	2024	11	F	47	0	1	1	0	0	0	0	0	0	0	1	1	0	0	1	2	1	1	1	1
3658	2023	3	F	47	1	1	0	0	0	0	0	0	0	0	1	0	0	0	0	2	0	1	0	0
3659	2024	4	F	47	1	0	0	0	1	0	0	0	0	0	1	1	0	0	0	2	0	1	1	0
3660	2021	9	M	47	0	0	1	1	0	0	0	0	0	1	1	0	0	0	0	2	0	1	0	0
3661	2021	11	F	47	0	0	1	0	0	1	0	0	0	1	0	1	0	0	0	2	1	1	0	1
3662	2021	2	F	47	1	1	0	0	1	0	0	0	0	0	1	1	0	0	0	NA	0	1	0	1
3663	2023	7	F	47	0	1	1	1	0	0	0	0	0	0	1	0	0	0	0	2	0	1	1	1
3664	2024	5	F	47	0	0	1	0	0	1	1	0	0	0	1	1	1	0	1	2	1	1	0	0
3665	2022	2	F	47	0	1	0	0	1	1	0	0	0	0	0	0	1	0	1	2	1	1	1	1
3666	2020	3	M	47	1	1	1	1	0	0	0	0	0	0	1	0	1	0	1	2	0	1	1	0
3667	2022	1	M	47	1	1	1	1	0	0	0	0	0	0	1	1	0	0	0	NA	0	0	0	0
3668	2020	9	F	48	0	0	0	0	0	0	0	0	0	0	1	0	1	0	0	NA	0	0	0	0
3669	2020	9	M	48	0	0	0	0	0	0	0	0	0	0	1	1	0	0	0	NA	0	0	0	0
3670	2020	10	M	48	0	0	0	0	0	0	0	0	0	0	0	0	0	0	0	2	0	0	1	1
3671	2020	10	F	48	0	0	0	0	0	0	0	0	0	0	0	0	1	0	0	2	0	1	1	1
3672	2020	12	F	48	0	0	0	0	0	0	0	0	0	0	1	0	1	0	0	2	0	1	0	0
3673	2021	4	F	48	0	0	0	0	0	0	0	0	0	0	0	0	0	0	1	NA	0	0	0	0
3674	2021	10	M	48	0	0	0	0	0	0	0	0	0	0	0	0	0	0	0	2	0	1	0	0
3675	2021	12	F	48	0	0	0	0	0	0	0	0	0	0	1	0	0	0	0	2	1	0	0	1
3676	2023	11	F	48	0	0	0	0	0	0	0	0	0	0	0	0	0	0	1	NA	0	0	0	0
3677	2021	5	M	48	0	0	0	0	0	0	0	0	0	0	0	0	0	1	0	2	0	1	0	1
3678	2022	1	F	48	0	0	0	0	0	0	0	0	0	0	0	0	1	0	0	2	0	1	0	0
3679	2022	1	M	48	0	0	0	0	0	0	0	0	0	0	0	0	0	0	1	NA	0	0	0	0
3680	2022	4	F	48	0	0	0	0	0	0	0	0	0	0	0	0	1	0	0	2	1	0	1	1
3681	2022	10	F	48	0	0	0	0	0	0	0	0	0	0	0	0	0	0	0	2	1	1	0	1
3682	2022	10	F	48	0	0	0	0	0	0	0	0	0	0	0	0	0	0	0	NA	0	0	0	0
3683	2023	6	F	48	0	0	0	0	0	0	0	0	0	0	0	0	0	0	1	2	1	1	0	1
3684	2023	8	F	48	0	0	0	0	0	0	0	0	0	0	1	0	0	0	0	2	0	0	0	0
3685	2023	9	F	48	0	0	0	0	0	0	0	0	0	0	0	0	0	0	1	NA	0	0	0	0
3686	2024	3	F	48	0	0	0	0	0	0	0	0	0	0	0	0	0	0	1	2	0	0	0	0
3687	2024	7	F	48	0	0	0	0	0	0	0	0	0	0	0	0	0	0	1	2	0	1	0	1
3688	2024	7	M	48	0	0	0	0	0	0	0	0	0	0	0	0	0	0	0	NA	0	1	1	0
3689	2024	9	F	48	0	0	0	0	0	0	0	0	0	0	0	0	0	0	1	2	0	1	0	0
3690	2021	9	M	48	0	0	0	0	0	0	0	0	0	1	0	0	0	0	0	2	0	0	1	1
3691	2022	12	M	48	0	0	0	0	0	0	0	0	0	1	0	0	0	0	1	2	0	1	0	0
3692	2020	3	F	48	0	1	0	0	0	0	0	0	0	0	1	0	1	0	0	2	0	0	1	1
3693	2020	3	F	48	0	0	1	0	0	0	0	0	0	0	0	1	1	0	0	NA	0	0	0	0
3694	2020	10	F	48	0	0	1	0	0	0	0	0	0	0	1	1	1	0	0	2	1	1	0	1
3695	2021	6	F	48	0	0	1	0	0	0	0	0	0	0	1	1	0	0	0	NA	0	0	0	0
3696	2022	3	F	48	0	0	1	0	0	0	0	0	0	0	1	0	0	0	0	NA	0	0	0	0
3697	2022	12	F	48	0	0	1	0	0	0	0	0	0	0	1	0	0	0	0	2	0	0	1	0
3698	2023	6	F	48	0	0	1	0	0	0	0	0	0	0	1	0	0	0	1	NA	0	0	0	0
3699	2023	9	M	48	0	0	0	0	0	0	1	0	0	0	1	0	1	0	0	1	0	1	0	0
3700	2021	4	F	48	0	0	0	0	0	0	1	0	0	1	1	1	0	0	0	2	0	1	0	0
3701	2021	1	M	48	0	0	0	1	0	0	1	0	0	0	1	0	1	0	0	NA	0	0	0	0
3702	2020	10	M	48	1	0	1	0	0	0	0	0	0	0	1	1	1	0	0	2	0	1	0	0
3703	2024	9	F	48	0	0	1	0	1	0	0	0	0	0	1	1	1	0	0	2	0	1	0	0
3704	2022	12	M	48	0	0	1	1	0	1	0	0	0	0	1	0	0	0	0	2	0	0	0	0
3705	2023	2	F	48	1	1	1	0	0	0	0	0	0	0	1	0	1	0	1	2	0	0	0	0
3706	2023	6	F	48	0	0	1	0	1	0	1	0	0	0	1	0	1	0	0	NA	0	0	0	0
3707	2023	11	M	48	1	1	1	0	0	0	0	0	0	0	1	1	0	0	0	1	0	1	0	1
3708	2022	10	F	48	1	1	0	0	1	0	0	0	0	0	1	0	0	0	1	2	0	1	0	0
3709	2020	9	F	48	0	1	1	0	1	0	1	0	0	0	1	1	0	0	0	2	0	1	1	1
3710	2023	4	F	48	1	1	1	0	1	0	0	0	0	0	1	0	1	0	0	2	0	1	1	0
3711	2021	5	M	48	1	1	1	0	0	1	0	0	0	1	1	0	0	0	0	NA	0	0	0	0
3712	2022	10	F	48	0	1	1	1	0	1	1	0	0	0	1	1	0	0	0	2	0	1	0	1
3713	2022	5	F	48	1	1	1	0	1	1	1	0	0	0	1	1	0	0	0	NA	0	0	0	0
3714	2020	2	F	49	0	0	0	0	0	0	0	0	0	0	0	0	0	0	0	2	1	0	1	0
3715	2020	7	M	49	0	0	0	0	0	0	0	0	0	0	0	0	0	0	0	NA	0	0	0	0
3716	2020	7	F	49	0	0	0	0	0	0	0	0	0	0	1	0	1	0	0	2	0	1	0	0
3717	2020	9	F	49	0	0	0	0	0	0	0	0	0	0	1	0	1	0	0	NA	0	0	0	0

ID	Yr	Mo	Sex	Age	Tre	Grs	Rag	Mug	HDM	Dan	Mld	Food	Drg	Ven	Rhi	Cnj	Ast	AsE	Urt	Sev	Prt	Obs	Rho	Snz
3718	2020	11	M	49	0	0	0	0	0	0	0	0	0	0	1	0	0	0	0	2	0	0	1	1
3719	2021	1	M	49	0	0	0	0	0	0	0	0	0	0	0	0	0	0	0	NA	0	0	0	0
3720	2021	9	F	49	0	0	0	0	0	0	0	0	0	0	0	0	0	0	1	2	1	1	1	0
3721	2021	3	F	49	0	0	0	0	0	0	0	0	0	0	1	0	0	0	0	NA	0	0	0	0
3722	2022	2	F	49	0	0	0	0	0	0	0	0	0	0	0	0	0	0	0	2	0	1	0	0
3723	2022	4	M	49	0	0	0	0	0	0	0	0	0	0	0	0	0	0	0	2	1	1	1	1
3724	2022	11	F	49	0	0	0	0	0	0	0	0	0	0	0	0	0	0	1	2	0	1	0	0
3725	2023	4	F	49	0	0	0	0	0	0	0	0	0	0	1	0	0	0	0	2	0	1	1	1
3726	2023	8	F	49	0	0	0	0	0	0	0	0	0	0	1	0	0	0	0	2	0	1	0	1
3727	2023	10	F	49	0	0	0	0	0	0	0	0	0	0	1	0	0	0	0	2	0	1	1	0
3728	2023	11	F	49	0	0	0	0	0	0	0	0	0	0	0	0	0	0	1	NA	0	0	0	0
3729	2024	3	F	49	0	0	0	0	0	0	0	0	0	0	0	0	0	0	0	2	0	1	0	1
3730	2024	3	F	49	0	0	0	0	0	0	0	0	0	0	0	0	1	0	0	NA	0	0	0	0
3731	2024	5	F	49	0	0	0	0	0	0	0	0	0	0	0	0	0	0	0	NA	0	0	0	0
3732	2024	5	F	49	0	0	0	0	0	0	0	0	0	0	1	0	0	0	0	NA	0	0	0	0
3733	2024	7	F	49	0	0	0	0	0	0	0	0	0	0	0	0	0	0	1	NA	0	0	0	0
3734	2024	7	F	49	0	0	0	0	0	0	0	0	0	0	0	0	0	0	1	2	0	1	0	0
3735	2024	8	F	49	0	0	0	0	0	0	0	0	0	0	1	0	0	0	0	2	1	1	1	1
3736	2024	10	F	49	0	0	0	0	0	0	0	0	0	0	0	0	0	0	0	2	0	0	0	0
3737	2021	8	M	49	0	0	0	0	0	0	0	0	0	1	1	0	0	1	0	2	0	1	1	1
3738	2022	1	F	49	0	0	0	0	0	0	0	0	1	0	0	0	1	0	0	2	1	1	1	1
3739	2023	5	F	49	0	0	0	0	0	0	0	0	1	0	0	0	0	0	1	NA	0	0	0	0
3740	2023	5	F	49	0	0	0	0	0	0	0	1	0	0	1	0	0	0	0	2	0	1	1	0
3741	2023	10	M	49	0	0	0	0	0	0	0	0	1	0	0	0	0	0	1	2	1	1	1	1
3742	2024	6	M	49	0	0	0	0	0	0	0	0	1	0	0	0	0	0	0	NA	0	0	0	0
3743	2021	1	F	49	0	0	0	0	1	0	0	0	0	0	1	1	1	0	0	NA	0	0	0	0
3744	2021	7	M	49	0	0	1	0	0	0	0	0	0	0	1	1	0	0	0	2	1	0	1	0
3745	2023	8	M	49	0	0	1	0	0	0	0	0	0	0	1	1	1	0	0	NA	0	0	0	0
3746	2024	9	M	49	0	0	1	0	0	0	0	0	0	0	1	1	1	0	0	2	0	1	1	1
3747	2023	11	F	49	0	0	0	0	1	0	0	0	0	0	0	1	1	0	0	NA	0	0	0	0
3748	2020	9	M	49	0	0	0	0	1	0	0	0	1	0	0	1	0	0	0	NA	0	0	0	0
3749	2023	10	F	49	0	0	1	0	0	0	0	1	0	0	1	0	1	0	1	NA	0	0	0	0
3750	2024	8	F	49	0	0	1	0	0	0	0	0	0	1	1	1	0	0	0	NA	0	0	0	0
3751	2021	5	M	49	0	0	1	0	0	0	0	0	0	1	1	0	0	0	0	NA	0	0	0	0
3752	2021	5	F	49	0	0	1	0	0	0	0	0	0	1	1	0	0	0	0	NA	0	0	0	0
3753	2021	10	M	49	0	1	1	0	0	0	0	0	0	0	1	1	0	0	1	NA	0	0	0	0
3754	2022	5	F	49	0	1	0	0	1	0	0	0	0	0	1	1	0	0	0	NA	0	0	0	0
3755	2024	6	M	49	0	1	1	0	0	0	0	1	0	0	1	1	0	0	1	NA	0	0	0	0
3756	2024	10	F	49	0	1	1	0	0	1	0	0	0	0	1	0	1	0	0	NA	0	0	0	0
3757	2020	10	F	50	0	0	0	0	0	0	0	0	0	0	0	0	1	0	0	NA	0	0	0	0
3758	2020	12	F	50	0	0	0	0	0	0	0	0	0	0	1	0	0	0	0	NA	0	0	0	0
3759	2021	3	M	50	0	0	0	0	0	0	0	0	0	0	1	0	1	0	0	NA	0	0	0	0
3760	2021	5	F	50	0	0	0	0	0	0	0	0	0	0	1	1	0	0	0	NA	0	0	0	0
3761	2021	7	F	50	0	0	0	0	0	0	0	0	0	0	1	0	1	0	0	NA	0	0	0	0
3762	2021	8	F	50	0	0	0	0	0	0	0	0	0	0	1	0	0	0	0	NA	0	0	0	0
3763	2021	10	M	50	0	0	0	0	0	0	0	0	0	0	1	1	1	0	0	NA	0	0	0	0
3764	2021	11	F	50	0	0	0	0	0	0	0	0	0	0	1	1	0	0	0	NA	0	0	0	0
3765	2021	12	M	50	0	0	0	0	0	0	0	0	0	0	0	0	1	0	0	NA	0	0	0	0
3766	2021	3	F	50	0	0	0	0	0	0	0	0	0	0	1	0	0	0	0	NA	0	0	0	0
3767	2021	1	F	50	0	0	0	0	0	0	0	0	0	0	0	0	0	0	1	NA	0	0	0	0
3768	2022	4	M	50	0	0	0	0	0	0	0	0	0	0	0	0	0	0	1	NA	0	0	0	0
3769	2022	5	F	50	0	0	0	0	0	0	0	0	0	0	0	0	0	0	1	NA	0	0	0	0
3770	2023	5	F	50	0	0	0	0	0	0	0	0	0	0	0	0	0	0	1	NA	0	0	0	0
3771	2024	4	F	50	0	0	0	0	0	0	0	0	0	0	0	0	0	0	1	NA	0	0	0	0
3772	2024	4	F	50	0	0	0	0	0	0	0	0	0	0	0	0	1	1	0	NA	0	0	0	0
3773	2024	4	F	50	0	0	0	0	0	0	0	0	0	0	0	0	0	0	1	NA	0	0	0	0
3774	2024	5	F	50	0	0	0	0	0	0	0	0	0	0	0	0	0	0	0	NA	0	0	0	0
3775	2024	5	M	50	0	0	0	0	0	0	0	0	0	0	0	0	0	0	1	NA	0	0	0	0
3776	2024	5	F	50	0	0	0	0	0	0	0	0	0	0	0	0	0	0	0	NA	0	0	0	0
3777	2024	6	F	50	0	0	0	0	0	0	0	0	0	0	0	0	0	0	0	NA	0	0	0	0
3778	2024	6	F	50	0	0	0	0	0	0	0	0	0	0	0	0	0	0	1	NA	0	0	0	0
3779	2024	10	M	50	0	0	0	0	0	0	0	0	0	0	0	0	0	0	0	NA	0	0	0	0
3780	2023	3	F	50	0	0	0	0	0	0	0	1	0	0	0	0	0	0	1	NA	0	0	0	0

ID	Yr	Mo	Sex	Age	Tre	Grs	Rag	Mug	HDM	Dan	Mld	Food	Drg	Ven	Rhi	Cnj	Ast	AsE	Urt	Sev	Prt	Obs	Rho	Snz
3781	2023	4	F	50	0	0	0	0	0	0	0	0	1	0	0	0	0	0	0	NA	0	0	0	0
3782	2024	9	F	50	0	0	0	0	0	0	0	0	1	0	0	0	0	0	0	NA	0	0	0	0
3783	2024	11	F	50	0	0	0	0	0	0	0	0	1	0	0	0	0	0	1	NA	0	0	0	0
3784	2023	5	M	50	0	0	0	0	0	0	0	1	1	0	0	0	0	0	0	NA	0	0	0	0
3785	2020	7	M	50	0	0	0	0	1	0	0	0	0	0	1	1	1	0	0	NA	0	0	0	0
3786	2022	5	M	50	0	0	1	0	0	0	0	0	0	0	0	0	0	0	1	NA	0	0	0	0
3787	2023	9	M	50	0	0	1	0	0	0	0	0	0	0	1	0	1	0	0	NA	0	0	0	0
3788	2024	11	M	50	0	1	0	0	0	0	0	0	0	0	1	0	1	0	0	NA	0	0	0	0
3789	2024	9	M	50	0	0	0	1	1	0	0	0	0	0	1	1	1	0	0	NA	0	0	0	0
3790	2021	10	F	50	0	1	1	0	0	0	0	0	0	1	1	0	0	0	0	NA	0	0	0	0
3791	2024	2	F	50	0	1	1	1	0	0	0	0	0	0	1	1	1	0	0	NA	0	0	0	0
3792	2024	2	M	50	1	0	0	0	1	1	0	0	0	0	1	0	1	0	0	2	0	1	0	1
3793	2023	8	F	50	1	1	1	0	0	0	0	0	1	0	1	1	0	0	0	2	0	0	1	1
3794	2020	1	M	51	0	0	0	0	0	0	0	0	0	0	0	0	0	0	1	NA	0	0	0	0
3795	2020	2	F	51	0	0	0	0	0	0	0	0	0	0	1	0	0	0	0	2	1	1	1	1
3796	2020	3	M	51	0	0	0	0	0	0	0	0	0	0	1	0	0	0	1	2	0	1	1	1
3797	2020	9	F	51	0	0	0	0	0	0	0	0	0	0	0	0	0	0	0	2	0	1	1	0
3798	2020	12	M	51	0	0	0	0	0	0	0	0	0	0	0	0	1	0	0	2	0	1	1	0
3799	2021	3	M	51	0	0	0	0	0	0	0	0	0	0	1	0	1	0	1	2	0	1	1	0
3800	2021	7	F	51	0	0	0	0	0	0	0	0	0	0	0	0	0	0	0	NA	0	0	0	0
3801	2021	8	F	51	0	0	0	0	0	0	0	0	0	0	0	0	0	0	1	2	0	1	1	0
3802	2021	2	F	51	0	0	0	0	0	0	0	0	0	0	0	0	1	1	1	2	0	1	1	1
3803	2021	8	M	51	0	0	0	0	0	0	0	0	0	0	0	0	0	1	0	NA	0	0	0	0
3804	2022	2	F	51	0	0	0	0	0	0	0	0	0	0	0	0	0	0	1	2	0	1	1	0
3805	2022	2	F	51	0	0	0	0	0	0	0	0	0	0	1	0	0	0	1	NA	0	0	0	0
3806	2022	3	M	51	0	0	0	0	0	0	0	0	0	0	0	0	0	0	0	2	0	1	1	0
3807	2022	4	F	51	0	0	0	0	0	0	0	0	0	0	0	0	0	0	0	2	0	1	1	1
3808	2022	5	F	51	0	0	0	0	0	0	0	0	0	0	0	0	0	0	1	1	0	1	1	1
3809	2022	7	M	51	0	0	0	0	0	0	0	0	0	0	0	0	0	0	0	NA	0	0	0	0
3810	2022	7	M	51	0	0	0	0	0	0	0	0	0	0	0	0	0	0	1	1	1	1	1	1
3811	2022	7	F	51	0	0	0	0	0	0	0	0	0	0	1	0	0	0	0	2	0	1	1	0
3812	2022	8	F	51	0	0	0	0	0	0	0	0	0	0	0	0	0	0	0	2	1	1	1	1
3813	2022	10	F	51	0	0	0	0	0	0	0	0	0	0	0	0	0	0	1	1	0	1	1	0
3814	2022	10	F	51	0	0	0	0	0	0	0	0	0	0	1	0	1	0	0	1	0	1	1	0
3815	2022	12	F	51	0	0	0	0	0	0	0	0	0	0	0	0	0	0	0	NA	0	0	0	0
3816	2023	4	F	51	0	0	0	0	0	0	0	0	0	0	1	0	0	0	0	1	0	1	1	0
3817	2023	4	F	51	0	0	0	0	0	0	0	0	0	0	1	0	0	0	0	2	0	1	0	0
3818	2023	5	F	51	0	0	0	0	0	0	0	0	0	0	0	0	0	0	1	NA	0	0	0	0
3819	2024	3	F	51	0	0	0	0	0	0	0	0	0	0	0	0	1	0	0	1	0	1	0	0
3820	2024	9	F	51	0	0	0	0	0	0	0	0	0	0	1	1	1	0	0	2	0	1	1	1
3821	2020	3	F	51	0	0	0	0	0	0	0	0	1	0	0	0	0	0	0	2	0	1	1	1
3822	2021	1	F	51	0	0	0	0	0	0	0	0	1	0	0	0	0	0	0	1	0	0	1	1
3823	2022	6	F	51	0	0	0	0	0	0	0	0	1	0	0	0	0	0	0	NA	0	0	0	0
3824	2023	9	F	51	0	0	0	0	0	0	0	0	1	0	0	0	0	0	0	2	1	1	0	1
3825	2021	1	M	51	0	0	1	0	0	0	0	0	0	0	1	1	0	0	0	NA	0	0	0	0
3826	2023	9	F	51	1	0	0	0	0	0	0	0	0	0	1	0	0	0	0	2	0	1	1	0
3827	2021	9	M	51	0	0	1	0	0	0	0	0	0	1	1	0	0	1	1	1	0	0	1	0
3828	2024	8	M	51	0	1	1	0	0	0	0	0	0	0	1	0	0	0	0	1	0	1	1	1
3829	2020	2	M	51	1	0	0	1	1	0	0	0	0	0	0	0	0	0	1	NA	0	0	0	0
3830	2021	10	M	51	0	0	1	1	0	1	0	0	0	0	1	1	0	0	0	1	0	1	1	0
3831	2023	3	F	51	1	0	1	1	0	0	0	0	0	0	1	0	1	0	0	2	0	1	1	1
3832	2021	12	F	51	1	1	1	0	0	0	0	1	0	0	1	1	0	0	0	2	0	1	1	0
3833	2024	5	F	51	0	1	1	0	1	1	0	0	0	0	1	1	1	0	0	2	1	1	1	1
3834	2020	1	F	52	0	0	0	0	0	0	0	0	0	0	0	0	0	0	1	1	0	1	1	1
3835	2020	2	F	52	0	0	0	0	0	0	0	0	0	0	0	0	0	0	1	NA	0	0	0	0
3836	2020	2	M	52	0	0	0	0	0	0	0	0	0	0	0	0	0	0	1	2	1	1	1	1
3837	2020	8	F	52	0	0	0	0	0	0	0	0	0	0	0	0	0	0	1	2	1	1	1	1
3838	2020	10	F	52	0	0	0	0	0	0	0	0	0	0	1	0	0	0	0	NA	0	0	0	0
3839	2020	10	M	52	0	0	0	0	0	0	0	0	0	0	0	0	0	0	0	2	1	1	1	0
3840	2021	4	F	52	0	0	0	0	0	0	0	0	0	0	0	0	0	0	1	1	0	0	1	0
3841	2021	7	F	52	0	0	0	0	0	0	0	0	0	0	1	0	0	0	0	NA	0	0	0	0
3842	2021	11	F	52	0	0	0	0	0	0	0	0	0	0	1	0	0	0	0	2	0	1	1	0
3843	2021	10	F	52	0	0	0	0	0	0	0	0	0	0	0	0	0	1	0	1	0	1	1	0

ID	Yr	Mo	Sex	Age	Tre	Grs	Rag	Mug	HDM	Dan	Mld	Food	Drg	Ven	Rhi	Cnj	Ast	AsE	Urt	Sev	Prt	Obs	Rho	Snz
3844	2021	8	F	52	0	0	0	0	0	0	0	0	0	0	0	0	0	1	0	1	0	1	1	1
3845	2022	2	F	52	0	0	0	0	0	0	0	0	0	0	1	0	1	0	0	2	0	1	1	1
3846	2022	2	F	52	0	0	0	0	0	0	0	0	0	0	0	0	0	0	0	NA	0	0	0	0
3847	2022	3	F	52	0	0	0	0	0	0	0	0	0	0	0	0	0	0	1	1	0	1	0	0
3848	2022	4	F	52	0	0	0	0	0	0	0	0	0	0	0	0	0	0	0	1	0	1	0	0
3849	2022	4	F	52	0	0	0	0	0	0	0	0	0	0	1	0	0	0	0	1	0	1	1	0
3850	2022	5	M	52	0	0	0	0	0	0	0	0	0	0	0	0	0	0	0	NA	0	0	0	0
3851	2022	5	F	52	0	0	0	0	0	0	0	0	0	0	0	0	0	0	1	2	1	1	1	1
3852	2022	5	F	52	0	0	0	0	0	0	0	0	0	0	0	0	0	0	1	1	1	1	0	0
3853	2022	6	M	52	0	0	0	0	0	0	0	0	0	0	1	1	0	0	0	2	0	1	1	1
3854	2022	9	F	52	0	0	0	0	0	0	0	0	0	0	0	0	0	0	0	1	0	1	1	0
3855	2022	9	F	52	0	0	0	0	0	0	0	0	0	0	0	0	0	0	0	NA	0	0	0	0
3856	2022	10	F	52	0	0	0	0	0	0	0	0	0	0	0	0	0	0	1	1	0	1	1	0
3857	2023	3	F	52	0	0	0	0	0	0	0	0	0	0	0	0	0	0	1	1	0	1	1	0
3858	2023	8	F	52	0	0	0	0	0	0	0	0	0	0	1	0	1	0	0	NA	0	0	0	0
3859	2023	12	M	52	0	0	0	0	0	0	0	0	0	0	0	0	0	0	0	1	0	0	1	1
3860	2024	1	F	52	0	0	0	0	0	0	0	0	0	0	0	0	0	0	1	2	1	1	1	1
3861	2024	2	F	52	0	0	0	0	0	0	0	0	0	0	1	0	0	0	0	2	0	1	1	1
3862	2024	6	F	52	0	0	0	0	0	0	0	0	0	0	1	0	1	0	0	1	0	0	1	0
3863	2023	10	F	52	0	0	0	0	0	0	0	0	0	0	0	0	0	0	1	2	0	1	1	1
3864	2020	1	F	52	0	0	0	0	0	0	0	0	1	0	0	0	0	0	0	NA	0	0	0	0
3865	2020	2	F	52	0	0	0	0	0	0	0	0	1	0	0	0	0	0	1	NA	0	0	0	0
3866	2021	7	F	52	0	0	0	0	0	0	0	0	1	0	0	0	0	0	1	2	1	0	1	1
3867	2021	11	F	52	0	0	0	0	0	0	0	0	1	0	1	1	0	0	0	2	1	1	1	1
3868	2021	9	F	52	0	0	0	0	0	0	0	0	0	1	1	0	0	0	0	1	1	1	0	0
3869	2020	9	F	52	0	0	0	0	0	1	0	0	0	0	1	1	0	0	0	NA	0	0	0	0
3870	2021	1	F	52	0	1	0	0	0	0	0	0	0	0	0	0	1	0	1	NA	0	0	0	0
3871	2020	2	F	52	0	0	1	0	0	0	0	0	0	0	1	1	1	0	1	NA	0	0	0	0
3872	2020	5	F	52	0	0	1	0	0	0	0	0	0	0	1	0	0	0	0	2	0	1	1	1
3873	2020	8	F	52	0	0	1	0	0	0	0	0	0	0	1	1	0	0	0	NA	0	0	0	0
3874	2021	1	M	52	0	0	1	0	0	0	0	0	0	0	1	1	1	0	0	NA	0	0	0	0
3875	2022	5	M	52	0	0	1	0	0	0	0	0	0	0	1	1	0	0	0	NA	0	0	0	0
3876	2022	2	F	52	0	1	0	0	0	0	0	0	0	0	1	0	1	0	0	NA	0	1	0	0
3877	2023	4	M	52	0	0	0	0	1	0	0	0	0	0	1	0	0	0	0	NA	0	0	0	0
3878	2024	7	F	52	0	0	0	0	0	0	1	0	0	0	1	0	1	0	0	2	0	1	1	1
3879	2021	11	F	52	0	0	1	0	1	0	0	0	0	0	1	1	1	0	0	NA	0	0	0	0
3880	2023	2	M	52	0	1	0	0	1	0	0	0	0	0	1	1	0	0	0	NA	0	0	0	0
3881	2023	5	F	52	1	0	0	0	0	0	1	0	0	0	1	0	0	0	0	1	0	1	0	0
3882	2024	8	M	52	0	0	0	0	1	1	0	0	0	0	1	1	0	0	0	2	0	1	1	0
3883	2023	11	F	52	1	0	0	0	0	1	0	0	1	0	0	0	0	0	1	2	1	1	0	0
3884	2024	1	F	52	0	1	1	0	1	0	0	0	0	0	1	1	1	0	0	NA	0	0	0	0
3885	2024	4	F	52	1	1	1	0	0	0	0	0	0	0	1	1	0	0	0	2	0	1	0	0
3886	2024	8	M	52	0	1	1	0	0	1	0	0	0	0	1	1	0	0	0	2	0	1	1	0
3887	2023	2	M	52	1	0	0	0	1	0	1	0	1	0	1	0	1	0	0	2	0	1	1	0
3888	2023	11	F	52	0	0	1	0	1	1	1	0	0	0	1	0	1	0	0	2	0	1	1	1
3889	2020	6	F	53	0	0	0	0	0	0	0	0	0	0	1	1	0	0	0	2	0	0	0	0
3890	2021	7	M	53	0	0	0	0	0	0	0	0	0	0	0	0	0	0	0	NA	0	0	0	0
3891	2021	3	M	53	0	0	0	0	0	0	0	0	0	0	0	0	0	1	1	1	1	1	1	0
3892	2022	2	F	53	0	0	0	0	0	0	0	0	0	0	0	0	0	0	0	NA	0	1	0	0
3893	2022	5	F	53	0	0	0	0	0	0	0	0	0	0	1	0	1	0	0	NA	0	0	0	0
3894	2022	5	F	53	0	0	0	0	0	0	0	0	0	0	1	0	0	0	1	NA	0	0	0	0
3895	2022	6	F	53	0	0	0	0	0	0	0	0	0	0	0	0	1	0	0	NA	0	0	0	0
3896	2023	4	F	53	0	0	0	0	0	0	0	0	0	0	1	0	1	0	1	2	0	1	0	0
3897	2023	6	F	53	0	0	0	0	0	0	0	0	0	0	0	0	1	0	0	NA	0	0	0	0
3898	2023	8	F	53	0	0	0	0	0	0	0	0	0	0	0	0	0	0	0	NA	0	1	1	1
3899	2023	10	F	53	0	0	0	0	0	0	0	0	0	0	0	0	1	0	0	NA	0	0	0	0
3900	2024	2	F	53	0	0	0	0	0	0	0	0	0	0	0	0	0	0	1	NA	0	0	0	0
3901	2024	2	M	53	0	0	0	0	0	0	0	0	0	0	0	0	0	0	1	NA	0	0	0	0
3902	2024	2	M	53	0	0	0	0	0	0	0	0	0	0	0	0	1	0	0	NA	0	0	0	0
3903	2024	5	M	53	0	0	0	0	0	0	0	0	0	0	0	0	0	0	1	2	0	0	0	0
3904	2024	5	M	53	0	0	0	0	0	0	0	0	0	0	0	0	0	0	0	2	0	1	1	0
3905	2021	7	M	53	0	0	0	0	0	0	0	0	0	1	0	0	0	0	0	NA	0	0	0	0
3906	2021	11	M	53	0	0	0	0	0	0	0	0	0	1	0	0	0	0	0	1	1	0	1	1

ID	Yr	Mo	Sex	Age	Tre	Grs	Rag	Mug	HDM	Dan	Mld	Food	Drg	Ven	Rhi	Cnj	Ast	AsE	Urt	Sev	Prt	Obs	Rho	Snz
3907	2021	2	F	53	0	0	0	0	0	0	0	0	0	1	0	0	0	0	0	NA	0	0	0	0
3908	2022	1	F	53	0	0	0	0	0	0	0	1	0	0	0	0	0	0	0	1	0	1	1	0
3909	2023	2	M	53	0	0	0	0	0	0	0	0	1	0	0	0	0	0	1	2	0	1	1	0
3910	2023	3	M	53	0	0	0	0	0	0	0	1	0	0	1	1	0	0	0	2	0	1	0	0
3911	2021	4	M	53	0	0	0	0	0	0	1	0	0	0	1	0	0	0	0	1	0	1	0	0
3912	2021	3	F	53	0	0	0	0	1	0	0	0	0	0	1	0	1	0	1	1	0	1	0	0
3913	2021	3	F	53	0	0	1	0	0	0	0	0	0	0	1	1	0	0	0	1	1	0	1	1
3914	2022	3	F	53	0	0	1	0	0	0	0	0	0	0	1	0	0	0	0	2	0	1	0	0
3915	2022	3	F	53	0	0	0	0	1	0	0	0	0	0	1	1	0	0	0	NA	0	0	0	0
3916	2023	10	M	53	0	1	0	0	0	0	0	0	0	0	1	0	1	0	0	NA	0	0	0	0
3917	2021	9	F	53	0	0	1	0	0	0	0	0	0	1	1	0	0	0	0	2	0	1	0	0
3918	2022	9	M	53	0	1	1	0	0	0	0	0	0	0	1	1	0	0	1	NA	0	0	0	0
3919	2022	9	F	53	0	0	1	0	1	0	0	0	0	0	1	1	1	0	0	NA	0	0	0	0
3920	2022	3	F	53	1	0	0	0	0	0	1	0	0	0	1	0	1	0	0	NA	0	0	0	0
3921	2023	10	M	53	1	1	0	0	0	0	0	0	0	0	1	0	1	0	0	NA	0	0	0	0
3922	2021	9	F	53	0	1	1	0	0	0	0	0	0	1	1	0	0	0	0	2	0	1	0	0
3923	2021	9	F	53	1	1	1	0	0	0	0	0	0	0	1	1	0	0	0	NA	0	0	0	0
3924	2020	1	M	54	0	0	0	0	0	0	0	0	0	0	0	0	0	0	1	2	1	0	1	1
3925	2020	1	M	54	0	0	0	0	0	0	0	0	0	0	0	1	0	0	0	NA	0	0	0	0
3926	2020	6	M	54	0	0	0	0	0	0	0	0	0	0	1	0	0	0	0	NA	0	0	0	0
3927	2020	7	F	54	0	0	0	0	0	0	0	0	0	0	1	0	0	0	0	NA	0	1	0	0
3928	2020	7	F	54	0	0	0	0	0	0	0	0	0	0	0	0	0	0	0	1	0	1	1	0
3929	2020	10	M	54	0	0	0	0	0	0	0	0	0	0	1	0	0	0	0	NA	0	0	0	0
3930	2020	11	F	54	0	0	0	0	0	0	0	0	0	0	1	0	0	0	0	NA	0	0	0	0
3931	2021	1	M	54	0	0	0	0	0	0	0	0	0	0	0	1	1	0	0	2	0	1	0	1
3932	2021	2	F	54	0	0	0	0	0	0	0	0	0	0	0	0	0	0	1	NA	0	0	0	0
3933	2021	8	F	54	0	0	0	0	0	0	0	0	0	0	1	0	1	0	0	NA	0	0	0	0
3934	2021	12	F	54	0	0	0	0	0	0	0	0	0	0	0	0	0	0	0	NA	0	0	0	0
3935	2021	3	F	54	0	0	0	0	0	0	0	0	0	0	0	0	1	0	1	2	0	1	0	0
3936	2022	2	F	54	0	0	0	0	0	0	0	0	0	0	0	0	1	0	0	NA	0	1	0	0
3937	2022	2	F	54	0	0	0	0	0	0	0	0	0	0	0	0	0	0	1	NA	0	0	0	0
3938	2022	3	F	54	0	0	0	0	0	0	0	0	0	0	0	0	0	0	1	1	0	0	1	1
3939	2022	8	F	54	0	0	0	0	0	0	0	0	0	0	0	0	0	0	1	1	0	0	1	1
3940	2022	9	M	54	0	0	0	0	0	0	0	0	0	0	0	0	1	0	0	1	0	1	0	0
3941	2022	10	F	54	0	0	0	0	0	0	0	0	0	0	0	0	1	0	0	2	0	1	1	1
3942	2023	2	F	54	0	0	0	0	0	0	0	0	0	0	0	0	0	0	1	1	0	0	0	0
3943	2023	2	F	54	0	0	0	0	0	0	0	0	0	0	0	0	0	0	1	2	1	1	1	1
3944	2023	2	M	54	0	0	0	0	0	0	0	0	0	0	0	0	0	0	1	NA	0	0	0	0
3945	2023	2	F	54	0	0	0	0	0	0	0	0	0	0	0	0	0	0	0	2	1	1	0	1
3946	2023	5	F	54	0	0	0	0	0	0	0	0	0	0	0	0	0	0	1	NA	0	0	0	0
3947	2023	8	M	54	0	0	0	0	0	0	0	0	0	0	0	0	0	0	1	2	0	1	0	0
3948	2023	10	M	54	0	0	0	0	0	0	0	0	0	0	1	0	0	0	0	2	0	1	0	0
3949	2024	2	F	54	0	0	0	0	0	0	0	0	0	0	0	0	0	0	0	NA	0	0	0	0
3950	2024	3	F	54	0	0	0	0	0	0	0	0	0	0	0	0	0	0	1	2	1	0	1	1
3951	2024	3	F	54	0	0	0	0	0	0	0	0	0	0	1	0	0	0	0	2	0	0	1	1
3952	2024	4	M	54	0	0	0	0	0	0	0	0	0	0	0	0	0	0	0	2	0	1	0	1
3953	2024	5	F	54	0	0	0	0	0	0	0	0	0	0	1	0	0	0	0	NA	0	0	0	0
3954	2024	6	F	54	0	0	0	0	0	0	0	0	0	0	0	0	0	0	1	2	0	0	1	1
3955	2024	7	F	54	0	0	0	0	0	0	0	0	0	0	0	0	0	0	0	2	0	0	1	1
3956	2024	7	F	54	0	0	0	0	0	0	0	0	0	0	0	0	0	0	0	2	0	1	1	1
3957	2024	10	F	54	0	0	0	0	0	0	0	0	0	0	0	0	0	0	0	2	0	1	1	1
3958	2024	10	M	54	0	0	0	0	0	0	0	0	0	0	0	0	0	0	0	2	0	0	0	0
3959	2024	10	F	54	0	0	0	0	0	0	0	0	0	0	0	0	0	0	1	2	1	1	0	0
3960	2020	9	M	54	0	0	0	0	0	0	0	0	1	0	0	0	0	0	0	2	0	1	0	1
3961	2021	7	F	54	0	0	0	0	0	0	0	0	1	0	0	0	0	0	0	1	0	1	0	0
3962	2022	7	M	54	0	0	0	0	0	0	0	0	0	1	0	0	0	0	1	NA	0	0	0	0
3963	2022	12	F	54	0	0	0	0	0	0	0	0	1	0	0	0	1	0	0	2	1	1	1	1
3964	2023	5	F	54	0	0	0	0	0	0	0	1	0	0	0	0	0	0	1	NA	0	0	0	0
3965	2023	5	F	54	0	0	0	0	0	0	0	0	1	0	0	0	0	0	1	NA	0	0	0	0
3966	2020	11	F	54	0	0	0	0	1	0	0	0	0	0	1	0	0	0	0	NA	0	0	0	0
3967	2023	4	F	54	0	0	1	0	0	0	0	0	0	0	1	0	1	0	0	NA	0	0	0	0
3968	2024	9	M	54	0	0	1	0	0	0	0	0	0	0	1	1	0	0	1	2	0	0	0	0
3969	2024	4	M	54	0	0	0	0	1	0	0	0	0	0	1	0	0	0	0	2	2	1	0	1

ID	Yr	Mo	Sex	Age	Tre	Grs	Rag	Mug	HDM	Dan	Mld	Food	Drg	Ven	Rhi	Cnj	Ast	AsE	Urt	Sev	Prt	Obs	Rho	Snz
3970	2024	10	F	54	0	0	0	0	1	0	0	0	0	0	1	0	0	0	0	2	1	0	1	1
3971	2021	10	F	54	0	0	0	0	0	0	1	0	0	1	1	1	0	0	1	1	0	0	0	0
3972	2021	11	M	54	0	0	1	0	0	0	0	0	1	1	0	1	0	0	0	2	1	0	0	0
3973	2022	2	F	54	1	0	1	0	0	0	0	0	0	0	1	1	0	0	0	2	1	1	0	1
3974	2023	8	M	54	0	0	1	1	0	0	0	0	0	0	1	1	0	0	0	2	0	0	0	0
3975	2023	9	M	54	0	0	1	1	1	0	0	0	0	0	1	1	0	0	1	2	1	1	1	0
3976	2020	8	M	55	0	0	0	0	0	0	0	0	0	0	1	1	0	0	0	NA	0	0	0	0
3977	2020	9	M	55	0	0	0	0	0	0	0	0	0	0	1	1	0	0	0	2	0	1	1	0
3978	2020	12	F	55	0	0	0	0	0	0	0	0	0	0	0	0	0	0	0	2	1	1	1	1
3979	2021	10	F	55	0	0	0	0	0	0	0	0	0	0	1	0	0	0	1	2	0	1	0	1
3980	2021	11	F	55	0	0	0	0	0	0	0	0	0	0	0	0	1	0	0	2	0	0	1	1
3981	2022	2	M	55	0	0	0	0	0	0	0	0	0	0	0	0	0	0	0	2	0	0	0	0
3982	2022	7	M	55	0	0	0	0	0	0	0	0	0	0	0	0	0	0	0	1	0	0	0	0
3983	2022	8	M	55	0	0	0	0	0	0	0	0	0	0	1	0	0	0	1	2	0	0	0	0
3984	2022	8	F	55	0	0	0	0	0	0	0	0	0	0	0	0	0	0	1	NA	0	0	0	0
3985	2022	9	F	55	0	0	0	0	0	0	0	0	0	0	0	0	0	0	0	NA	0	0	0	0
3986	2022	11	F	55	0	0	0	0	0	0	0	0	0	0	0	0	0	0	0	2	0	1	0	1
3987	2022	12	F	55	0	0	0	0	0	0	0	0	0	0	0	0	0	0	1	2	0	1	0	0
3988	2023	1	F	55	0	0	0	0	0	0	0	0	0	0	0	0	1	0	1	NA	0	0	0	0
3989	2023	3	M	55	0	0	0	0	0	0	0	0	0	0	1	0	1	0	0	2	1	1	0	0
3990	2023	4	F	55	0	0	0	0	0	0	0	0	0	0	0	0	0	0	1	NA	0	0	0	0
3991	2023	4	F	55	0	0	0	0	0	0	0	0	0	0	0	0	0	0	0	2	0	1	0	0
3992	2023	5	F	55	0	0	0	0	0	0	0	0	0	0	0	0	1	0	0	2	1	1	0	1
3993	2023	8	M	55	0	0	0	0	0	0	0	0	0	0	0	0	0	0	1	2	0	0	0	0
3994	2023	9	M	55	0	0	0	0	0	0	0	0	0	0	0	0	0	0	1	2	0	0	1	1
3995	2024	2	F	55	0	0	0	0	0	0	0	0	0	0	1	0	0	0	0	2	1	1	1	1
3996	2024	8	M	55	0	0	0	0	0	0	0	0	0	0	1	0	1	0	0	NA	0	0	0	0
3997	2024	9	F	55	0	0	0	0	0	0	0	0	0	0	1	0	1	0	0	2	0	1	1	1
3998	2024	10	M	55	0	0	0	0	0	0	0	0	0	0	0	0	0	0	1	NA	0	0	0	0
3999	2024	12	M	55	0	0	0	0	0	0	0	0	0	0	0	0	0	0	0	2	1	1	0	0
4000	2021	10	F	55	0	0	0	0	0	0	0	0	1	0	0	0	0	0	0	NA	0	0	0	0
4001	2022	7	F	55	0	0	0	0	0	0	0	0	1	0	0	0	0	0	0	NA	0	0	0	0
4002	2023	8	F	55	0	0	1	0	0	0	0	0	0	0	1	0	0	0	1	2	0	1	0	0
4003	2021	7	M	55	0	0	1	0	0	0	0	1	0	0	0	0	0	0	1	2	1	1	1	1
4004	2022	7	F	55	0	0	0	0	1	0	0	0	0	1	0	0	1	0	1	2	0	1	0	1
4005	2023	5	F	55	0	0	1	0	0	0	0	1	1	0	1	0	1	0	1	2	0	0	0	0
4006	2021	11	F	55	0	1	1	0	0	0	0	0	0	1	1	0	0	0	0	2	0	1	1	1
4007	2020	11	F	55	1	1	1	0	0	0	0	0	0	0	1	1	0	0	0	2	0	1	0	0
4008	2022	5	M	55	1	1	1	0	1	0	0	0	0	0	1	1	0	1	0	2	0	1	1	1
4009	2023	3	M	55	1	1	1	1	0	0	0	0	0	0	1	0	0	0	1	2	1	1	1	1
4010	2020	11	F	56	0	0	0	0	0	0	0	0	0	0	1	0	0	0	1	1	0	1	0	0
4011	2020	11	F	56	0	0	0	0	0	0	0	0	0	0	0	0	1	0	0	1	1	0	0	0
4012	2021	12	F	56	0	0	0	0	0	0	0	0	0	0	0	0	0	0	0	2	0	1	0	0
4013	2022	1	F	56	0	0	0	0	0	0	0	0	0	0	0	1	0	0	0	1	0	0	0	0
4014	2022	2	M	56	0	0	0	0	0	0	0	0	0	0	0	0	0	0	0	2	1	1	0	1
4015	2022	3	F	56	0	0	0	0	0	0	0	0	0	0	0	0	0	0	0	2	1	1	1	1
4016	2023	6	F	56	0	0	0	0	0	0	0	0	0	0	1	0	0	0	0	1	0	0	1	0
4017	2023	9	F	56	0	0	0	0	0	0	0	0	0	0	0	0	0	0	1	1	0	1	0	0
4018	2024	1	F	56	0	0	0	0	0	0	0	0	0	0	0	0	0	0	0	2	0	1	0	1
4019	2024	2	F	56	0	0	0	0	0	0	0	0	0	0	0	0	0	0	0	1	0	0	0	0
4020	2024	5	F	56	0	0	0	0	0	0	0	0	0	0	0	0	0	0	0	2	1	0	0	0
4021	2024	9	F	56	0	0	0	0	0	0	0	0	0	0	0	0	0	0	0	1	0	0	0	0
4022	2021	11	F	56	0	0	0	0	0	0	0	1	1	0	0	0	0	0	0	1	0	1	0	0
4023	2021	10	F	56	0	0	0	0	1	0	0	0	0	0	1	0	0	0	1	1	0	1	0	0
4024	2024	7	M	56	0	0	1	0	0	0	0	0	0	1	1	1	0	0	0	2	1	0	0	0
4025	2022	3	M	56	0	0	1	0	1	0	0	0	0	0	1	0	1	0	0	NA	0	0	0	0
4026	2024	9	M	56	0	1	1	0	0	1	0	0	0	0	1	1	1	1	0	NA	0	0	0	0
4027	2024	2	M	56	1	1	0	0	1	0	1	0	0	0	1	0	0	0	1	1	0	1	1	0
4028	2020	8	F	57	0	0	0	0	0	0	0	0	0	0	0	0	0	0	0	1	1	0	0	0
4029	2020	9	M	57	0	0	0	0	0	0	0	0	0	0	1	1	0	0	0	1	1	0	0	1
4030	2021	2	F	57	0	0	0	0	0	0	0	0	0	0	0	0	0	0	0	NA	0	0	0	0
4031	2021	4	M	57	0	0	0	0	0	0	0	0	0	0	0	0	0	0	0	NA	0	0	0	0
4032	2021	7	M	57	0	0	0	0	0	0	0	0	0	0	0	0	1	0	0	1	0	1	1	1

ID	Yr	Mo	Sex	Age	Tre	Grs	Rag	Mug	HDM	Dan	Mld	Food	Drg	Ven	Rhi	Cnj	Ast	AsE	Urt	Sev	Prt	Obs	Rho	Snz
4033	2021	8	F	57	0	0	0	0	0	0	0	0	0	0	0	0	0	0	1	1	0	1	0	0
4034	2021	10	F	57	0	0	0	0	0	0	0	0	0	0	1	0	0	0	0	1	0	1	0	0
4035	2021	10	F	57	0	0	0	0	0	0	0	0	0	0	0	0	0	0	0	1	1	1	1	0
4036	2021	9	M	57	0	0	0	0	0	0	0	0	0	0	0	0	0	1	0	1	1	1	1	1
4037	2021	10	F	57	0	0	0	0	0	0	0	0	0	0	0	0	0	0	0	2	0	0	0	1
4038	2021	3	F	57	0	0	0	0	0	0	0	0	0	0	0	0	1	0	1	2	1	0	1	1
4039	2023	3	F	57	0	0	0	0	0	0	0	0	0	0	1	0	0	0	1	1	0	0	0	0
4040	2023	4	F	57	0	0	0	0	0	0	0	0	0	0	0	0	0	0	0	1	1	0	1	1
4041	2023	7	F	57	0	0	0	0	0	0	0	0	0	0	1	0	1	0	1	2	1	1	1	1
4042	2023	9	F	57	0	0	0	0	0	0	0	0	0	0	0	0	1	0	1	1	0	1	0	0
4043	2023	11	F	57	0	0	0	0	0	0	0	0	0	0	1	0	0	0	0	1	0	1	1	1
4044	2023	11	F	57	0	0	0	0	0	0	0	0	0	0	0	0	0	0	1	2	0	1	1	1
4045	2024	6	M	57	0	0	0	0	0	0	0	0	0	0	0	0	0	0	1	2	1	1	0	0
4046	2024	7	F	57	0	0	0	0	0	0	0	0	0	0	0	0	0	0	0	2	0	1	1	1
4047	2024	7	M	57	0	0	0	0	0	0	0	0	0	0	1	0	0	0	0	1	0	0	0	0
4048	2024	7	F	57	0	0	0	0	0	0	0	0	0	0	0	0	1	0	0	1	1	1	1	0
4049	2024	8	F	57	0	0	0	0	0	0	0	0	0	0	0	0	0	0	0	NA	0	0	0	0
4050	2024	9	F	57	0	0	0	0	0	0	0	0	0	0	0	0	1	0	0	1	0	1	0	0
4051	2024	12	F	57	0	0	0	0	0	0	0	0	0	0	0	0	1	0	0	NA	0	0	0	0
4052	2021	7	F	57	0	0	0	0	0	0	0	0	1	0	0	0	0	0	1	1	0	1	1	1
4053	2024	6	F	57	0	0	0	0	0	0	0	0	1	0	0	0	0	0	0	NA	0	0	0	0
4054	2020	1	M	57	0	0	1	0	0	0	1	0	0	0	1	1	0	0	0	2	1	1	0	1
4055	2021	1	F	57	0	0	1	0	0	1	0	0	0	0	1	1	1	0	0	2	0	1	0	0
4056	2023	1	M	57	0	1	1	0	0	0	0	0	0	0	1	0	0	0	0	1	0	0	0	0
4057	2022	10	F	57	0	0	0	0	0	1	1	0	0	0	1	0	1	0	0	1	0	1	0	0
4058	2022	10	F	57	1	1	1	0	0	0	0	0	0	0	1	0	1	0	0	NA	0	0	0	0
4059	2023	5	F	57	0	0	1	0	1	1	0	0	0	0	1	0	1	0	0	1	0	1	0	0
4060	2024	12	F	57	1	1	1	0	0	1	1	0	0	0	1	1	1	0	0	NA	0	0	0	0
4061	2020	1	F	58	0	0	0	0	0	0	0	0	0	0	0	0	1	0	0	1	0	1	1	1
4062	2020	3	F	58	0	0	0	0	0	0	0	0	0	0	0	0	0	0	1	NA	0	0	0	0
4063	2020	10	F	58	0	0	0	0	0	0	0	0	0	0	0	0	0	0	0	1	0	1	0	0
4064	2021	2	M	58	0	0	0	0	0	0	0	0	0	0	0	0	0	0	0	1	0	0	1	0
4065	2021	2	M	58	0	0	0	0	0	0	0	0	0	0	0	0	0	1	0	1	1	0	0	0
4066	2022	1	F	58	0	0	0	0	0	0	0	0	0	0	0	0	0	0	0	2	0	1	1	1
4067	2022	2	M	58	0	0	0	0	0	0	0	0	0	0	1	0	1	0	0	1	1	0	0	0
4068	2022	3	F	58	0	0	0	0	0	0	0	0	0	0	1	0	1	0	0	1	1	0	0	0
4069	2022	12	F	58	0	0	0	0	0	0	0	0	0	0	0	0	0	0	1	1	0	1	1	1
4070	2023	2	F	58	0	0	0	0	0	0	0	0	0	0	0	0	0	0	0	NA	0	0	0	0
4071	2023	3	F	58	0	0	0	0	0	0	0	0	0	0	0	0	0	0	1	1	0	0	1	0
4072	2023	9	F	58	0	0	0	0	0	0	0	0	0	0	1	0	0	0	0	NA	0	0	0	0
4073	2024	2	M	58	0	0	0	0	0	0	0	0	0	0	0	0	0	0	0	NA	0	0	0	0
4074	2024	3	M	58	0	0	0	0	0	0	0	0	0	0	0	0	0	0	0	2	0	1	0	0
4075	2024	4	M	58	0	0	0	0	0	0	0	0	0	0	0	0	0	0	1	1	1	1	1	0
4076	2024	5	M	58	0	0	0	0	0	0	0	0	0	0	0	0	0	0	0	1	0	0	1	1
4077	2024	5	F	58	0	0	0	0	0	0	0	0	0	0	0	0	0	0	1	1	1	0	0	0
4078	2022	2	M	58	0	0	1	0	0	0	0	0	0	0	1	1	0	0	0	1	1	0	0	0
4079	2023	11	F	58	0	0	1	0	0	0	0	0	0	0	1	1	1	0	0	1	0	0	1	1
4080	2024	3	F	58	0	0	0	0	0	1	0	0	0	0	1	0	0	0	0	NA	0	0	0	0
4081	2023	9	F	58	0	0	1	0	0	0	0	1	0	0	1	1	0	0	1	1	0	1	0	0
4082	2020	10	F	58	1	0	1	0	0	0	0	0	0	0	1	1	0	0	0	1	0	0	1	0
4083	2020	10	F	58	1	1	1	0	0	0	0	0	0	0	1	1	0	0	0	NA	0	0	0	0
4084	2021	2	F	58	0	0	1	1	1	1	0	0	0	0	1	1	1	0	1	NA	0	0	0	0
4085	2021	2	F	59	0	0	0	0	0	0	0	0	0	0	0	0	0	0	1	NA	0	0	0	0
4086	2020	5	F	59	0	0	0	0	0	0	0	0	0	0	0	0	0	0	1	NA	0	0	0	0
4087	2022	3	F	59	0	0	0	0	0	0	0	0	0	0	0	0	0	0	1	NA	0	0	0	0
4088	2022	6	M	59	0	0	0	0	0	0	0	0	0	0	0	0	0	0	0	NA	0	0	0	0
4089	2022	10	M	59	0	0	0	0	0	0	0	0	0	0	0	0	0	0	0	2	1	1	1	0
4090	2022	10	F	59	0	0	0	0	0	0	0	0	0	0	0	0	0	0	0	NA	0	0	0	0
4091	2024	5	M	59	0	0	0	0	0	0	0	0	0	0	1	0	1	0	0	2	0	1	1	0
4092	2024	5	F	59	0	0	0	0	0	0	0	0	0	0	1	1	1	0	0	2	1	1	0	0
4093	2024	11	F	59	0	0	0	0	0	0	0	0	0	0	1	0	0	0	0	2	0	1	0	0
4094	2021	8	F	59	0	0	0	0	0	0	0	0	1	0	0	0	0	0	0	NA	0	0	0	0
4095	2021	10	F	59	0	0	0	0	0	0	0	0	0	1	0	1	0	0	0	2	0	1	1	1

ID	Yr	Mo	Sex	Age	Tre	Grs	Rag	Mug	HDM	Dan	Mld	Food	Drg	Ven	Rhi	Cnj	Ast	AsE	Urt	Sev	Prt	Obs	Rho	Snz
4096	2021	1	F	59	0	0	0	0	0	0	0	0	1	0	1	0	0	0	0	NA	0	0	0	0
4097	2022	1	M	59	0	0	0	0	0	0	0	1	0	0	1	0	1	0	1	2	0	1	0	0
4098	2020	1	M	59	0	1	0	0	0	0	0	0	0	0	1	1	0	0	0	2	0	1	1	0
4099	2022	11	F	59	0	0	1	0	0	0	0	0	0	0	1	0	0	0	0	2	0	1	0	0
4100	2024	5	F	59	0	0	1	0	0	0	0	0	0	0	1	0	0	0	0	2	0	1	1	1
4101	2024	11	M	59	0	0	1	0	0	0	0	0	0	0	1	1	1	0	0	2	0	0	0	0
4102	2022	11	F	59	0	0	0	0	0	1	0	0	0	0	1	0	0	0	1	2	0	1	1	1
4103	2021	10	M	59	0	1	0	0	1	0	0	0	0	0	1	0	0	0	0	NA	0	0	0	0
4104	2023	1	M	59	1	0	0	0	0	0	1	0	0	0	1	0	1	0	0	NA	0	0	0	0
4105	2020	2	F	60	0	0	0	0	0	0	0	0	0	0	1	1	0	0	0	1	0	0	1	0
4106	2020	3	F	60	0	0	0	0	0	0	0	0	0	0	0	0	0	0	0	1	0	1	0	0
4107	2021	4	F	60	0	0	0	0	0	0	0	0	0	0	0	0	0	0	1	NA	0	0	0	0
4108	2024	2	F	60	0	0	0	0	0	0	0	0	0	0	0	0	0	0	1	NA	0	0	0	0
4109	2024	3	F	60	0	0	0	0	0	0	0	0	0	0	0	0	1	0	0	1	0	1	1	0
4110	2024	3	M	60	0	0	0	0	0	0	0	0	0	0	0	0	0	0	1	1	0	1	0	0
4111	2024	10	F	60	0	0	0	0	0	0	0	0	0	0	0	0	0	0	0	NA	0	0	0	0
4112	2020	2	F	60	0	0	0	0	0	0	0	0	1	0	0	0	0	0	0	NA	0	0	0	0
4113	2021	6	F	60	0	0	0	0	0	0	0	0	1	0	1	0	0	0	0	NA	0	0	0	0
4114	2024	5	M	60	0	0	0	0	0	0	0	0	0	1	0	0	0	0	1	1	0	1	1	1
4115	2020	2	M	60	0	0	1	0	0	0	0	0	0	0	1	0	0	0	0	1	1	0	1	1
4116	2020	6	M	60	0	0	1	0	0	0	0	0	0	0	1	1	1	0	0	NA	0	0	0	0
4117	2022	9	F	60	0	0	1	0	0	0	1	0	0	0	1	0	1	0	0	NA	0	0	0	0
4118	2021	5	F	60	0	1	1	1	0	0	0	0	0	1	0	1	0	0	0	NA	0	0	0	0
4119	2022	12	F	60	0	1	1	0	1	1	1	0	0	0	1	0	0	0	0	NA	0	0	0	0
4120	2020	1	F	61	0	0	0	0	0	0	0	0	0	0	0	0	1	0	0	1	1	1	0	1
4121	2020	7	F	61	0	0	0	0	0	0	0	0	0	0	0	0	0	0	1	1	0	1	1	0
4122	2020	7	M	61	0	0	0	0	0	0	0	0	0	0	0	0	0	0	0	NA	0	0	0	0
4123	2021	4	M	61	0	0	0	0	0	0	0	0	0	0	0	0	0	0	0	2	0	0	0	0
4124	2021	2	M	61	0	0	0	0	0	0	0	0	0	0	0	0	1	0	1	NA	0	0	0	0
4125	2023	1	F	61	0	0	0	0	0	0	0	0	0	0	0	0	0	0	1	2	1	1	1	1
4126	2023	4	M	61	0	0	0	0	0	0	0	0	0	0	0	0	0	0	0	NA	0	0	0	0
4127	2020	6	M	61	0	0	0	0	0	0	0	1	0	0	0	0	0	0	1	1	0	1	0	0
4128	2020	2	F	61	0	0	0	0	0	0	0	0	1	1	0	0	0	0	0	1	0	1	1	0
4129	2020	9	M	61	1	0	0	0	0	0	0	0	0	0	1	1	1	0	0	NA	0	0	0	0
4130	2022	2	F	61	0	0	1	0	0	0	0	0	0	0	1	0	1	0	0	NA	0	0	0	0
4131	2022	10	F	61	0	1	0	0	0	0	0	0	0	0	0	0	1	0	0	1	0	0	1	1
4132	2021	4	F	61	0	0	0	0	1	0	1	0	0	1	0	0	0	0	0	NA	0	0	0	0
4133	2020	8	M	62	0	0	0	0	0	0	0	0	0	0	0	0	0	0	1	NA	0	0	0	0
4134	2020	9	F	62	0	0	0	0	0	0	0	0	0	0	0	0	0	0	0	NA	0	0	0	0
4135	2021	1	M	62	0	0	0	0	0	0	0	0	0	0	0	0	0	0	1	1	0	1	0	0
4136	2021	3	F	62	0	0	0	0	0	0	0	0	0	0	1	1	0	0	0	NA	0	0	0	0
4137	2021	4	F	62	0	0	0	0	0	0	0	0	0	0	0	0	0	0	0	NA	0	0	0	0
4138	2021	9	F	62	0	0	0	0	0	0	0	0	0	0	0	0	0	1	0	1	0	1	1	1
4139	2021	9	M	62	0	0	0	0	0	0	0	0	0	0	0	0	0	0	0	NA	0	0	0	0
4140	2021	10	M	62	0	0	0	0	0	0	0	0	0	0	1	1	0	0	0	NA	0	0	0	0
4141	2021	10	F	62	0	0	0	0	0	0	0	0	0	0	0	0	0	1	0	NA	0	0	0	0
4142	2022	3	F	62	0	0	0	0	0	0	0	0	0	0	0	0	0	0	0	2	0	1	1	1
4143	2022	3	M	62	0	0	0	0	0	0	0	0	0	0	0	0	0	0	1	1	0	0	1	0
4144	2022	5	M	62	0	0	0	0	0	0	0	0	0	0	1	0	0	0	1	NA	0	0	0	0
4145	2022	8	F	62	0	0	0	0	0	0	0	0	0	0	0	0	0	0	1	2	1	0	0	0
4146	2022	9	F	62	0	0	0	0	0	0	0	0	0	0	0	0	1	0	0	2	0	0	0	0
4147	2022	10	F	62	0	0	0	0	0	0	0	0	0	0	1	0	0	0	0	2	0	1	0	1
4148	2022	10	F	62	0	0	0	0	0	0	0	0	0	0	0	0	0	0	0	2	0	1	0	0
4149	2022	10	F	62	0	0	0	0	0	0	0	0	0	0	0	0	0	0	0	1	0	1	1	0
4150	2022	12	M	62	0	0	0	0	0	0	0	0	0	0	0	0	0	0	1	2	0	0	0	0
4151	2023	5	F	62	0	0	0	0	0	0	0	0	0	0	0	0	0	0	1	2	1	0	0	0
4152	2023	6	M	62	0	0	0	0	0	0	0	0	0	0	0	0	0	0	1	NA	1	1	0	1
4153	2023	7	F	62	0	0	0	0	0	0	0	0	0	0	1	0	0	0	0	1	0	1	0	1
4154	2023	9	F	62	0	0	0	0	0	0	0	0	0	0	0	0	0	0	0	2	1	0	0	0
4155	2023	11	F	62	0	0	0	0	0	0	0	0	0	0	0	0	1	0	0	1	0	0	0	0
4156	2024	3	F	62	0	0	0	0	0	0	0	0	0	0	0	0	1	0	0	2	0	0	0	0
4157	2020	8	M	62	0	0	0	0	0	0	0	0	1	0	1	1	0	0	0	1	0	0	0	0
4158	2021	10	M	62	0	0	0	0	0	0	0	0	1	0	0	0	0	0	0	1	0	0	1	0

ID	Yr	Mo	Sex	Age	Tre	Grs	Rag	Mug	HDM	Dan	Mld	Food	Drg	Ven	Rhi	Cnj	Ast	AsE	Urt	Sev	Prt	Obs	Rho	Snz
4159	2021	11	F	62	0	0	0	0	0	0	0	0	0	1	0	1	0	0	0	1	0	0	1	1
4160	2024	1	M	62	0	0	0	0	0	0	0	0	0	1	0	0	0	0	0	NA	0	0	0	0
4161	2024	5	F	62	0	0	0	0	0	0	0	0	1	0	0	0	0	0	0	2	0	1	1	0
4162	2020	8	M	62	0	0	1	0	0	0	0	0	0	0	1	1	0	0	0	2	0	0	1	1
4163	2023	8	M	62	0	0	1	0	0	0	0	0	0	0	1	0	0	0	0	1	0	1	0	0
4164	2023	6	F	62	0	0	1	0	0	0	0	0	0	1	1	0	1	0	1	NA	0	0	0	0
4165	2022	9	F	62	0	1	1	0	0	0	0	0	0	0	1	0	1	0	0	NA	0	0	0	0
4166	2023	10	M	62	0	0	0	0	1	0	1	0	0	0	1	1	1	0	0	NA	0	0	0	0
4167	2021	5	M	62	0	1	0	0	1	0	0	0	0	1	1	0	0	0	0	2	0	0	0	0
4168	2023	8	F	62	0	1	1	1	0	0	0	0	0	0	1	0	1	0	0	NA	0	0	0	0
4169	2020	1	F	63	0	0	0	0	0	0	0	0	0	0	0	0	0	0	0	NA	0	0	0	0
4170	2020	2	F	63	0	0	0	0	0	0	0	0	0	0	0	0	0	0	1	NA	0	0	0	0
4171	2020	10	F	63	0	0	0	0	0	0	0	0	0	0	0	0	1	0	0	2	0	0	0	0
4172	2020	10	F	63	0	0	0	0	0	0	0	0	0	0	0	0	0	0	0	2	0	1	0	0
4173	2021	2	F	63	0	0	0	0	0	0	0	0	0	0	0	0	0	0	0	2	0	1	0	0
4174	2021	4	F	63	0	0	0	0	0	0	0	0	0	0	0	1	0	1	0	2	0	0	1	1
4175	2022	4	M	63	0	0	0	0	0	0	0	0	0	0	0	1	0	0	0	2	0	1	0	0
4176	2022	5	F	63	0	0	0	0	0	0	0	0	0	0	0	0	0	0	0	2	0	1	0	1
4177	2022	6	F	63	0	0	0	0	0	0	0	0	0	0	1	0	1	0	0	2	0	1	0	0
4178	2022	8	M	63	0	0	0	0	0	0	0	0	0	0	0	0	0	0	1	1	0	0	0	0
4179	2022	11	F	63	0	0	0	0	0	0	0	0	0	0	0	0	0	0	0	2	1	1	1	1
4180	2022	11	M	63	0	0	0	0	0	0	0	0	0	0	0	0	0	0	0	NA	0	0	0	0
4181	2023	11	F	63	0	0	0	0	0	0	0	0	0	0	0	0	0	0	1	2	0	1	1	1
4182	2024	2	M	63	0	0	0	0	0	0	0	0	0	0	0	0	0	0	1	2	1	1	0	0
4183	2020	2	F	63	0	0	0	0	0	0	0	0	1	0	1	0	0	0	0	2	0	0	0	0
4184	2024	11	F	63	0	0	0	0	0	0	0	0	1	0	0	0	0	0	0	NA	0	0	0	0
4185	2020	8	M	63	0	1	0	0	0	0	0	0	0	0	1	0	0	0	0	NA	0	0	0	0
4186	2020	9	M	63	0	0	0	0	1	0	0	0	0	0	1	0	0	0	0	NA	0	0	0	0
4187	2023	9	F	63	0	1	0	0	0	0	0	0	0	0	1	0	1	0	0	2	0	0	1	1
4188	2020	11	F	63	1	1	0	0	0	0	0	0	0	0	1	0	1	0	0	2	0	0	0	0
4189	2023	10	F	63	1	0	1	0	0	0	1	0	0	0	1	0	1	1	1	1	1	1	0	0
4190	2020	2	M	63	1	1	1	0	1	0	0	0	0	0	1	1	0	0	1	1	0	1	0	0
4191	2020	1	F	64	0	0	0	0	0	0	0	0	0	0	1	1	1	0	1	NA	0	0	0	0
4192	2020	2	F	64	0	0	0	0	0	0	0	0	0	0	0	0	0	0	1	2	0	1	0	0
4193	2020	6	F	64	0	0	0	0	0	0	0	0	0	0	0	0	0	0	0	1	1	0	0	1
4194	2021	2	F	64	0	0	0	0	0	0	0	0	0	0	0	0	0	0	1	NA	0	0	0	0
4195	2021	3	F	64	0	0	0	0	0	0	0	0	0	0	0	0	0	0	0	2	0	0	0	1
4196	2021	5	M	64	0	0	0	0	0	0	0	0	0	0	0	0	1	1	0	1	0	1	0	0
4197	2021	9	M	64	0	0	0	0	0	0	0	0	0	0	1	0	1	0	0	2	0	1	1	1
4198	2021	9	F	64	0	0	0	0	0	0	0	0	0	0	0	0	0	1	0	NA	0	0	0	0
4199	2021	2	M	64	0	0	0	0	0	0	0	0	0	0	1	0	1	0	1	NA	0	0	0	0
4200	2021	6	M	64	0	0	0	0	0	0	0	0	0	0	0	0	0	0	0	2	0	1	0	0
4201	2022	3	M	64	0	0	0	0	0	0	0	0	0	0	0	0	0	0	0	NA	0	0	0	0
4202	2022	6	M	64	0	0	0	0	0	0	0	0	0	0	0	0	0	0	0	2	1	1	0	1
4203	2022	9	M	64	0	0	0	0	0	0	0	0	0	0	1	0	0	0	0	1	0	0	0	0
4204	2022	10	F	64	0	0	0	0	0	0	0	0	0	0	0	0	1	0	0	2	0	0	0	0
4205	2023	4	F	64	0	0	0	0	0	0	0	0	0	0	0	0	1	0	0	2	1	1	0	1
4206	2023	10	F	64	0	0	0	0	0	0	0	0	0	0	0	0	0	0	1	1	0	0	0	0
4207	2023	11	F	64	0	0	0	0	0	0	0	0	0	0	1	0	1	0	0	1	0	1	0	1
4208	2023	11	F	64	0	0	0	0	0	0	0	0	0	0	0	0	1	0	0	1	0	1	0	0
4209	2024	6	M	64	0	0	0	0	0	0	0	0	0	0	0	0	0	0	1	2	0	0	0	0
4210	2024	9	F	64	0	0	0	0	0	0	0	0	0	0	0	0	0	0	0	2	0	1	0	0
4211	2021	10	M	64	0	0	0	0	0	0	0	0	0	1	0	1	0	0	0	1	0	0	1	1
4212	2021	3	F	64	0	0	0	0	0	0	0	0	1	1	1	1	0	0	0	1	1	0	1	0
4213	2020	8	M	64	0	0	1	0	0	0	0	0	0	0	1	1	0	0	0	2	1	1	0	1
4214	2021	9	F	64	0	0	1	0	0	0	0	0	0	0	1	1	0	0	0	1	0	0	0	0
4215	2021	1	F	64	0	0	0	0	0	0	1	0	1	0	1	0	0	0	0	2	0	1	0	0
4216	2021	2	F	64	0	0	1	1	1	0	1	0	0	1	1	1	1	0	0	1	0	1	1	0
4217	2020	2	M	65	0	0	0	0	0	0	0	0	0	0	1	0	0	0	0	NA	0	0	0	0
4218	2020	9	F	65	0	0	0	0	0	0	0	0	0	0	0	0	0	0	1	NA	0	0	0	0
4219	2020	9	F	65	0	0	0	0	0	0	0	0	0	0	1	1	0	0	0	2	0	1	0	0
4220	2021	9	M	65	0	0	0	0	0	0	0	0	0	0	1	0	1	0	0	NA	0	0	0	0
4221	2021	10	F	65	0	0	0	0	0	0	0	0	0	0	0	0	1	0	1	2	0	0	1	1

ID	Yr	Mo	Sex	Age	Tre	Grs	Rag	Mug	HDM	Dan	Mld	Food	Drg	Ven	Rhi	Cnj	Ast	AsE	Urt	Sev	Prt	Obs	Rho	Snz
4222	2021	5	M	65	0	0	0	0	0	0	0	0	0	0	0	0	0	1	0	NA	0	0	0	0
4223	2022	1	F	65	0	0	0	0	0	0	0	0	0	0	0	0	0	0	1	NA	0	0	0	0
4224	2022	4	F	65	0	0	0	0	0	0	0	0	0	0	0	0	0	0	0	NA	0	0	0	0
4225	2022	5	M	65	0	0	0	0	0	0	0	0	0	0	0	0	0	0	0	NA	0	0	0	0
4226	2022	11	F	65	0	0	0	0	0	0	0	0	0	0	0	0	0	0	1	1	0	1	0	0
4227	2023	3	M	65	0	0	0	0	0	0	0	0	0	0	0	0	0	0	1	2	0	1	0	0
4228	2023	4	M	65	0	0	0	0	0	0	0	0	0	0	0	0	0	0	0	2	0	0	0	0
4229	2023	4	M	65	0	0	0	0	0	0	0	0	0	0	0	0	0	0	0	2	0	1	1	0
4230	2023	8	M	65	0	0	0	0	0	0	0	0	0	0	1	0	1	0	0	1	0	0	0	0
4231	2023	11	F	65	0	0	0	0	0	0	0	0	0	0	0	0	0	0	1	2	1	1	1	1
4232	2024	2	M	65	0	0	0	0	0	0	0	0	0	0	0	0	1	0	0	1	0	1	0	0
4233	2024	3	F	65	0	0	0	0	0	0	0	0	0	0	1	0	0	0	1	2	0	0	0	0
4234	2024	3	F	65	0	0	0	0	0	0	0	0	0	0	1	0	0	0	1	2	1	0	1	0
4235	2024	7	M	65	0	0	0	0	0	0	0	0	0	0	1	0	0	0	0	NA	0	0	0	0
4236	2021	4	F	65	0	0	0	0	0	0	0	0	1	0	0	0	0	0	1	1	0	0	1	0
4237	2020	6	F	65	0	0	1	0	0	0	0	0	0	0	1	1	0	0	0	1	1	1	1	1
4238	2023	5	F	65	0	0	1	0	0	0	0	0	0	0	1	0	0	0	0	NA	0	0	0	0
4239	2023	6	F	65	0	0	0	0	0	0	1	0	0	0	1	0	1	0	0	2	0	0	0	0
4240	2023	10	F	65	0	0	0	0	1	0	0	0	0	0	1	0	0	0	1	2	1	1	1	0
4241	2024	4	F	65	0	0	0	0	1	0	0	0	0	0	0	0	1	0	0	NA	0	0	0	0
4242	2020	1	F	65	0	0	0	0	1	0	1	0	0	0	1	1	0	0	0	2	0	1	0	1
4243	2021	3	F	65	0	0	0	0	1	1	0	0	1	0	1	0	1	0	0	2	0	1	1	0
4244	2021	2	M	65	1	0	1	0	0	0	0	1	0	1	0	0	0	0	1	2	1	0	2	0
4245	2022	11	M	65	0	0	1	0	1	1	0	0	0	0	1	1	1	0	0	2	0	0	0	0
4246	2020	3	F	66	0	0	0	0	0	0	0	0	0	0	0	0	0	0	0	NA	0	0	0	0
4247	2020	9	F	66	0	0	0	0	0	0	0	0	0	0	1	1	0	0	0	NA	0	0	0	0
4248	2020	9	F	66	0	0	0	0	0	0	0	0	0	0	0	0	0	0	0	NA	0	0	0	0
4249	2020	12	F	66	0	0	0	0	0	0	0	0	0	0	0	0	0	0	1	NA	0	0	0	0
4250	2021	10	M	66	0	0	0	0	0	0	0	0	0	0	0	0	0	0	1	NA	0	0	0	0
4251	2021	11	F	66	0	0	0	0	0	0	0	0	0	0	0	0	0	1	0	NA	0	0	0	0
4252	2022	3	F	66	0	0	0	0	0	0	0	0	0	0	1	0	0	0	0	1	0	0	0	0
4253	2022	8	M	66	0	0	0	0	0	0	0	0	0	0	0	0	0	0	1	2	0	1	0	0
4254	2023	9	M	66	0	0	0	0	0	0	0	0	0	0	1	0	0	0	0	NA	0	0	0	0
4255	2023	10	F	66	0	0	0	0	0	0	0	0	0	0	0	0	0	0	1	2	0	1	0	1
4256	2024	6	F	66	0	0	0	0	0	0	0	0	0	0	0	0	0	0	0	2	0	1	1	1
4257	2024	6	F	66	0	0	0	0	0	0	0	0	0	0	0	0	0	0	1	2	1	1	1	1
4258	2024	10	F	66	0	0	0	0	0	0	0	0	0	0	0	0	0	0	0	2	0	0	0	0
4259	2021	4	F	66	0	0	0	0	0	0	0	0	1	0	0	0	0	0	1	2	0	0	0	0
4260	2024	3	F	66	0	0	0	0	0	0	0	0	1	0	1	0	0	0	0	NA	1	0	0	0
4261	2021	1	F	66	0	0	0	0	1	0	0	0	0	0	0	0	1	0	0	2	0	1	0	0
4262	2024	8	M	66	0	1	0	0	0	0	0	0	1	0	0	0	0	0	0	NA	0	0	0	0
4263	2023	5	M	66	0	0	1	1	0	0	0	0	0	0	1	0	1	0	0	NA	0	0	0	0
4264	2024	7	M	66	0	0	1	0	0	0	1	0	0	0	1	0	0	0	0	2	0	0	0	0
4265	2024	11	M	66	1	0	1	0	0	1	0	0	0	0	1	1	0	0	0	1	0	0	1	1
4266	2020	2	F	67	0	0	0	0	0	0	0	0	0	0	0	0	0	0	0	NA	0	0	0	0
4267	2021	8	F	67	0	0	0	0	0	0	0	0	0	0	0	0	0	0	1	2	0	0	0	0
4268	2021	10	F	67	0	0	0	0	0	0	0	0	0	0	0	0	0	0	0	2	0	1	0	0
4269	2022	2	F	67	0	0	0	0	0	0	0	0	0	0	0	0	0	0	0	2	0	1	0	0
4270	2022	4	F	67	0	0	0	0	0	0	0	0	0	0	0	0	0	0	1	2	0	1	1	1
4271	2022	9	F	67	0	0	0	0	0	0	0	0	0	0	0	0	0	0	0	1	0	0	0	0
4272	2023	3	F	67	0	0	0	0	0	0	0	0	0	0	1	0	0	0	0	2	0	1	0	1
4273	2024	2	M	67	0	0	0	0	0	0	0	0	0	0	0	0	0	0	0	NA	0	0	0	0
4274	2024	7	F	67	0	0	0	0	0	0	0	0	0	0	1	0	0	0	0	2	0	1	0	0
4275	2024	12	F	67	0	0	0	0	0	0	0	0	0	0	0	0	0	0	1	2	0	0	0	0
4276	2021	5	M	67	0	0	0	0	0	0	0	0	1	0	0	0	0	0	0	NA	0	0	0	0
4277	2021	9	M	67	0	0	0	0	0	0	0	0	0	1	1	0	0	0	0	NA	0	0	0	0
4278	2021	10	F	67	0	0	0	0	0	0	0	0	1	1	1	0	0	0	0	NA	0	0	0	0
4279	2023	5	M	67	0	1	0	0	0	0	0	0	0	0	1	0	1	0	0	2	0	0	0	0
4280	2024	8	F	67	0	1	0	0	0	0	0	0	0	0	1	0	0	0	1	1	0	0	0	0
4281	2024	6	M	67	0	0	1	1	0	0	0	0	0	0	1	1	0	0	0	2	0	1	0	0
4282	2022	2	F	67	0	0	0	0	1	1	0	0	1	0	1	0	1	0	0	2	0	0	0	0
4283	2021	1	F	67	1	1	1	1	1	0	0	0	0	0	1	1	0	0	0	NA	0	0	0	0
4284	2020	2	F	68	0	0	0	0	0	0	0	0	0	0	0	0	0	0	1	2	1	1	1	1

ID	Yr	Mo	Sex	Age	Tre	Grs	Rag	Mug	HDM	Dan	Mld	Food	Drg	Ven	Rhi	Cnj	Ast	AsE	Urt	Sev	Prt	Obs	Rho	Snz
4285	2020	3	F	68	0	0	0	0	0	0	0	0	0	0	1	0	1	0	0	1	0	0	0	0
4286	2021	9	F	68	0	0	0	0	0	0	0	0	0	0	0	1	0	0	0	2	0	1	0	0
4287	2021	10	M	68	0	0	0	0	0	0	0	0	0	0	0	0	0	0	0	2	0	1	0	0
4288	2021	10	F	68	0	0	0	0	0	0	0	0	0	0	0	0	0	0	1	1	0	0	0	0
4289	2023	4	F	68	0	0	0	0	0	0	0	0	0	0	0	0	0	0	0	1	0	0	0	0
4290	2024	6	F	68	0	0	0	0	0	0	0	0	0	0	0	0	0	0	1	2	0	0	0	0
4291	2024	9	F	68	0	0	0	0	0	0	0	0	0	0	1	0	1	0	0	2	0	0	0	0
4292	2024	11	F	68	0	0	0	0	0	0	0	0	0	0	0	0	0	0	1	NA	0	0	0	0
4293	2021	1	F	68	0	0	0	0	0	0	0	0	1	0	1	0	1	0	0	NA	0	0	0	0
4294	2021	10	M	68	0	0	0	0	0	0	0	0	0	1	0	0	0	0	0	2	0	0	0	0
4295	2023	8	F	68	0	0	0	0	0	0	0	0	1	0	0	0	1	0	1	1	0	0	0	1
4296	2023	12	F	68	0	0	0	0	0	0	0	0	1	0	0	0	0	0	1	NA	0	0	0	0
4297	2022	1	F	68	0	0	0	0	0	0	0	1	1	0	0	0	0	0	0	NA	0	0	0	0
4298	2022	7	F	68	0	0	1	0	0	0	0	0	0	0	1	1	0	0	0	NA	0	0	0	0
4299	2024	8	M	68	0	0	1	0	0	0	0	0	0	0	1	1	0	0	0	1	0	0	1	1
4300	2021	11	F	68	0	1	0	0	1	0	0	0	0	0	1	0	0	0	1	2	0	1	0	0
4301	2023	6	F	68	0	0	0	0	1	1	0	0	0	0	0	0	0	0	1	2	0	0	1	0
4302	2023	6	F	68	0	1	0	1	0	0	1	0	0	0	1	0	1	0	0	1	0	1	1	0
4303	2020	2	F	69	0	0	0	0	0	0	0	0	0	0	0	0	1	0	0	NA	0	1	0	0
4304	2020	9	F	69	0	0	0	0	0	0	0	0	0	0	1	0	0	0	0	2	0	0	0	0
4305	2021	3	F	69	0	0	0	0	0	0	0	0	0	0	0	0	1	0	0	2	1	1	1	0
4306	2021	4	M	69	0	0	0	0	0	0	0	0	0	0	0	0	0	1	0	NA	0	0	0	0
4307	2022	5	M	69	0	0	0	0	0	0	0	0	0	0	0	0	0	0	0	2	0	1	0	0
4308	2023	8	M	69	0	0	0	0	0	0	0	0	0	0	0	0	1	0	0	NA	0	0	0	0
4309	2023	10	F	69	0	0	0	0	0	0	0	0	0	0	0	0	0	0	1	1	1	1	0	1
4310	2023	10	F	69	0	0	0	0	0	0	0	0	0	0	0	0	0	0	1	2	0	0	0	0
4311	2024	4	F	69	0	0	0	0	0	0	0	0	0	0	0	0	0	0	0	NA	0	0	0	0
4312	2024	7	F	69	0	0	0	0	0	0	0	0	0	0	0	0	0	0	1	NA	0	0	0	0
4313	2024	7	F	69	0	0	0	0	0	0	0	0	0	0	0	0	0	0	0	1	0	1	0	0
4314	2024	9	F	69	0	0	0	0	0	0	0	0	0	0	1	0	1	0	0	NA	0	0	0	0
4315	2024	11	F	69	0	0	0	0	0	0	0	0	0	0	0	0	0	0	0	NA	0	0	0	0
4316	2023	10	F	69	0	0	1	0	0	0	0	0	0	0	1	1	0	0	0	1	0	0	0	0
4317	2020	1	F	70	0	0	0	0	0	0	0	0	0	0	1	0	1	0	0	NA	0	0	0	0
4318	2020	1	F	70	0	0	0	0	0	0	0	0	0	0	0	0	0	0	1	NA	0	0	0	0
4319	2020	6	F	70	0	0	0	0	0	0	0	0	0	0	0	0	0	0	1	2	1	1	0	0
4320	2020	9	M	70	0	0	0	0	0	0	0	0	0	0	1	0	1	0	0	2	1	1	1	0
4321	2020	10	F	70	0	0	0	0	0	0	0	0	0	0	0	0	1	0	0	2	0	0	0	0
4322	2021	6	F	70	0	0	0	0	0	0	0	0	0	0	0	0	0	0	0	2	0	0	0	0
4323	2021	8	M	70	0	0	0	0	0	0	0	0	0	0	0	0	0	0	1	1	1	0	0	0
4324	2021	9	F	70	0	0	0	0	0	0	0	0	0	0	0	0	0	1	0	1	0	0	0	0
4325	2021	5	F	70	0	0	0	0	0	0	0	0	0	0	0	0	0	1	0	2	0	1	0	0
4326	2022	2	F	70	0	0	0	0	0	0	0	0	0	0	0	0	0	0	0	2	0	1	0	0
4327	2022	10	F	70	0	0	0	0	0	0	0	0	0	0	0	0	0	0	1	2	0	0	0	0
4328	2023	3	F	70	0	0	0	0	0	0	0	0	0	0	1	0	0	0	0	2	1	1	1	1
4329	2023	5	F	70	0	0	0	0	0	0	0	0	0	0	0	0	0	0	0	2	0	0	0	0
4330	2023	5	F	70	0	0	0	0	0	0	0	0	0	0	1	0	0	0	0	2	0	1	0	0
4331	2023	11	F	70	0	0	0	0	0	0	0	0	0	0	0	0	0	0	1	1	1	0	0	0
4332	2024	2	M	70	0	0	0	0	0	0	0	0	0	0	0	0	0	0	1	NA	0	0	0	0
4333	2024	3	M	70	0	0	0	0	0	0	0	0	0	0	0	0	0	0	1	NA	0	0	0	0
4334	2024	8	F	70	0	0	0	0	0	0	0	0	0	0	0	0	0	0	1	NA	0	0	0	0
4335	2021	5	F	70	0	0	0	0	0	0	0	0	1	0	0	0	0	0	0	NA	0	0	0	0
4336	2020	10	F	70	1	0	0	0	0	0	0	0	0	0	1	0	0	0	0	2	0	1	0	1
4337	2021	8	F	70	0	0	0	0	0	0	1	0	0	0	0	1	0	0	0	NA	0	0	0	0
4338	2020	10	F	71	0	0	0	0	0	0	0	0	0	0	1	0	1	0	0	2	0	0	0	0
4339	2021	2	M	71	0	0	0	0	0	0	0	0	0	0	0	0	0	0	1	2	0	1	1	1
4340	2023	8	F	71	0	0	0	0	0	0	0	0	0	0	1	0	0	0	0	2	1	0	0	0
4341	2024	2	F	71	0	0	0	0	0	0	0	0	0	0	0	0	0	0	0	NA	0	0	0	0
4342	2024	7	F	71	0	0	0	0	0	0	0	0	0	0	1	0	0	0	0	1	0	1	0	0
4343	2024	10	F	71	0	0	0	0	0	0	0	0	0	0	0	0	0	0	1	1	1	0	0	0
4344	2021	5	F	71	0	0	0	0	0	0	0	0	0	1	0	0	0	0	1	NA	0	0	0	0
4345	2020	7	M	71	0	0	0	1	0	0	0	0	0	0	1	1	0	0	0	NA	0	0	0	0
4346	2021	2	F	71	0	1	0	0	1	0	0	0	0	0	1	0	0	0	0	1	1	0	0	0
4347	2020	1	F	72	0	0	0	0	0	0	0	0	0	0	1	0	1	0	0	1	1	0	0	0

ID	Yr	Mo	Sex	Age	Tre	Grs	Rag	Mug	HDM	Dan	Mld	Food	Drg	Ven	Rhi	Cnj	Ast	AsE	Urt	Sev	Prt	Obs	Rho	Snz
4348	2022	1	F	72	0	0	0	0	0	0	0	0	0	0	0	0	0	0	0	2	0	1	0	1
4349	2022	9	F	72	0	0	0	0	0	0	0	0	0	0	0	1	1	0	0	2	0	1	0	0
4350	2023	6	F	72	0	0	0	0	0	0	0	0	0	0	0	0	0	0	1	NA	0	0	0	0
4351	2024	3	F	72	0	0	0	0	0	0	0	0	0	0	1	0	1	0	0	1	1	0	0	0
4352	2024	5	F	72	0	0	0	0	0	0	0	0	0	0	0	0	1	0	1	1	1	0	0	0
4353	2022	5	M	73	0	0	0	0	0	0	0	0	0	0	0	0	1	0	1	2	0	1	1	0
4354	2022	5	F	73	0	0	0	0	0	0	0	0	0	0	0	0	0	0	0	NA	0	0	0	0
4355	2023	9	F	73	0	0	0	0	0	0	0	0	0	0	1	0	1	0	0	NA	0	0	0	0
4356	2024	11	F	73	0	0	0	0	0	0	0	0	0	0	1	0	1	0	0	1	1	0	0	0
4357	2024	12	F	73	0	0	0	0	0	0	0	0	0	0	0	0	0	0	0	1	0	0	1	0
4358	2023	8	M	73	0	0	0	0	0	0	0	0	1	0	0	0	0	0	1	NA	0	0	0	0
4359	2020	9	F	73	0	0	0	0	1	0	0	0	0	0	1	0	1	0	0	NA	0	0	0	0
4360	2020	3	F	74	0	0	0	0	0	0	0	0	0	0	0	0	1	0	0	2	1	1	0	1
4361	2020	11	M	74	0	0	0	0	0	0	0	0	0	0	0	0	0	0	1	1	1	0	0	0
4362	2021	9	M	74	0	0	0	0	0	0	0	0	0	0	0	0	0	1	0	2	0	1	0	0
4363	2022	11	F	74	0	0	0	0	0	0	0	0	0	0	0	0	0	0	1	NA	0	0	0	0
4364	2021	1	F	74	0	0	0	0	0	1	0	0	0	0	1	0	0	0	0	NA	0	0	0	0
4365	2022	2	F	74	0	0	0	0	1	0	0	0	0	0	1	0	1	0	1	1	1	0	0	0
4366	2022	8	F	74	0	1	0	0	0	0	0	0	0	0	1	0	1	0	0	NA	0	0	0	0
4367	2023	4	M	74	0	1	0	0	0	0	0	0	0	0	1	0	0	0	0	1	1	1	1	1
4368	2021	8	F	75	0	0	0	0	0	0	0	0	0	0	0	0	0	0	0	2	1	0	0	0
4369	2021	2	F	75	0	0	0	0	0	0	0	0	0	0	0	0	1	0	1	1	1	0	0	0
4370	2022	3	F	75	0	0	0	0	0	0	0	0	0	0	0	0	0	0	0	2	1	0	0	0
4371	2022	9	F	75	0	0	0	0	0	0	0	0	0	0	0	0	1	0	0	1	0	1	0	0
4372	2021	4	F	75	0	0	0	0	0	0	0	0	1	0	0	0	0	0	0	NA	0	0	0	0
4373	2022	8	M	75	0	0	1	0	0	0	0	0	0	0	1	1	1	1	0	1	0	1	0	0
4374	2023	5	F	75	0	1	0	0	0	0	0	0	0	0	1	0	0	0	0	NA	0	0	0	0
4375	2021	5	F	76	0	0	0	0	0	0	0	0	0	0	0	0	0	1	0	1	0	0	1	1
4376	2022	3	F	76	0	0	0	0	0	0	0	0	0	0	1	0	1	0	0	1	0	1	0	0
4377	2023	3	M	76	0	0	0	0	0	0	0	1	0	0	1	0	0	0	0	2	1	1	0	0
4378	2021	7	F	77	0	0	0	0	0	0	0	0	0	0	0	0	0	0	0	1	0	1	0	0
4379	2022	3	M	77	0	0	0	0	0	0	0	0	0	0	0	0	0	0	1	2	0	0	1	1
4380	2023	11	F	77	0	0	0	0	0	0	0	0	0	0	0	0	0	0	1	2	1	1	1	1
4381	2023	11	F	77	0	1	1	0	0	0	0	0	0	0	1	0	0	0	1	1	0	1	0	0
4382	2022	1	F	78	0	0	0	0	0	0	0	0	0	0	0	0	0	0	0	NA	0	0	0	0
4383	2023	4	F	78	0	0	0	0	0	0	0	0	0	0	1	0	0	0	0	NA	0	0	0	0
4384	2020	1	F	79	0	0	0	0	0	0	0	0	0	0	1	0	1	0	0	1	1	1	1	1
4385	2020	7	F	79	0	0	0	0	0	0	0	0	0	0	0	0	0	0	0	NA	0	0	0	0
4386	2022	6	F	79	1	0	0	0	0	0	0	0	0	0	1	0	1	0	0	NA	0	0	0	0
4387	2022	7	F	80	0	0	0	0	0	0	0	0	0	0	0	0	0	0	1	NA	0	0	0	0
4388	2022	5	F	81	0	0	0	0	0	0	0	0	0	0	0	0	0	0	1	NA	0	0	0	0
4389	2024	10	F	81	0	0	0	0	0	0	0	0	0	0	0	0	1	0	0	NA	0	0	0	0
4390	2024	1	F	82	0	0	0	0	0	0	0	0	0	0	0	0	0	0	1	NA	0	0	0	0
4391	2023	3	M	83	0	0	0	0	0	0	0	0	1	0	0	0	0	0	1	1	0	1	0	0
4392	2022	1	M	84	0	0	0	0	0	0	0	0	0	0	1	0	0	0	0	NA	0	0	0	0
4393	2021	10	F	85	0	0	0	0	0	0	0	0	0	0	0	0	0	0	1	1	0	0	0	1
4394	2023	6	F	85	0	0	0	0	0	0	0	0	0	0	0	0	0	0	1	1	0	1	0	1
4395	2020	2	F	85	0	0	0	0	0	0	0	0	1	0	0	0	0	0	1	1	0	1	0	0
4396	2022	12	F	86	0	0	0	0	0	0	0	0	0	0	0	0	0	0	0	NA	0	0	0	0
4397	2021	12	F	86	0	0	0	0	0	0	0	0	0	1	0	0	0	1	0	1	0	0	0	0
4398	2020	6	F	86	0	1	0	0	0	1	0	0	0	0	1	0	0	0	1	1	1	0	0	0
4399	2021	2	F	87	0	0	0	0	0	0	0	0	0	0	1	0	1	0	0	2	0	0	1	1
4400	2021	11	M	87	0	0	0	0	0	0	0	0	0	0	0	0	0	0	0	2	1	1	1	1
4401	2022	5	F	90	0	0	0	0	0	0	0	0	1	0	0	0	0	0	1	NA	0	0	0	0
4402	2021	4	F	90	0	0	1	0	1	0	0	0	0	0	1	0	0	0	0	1	0	1	0	1
4403	2022	1	M	0	0	0	0	0	0	0	0	0	0	0	0	0	0	0	1	NA	0	0	0	0
4404	2020	9	F	30	0	0	0	0	0	0	0	0	0	0	0	0	0	0	0	NA	0	0	0	0

Table S2. De-identified individual-level responses of the population survey (n = 500), with codebook

De-identified individual-level responses of all 500 participants in the cross-sectional survey on food allergy knowledge, attitudes and behaviours (Study 2).
One row per respondent; response options are coded — see the codebook on the next page.
These data underlie every Study 2 statistic reported in the article.

Codebook

```
ID   respondent number   |  Age  years  |  Sex M/F/N (N = preferred not to say)  |  Res U = urban, R = rural
Edu  education: 1 lower secondary, 2 upper secondary, 3 post-secondary, 4 university, 5 postgraduate
Med  medical/health training: 1 yes, 0 no
Q6   self-reported food allergy: Y yes, N no, U unsure
Q7   basis of reported allergy: T allergy testing, S physician diagnosis on symptoms only, A own assessment, - not applicable
Q8   foods incriminated (multi): L milk, O egg, P peanut, N tree nuts, F fish, S shellfish, G wheat/gluten,
     Y soy, V fruit/vegetables, - none  (lower-case letters = free-text 'other')
Q9   family history of diagnosed food allergy: Y/N/U
Q10-Q17 (true/false items): T true, F false, U do not know.  Correct answers: Q10 F, Q11 T, Q13 T, Q14 F, Q15 F, Q17 T
Q12  allergens subject to mandatory labelling (multi): same food codes; z sugar, w water (distractors).
     Correct = exactly {L,O,P,G,S,Y} with no distractor
Q16  manifestations of an allergic reaction (multi): U urticaria/itching, S facial/lip swelling, B breathing difficulty,
     H hypotension/fainting, d headache without other symptoms (distractor). Correct = exactly {U,S,B,H}
Q18  first-line drug in anaphylaxis: A adrenaline (correct), H oral antihistamine, C corticosteroid tablets, U do not know
Q19  adrenaline auto-injector: K knows how to use, H heard of it only, N never heard of it
Q20  mild reaction: H antihistamine, E emergency department, C call 112, W wait, U do not know
Q21  severe reaction: C call 112/use auto-injector (correct), H antihistamine and stay home, W wait, U do not know
Q22  reads allergen labels: A always, S sometimes, I only if an allergy is present, N never
Q23  confidence in recognizing a severe reaction: 0 not at all - 3 very confident
Q24  physician consulted first: F family physician, A allergist, E emergency, G gastroenterologist, N none
Q25  main information source: D doctor, I internet/social media, P family/friends, T press/TV, Ph pharmacist, N none
Q26  feels knowledge sufficient: Y yes, P partly, N no    |    Q27  interest in education programmes: Y/N/U

Knowledge score = one point for each correct answer to Q10-Q18 (excluding Q19) and Q21; maximum 10.
Responses were collected anonymously; the dataset contains no identifying information.
```

ID	Age	Sex	Res	Edu	Med	Q6	Q7	Q8	Q9	Q10	Q11	Q12	Q13	Q14	Q15	Q16	Q17	Q18	Q19	Q20	Q21	Q22	Q23	Q24	Q25	Q26	Q27
1	33	M	R	4	0	N	-	-	N	F	T	PSYz	T	F	T	BHSU	U	H	H	H	C	N	1	F	Ph	P	Y
2	22	M	R	2	0	N	-	-	U	T	U	O	U	T	U	U	T	U	N	C	H	S	2	E	D	Y	U
3	83	M	U	2	0	N	-	-	N	T	F	GLO	T	U	U	BHS	T	C	N	C	C	S	3	A	D	N	Y
4	23	M	R	4	0	N	-	-	Y	F	T	GPS	T	F	T	BHSU	T	H	H	H	C	A	3	G	I	N	Y
5	51	M	U	2	0	N	-	-	Y	T	T	GOSz	T	U	F	BSU	T	A	K	U	W	A	2	F	I	P	Y
6	19	F	R	4	0	N	-	-	U	U	T	GLOPS	T	F	F	BHSU	T	H	N	H	C	I	2	F	I	Y	Y
7	69	F	U	2	1	Y	S	L	N	T	T	GLPY	T	F	U	BHS	T	A	N	H	C	A	2	A	N	Y	Y
8	31	F	U	3	0	Y	S	S	N	T	U	GLPS	T	T	U	-	T	H	K	H	H	S	1	A	D	P	Y
9	70	M	R	3	0	N	-	-	Y	F	F	GLP	T	T	F	BHSU	T	U	K	H	C	S	0	F	T	Y	Y
10	51	F	R	3	0	N	-	-	N	F	T	GLOPw	T	U	F	BHSU	T	A	H	H	C	S	2	A	N	P	Y
11	82	M	U	4	0	N	-	-	N	F	U	GLOP	U	F	F	BHSU	F	H	H	H	W	N	1	F	I	P	Y
12	24	M	R	4	0	N	-	-	N	F	T	GLSY	T	T	T	BHSUd	T	A	H	C	C	N	2	F	D	P	Y
13	44	M	R	1	0	N	-	-	N	T	F	LOPS	F	T	T	SU	U	U	N	E	H	N	0	N	P	N	U
14	52	F	U	2	0	N	-	-	N	F	U	GLOPSY	T	F	T	BHS	T	C	K	H	H	S	1	A	D	P	Y
15	57	F	R	3	1	Y	S	P	N	F	T	GLOPS	T	F	F	BSU	T	A	K	H	C	I	2	F	D	Y	N
16	73	M	U	5	0	Y	T	LNY	N	U	T	LOPY	T	T	F	BHU	T	A	N	H	C	A	2	F	P	Y	U
17	51	M	R	4	0	N	-	-	Y	F	T	GLOS	T	T	F	BHS	T	A	K	H	C	A	3	A	I	P	Y
18	25	F	R	2	0	N	-	-	N	T	T	GOP	U	T	T	BHU	F	C	N	H	H	S	0	N	I	N	Y
19	24	M	R	5	1	Y	S	FNV	Y	F	T	GLOPSY	T	F	F	BHSU	T	A	K	U	C	I	2	A	P	P	Y
20	46	F	R	1	0	N	-	-	N	U	T	GOP	F	T	F	BS	T	U	N	H	C	N	2	G	D	P	U
21	35	F	U	2	0	N	-	-	U	T	T	GOPS	T	F	T	BH	T	A	H	H	C	N	0	A	I	P	N
22	37	F	U	4	0	Y	S	OS	N	U	T	GLPY	T	F	F	SU	T	U	K	H	C	S	2	A	I	Y	Y
23	18	F	U	3	0	N	-	-	U	F	T	LO	F	U	F	BSU	U	U	K	H	U	A	1	F	I	Y	Y
24	26	F	U	2	0	U	-	V	Y	F	F	GLOP	T	F	F	BU	T	H	K	H	C	A	2	A	P	P	U
25	30	F	U	3	0	Y	S	L	N	F	T	GLOPS	U	F	F	BSU	T	C	H	H	C	I	2	A	D	Y	N
26	86	M	U	3	0	N	-	-	N	T	T	GLPSY	U	F	T	BSUd	T	A	N	H	W	S	1	F	Ph	Y	Y
27	36	M	U	4	1	N	-	-	N	F	T	GLOPS	T	F	F	BHSU	T	A	K	U	C	I	3	A	Ph	Y	Y
28	45	M	U	3	0	U	-	V	N	F	T	GLOY	T	U	F	BSU	T	A	K	C	C	S	0	A	I	P	Y
29	55	F	R	1	0	N	-	-	U	T	U	GOPS	T	T	T	H	U	H	K	H	W	N	1	A	N	P	Y
30	60	F	R	3	0	N	-	-	N	U	U	L	F	T	F	BSU	U	A	N	E	U	S	1	F	D	Y	U
31	59	F	U	4	0	N	-	-	Y	T	T	GOPSz	F	F	F	BHSUd	T	C	N	H	C	A	2	N	I	P	Y
32	21	M	U	2	0	N	-	-	N	F	T	LOPSY	T	U	T	HSU	T	C	K	H	H	S	1	A	I	Y	Y
33	34	M	U	4	0	N	-	-	U	U	T	GLOS	U	T	F	BU	T	A	N	H	H	N	2	A	D	Y	Y
34	85	N	R	2	0	U	-	N	U	T	F	LOPY	F	U	T	BHU	U	U	K	W	W	N	1	F	P	P	N
35	32	F	U	3	1	N	-	-	N	F	T	GLOPSY	T	F	F	BHSU	T	A	K	H	C	I	3	F	Ph	Y	Y
36	62	F	R	1	0	N	-	-	N	T	F	GSYwz	T	T	F	SUd	T	C	H	H	W	S	3	A	T	N	Y
37	48	F	U	2	0	N	-	-	U	U	U	GLSY	F	F	T	BSU	T	U	N	H	W	S	2	N	T	Y	Y
38	35	M	U	2	0	U	-	N	Y	F	T	GLOPY	T	F	F	BU	T	A	H	H	C	I	1	A	I	Y	U
39	31	F	U	4	0	N	-	-	N	F	U	GLOPSYw	T	T	F	BU	T	A	K	H	C	N	1	A	I	Y	Y
40	42	F	R	2	0	N	-	-	Y	U	T	LOS	U	T	U	BHUd	U	U	K	W	C	A	1	N	D	N	Y
41	66	F	U	5	0	N	-	-	N	F	T	GLOPSYz	T	F	F	HSU	T	A	N	H	C	I	2	F	P	Y	N
42	65	F	U	4	0	N	-	-	Y	F	T	GLPY	T	T	F	BSUd	T	U	K	W	C	A	2	F	N	N	Y
43	64	M	U	2	0	N	-	-	N	U	T	GS	T	F	F	BHSU	T	A	N	H	C	I	3	F	D	P	Y
44	54	F	R	2	0	N	-	-	N	T	T	GLOS	F	F	T	BU	T	H	K	C	U	S	3	F	I	P	Y
45	63	M	R	1	1	N	-	-	U	F	T	PS	T	T	F	S	T	A	K	E	C	N	1	F	P	P	Y
46	24	F	R	4	0	N	-	-	N	F	U	GLOP	T	F	F	HSU	T	H	H	H	C	S	0	F	I	N	N
47	61	F	U	4	0	N	-	-	N	F	U	LOPSz	T	T	U	BHSU	T	U	N	H	C	A	0	E	P	P	U
48	33	F	U	5	0	N	-	-	N	T	T	GLOPSY	T	F	F	BS	U	U	H	H	C	S	2	E	D	Y	Y
49	59	F	U	5	0	N	-	-	Y	F	T	LOPSY	U	F	F	BHSU	T	H	N	H	C	A	3	F	D	N	Y
50	71	F	R	2	0	N	-	-	N	T	T	GLOS	F	F	T	HU	U	H	K	W	H	S	0	F	I	P	Y
51	22	F	U	3	0	Y	A	O	U	U	T	LOPS	T	F	F	HU	T	A	K	H	C	N	2	F	N	P	Y
52	51	F	R	1	0	N	-	-	N	U	F	L	F	F	F	BHSU	T	H	H	C	W	S	2	A	I	P	Y
53	57	F	R	4	0	Y	A	N	Y	T	T	GLOPS	T	F	F	BU	U	A	H	H	C	S	2	A	N	P	Y
54	35	F	U	2	0	U	-	L	N	T	T	LOPSz	T	F	F	HU	U	A	N	C	C	S	2	F	I	Y	Y
55	59	M	R	5	0	Y	T	L	N	F	T	GLPS	T	F	F	BSU	T	A	K	H	H	N	0	F	Ph	Y	N
56	64	M	R	2	0	N	-	-	Y	F	F	GLOz	T	U	U	SU	U	A	K	H	H	S	2	F	P	Y	Y
57	52	M	R	3	0	Y	A	V	N	T	T	GLOPSY	U	F	F	SU	T	H	N	H	C	S	1	F	D	N	Y
58	50	F	R	1	0	N	-	-	N	T	T	GLOPSYwz	U	U	U	BHSUd	F	H	N	E	C	S	2	G	Ph	P	Y
59	31	F	R	2	0	N	-	-	N	F	T	GOS	F	F	T	BSU	T	A	K	H	C	N	1	F	D	Y	U
60	23	M	U	2	0	N	-	-	Y	T	T	GLOPS	F	F	U	BH	F	H	K	H	C	S	1	F	I	Y	Y
61	26	M	U	3	0	Y	A	N	N	F	T	LOSY	T	F	F	HSU	F	A	K	E	C	I	2	F	I	P	U

ID	Age	Sex	Res	Edu	Med	Q6	Q7	Q8	Q9	Q10	Q11	Q12	Q13	Q14	Q15	Q16	Q17	Q18	Q19	Q20	Q21	Q22	Q23	Q24	Q25	Q26	Q27
62	66	M	U	4	0	N	-	-	N	F	U	GOS	T	F	F	BSU	T	U	N	U	C	N	2	E	D	P	N
63	60	F	U	4	0	N	-	-	Y	F	T	GLOPSY	T	F	F	SU	T	A	K	E	C	I	3	A	I	N	U
64	21	F	U	2	0	N	-	-	N	T	F	LPSz	F	T	T	BSU	T	A	H	E	H	I	1	A	P	P	Y
65	22	F	U	4	0	Y	A	VY	U	T	T	GLOSY	T	T	F	BSUd	T	A	H	H	C	A	1	A	I	P	N
66	54	M	U	2	0	N	-	-	N	U	U	GOPSY	T	U	F	HSU	U	H	K	H	W	I	1	A	T	Y	N
67	60	M	U	1	0	N	-	-	N	U	T	OY	F	U	T	BHSU	U	H	N	W	H	N	1	G	D	N	Y
68	25	M	U	2	0	U	-	G	N	F	U	LS	T	F	T	HS	T	H	N	H	C	N	2	A	I	P	Y
69	50	M	R	4	0	N	-	-	N	T	T	LOPSz	T	F	U	BHSU	T	A	N	C	C	S	1	F	D	P	U
70	25	F	U	2	0	Y	T	FNOV	N	F	F	LOPY	U	F	F	BHS	T	H	K	H	C	I	2	A	I	P	U
71	46	M	U	2	0	N	-	-	U	U	U	LPS	U	U	T	BU	U	H	K	H	C	N	1	F	I	P	U
72	56	M	U	4	0	N	-	-	N	T	T	GLOPSz	T	U	U	BHS	T	C	K	H	H	A	3	E	D	P	Y
73	64	F	R	4	0	Y	T	N	Y	F	T	GLOPSw	T	U	F	BSU	T	A	K	W	C	S	1	F	Ph	Y	Y
74	74	F	U	2	0	N	-	-	N	F	F	GLPSY	T	F	T	SU	T	H	H	E	H	S	1	F	T	P	Y
75	45	F	U	4	0	N	-	-	U	U	U	GLOPS	T	F	U	BHSU	T	A	K	H	C	N	3	F	N	Y	U
76	29	F	R	3	0	N	-	-	N	T	U	LOP	T	T	F	BHU	T	U	H	H	C	S	1	A	I	P	Y
77	22	F	U	2	0	N	-	-	N	T	F	GPS	T	F	U	BHU	U	A	K	H	C	S	3	A	I	Y	Y
78	21	M	U	2	0	N	-	-	N	F	T	OPS	U	T	F	BSU	T	H	H	H	H	S	3	E	D	N	U
79	26	F	U	2	0	N	-	-	Y	U	F	GLPS	U	F	F	BU	T	A	K	W	U	I	2	A	I	P	Y
80	82	F	R	2	0	N	-	-	N	T	T	GLOY	T	T	F	BHU	T	A	N	W	H	N	0	F	I	P	Y
81	66	F	R	2	0	N	-	-	Y	F	T	GO	T	F	U	BSU	U	A	H	H	H	A	2	G	T	N	Y
82	49	F	R	1	0	Y	T	Y	N	F	T	GLOS	F	T	T	BSU	U	C	K	H	C	I	1	A	D	P	U
83	86	F	R	4	0	Y	T	L	N	F	T	GLOS	T	U	U	HSU	T	A	H	H	U	S	0	F	Ph	P	N
84	58	M	U	3	0	N	-	-	U	T	T	GLOPSY	T	F	F	BU	F	H	K	U	H	N	3	F	I	P	N
85	38	M	R	4	0	N	-	-	Y	F	T	GLOPSY	T	F	F	BHSU	T	H	H	U	C	S	1	F	I	P	N
86	38	F	U	5	0	N	-	-	N	F	T	GLOPSY	T	U	F	BU	T	A	N	H	C	S	2	A	N	P	Y
87	20	F	U	4	0	N	-	-	N	T	T	LPYz	T	F	F	BSU	T	A	H	C	W	I	3	A	D	P	N
88	54	M	R	2	0	N	-	-	U	T	U	GLPY	T	F	T	BHSU	T	C	K	W	U	I	1	A	D	Y	Y
89	49	F	U	3	0	N	-	-	N	T	U	OPS	F	F	T	HSU	T	C	K	E	C	I	2	E	P	P	Y
90	70	F	R	1	0	N	-	-	N	F	U	LO	U	T	T	SUd	T	C	H	E	H	S	3	N	T	P	Y
91	20	F	R	2	0	N	-	-	N	F	U	GLOPY	U	T	F	SU	F	A	H	E	C	N	1	A	Ph	Y	Y
92	26	F	U	5	1	N	-	-	N	T	T	GLPSY	T	F	F	BHSU	T	A	K	H	C	S	2	F	P	N	Y
93	27	M	U	3	0	N	-	-	N	F	U	GLOPS	U	T	T	BSU	F	A	K	H	C	N	0	F	I	P	N
94	30	F	R	2	0	N	-	-	N	T	F	G	U	F	F	BHSU	T	U	N	E	C	N	3	A	I	N	Y
95	47	F	U	3	0	N	-	-	N	T	T	GLOS	U	T	F	HS	T	U	N	W	C	N	3	G	I	P	U
96	49	F	U	1	0	N	-	-	N	F	T	GLOS	U	F	F	BU	U	C	H	W	U	N	1	F	I	N	N
97	49	F	R	2	0	N	-	-	N	F	T	OSY	F	F	U	BHSU	T	A	K	H	H	S	1	A	T	P	U
98	36	M	R	4	0	U	-	O	U	F	T	GOPSY	T	T	F	BSU	T	A	N	E	C	S	2	A	I	P	Y
99	50	F	R	2	0	N	-	-	U	T	T	G	U	F	F	SUd	T	U	N	H	W	I	3	A	I	N	Y
100	48	F	U	4	0	N	-	-	U	F	T	LOPSY	T	T	F	BSU	T	A	N	H	C	S	2	E	P	P	Y
101	62	M	U	4	0	Y	T	LNOPV	N	F	T	GLOPY	F	F	F	BHSU	T	A	K	H	C	I	1	F	D	N	Y
102	83	M	U	1	0	Y	T	O	Y	F	T	LOPSY	F	T	T	B	U	U	N	H	C	S	1	A	P	P	N
103	30	F	U	4	0	N	-	-	Y	F	F	GLOPSY	U	T	F	HUd	T	C	K	W	C	S	1	A	N	P	N
104	58	F	U	5	0	N	-	-	N	T	F	GLPSw	T	F	F	BSU	T	A	K	E	H	S	2	A	T	P	Y
105	25	F	U	3	0	N	-	-	N	F	T	GLOPS	U	F	F	BU	T	A	N	E	C	N	3	F	I	P	Y
106	45	F	R	2	0	N	-	-	N	T	T	LPY	U	T	U	BHU	T	U	H	E	H	A	2	F	N	P	U
107	29	M	R	4	1	N	-	-	N	F	T	GOPSY	T	F	F	BHU	T	A	K	H	C	N	2	F	P	Y	Y
108	69	M	U	4	1	U	-	V	Y	F	T	GLOPS	T	F	F	SU	T	A	K	U	C	I	3	F	I	Y	U
109	20	M	R	2	0	U	-	Y	Y	F	F	GLz	F	U	U	BHUd	U	H	H	H	C	I	1	F	I	P	Y
110	33	M	U	2	0	N	-	-	U	T	F	LO	U	T	F	BHU	T	U	N	H	C	S	1	F	D	P	N
111	36	F	U	3	0	N	-	-	N	F	F	GLOPSw	T	U	F	S	T	A	N	H	W	I	2	A	I	P	N
112	37	F	U	5	1	U	-	L	U	F	T	GLOP	T	F	F	SU	T	A	H	H	C	N	2	F	I	P	Y
113	50	F	U	2	1	N	-	-	Y	F	T	GOPS	T	F	F	BSU	T	A	K	H	C	N	3	A	Ph	Y	Y
114	27	F	U	5	0	N	-	-	U	F	T	GLOPSY	T	F	F	BSU	T	C	K	H	C	S	2	A	I	N	Y
115	39	M	R	2	0	N	-	-	U	F	T	GOz	F	F	F	BSU	U	A	H	H	C	N	3	A	I	P	U
116	21	M	U	4	0	N	-	-	N	U	F	GPSY	U	F	F	U	T	H	K	H	H	N	3	F	I	P	Y
117	59	F	U	5	0	U	-	Y	Y	U	T	GLOY	U	F	F	BHSU	F	A	K	H	C	S	1	F	D	P	Y
118	29	M	U	5	0	N	-	-	N	F	T	GLOPS	T	U	F	SU	T	U	N	H	C	S	1	A	Ph	Y	Y
119	34	M	R	2	0	N	-	-	N	T	F	SY	F	T	U	BSU	T	A	N	H	C	S	2	F	N	N	Y
120	70	M	U	2	0	N	-	-	U	F	U	GLPS	T	F	F	S	F	U	N	W	H	I	2	F	I	P	Y
121	22	M	R	2	0	N	-	-	N	F	F	GLOPw	F	F	F	SU	F	H	N	U	W	N	2	E	P	Y	Y
122	71	M	U	4	0	N	-	-	N	F	T	GLOPSY	T	T	U	BHS	T	H	H	W	U	A	3	A	P	P	Y

ID	Age	Sex	Res	Edu	Med	Q6	Q7	Q8	Q9	Q10	Q11	Q12	Q13	Q14	Q15	Q16	Q17	Q18	Q19	Q20	Q21	Q22	Q23	Q24	Q25	Q26	Q27
123	39	M	U	4	0	N	-	-	N	F	F	GLOPSY	T	F	F	BHSU	U	A	N	U	H	S	2	A	I	N	U
124	32	F	U	4	0	N	-	-	U	F	F	GOPS	T	F	F	BHSU	T	A	N	H	C	N	1	G	P	P	U
125	33	F	U	3	0	N	-	-	N	F	F	GLOPSY	U	T	F	BHSU	T	A	N	E	H	S	2	A	I	Y	U
126	74	M	R	1	0	U	-	NS	N	T	F	GOPS	U	T	U	S	F	C	H	H	W	S	1	F	I	P	N
127	44	F	R	1	0	Y	T	L	N	U	T	GLOPS	U	U	F	BHUd	U	H	K	H	C	A	3	E	T	N	Y
128	74	F	R	4	0	N	-	-	N	F	T	LOPY	T	F	F	BSU	T	C	N	H	C	A	1	F	I	Y	U
129	62	F	U	4	1	N	-	-	N	F	T	GLOPYz	T	F	T	HSU	U	A	N	U	C	I	3	A	D	P	Y
130	76	F	R	5	1	N	-	-	Y	F	T	GLOPSY	T	T	F	BSU	T	A	K	W	C	S	1	A	N	Y	N
131	32	F	U	3	1	N	-	-	N	F	T	GLOPSz	T	F	F	BHSU	T	A	K	H	C	I	2	E	Ph	N	N
132	26	F	R	3	0	N	-	-	N	T	T	GS	T	T	U	BSU	T	U	K	E	C	S	3	A	D	P	Y
133	34	F	R	4	0	N	-	-	N	T	T	GLOPY	F	F	T	BHSU	U	U	N	H	C	S	1	A	D	P	Y
134	19	M	U	2	0	Y	S	VY	U	F	T	GLP	T	T	F	BHSU	U	A	H	E	C	I	2	F	D	N	Y
135	34	F	U	2	0	U	-	LN	Y	U	U	GLOP	T	F	F	BU	F	C	H	C	U	A	1	F	T	N	N
136	49	F	U	2	0	Y	S	F	N	U	T	LOY	T	F	F	U	T	A	H	H	C	A	0	F	T	Y	N
137	44	F	U	4	0	Y	S	OS	Y	F	T	GLOPS	T	F	F	BSU	T	A	K	H	C	I	1	A	I	P	N
138	46	F	U	2	0	U	-	OS	N	F	T	PS	F	F	T	S	T	H	H	C	H	N	1	G	D	N	N
139	84	F	U	1	0	N	-	-	N	T	F	LOPSY	T	T	T	BHSU	T	H	H	H	W	A	1	N	D	P	Y
140	39	F	U	4	0	N	-	-	N	F	T	GLO	T	F	F	BHS	T	U	H	U	C	I	2	F	D	Y	U
141	59	M	R	2	1	N	-	-	N	F	T	GLOPS	T	F	U	BHSU	T	A	K	W	C	A	1	F	D	N	Y
142	68	M	R	2	0	N	-	-	N	F	T	OS	T	F	T	S	U	H	K	U	W	S	2	F	D	N	N
143	25	F	U	4	1	U	-	V	N	F	T	LPz	F	F	F	BHSU	T	A	H	E	C	N	3	E	I	P	Y
144	23	F	U	2	0	N	-	-	N	F	T	GLOPSz	T	T	U	BSU	T	U	H	H	H	I	2	A	D	N	Y
145	19	M	U	4	0	N	-	-	N	F	T	GLOPY	T	F	F	BSU	U	A	N	E	C	S	3	F	N	Y	Y
146	45	M	U	2	0	Y	T	L	N	F	T	LPSz	U	F	U	BS	T	U	K	C	W	A	1	F	I	Y	Y
147	61	F	U	4	0	N	-	-	Y	T	T	OPSY	F	F	U	BHSU	U	H	H	C	W	N	2	F	D	Y	Y
148	34	M	R	2	0	N	-	-	N	F	U	LPS	U	F	U	BS	U	U	H	H	C	N	1	A	I	Y	Y
149	46	F	U	2	0	N	-	-	N	T	T	LPS	T	F	F	U	T	A	N	C	C	I	1	F	Ph	P	Y
150	73	M	U	4	0	N	-	-	N	U	T	GLSz	T	T	F	Bd	T	U	N	H	C	A	1	E	D	N	Y
151	49	F	U	4	1	N	-	-	N	F	T	GLOPSY	T	T	F	BHSUd	T	A	N	H	C	S	2	F	N	Y	Y
152	35	M	R	2	0	Y	A	L	U	F	F	GLOSY	T	U	F	BHSU	U	H	N	H	C	A	1	E	I	Y	Y
153	46	F	U	4	0	N	-	-	N	F	T	GLOS	U	U	F	BSU	T	U	H	C	C	N	2	G	N	P	Y
154	50	M	U	1	0	N	-	-	N	U	F	OPY	U	U	F	BSU	U	H	H	E	W	N	1	F	I	Y	U
155	38	F	R	2	0	N	-	-	Y	T	U	LPY	T	F	T	U	F	A	H	H	H	I	1	F	P	Y	Y
156	39	M	R	2	1	N	-	-	N	F	T	LOPSwz	T	F	F	BU	T	A	K	W	C	S	3	A	Ph	Y	Y
157	52	M	U	2	0	N	-	-	N	T	T	GLOPSY	T	U	F	U	T	H	K	C	H	S	0	F	P	P	Y
158	23	F	U	4	0	N	-	-	N	T	U	GLPSY	T	F	F	BHSU	T	A	N	H	C	S	2	F	I	Y	Y
159	63	F	R	4	0	N	-	-	N	T	F	GLOPS	T	F	F	BHd	T	H	H	H	C	S	1	F	I	N	Y
160	29	M	R	2	0	N	-	-	U	F	F	GOPSYz	U	U	U	BSU	T	U	N	H	W	I	2	F	P	Y	Y
161	27	F	R	4	0	N	-	-	N	F	T	GOPSY	F	F	T	BUd	T	C	N	U	C	N	1	A	P	P	Y
162	43	F	U	2	0	Y	T	OV	N	F	T	OSz	T	T	F	BHUd	T	H	H	H	W	S	3	F	Ph	Y	Y
163	58	F	R	2	0	N	-	-	N	T	T	PS	F	T	U	B	U	U	N	U	U	S	2	F	I	P	Y
164	50	M	U	4	0	Y	T	F	U	F	T	GLPz	T	F	U	BSU	T	U	K	C	W	I	0	F	D	P	Y
165	68	F	U	4	0	N	-	-	N	F	T	GOPY	T	F	F	BHSU	U	A	N	H	C	S	1	F	I	P	Y
166	44	F	U	4	0	Y	A	FLN	N	T	U	GLOPSYz	T	T	F	BHSU	T	H	N	H	C	I	1	E	I	N	Y
167	60	M	U	5	0	N	-	-	N	F	T	GLOP	T	F	F	BHSU	T	A	N	U	C	S	1	F	I	N	Y
168	27	F	U	3	0	N	-	-	N	T	F	GLPS	T	F	T	-	T	H	H	U	C	A	2	F	I	P	U
169	50	M	R	3	1	N	-	-	N	U	T	GLOPS	T	T	F	BSU	T	A	H	C	C	N	2	A	D	Y	Y
170	31	F	U	4	0	N	-	-	N	T	T	GLOPSYw	U	F	F	HS	T	H	K	H	C	S	3	F	I	P	Y
171	34	N	U	4	0	N	-	-	N	F	T	LS	T	F	F	BS	U	H	N	H	C	I	1	A	I	N	Y
172	30	M	R	1	0	N	-	-	U	F	T	GLOSYz	U	T	T	BS	U	C	N	H	C	S	2	F	I	N	Y
173	54	F	U	2	0	U	-	L	U	F	F	GLPSY	F	F	U	B	T	A	N	E	W	S	2	A	T	P	Y
174	60	M	R	1	0	Y	S	VY	Y	U	T	LOS	T	U	F	HU	U	C	H	E	W	S	1	F	D	P	Y
175	74	M	U	2	0	N	-	-	N	F	F	GLOPS	T	T	T	BHS	T	U	H	W	C	A	0	F	T	P	Y
176	35	M	U	2	0	Y	S	FV	Y	F	T	GLOPSz	T	F	T	BSU	T	U	K	H	W	I	2	F	D	P	Y
177	53	F	R	2	0	N	-	-	Y	T	T	LPS	U	F	U	BHU	T	A	K	H	H	I	1	A	I	P	Y
178	36	N	R	3	1	N	-	-	N	F	T	LPS	T	U	F	BHU	T	A	N	H	C	N	3	F	N	N	Y
179	37	M	R	2	1	N	-	-	N	T	T	GOPS	T	F	F	BSU	T	A	K	H	C	S	2	A	I	P	Y
180	26	F	U	4	0	N	-	-	N	U	T	LOPS	F	F	F	BSU	U	H	N	H	C	I	2	G	I	N	Y
181	47	F	U	3	0	Y	A	LV	Y	F	T	GLOPSYw	T	T	U	BHSU	T	A	K	H	C	I	2	F	I	N	Y
182	21	M	U	3	0	N	-	-	N	T	T	GOPSz	F	F	F	BHS	T	H	H	E	W	S	1	A	D	P	Y
183	75	M	U	4	1	N	-	-	N	F	T	GPS	T	F	F	BU	T	A	K	C	C	I	2	F	P	P	Y

ID	Age	Sex	Res	Edu	Med	Q6	Q7	Q8	Q9	Q10	Q11	Q12	Q13	Q14	Q15	Q16	Q17	Q18	Q19	Q20	Q21	Q22	Q23	Q24	Q25	Q26	Q27
184	31	M	U	3	0	N	-	-	N	F	T	GLOSz	T	F	T	HSU	F	A	N	W	U	I	1	E	D	N	Y
185	61	F	U	2	0	N	-	-	N	F	T	GLOPSz	F	T	T	BHSU	U	H	H	E	C	N	0	F	I	P	Y
186	70	F	U	4	1	N	-	-	N	T	T	GLY	T	F	F	BSU	T	A	K	H	C	A	2	F	D	P	Y
187	24	F	R	2	0	N	-	-	N	F	T	GLOPYz	F	U	U	BHU	T	H	N	H	C	N	0	F	I	N	Y
188	57	F	U	2	0	N	-	-	N	U	U	GOY	T	T	T	S	T	A	K	H	H	S	2	F	N	Y	N
189	37	F	R	1	0	N	-	-	Y	F	F	GOPS	T	T	T	HSU	F	H	K	E	W	A	2	A	D	N	Y
190	58	F	U	1	0	N	-	-	U	F	U	LOY	T	F	F	HSU	T	H	N	E	C	I	3	A	I	P	Y
191	18	M	R	2	0	N	-	-	N	T	U	GLO	U	T	T	BHSU	U	A	N	E	C	I	1	G	I	Y	Y
192	57	M	R	1	0	N	-	-	U	U	T	GPS	U	U	T	BH	F	C	N	H	U	N	2	F	D	Y	Y
193	40	F	R	4	1	N	-	-	Y	F	T	GLOPSY	T	F	F	BHSU	T	A	K	H	C	I	1	F	I	P	Y
194	39	M	R	2	0	N	-	-	N	T	U	GLOP	U	U	T	BS	U	C	H	H	C	I	2	F	I	P	Y
195	74	F	R	4	0	N	-	-	Y	F	F	GLOPY	T	F	F	BSd	U	A	K	H	C	I	2	F	P	N	Y
196	73	M	R	2	0	N	-	-	N	F	F	GOPz	T	U	F	S	T	U	H	H	C	N	1	A	D	N	N
197	64	F	U	2	0	Y	S	GV	Y	F	T	GLOPS	F	F	U	BS	T	H	N	H	C	A	0	A	T	P	Y
198	30	F	R	4	0	Y	S	N	Y	F	T	GLOY	T	U	F	BSU	T	A	K	H	C	I	1	A	I	P	N
199	22	F	R	1	0	N	-	-	Y	F	F	LOPYz	U	F	F	BSU	T	H	N	E	C	A	0	N	I	P	Y
200	39	F	R	2	0	Y	T	NO	Y	U	T	GLOPS	F	U	F	BUd	T	U	N	H	C	S	0	N	I	P	N
201	59	M	U	4	0	Y	A	GLN	Y	F	T	LOPS	T	T	F	BH	T	H	N	H	C	S	2	A	I	Y	Y
202	73	F	R	3	0	N	-	-	Y	F	U	GLO	T	F	F	BU	U	A	H	H	U	I	2	F	I	Y	Y
203	19	M	U	4	0	N	-	-	N	T	T	LOPSY	T	T	F	BHUd	T	A	N	H	W	N	2	A	D	P	Y
204	35	M	U	4	0	N	-	-	N	F	T	GLSY	T	F	F	HSU	T	A	H	E	C	S	1	F	D	Y	Y
205	56	F	R	5	0	N	-	-	Y	T	U	LOPY	T	F	F	SU	T	H	K	W	C	I	1	A	P	P	U
206	50	F	R	1	0	N	-	-	N	T	F	LOP	F	F	T	SU	T	H	N	H	H	S	2	A	I	N	Y
207	45	M	U	3	0	N	-	-	N	T	T	GLPSY	U	F	F	U	T	C	K	H	C	S	1	G	N	P	Y
208	69	F	R	4	0	N	-	-	U	T	U	PS	U	T	F	B	T	H	H	E	U	I	2	F	D	Y	Y
209	43	F	U	2	0	N	-	-	Y	U	T	LSYz	F	F	T	HSU	F	C	K	H	H	N	1	F	T	P	N
210	36	F	R	2	0	N	-	-	Y	T	F	Oz	T	T	F	B	U	A	K	U	H	S	2	A	P	P	Y
211	56	M	U	4	0	N	-	-	N	F	T	LOSz	T	F	F	BHU	T	A	N	H	C	I	1	A	N	P	Y
212	51	F	R	2	0	U	-	V	U	F	T	LOSY	T	U	F	BS	F	U	N	H	H	S	1	G	N	Y	U
213	22	F	U	2	0	N	-	-	N	F	T	GLOPY	T	F	T	BHS	T	A	N	W	C	N	2	A	I	P	Y
214	29	M	U	3	0	U	-	N	N	F	F	LOS	T	T	F	SU	U	A	H	W	C	S	2	A	I	Y	Y
215	74	F	U	2	0	N	-	-	N	F	F	GOSYz	T	T	F	BSU	F	H	N	E	H	S	1	E	P	P	Y
216	47	F	U	4	0	N	-	-	U	U	T	LY	T	T	U	SU	T	A	N	C	C	S	0	A	I	N	N
217	57	F	U	3	0	N	-	-	N	U	U	LO	T	T	T	SU	U	U	K	U	H	S	2	F	D	P	Y
218	46	M	U	5	0	Y	T	V	Y	T	T	GLOSY	T	F	F	BSU	T	A	K	H	C	A	3	A	I	Y	Y
219	48	F	U	1	0	N	-	-	U	T	U	LY	F	U	U	BS	T	H	N	H	C	N	1	F	I	Y	N
220	51	F	U	2	0	N	-	-	N	T	F	GOPY	U	F	U	BHUd	T	A	K	H	C	S	3	F	T	Y	Y
221	36	M	U	4	0	N	-	-	U	U	T	LOSY	T	F	F	S	T	A	N	U	C	S	2	A	I	Y	U
222	57	M	R	3	0	N	-	-	N	F	T	GLOS	F	U	T	BU	F	H	H	H	C	N	1	A	N	N	Y
223	49	F	R	2	0	N	-	-	Y	T	F	OPSY	F	T	T	Sd	T	A	K	H	W	A	0	F	I	P	Y
224	40	F	R	5	0	Y	A	L	Y	F	T	GLP	T	F	F	BHSU	T	A	H	H	C	A	2	A	D	P	Y
225	38	F	U	4	0	N	-	-	Y	F	T	GOPS	T	F	F	BHS	T	H	N	H	C	S	2	A	I	P	N
226	63	F	U	4	0	N	-	-	N	F	T	GLOPY	F	F	F	BHSU	T	A	N	H	C	A	1	F	D	Y	Y
227	39	M	U	4	0	N	-	-	N	U	U	GLOPY	T	U	F	HS	T	H	H	H	C	N	2	F	T	Y	Y
228	43	F	U	1	0	N	-	-	N	T	U	GLOPS	F	F	T	BHSU	U	H	H	U	C	I	2	E	D	Y	Y
229	79	F	U	3	0	N	-	-	N	U	U	GLOPSY	T	U	T	BS	U	A	H	H	C	A	2	A	I	Y	U
230	57	F	U	2	0	N	-	-	Y	U	U	GOPYz	F	F	F	BHUd	T	C	H	C	C	A	3	F	P	P	Y
231	76	M	U	2	0	N	-	-	U	F	T	GLOS	F	F	U	U	U	A	H	C	H	S	2	A	D	P	Y
232	55	F	U	4	0	U	-	Y	N	T	T	GLPSYz	F	F	F	BHSU	T	A	N	H	H	I	2	A	D	N	N
233	77	F	U	1	0	N	-	-	N	U	T	GLP	T	F	F	BU	U	U	H	C	H	S	2	A	I	P	Y
234	35	M	U	4	0	N	-	-	N	T	T	GOPSY	F	T	F	BSU	T	H	K	H	C	S	2	E	I	P	N
235	35	F	U	4	0	N	-	-	N	T	T	GOPSY	T	F	F	BHSU	T	A	H	H	C	I	2	F	D	P	Y
236	42	F	R	1	0	Y	S	V	Y	F	F	LOPYz	U	F	T	S	U	A	H	H	W	A	1	F	I	N	N
237	22	M	R	4	0	N	-	-	N	T	T	GLPS	T	U	F	BS	T	U	N	E	C	A	3	F	D	Y	N
238	27	F	U	2	0	N	-	-	U	T	T	LOP	T	U	F	BHSUd	T	A	N	H	H	S	3	F	I	N	N
239	85	F	R	2	0	N	-	-	N	T	T	LOPY	F	T	T	SU	U	U	H	U	C	I	1	A	D	P	N
240	44	F	U	3	0	N	-	-	U	U	T	GOSYz	F	F	F	BHSU	T	A	H	E	C	S	1	F	D	N	Y
241	40	M	U	1	0	N	-	-	N	F	U	LOPS	F	T	T	B	T	H	N	H	C	A	1	F	D	P	Y
242	23	F	R	2	0	N	-	-	N	T	T	GLOY	T	T	F	BHS	T	C	K	H	W	S	2	G	T	P	Y
243	32	M	U	4	0	N	-	-	N	F	T	LOS	T	F	F	BSU	T	H	K	H	H	S	3	F	P	P	N
244	22	M	U	2	0	N	-	-	N	U	U	GLSz	U	U	U	HSU	F	C	K	H	H	S	1	F	I	N	Y

ID	Age	Sex	Res	Edu	Med	Q6	Q7	Q8	Q9	Q10	Q11	Q12	Q13	Q14	Q15	Q16	Q17	Q18	Q19	Q20	Q21	Q22	Q23	Q24	Q25	Q26	Q27
245	45	M	U	2	0	N	-	-	U	T	U	LOPS	F	T	T	BSU	U	H	N	H	C	N	0	F	T	P	Y
246	44	N	U	4	0	N	-	-	N	F	T	GLPS	T	F	T	HS	T	A	K	H	C	N	3	F	D	P	Y
247	35	F	U	3	0	N	-	-	N	F	T	GLOPSY	T	F	F	BU	T	H	K	H	C	I	1	E	D	P	Y
248	27	F	R	2	0	N	-	-	N	T	U	GLOS	U	U	T	U	T	H	N	H	C	N	2	G	N	N	U
249	84	M	U	4	0	N	-	-	N	U	T	GLOPYz	T	U	F	HSU	U	H	K	H	C	S	3	A	I	Y	Y
250	59	F	R	1	0	N	-	-	N	T	T	LOPSYz	T	F	T	BSU	F	A	K	E	W	I	3	F	D	Y	Y
251	38	M	U	2	0	N	-	-	N	T	U	GLOPSY	T	T	F	SU	U	H	H	U	U	I	1	F	D	N	N
252	20	F	U	2	0	N	-	-	N	F	U	GLOPSY	T	T	F	BHSU	T	A	H	W	C	I	1	A	T	P	N
253	54	M	U	4	0	N	-	-	N	F	T	GLOPSY	T	T	U	BSU	T	H	N	H	C	N	3	N	D	P	Y
254	39	M	R	5	0	U	-	F	N	F	T	GLOPSYz	T	F	F	HS	T	U	H	E	C	S	3	F	I	N	N
255	28	F	R	3	0	Y	A	N	Y	F	T	GLOPSYz	U	T	U	BHS	T	A	H	U	H	I	3	F	T	Y	Y
256	66	F	R	2	0	N	-	-	U	F	U	GLOPS	T	T	F	HSU	U	A	H	H	C	I	3	A	D	N	Y
257	78	F	R	1	0	N	-	-	N	F	U	GLOYw	U	U	T	BHU	U	H	H	H	C	S	0	F	P	N	N
258	58	F	U	2	0	Y	T	NY	N	U	T	GLO	T	U	F	BHU	T	A	N	H	C	A	1	A	Ph	P	N
259	36	M	U	1	0	Y	T	LS	N	F	T	GLOS	T	T	U	SU	T	H	K	H	U	A	2	E	I	P	U
260	30	M	U	3	1	Y	S	N	U	F	F	GLOPSY	T	F	F	BSU	T	A	K	H	C	S	3	F	I	Y	Y
261	34	F	U	2	0	Y	T	NS	Y	F	F	GLOPSY	U	T	F	BHSU	T	A	K	U	C	S	2	A	Ph	Y	Y
262	28	F	U	4	0	N	-	-	Y	F	T	GLOPY	T	F	F	BHSU	T	A	H	U	C	S	3	F	P	P	U
263	63	F	R	4	0	N	-	-	N	F	F	GLOPS	T	T	T	BHSU	T	H	K	H	C	N	0	A	P	P	Y
264	31	M	U	2	0	N	-	-	N	F	U	OPS	T	F	F	BHU	T	H	H	H	C	S	2	F	I	P	Y
265	19	F	R	2	0	N	-	-	N	U	T	GLPS	T	T	U	BU	T	A	N	H	H	S	2	A	Ph	Y	N
266	60	M	U	3	0	N	-	-	N	F	T	GLOPSY	T	F	T	BS	U	H	K	H	C	I	3	A	I	P	N
267	69	F	U	4	0	N	-	-	N	T	T	GLOPSYw	F	F	T	BU	U	A	H	H	H	N	2	N	D	Y	Y
268	35	M	R	2	0	N	-	-	Y	T	T	GLOPSY	T	F	T	HSU	T	U	K	H	U	A	1	F	Ph	P	Y
269	39	F	U	2	0	N	-	-	N	T	T	OPSY	T	U	U	BHSUd	T	U	H	E	W	N	2	F	I	P	Y
270	42	F	R	2	0	Y	T	LOV	N	F	T	LOPSY	T	F	T	BSU	T	U	K	C	U	A	2	F	T	P	Y
271	68	M	R	1	0	N	-	-	N	T	U	OPSY	F	T	T	BHU	T	H	N	H	H	S	2	F	I	N	Y
272	64	F	R	1	0	N	-	-	N	T	T	LPS	T	F	F	BHS	T	A	N	E	C	S	1	F	D	P	Y
273	84	M	U	4	0	N	-	-	U	F	F	OPSYz	F	T	F	SU	T	C	H	H	C	I	1	F	N	P	Y
274	57	F	U	5	0	N	-	-	N	F	U	GLOPSY	T	F	T	BHS	T	A	N	E	C	S	0	A	P	P	Y
275	50	M	U	5	0	N	-	-	N	F	U	GLOPSY	T	F	F	BHSU	T	A	K	W	C	I	2	A	D	Y	Y
276	37	F	U	4	0	N	-	-	N	F	T	GLPY	T	F	F	BSU	T	A	N	H	C	I	3	F	D	N	Y
277	31	M	U	2	0	N	-	-	U	F	T	GLOPSY	T	F	F	BSU	T	H	K	H	C	S	2	A	I	P	U
278	32	F	U	5	0	N	-	-	Y	F	T	LOSY	T	F	F	BHSU	T	H	K	H	C	S	3	F	Ph	P	N
279	25	F	U	4	0	N	-	-	N	F	T	LPY	T	U	F	BHSU	T	A	N	H	H	N	0	F	I	N	Y
280	36	F	U	3	0	N	-	-	N	U	T	GLOY	T	F	F	U	T	U	H	C	C	S	1	A	Ph	P	Y
281	41	F	R	4	0	N	-	-	N	F	T	GLOPS	T	F	U	BHSU	T	U	H	H	W	S	2	F	N	P	Y
282	43	M	U	3	0	N	-	-	N	U	T	OPSY	F	F	U	HSU	T	U	K	E	C	S	2	F	D	P	Y
283	86	M	U	2	0	N	-	-	N	F	U	GLPS	F	U	F	U	U	H	K	H	C	S	2	F	D	N	N
284	49	F	R	2	0	N	-	-	Y	F	T	GLPz	U	F	T	BS	T	H	K	H	C	S	3	G	D	P	U
285	80	F	U	4	0	U	-	OV	N	F	U	GLOPSY	T	F	T	SU	U	A	K	H	C	N	1	F	P	P	N
286	37	F	U	4	1	Y	S	NPV	N	F	T	GLOSY	T	F	F	BHSU	T	A	K	H	C	A	2	A	N	Y	Y
287	46	F	U	1	0	N	-	-	U	F	T	GLOP	T	U	T	BSU	U	U	N	H	W	I	2	F	D	P	Y
288	23	M	R	4	0	U	-	O	U	T	T	GLOPY	T	U	F	HS	T	H	N	H	C	N	1	N	Ph	N	Y
289	81	F	U	4	0	N	-	-	Y	T	T	GLO	T	F	F	BSU	T	A	K	H	C	I	1	E	D	P	N
290	85	F	U	2	0	N	-	-	U	F	U	GLOPYz	T	F	T	SU	T	H	K	H	C	I	1	F	D	N	Y
291	29	M	R	2	0	N	-	-	N	F	T	LOSY	T	F	T	BHSUd	U	U	H	U	W	S	1	A	T	P	Y
292	31	F	U	4	0	N	-	-	N	F	T	LOPS	T	F	F	HSU	T	A	H	H	C	N	2	A	I	Y	Y
293	34	F	R	2	0	N	-	-	N	F	F	GLS	T	F	F	SU	U	A	H	H	W	A	2	F	D	Y	U
294	55	F	R	2	0	N	-	-	N	F	F	GOP	F	T	F	BHSUd	U	H	H	H	W	N	0	E	D	N	Y
295	63	M	R	2	0	N	-	-	Y	F	T	LPSw	T	F	F	BU	U	A	K	H	C	I	2	A	D	P	Y
296	39	F	U	2	0	N	-	-	N	F	T	GLS	T	F	T	BSd	T	C	H	E	C	S	2	G	D	P	Y
297	60	M	U	4	0	U	-	L	Y	T	U	GOS	F	F	F	SU	T	H	H	W	H	A	1	F	D	Y	Y
298	51	F	R	4	0	Y	S	GV	Y	F	U	LOPYwz	T	F	F	BSU	T	A	K	U	C	A	0	G	D	P	Y
299	45	M	U	2	0	N	-	-	N	F	F	GLO	T	U	F	BSU	T	A	N	H	C	N	2	N	Ph	P	Y
300	55	F	U	5	0	N	-	-	Y	T	T	LOPSY	T	F	F	BSUd	T	A	K	H	C	S	2	A	T	Y	Y
301	73	F	R	2	0	N	-	-	N	T	U	GLPSY	U	F	F	BHS	F	H	H	W	H	S	2	F	I	Y	U
302	18	M	R	1	0	N	-	-	N	T	F	PSw	T	T	U	BH	F	H	N	H	C	I	1	A	N	Y	Y
303	79	M	R	3	1	N	-	-	Y	F	U	LOPS	T	F	F	HSU	T	A	K	H	C	A	3	A	I	P	Y
304	29	M	U	4	0	Y	A	F	N	F	T	LPY	T	F	F	BU	T	A	N	H	C	I	3	A	N	P	N
305	50	F	U	2	0	N	-	-	U	F	F	GLO	F	T	F	SU	T	H	H	H	W	I	1	F	N	N	U

ID	Age	Sex	Res	Edu	Med	Q6	Q7	Q8	Q9	Q10	Q11	Q12	Q13	Q14	Q15	Q16	Q17	Q18	Q19	Q20	Q21	Q22	Q23	Q24	Q25	Q26	Q27
306	53	F	U	4	0	N	-	-	U	F	T	GLOPSYw	T	F	U	BSU	T	A	N	C	C	S	0	F	N	P	Y
307	40	F	U	2	0	N	-	-	N	T	T	GOPSY	T	U	U	BS	U	A	H	H	W	S	0	F	N	P	N
308	63	F	U	2	0	N	-	-	Y	F	T	GLOSY	T	F	F	HSU	T	H	K	H	C	A	2	A	T	P	N
309	33	F	R	4	1	N	-	-	N	T	T	GLOS	T	F	U	BSU	T	A	K	H	C	N	3	F	N	Y	Y
310	71	F	U	4	0	Y	S	P	U	F	T	L	T	F	F	BSU	T	C	H	H	C	A	1	A	D	Y	Y
311	77	M	U	4	0	N	-	-	N	F	T	LOYz	F	T	F	BHSU	T	H	K	H	C	I	0	F	D	P	U
312	48	F	U	4	0	Y	S	VY	Y	T	T	GLPS	U	T	F	BU	T	A	H	C	C	A	2	G	D	N	Y
313	34	M	R	1	0	N	-	-	U	T	F	GLOPSY	U	U	F	SU	U	U	K	H	C	A	1	A	T	P	Y
314	25	F	R	4	0	Y	T	LOV	U	F	T	GLOPSz	T	F	F	BHSU	U	A	K	H	C	S	3	A	I	P	U
315	31	M	R	1	0	Y	S	SV	U	T	U	GLO	T	T	F	Ud	T	U	N	C	C	A	1	F	D	N	Y
316	24	F	R	1	0	N	-	-	N	T	F	P	F	U	F	SU	T	C	N	W	C	S	1	A	N	N	N
317	22	F	U	4	0	N	-	-	Y	F	T	LOPS	T	F	F	BU	T	A	N	H	C	I	1	F	I	Y	Y
318	19	F	U	2	0	U	-	N	Y	F	T	GOSY	T	U	T	B	T	C	H	H	W	S	1	E	N	N	N
319	34	N	R	4	0	N	-	-	N	F	T	LPSY	T	U	F	HSU	T	A	H	H	C	I	2	F	I	Y	Y
320	28	F	R	2	0	N	-	-	N	U	T	GLOSz	U	T	F	S	F	A	K	H	C	S	1	A	P	P	N
321	23	F	R	1	0	N	-	-	N	T	T	GOSY	T	F	F	BSU	U	C	N	E	C	S	2	F	Ph	N	Y
322	19	M	R	1	0	N	-	-	N	T	U	GLOPYz	F	T	F	BHSU	T	U	K	H	W	N	1	F	D	P	Y
323	64	F	U	4	0	N	-	-	N	U	T	GLOPSYw	T	F	T	BSU	T	A	N	H	C	N	3	F	N	Y	Y
324	50	F	R	2	1	Y	A	L	U	F	T	GLOPSYz	T	F	F	BHSU	T	A	K	C	C	I	2	N	I	P	Y
325	33	F	R	3	1	N	-	-	N	F	T	GP	T	F	F	SU	T	A	K	H	C	S	3	G	I	P	Y
326	30	F	U	2	0	N	-	-	U	T	T	GLOPS	T	F	U	BHU	T	A	N	E	W	N	2	F	I	N	N
327	65	F	R	2	0	N	-	-	N	T	U	GLPS	T	F	T	BHU	T	H	H	H	C	S	1	A	Ph	P	N
328	32	F	R	2	0	Y	S	LPS	N	T	T	LOP	F	F	F	BSU	T	H	K	H	H	A	2	A	I	P	Y
329	33	F	U	2	0	N	-	-	N	U	T	GLPSY	T	U	F	BS	U	C	H	W	C	N	3	F	D	Y	Y
330	64	F	U	2	0	U	-	LN	N	F	T	GOPz	T	F	T	BHU	U	U	N	H	C	S	1	F	N	N	Y
331	31	M	U	2	0	Y	S	L	N	T	U	LOPS	U	F	U	BHSU	T	A	K	C	C	S	2	F	I	Y	Y
332	85	M	U	2	1	U	-	N	N	T	T	OPSYz	T	F	U	BHSU	U	A	H	W	C	S	2	F	T	Y	Y
333	46	M	U	4	0	N	-	-	N	U	T	GLOPSYw	T	F	U	HSU	T	U	N	W	H	S	2	F	D	Y	Y
334	34	F	U	5	0	U	-	L	N	F	T	GLOPSY	T	F	T	BU	T	H	K	U	W	I	2	N	I	N	Y
335	19	F	R	2	0	N	-	-	N	T	U	OPY	F	F	F	BHU	T	U	K	H	H	S	1	F	I	P	Y
336	59	M	U	4	0	N	-	-	Y	U	T	LOPSY	T	U	F	BHSU	T	A	K	H	C	A	1	F	T	P	Y
337	43	M	U	4	0	Y	T	O	N	F	T	GLOPSY	T	F	F	S	T	A	K	H	C	S	0	F	T	P	Y
338	20	F	R	2	0	N	-	-	N	T	T	GLS	F	T	T	BSU	U	A	K	E	C	S	1	F	D	N	Y
339	56	F	U	2	1	U	-	L	N	F	U	GLOPS	T	T	F	BHSU	T	A	K	W	C	S	3	F	Ph	Y	Y
340	43	M	R	1	0	N	-	-	Y	F	T	GLPSY	U	U	U	BHS	T	C	H	E	H	A	3	N	D	N	N
341	50	F	U	4	0	N	-	-	U	U	T	LOPS	T	F	U	HSU	T	H	N	E	C	S	2	A	D	N	U
342	61	F	R	3	0	Y	S	LPV	Y	F	T	LOPY	T	F	F	BHSU	T	A	H	U	C	A	1	A	P	Y	Y
343	31	F	R	1	0	N	-	-	N	T	U	GOP	T	U	U	BHSUd	T	A	K	H	H	S	1	F	D	P	Y
344	19	M	U	4	0	N	-	-	N	F	T	GLOSY	T	U	F	BSU	T	A	N	H	W	S	1	F	N	Y	Y
345	67	F	U	4	0	N	-	-	N	F	U	GLOPSY	T	F	U	HU	U	U	H	H	W	I	2	F	N	N	Y
346	54	F	R	3	0	U	-	V	Y	T	T	LPSz	T	F	F	BSU	U	H	H	H	C	S	2	A	Ph	Y	U
347	83	M	U	4	0	N	-	-	N	F	T	GLOSYz	T	F	T	BHS	T	U	N	E	C	I	2	F	D	P	Y
348	50	F	U	5	0	N	-	-	U	F	T	GLOPS	T	F	F	BHSUd	T	A	H	H	C	S	2	F	I	N	Y
349	27	F	U	3	0	U	-	V	Y	F	F	LPS	T	F	T	S	U	A	N	H	H	I	1	A	P	Y	Y
350	47	F	U	2	0	N	-	-	U	F	T	GLOSY	T	T	F	U	U	H	K	H	H	S	1	F	T	Y	Y
351	37	M	U	4	0	N	-	-	N	T	T	OSY	T	F	F	HSU	T	A	K	E	C	I	1	A	T	P	Y
352	44	N	U	4	0	Y	T	O	N	F	T	GLPS	T	F	F	HSUd	T	A	K	E	C	I	1	A	D	P	Y
353	29	F	R	2	0	Y	S	GV	Y	F	T	LOPSY	T	F	U	BH	T	H	N	E	C	S	0	A	T	N	Y
354	33	M	R	3	0	N	-	-	N	F	T	GLOPSYz	T	F	F	BHSU	U	A	K	H	C	I	2	A	I	N	N
355	40	F	R	2	0	N	-	-	N	F	U	GLOPS	T	U	T	BSU	T	A	H	U	H	N	2	F	T	N	Y
356	56	M	R	2	0	N	-	-	N	T	T	GLS	U	T	F	SU	T	H	K	H	W	I	2	N	T	N	Y
357	72	F	U	4	0	N	-	-	Y	F	U	GO	T	F	F	HS	T	A	N	H	H	S	0	A	D	Y	Y
358	45	F	U	4	0	N	-	-	N	U	T	GLOPSw	U	T	F	BHSU	T	A	K	H	C	S	2	N	D	Y	Y
359	41	F	U	1	0	N	-	-	N	U	F	LOz	T	T	U	BU	T	H	N	H	C	S	3	A	Ph	P	Y
360	70	M	U	5	0	U	-	F	Y	F	T	LPSY	T	F	F	BSU	T	C	N	C	H	A	2	F	T	P	Y
361	44	F	U	1	0	N	-	-	N	T	F	LY	T	T	T	BSU	T	H	N	H	C	I	2	F	I	N	Y
362	58	F	U	2	0	Y	A	GN	U	F	T	GLPS	U	F	F	BS	U	H	K	H	C	N	2	E	Ph	P	Y
363	49	F	U	2	0	Y	T	LNO	N	F	T	GLOPY	U	T	F	HSUd	U	C	K	W	H	A	1	A	P	Y	Y
364	33	F	U	2	0	U	-	FO	Y	F	T	PYz	T	F	U	BU	F	A	K	W	C	S	2	F	I	Y	Y
365	46	M	R	2	1	N	-	-	U	F	T	LOPSY	T	T	T	BSU	T	A	K	H	W	N	2	F	Ph	P	Y
366	75	F	U	4	0	N	-	-	N	T	T	GOPSY	T	F	T	BUd	T	U	H	H	C	S	0	A	D	P	Y

ID	Age	Sex	Res	Edu	Med	Q6	Q7	Q8	Q9	Q10	Q11	Q12	Q13	Q14	Q15	Q16	Q17	Q18	Q19	Q20	Q21	Q22	Q23	Q24	Q25	Q26	Q27
367	52	M	U	4	0	N	-	-	Y	F	T	GLOPS	T	U	U	BHU	T	A	K	U	C	I	2	A	T	P	Y
368	23	F	U	2	0	N	-	-	Y	F	U	GLOPSY	T	F	F	BU	T	H	H	H	H	S	1	A	D	N	Y
369	57	M	U	2	0	N	-	-	N	T	T	GLOS	U	F	U	HSU	U	A	N	H	H	A	3	F	T	P	Y
370	45	F	R	4	0	U	-	P	Y	T	T	GPSY	F	F	F	SU	T	U	H	H	H	I	2	A	Ph	Y	Y
371	66	F	U	2	0	Y	A	GLV	Y	U	T	LOPS	U	U	F	SU	U	A	H	E	C	I	3	F	I	P	U
372	50	F	U	4	0	N	-	-	N	F	T	LS	T	F	F	BHSU	T	A	N	W	C	I	1	F	I	P	Y
373	51	M	U	5	0	N	-	-	Y	F	F	GLOP	T	F	F	BU	T	A	N	H	C	I	2	A	Ph	N	Y
374	40	F	R	4	0	Y	A	LV	Y	F	T	GLOS	T	F	F	BSU	T	A	K	H	C	A	2	F	D	Y	Y
375	40	M	U	3	0	U	-	V	N	F	T	GLOSY	T	F	U	BHU	T	U	H	E	C	S	1	A	I	P	N
376	53	F	U	5	0	N	-	-	N	F	T	GLOSY	T	F	U	BU	T	U	K	H	C	S	3	F	D	N	U
377	41	F	U	4	1	Y	A	N	Y	F	T	GLOPS	T	F	F	HSU	T	A	H	E	C	I	2	G	N	N	Y
378	32	M	U	4	0	N	-	-	N	T	T	GLP	T	F	F	BHSU	T	H	K	H	C	I	2	F	Ph	P	N
379	21	M	U	3	1	Y	S	P	U	F	T	GLOPY	T	F	F	SU	T	A	K	U	C	N	3	F	D	P	Y
380	18	F	U	2	0	N	-	-	Y	T	U	GLOPS	F	T	T	BHS	T	H	K	E	C	I	1	A	I	P	N
381	71	F	U	2	0	N	-	-	N	F	F	LPSz	T	F	F	BSU	U	H	K	E	C	S	0	A	D	P	Y
382	58	F	R	2	0	N	-	-	N	T	U	GPS	U	T	U	BSd	T	C	N	E	C	N	2	N	T	Y	Y
383	69	F	U	2	0	N	-	-	Y	F	T	LOS	T	F	T	BHSU	T	A	K	H	H	I	2	A	D	P	U
384	42	F	R	4	0	N	-	-	U	F	U	LOPSYw	T	F	F	U	T	A	H	C	C	A	3	F	Ph	P	N
385	64	F	R	3	0	Y	T	NOY	U	F	T	GLOPSY	T	T	T	BSU	T	A	H	H	C	S	1	F	Ph	N	Y
386	65	M	U	2	0	Y	T	L	U	F	T	GPS	F	T	F	SU	U	U	N	E	C	I	0	N	I	P	Y
387	56	F	U	5	0	N	-	-	N	U	T	LOPS	T	T	F	SU	T	A	H	E	C	S	2	A	D	N	Y
388	18	F	U	4	0	N	-	-	N	U	T	GLOP	T	F	U	SU	T	U	N	H	C	N	0	F	P	N	N
389	32	F	U	4	0	N	-	-	N	F	T	LOPS	T	F	F	BHSU	T	A	K	H	W	N	3	F	I	P	Y
390	48	F	R	3	0	Y	T	FV	Y	F	T	GLOS	T	F	F	SU	T	A	K	E	C	S	1	A	I	P	Y
391	27	M	R	2	0	U	-	V	U	F	T	GLOPSw	U	F	U	BSU	T	C	H	E	C	N	2	F	D	Y	U
392	26	F	U	2	0	N	-	-	Y	U	U	GLOPS	T	T	F	BS	U	A	K	H	C	S	3	F	I	Y	Y
393	27	M	U	2	0	N	-	-	Y	F	T	GLOPSY	T	F	F	BHSU	T	A	K	W	C	I	1	E	Ph	P	Y
394	53	M	U	4	0	N	-	-	U	F	F	GLOSz	T	T	T	BSU	T	U	N	H	C	S	3	A	I	Y	Y
395	68	F	U	4	0	N	-	-	U	T	T	GLOY	T	U	T	BSU	T	A	K	H	H	S	1	F	T	P	Y
396	41	F	U	4	0	N	-	-	N	T	U	GLPY	T	U	F	HSU	T	A	K	H	C	S	2	F	N	N	Y
397	63	F	U	4	0	N	-	-	N	U	T	GLOPSYz	T	T	F	BHU	T	A	N	H	W	S	2	F	T	P	Y
398	65	F	U	4	0	N	-	-	N	F	T	GOz	T	F	F	BHSUd	T	A	K	H	C	S	1	A	T	P	Y
399	67	F	U	3	0	N	-	-	U	F	T	LOPSz	U	T	F	BHS	T	A	N	E	C	N	2	F	Ph	Y	Y
400	24	M	U	4	0	N	-	-	N	T	T	GOPY	F	T	F	BU	T	A	N	H	C	N	0	E	P	Y	Y
401	53	F	R	4	0	Y	S	LPV	N	F	U	GLS	T	F	F	BHSUd	T	A	K	H	C	A	1	N	T	Y	Y
402	52	M	R	3	1	N	-	-	Y	F	T	GLP	T	F	F	U	T	A	K	E	C	I	2	N	D	P	Y
403	50	M	U	2	0	N	-	-	N	F	T	L	F	U	U	BSU	T	U	N	H	C	A	1	F	D	Y	U
404	57	F	R	1	0	U	-	Y	N	F	F	LS	T	F	T	BS	U	H	K	W	C	S	2	A	D	P	U
405	54	F	R	2	0	N	-	-	N	T	T	O	U	F	U	BSU	U	H	N	H	C	N	2	A	D	N	Y
406	71	F	U	3	0	N	-	-	U	F	T	GLPSY	U	T	U	HSU	U	A	H	H	C	I	0	F	D	P	N
407	37	F	U	4	0	N	-	-	N	U	F	GLP	T	F	U	BHSUd	F	U	N	H	W	N	2	G	D	N	Y
408	80	M	R	4	0	N	-	-	Y	U	T	GLOPS	T	F	F	SU	T	U	K	C	C	I	2	A	D	Y	Y
409	43	F	R	1	0	Y	S	FG	Y	T	F	GLOPSw	F	T	U	HU	F	H	K	E	H	I	2	A	T	P	Y
410	69	M	U	4	0	Y	T	GV	U	U	T	GLOPSY	T	F	U	BHSU	U	A	N	E	C	S	1	F	T	P	Y
411	71	F	U	2	0	N	-	-	N	F	U	GLOPSw	F	T	F	BSU	T	U	N	H	W	S	2	E	N	P	Y
412	31	F	U	4	0	N	-	-	N	T	F	GLOPSz	T	T	F	BHSU	T	U	N	H	C	N	1	G	P	Y	Y
413	43	M	U	4	0	N	-	-	U	F	T	GLPY	T	T	T	BHSU	T	H	N	H	C	N	1	A	I	Y	Y
414	24	F	U	5	0	Y	T	G	N	F	T	GPS	T	U	F	BU	T	A	K	U	C	S	2	G	D	N	Y
415	64	F	U	4	0	U	-	V	Y	F	U	OPSY	U	F	F	HSU	U	A	H	H	C	I	1	G	I	P	Y
416	25	F	R	5	0	Y	S	L	N	F	T	GOP	T	F	F	BSUd	T	U	K	H	C	A	2	F	D	P	Y
417	69	M	U	3	0	N	-	-	U	F	T	GLOPS	T	U	F	BHSU	U	A	H	H	H	S	1	F	I	Y	Y
418	29	F	R	1	0	Y	T	FOV	Y	U	U	LOPz	T	F	T	BHSU	T	A	N	E	C	I	2	F	D	Y	Y
419	20	F	U	2	0	N	-	-	U	F	U	LOSY	T	T	F	BHSU	T	U	H	E	W	I	0	F	Ph	Y	Y
420	57	M	U	5	0	N	-	-	N	F	T	GLOPS	T	F	F	BSU	T	A	H	W	C	I	1	F	Ph	Y	Y
421	33	F	U	3	0	N	-	-	Y	T	T	GLOY	T	F	U	BHSU	U	A	N	H	W	S	0	A	D	P	Y
422	28	F	U	3	0	N	-	-	N	U	U	GLOPS	T	F	T	BHSU	T	A	H	C	C	A	0	N	I	P	Y
423	49	F	U	4	0	Y	S	L	Y	F	T	GLOSY	T	F	F	HSU	T	H	N	C	C	I	2	A	I	N	Y
424	39	F	R	2	0	N	-	-	N	F	F	LS	T	F	T	HU	T	H	N	H	C	N	0	A	I	N	Y
425	59	M	R	1	0	Y	S	L	U	U	T	GLOSY	T	F	F	S	U	U	N	H	H	I	1	A	Ph	N	Y
426	62	M	U	3	0	N	-	-	N	F	T	OSY	T	F	F	HSU	T	H	K	E	C	S	3	F	T	N	N
427	64	N	R	1	0	N	-	-	N	T	U	LO	F	T	F	BU	T	C	H	H	H	I	1	F	D	P	Y

ID	Age	Sex	Res	Edu	Med	Q6	Q7	Q8	Q9	Q10	Q11	Q12	Q13	Q14	Q15	Q16	Q17	Q18	Q19	Q20	Q21	Q22	Q23	Q24	Q25	Q26	Q27
428	26	M	R	4	0	N	-	-	N	F	T	GLOP	T	F	F	BHU	T	U	H	H	C	S	1	F	I	P	U
429	57	M	U	5	0	N	-	-	N	F	T	GLOPSY	T	F	F	HSU	T	A	N	H	C	N	1	F	D	Y	Y
430	47	M	R	1	0	N	-	-	N	F	F	OPY	F	T	F	BS	T	H	H	E	W	S	0	E	T	P	Y
431	47	F	U	2	0	Y	T	OS	N	F	T	GPSY	T	F	T	BHSU	T	H	N	H	C	S	2	A	T	P	U
432	79	M	U	3	0	N	-	-	N	T	T	GOP	T	U	F	BSU	U	A	H	E	H	I	3	A	Ph	Y	Y
433	48	M	U	3	0	N	-	-	N	T	F	GLOPS	T	F	U	SU	U	A	K	H	C	S	1	F	T	Y	N
434	38	M	U	2	0	N	-	-	N	T	T	GLOPS	T	T	T	BU	T	A	N	H	W	S	2	F	I	Y	Y
435	40	N	R	2	0	N	-	-	N	T	T	GLOP	T	T	T	SU	T	A	N	E	C	S	1	A	D	P	N
436	48	M	U	5	0	Y	S	NV	Y	F	T	GLOPS	T	F	F	BHSU	T	A	N	H	C	I	1	F	D	P	U
437	50	F	U	5	1	U	-	N	N	F	T	GLP	T	F	F	BHU	T	A	K	E	C	I	0	F	D	Y	N
438	49	F	U	1	0	N	-	-	N	T	T	GLOSY	F	T	F	BSU	T	C	H	H	C	I	2	F	I	N	Y
439	58	M	U	1	0	N	-	-	N	T	U	GLYz	U	F	U	HSU	U	H	H	U	C	N	1	F	I	N	Y
440	28	M	U	3	0	N	-	-	N	T	T	LPY	T	T	U	SU	U	H	N	H	C	S	3	G	I	P	Y
441	64	M	U	3	0	N	-	-	U	F	U	GLPS	F	F	F	BHU	T	A	N	H	C	S	3	F	Ph	Y	Y
442	41	F	U	4	0	Y	T	V	Y	F	T	LOPSY	T	F	F	BHSU	T	C	N	H	C	A	3	F	D	Y	Y
443	34	M	U	5	1	N	-	-	N	F	T	LOPY	T	U	F	BHSU	T	A	N	E	C	I	2	N	I	Y	Y
444	41	F	U	2	0	U	-	L	Y	T	T	GLP	T	U	F	U	F	H	H	E	H	A	3	A	T	P	N
445	37	F	R	5	0	N	-	-	U	F	T	GLOP	T	F	F	BHU	U	C	H	H	C	I	3	N	I	Y	Y
446	34	M	U	2	0	N	-	-	Y	F	U	GLOP	T	F	F	BSU	T	A	K	U	C	I	2	F	D	N	Y
447	28	M	U	3	0	N	-	-	N	F	T	GLOSY	T	U	F	U	F	A	H	U	H	N	2	F	I	Y	U
448	60	M	U	2	0	N	-	-	N	T	F	GLOP	F	T	F	BHU	U	U	N	H	C	I	2	F	D	P	N
449	63	M	U	4	0	N	-	-	U	U	T	GLOPS	T	F	F	BUd	F	A	K	E	C	S	3	F	D	N	Y
450	59	M	U	4	0	N	-	-	Y	F	U	GLO	T	T	T	BSU	U	U	H	H	C	I	2	G	Ph	N	N
451	69	F	U	5	0	N	-	-	Y	F	T	GLOPSYz	T	F	F	BU	T	A	K	W	C	A	2	G	I	Y	Y
452	57	F	U	2	0	N	-	-	U	F	T	GP	T	T	F	BS	T	U	N	C	C	S	2	F	D	N	Y
453	46	F	U	2	0	N	-	-	Y	F	U	LOY	U	F	T	HSU	T	A	N	E	U	A	1	F	D	P	Y
454	40	M	R	2	0	N	-	-	Y	T	T	GLOPS	F	F	F	U	F	A	N	H	W	A	2	A	D	P	N
455	70	F	R	2	0	N	-	-	U	F	F	GPw	F	F	F	BHSUd	F	A	N	H	C	N	2	N	I	P	Y
456	50	M	R	2	0	Y	S	GN	Y	F	U	LOPS	T	T	F	BSU	F	H	K	H	C	S	2	F	Ph	Y	Y
457	28	F	R	1	0	Y	T	GLY	Y	F	T	GL	U	F	T	BSUd	T	H	K	E	W	S	0	F	I	P	Y
458	39	M	U	2	0	N	-	-	Y	F	T	GSY	T	F	T	U	T	U	N	H	C	S	2	F	D	N	Y
459	19	F	R	1	0	N	-	-	Y	T	F	GY	T	F	U	-	U	H	K	H	U	I	2	A	I	N	Y
460	59	M	U	5	0	N	-	-	U	U	F	GOSY	T	F	F	BHSU	T	A	K	W	C	N	2	F	D	Y	U
461	58	M	U	3	0	N	-	-	Y	T	T	LOPSz	T	U	F	BU	T	U	K	E	C	I	1	F	D	P	Y
462	31	F	R	2	0	N	-	-	N	F	U	LOSY	T	F	F	S	T	A	N	E	C	I	1	E	D	Y	Y
463	77	M	U	2	0	N	-	-	N	T	U	GLOPS	T	T	F	BU	F	A	N	H	C	I	2	F	D	Y	Y
464	36	F	R	2	0	N	-	-	N	T	U	GLOS	T	U	F	BHSU	F	A	K	E	C	S	1	N	T	P	Y
465	61	F	R	2	0	Y	S	LNS	N	F	U	GLOPS	U	F	T	BHSU	T	A	H	H	W	S	0	F	I	Y	Y
466	52	F	U	4	0	N	-	-	N	U	T	LPSY	U	T	F	BSU	U	C	H	H	C	S	3	A	D	P	U
467	66	M	R	2	0	Y	T	SV	N	F	T	LOPSY	T	F	U	HSU	T	A	K	H	C	S	2	A	I	Y	Y
468	22	F	U	4	0	N	-	-	N	F	F	GL	T	U	F	BHU	T	A	H	U	C	S	0	F	I	P	Y
469	43	M	U	1	0	N	-	-	N	T	T	OSw	T	U	F	U	F	H	N	H	C	I	0	F	I	Y	Y
470	41	F	U	2	0	Y	A	GNP	Y	F	U	LOPSY	T	T	F	SU	T	A	K	W	C	I	2	A	D	N	Y
471	52	F	R	3	0	N	-	-	N	F	T	GL	T	F	F	BHSU	F	A	K	U	H	I	3	F	T	Y	Y
472	42	M	U	4	0	N	-	-	N	U	T	OPSY	T	T	F	SU	T	C	N	U	C	I	2	A	P	Y	Y
473	48	M	U	4	0	Y	S	V	N	F	T	LOPSz	T	T	F	BSU	T	A	K	U	C	I	2	F	N	P	Y
474	72	F	R	5	1	N	-	-	N	F	T	GOPSY	F	F	F	BSU	T	A	K	H	C	N	3	A	D	P	Y
475	59	M	U	2	0	N	-	-	N	F	F	LOPSz	U	U	F	SU	T	U	N	H	H	N	1	F	Ph	Y	Y
476	59	F	U	4	0	Y	S	FP	N	T	T	GPSY	T	F	F	BHSU	T	A	N	W	C	A	3	F	D	N	Y
477	31	M	R	1	0	N	-	-	N	T	T	GLOPYz	T	F	T	HSU	T	H	N	E	C	N	2	A	I	N	Y
478	61	M	U	2	0	N	-	-	N	F	U	GLOPSY	T	F	T	BHd	T	U	K	E	C	A	1	F	D	N	Y
479	80	F	U	2	0	N	-	-	Y	F	U	GOP	T	F	U	BUd	F	A	H	H	C	I	1	A	T	P	Y
480	44	F	U	4	0	N	-	-	U	F	T	GLOPSz	T	F	U	HSU	T	A	H	W	C	S	1	G	I	P	N
481	45	M	U	2	0	N	-	-	N	T	T	GLOPY	T	F	F	BU	U	C	K	H	C	N	3	G	P	N	Y
482	25	F	R	2	1	N	-	-	N	F	U	GLOPSYz	T	F	F	B	T	A	K	H	C	S	2	E	T	P	Y
483	50	F	R	2	0	Y	S	V	U	T	T	GLOPSY	T	U	U	HSU	T	U	K	H	W	N	1	N	P	Y	Y
484	68	M	R	1	0	N	-	-	N	U	T	LPSY	T	T	U	HSU	U	C	N	H	U	S	2	F	N	Y	Y
485	26	M	R	1	0	N	-	-	Y	U	U	GLO	F	F	T	BH	T	H	H	E	W	S	2	F	D	P	Y
486	32	M	U	3	0	N	-	-	U	F	T	GLPSY	T	F	F	U	T	C	K	H	C	S	2	A	Ph	N	Y
487	78	M	U	3	0	N	-	-	Y	F	U	GLPSz	U	T	F	BHSd	T	U	H	H	C	I	2	A	Ph	N	N
488	74	F	R	1	0	N	-	-	N	U	U	GOPSY	U	U	T	H	U	H	H	E	H	S	2	F	D	N	U

ID	Age	Sex	Res	Edu	Med	Q6	Q7	Q8	Q9	Q10	Q11	Q12	Q13	Q14	Q15	Q16	Q17	Q18	Q19	Q20	Q21	Q22	Q23	Q24	Q25	Q26	Q27
489	54	F	R	2	0	N	-	-	N	U	U	OPS	F	T	T	BU	T	A	N	H	C	N	0	A	I	N	N
490	85	F	R	2	0	N	-	-	N	T	T	GLSY	U	T	T	BHS	T	U	N	E	H	N	2	F	I	P	U
491	56	M	U	4	0	N	-	-	N	T	T	OPSw	T	F	F	BS	T	A	K	E	C	S	2	F	I	P	Y
492	49	M	R	1	0	N	-	-	N	T	U	OPSY	U	T	T	BSU	U	H	H	H	C	N	2	E	I	Y	Y
493	82	M	U	5	0	N	-	-	N	F	T	GLOP	T	F	F	BSU	T	H	H	H	W	I	1	G	I	P	Y
494	40	F	U	2	0	N	-	-	Y	U	T	GLOSz	T	U	U	S	T	A	K	E	H	A	2	F	T	P	U
495	55	F	U	2	0	N	-	-	U	F	U	GOPS	F	F	T	HU	T	A	K	H	U	S	2	A	I	P	U
496	34	M	U	2	0	N	-	-	Y	T	U	GLOPSY	U	U	F	BHSU	U	C	N	H	H	A	0	F	D	P	Y
497	85	F	R	3	0	Y	T	G	N	F	T	GL	T	T	F	BSU	T	A	K	H	C	I	1	F	D	P	Y
498	30	F	R	2	0	U	-	L	N	U	T	LOPS	F	T	F	HUd	T	H	H	H	C	I	3	F	I	P	Y
499	37	F	R	4	0	Y	S	FOV	Y	F	T	GLPYz	T	F	F	BHSU	T	H	K	H	C	I	2	N	D	P	Y
500	36	F	R	3	0	N	-	-	N	F	T	GLOPY	T	F	F	BHSU	T	A	K	H	C	N	2	E	Ph	P	U
